# The Art of Writing and Implementing Standard Operating Procedures (SOPs) for Laboratories in Low-Resource Settings: Review of Guidelines and Best Practices

**DOI:** 10.1371/journal.pntd.0005053

**Published:** 2016-11-03

**Authors:** Barbara Barbé, Kristien Verdonck, Deby Mukendi, Veerle Lejon, Jean-Roger Lilo Kalo, Emilie Alirol, Philippe Gillet, Ninon Horié, Raffaella Ravinetto, Emmanuel Bottieau, Cedric Yansouni, Andrea S. Winkler, Harry van Loen, Marleen Boelaert, Pascal Lutumba, Jan Jacobs

**Affiliations:** 1 Institute of Tropical Medicine, Antwerp, Belgium; 2 Institut National de Recherche Biomédicale, Kinshasa, Democratic Republic of the Congo; 3 Institut de Recherche pour le Développement, Montpellier, France; 4 Geneva University Hospitals, Geneva, Switzerland; 5 Department of Pharmaceutical and Pharmacological Sciences, KU Leuven, Leuven, Belgium; 6 JD MacLean Centre for Tropical Diseases, McGill University Health Centre, Montreal, Canada; 7 Department of Neurology, Technical University of Munich, Munich, Germany; 8 Centre for Global Health, University of Oslo, Oslo, Norway; 9 Université de Kinshasa, Kinshasa, Democratic Republic of the Congo; 10 Department of Microbiology and Immunology, KU Leuven, Leuven, Belgium; University of Texas Medical Branch, UNITED STATES

For a clinical study in the European research network on better diagnosis for neglected infectious diseases (NIDIAG) project (Better Diagnosis of Neglected Infectious Diseases: www.nidiag.org), we developed Standard Operating Procedures (SOPs), which we implemented in a basically equipped laboratory in a 380-bed rural hospital (“Hôpital Général de Référence Mosango”) in the Kwilu province in the Democratic Republic of the Congo (DRC). The study aimed to improve the early diagnosis of severe and treatable infections among patients with neurological disorders and took place over a 20-month period (14/09/2012–24/05/2014) (ClinicalTrials.gov Identifier: NCT01589289). The set of 50 SOPs (S1 Appendix), all in French, include procedures related to the inclusion and clinical management of patients with neurological disorders (n = 4), diagnostic testing (n = 33), data collection and management (n = 5), and quality assurance (n = 8).

In this symposium paper, we (i) review current standards and guidelines about writing and implementing laboratory SOPs, (ii) discuss best practices for writing and implementing laboratory SOPs in low-resource settings, and (iii) share some lessons learned in the NIDIAG study in the DRC. This paper targets clinical investigators of Neglected Tropical Diseases (NTDs), but also laboratory managers involved in routine patient care and policy makers developing national laboratory regulations in low-resource settings.

## Why are SOPs important?

SOPs are written step-by-step instructions on how to carry out procedures correctly. SOPs are meant to ensure consistency, accuracy, and quality of data [1]. SOPs harmonize laboratory practices, reduce user errors, and can be used as training tools. Moreover, they help ensure compliance to the study protocol, regulations, and international standards. SOPs are the main building blocks of a laboratory quality assurance framework and are, as such, embedded in the Quality Management System (QMS), which defines and rules the quality organization and management of a laboratory service.

By their nature and objective, SOPs connect to all other building blocks of the QMS, such as organization and personnel, equipment, procurement, process control, biosafety, and corrective and preventive actions. Within a laboratory QMS, SOPs (or procedures) are considered as documents together with policies, processes, and forms [2]. SOPs are subject to version control (e.g., version number and date), review and approval, distribution and implementation, update and revision, and archiving of superseded versions. Training of staff on SOPs (with competence assessment) is an essential QMS requirement and connects the SOPs to the “organization and personnel” building block [2].

## How to write and implement SOPs?

We reviewed QMS documents (identified through an unstructured internet search) that address SOP development. We also searched for evidence about legibility, readability, and comprehensibility of other written documents (i.e., package leaflets of medicines and medical devices). S2 Appendix gives the scope and content of the assessed documents.

### A well-conceived template of the SOP assures completeness and comprehension

QMS standards and guidance documents vary in scope and level of detail about SOP content, formatting, and version control (Table 1). The Clinical and Laboratory Standards Institute (CLSI) QMS02-A6 guideline, the World Health Organization (WHO) Laboratory QMS handbook, and the Strengthening Laboratory Management Toward Accreditation (SLMTA) provide the most extensive information [1–3]. They highlight the importance of a logical and consistent structure and promote the use of a template in order to assure completeness and comprehension [1–2]. Table 2 describes the SOP template recommended by CLSI QMS02-A6. It comprises 17 sections and is, therefore, comprehensive but long. Of note, none of the SOP templates recommended by the assessed QMS documents include a separate section about waste management.

**Table 1:** Overview of laboratory QMS standards and guidance documents and the information they include about writing and implementing SOPs.

Name	Title	Edition	Category	Document type	Job aids	SOP topics	SOP content	Formatting and layout	Readability	Language	Use of graphics	Document control	Review/approval	Availability/distribution	Pretesting	Training	Implementation	Ref
ISO 17025:2005	General requirements for the competence of testing and calibration laboratories	2nd	Nonclinical	International standard	−^a^	+^b^	+	−	−	−	−	+	+	+	−	+	−	[4]
ISO 15189:2012	Medical laboratories—Requirements for quality and competence	3rd	Clinical	International standard	+/−^c^	+	+	−	+/−	+/−	−	+	−	+	−	+	−	[5]
JCI 2010	Accreditation standards for clinical laboratories	2nd	Clinical	International standard and guideline	−	+	+	−	+/−	−	−	+/−	+/−	−	−	+	−	[6]
WHO GCLP 2009	Good clinical laboratory practice (GCLP)	1st	Clinical	International standard	−	+	−	−	−	−	−	+/−	+/−	+	−	+	−	[7]
CLSI QMS01-A4 2011	QMS: A model for laboratory services	4rd	Clinical	International guideline	−	+	−	−	+	−	−	++^d^	++	+	−	+	−	[8]
CLSI QMS02-A6 2013	QMS: Development and management of laboratory documents	6th	Nonclinical	International guideline	+	−	+	++	++	+/−	−	++	++	+	−	+	+	[2]
WHO LQMS handbook 2011	Laboratory QMS handbook	1st	Clinical	International guideline	+	−	+	+	+/−	−	−	++	+	+	−	+	+/−	[1]
SLMTA 2009	SLMTA	1st	Clinical	International guideline	+/−	−	+	+/−	+/−	+/−	−	+	+/−	+	−	+	+/−	[3]

CLSI, Clinical and Laboratory Standards Institute; ISO, International Organization for Standardization; JCI, Joint Commission International; (L)QMS, (Laboratory) Quality Management System; SLMTA, Strengthening Laboratory Management Toward Accreditation; SOP, Standard Operating Procedure; WHO, World Health Organization. Categories are “clinical” (in context of patient care) and “nonclinical” (laboratory work not related to patient care, clinical research). Document types are categorized according to Datema et al. [9]. S2 Appendix gives the scope and content of the documents and their organizations.

^a^ “−” Not mentioned

^b^ “+” Detailed

^c^ “+/−” Mentioned but not detailed

^d^ “++” Extensively detailed

**Table 2:** SOP template with section headings according to CLSI guideline QMS02-A6 2013 [2].

1	Purpose	
2	Scope/applicability	
3	Reagents/media	
4	Supplies/materials	
5	Equipment	
6	Safety precautions	
7	Sample requirements	
8	Quality control	
9	Procedure	
	Qualitative method:	Quantitative method:
10	- Expected results	- Calculations
11	- Interpretation	- Reference interval
12	- Critical values	- Critical values
13	- Results reporting	- Results reporting
14	- Method performance specifications	- Method limitations
15	References	
16	Related documents (forms, job aids)	
17	Attachments/appendices	

CLSI, Clinical and Laboratory Standards Institute; QMS, Quality Management System; SOP, Standard Operating Procedure.

### The art of SOP writing: Legibility, readability, and comprehensibility

Apart from the QMS documents in Table 1, we aggregated additional guidelines for clarity of writing, mostly about writing for patients and health care workers (Table 3). Legibility is defined as the ease with which a reader can recognize the characters and words in a text. It is mainly determined by typography (e.g., font and point size). Readability measures the complexity of words and sentence structure (e.g., numbers of syllables in a word, difficulty of words, and sentence length). Comprehensibility (also referred to as “comprehension”) refers to whether or not a reader understands the intended meaning of a text and is able to draw the correct conclusions. [10]

**Table 3:** Overview of guidance on legibility and readability of labeling and instructions for use of medicinal products and medical devices.

Name	Title	Edition	Required contents	Formatting and lay-out	Readability	Language	Use of symbols/graphics	Document control	Review/approval process	Availability/distribution	Pretesting	Training	Implementation	Ref
ENTR/F/2/SF/ jr(2009)D/869	Guideline on the readability of the labeling and package leaflet of medicinal products for human use	2nd	−^a^	++^b^	++	+^c^	+	−	−	−	++	−	−	[11]
FDA 1993	"Write it right"—Recommendations for developing user instruction manuals for medical devices used in home health care	1st	−	++	++	−	++	−	−	+	++	−	−	[12]
FDA 2001	Guidance on medical device patient labeling; Final guidance for industry and FDA reviewers	1st	+	++	++	−	++	−	−	−	++	−	−	[13]

FDA, United States Food and Drug Administration.

^a^ “−” Not mentioned

^b^ “++” Extensively detailed

^c^ “+” Detailed

The guidelines we assessed give different recommendations on legibility. There is no agreement on font types (with or without serifs) or font sizes: a minimum type size of nine points (font “Times New Roman,” not narrowed) with space between the lines of at least 3 mm (for package leaflets) versus type sizes of 10–14 points (for laboratory documents, user manuals, and medical device labeling) have been recommended [2,11–13]. As for design and layout, emphasis is put on the proper use of white space, headers and templates, and bulleted or numbered lists. Graphics are promoted if they are clear, simple, precise, and depicted at appropriate size and resolution. Drawings are preferred over pictures [12,14]. Box 1 lists the basic principles of SOP writing.

**Box 1. Basic principles on the art of SOP writing**

- Use a unique and meaningful title
- Write in simple language: simple words with a maximum three syllables, sentences with a maximum of 25 words, avoid formal language
- Choose legible font and font size (e.g., Arial, 11 point type)
- Use SOP templates
- Do not use more than two levels of headings
- Use active voice, use “you”
- Turn any list into a bulleted or numbered list
- Put instructions in a logical order
- Be precise, use concrete examples
- Put warnings/precautions before the action
- First put the instruction, then the reasoning
- Stress important information (capitals, bold, colour)
- Avoid abbreviations and acronyms
- Use drawings and place them next to corresponding text
- Drawings are preferred over pictures
- Think about resolution, color, and size
- Use job aids

### Testing for readability

Readability can be assessed by formulas and is expressed as the grade level (years of formal education) needed to easily read the text [10]. Examples are the Simple Measure of Gobbledygook grading (McLaughlin 1969) and the Flesch–Kincaid grade level. Some readability formulas are part of text processing programs such as Microsoft Office Word 2010. Recommended grade levels are a maximum of 6th grade for patient education material [15,16]. The United States Food and Drug Administration (FDA) guidelines recommend 6th or 7th grade [12] up to a maximum of 8th grade level [13] to reach most of the population of the United States. It should be noted that these recommendations are meant for documents written for patients and that the audience targeted by laboratory SOPs is different. Most laboratory staff have a good education level and are trained in understanding technical documents. None of the QMS documents recommend readability testing of SOPs, and to our knowledge, it is rarely practiced for SOPs. Furthermore, the reading level of a text does not reflect its comprehensibility as reading formulas do not take into account the content or the organization of a text.

### Assessing comprehensibility of SOPs through pretesting

To enhance its comprehensibility, each SOP should be pretested and adapted before finalization. Pretesting is the systematic and formal gathering of user reactions after reading a document and is a prerequisite to distribution, training, and implementation [11–13]. The European Commission guideline explains the concept of user testing, discusses the testing of multiple language versions, and describes a method for pretesting package leaflets [11]. The FDA guidelines discuss different methods for pretesting such as focus group interviews, individual in-depth interviews, questionnaire surveys, and operator performance studies [12–13]. Of note, none of the QMS documents cites nor recommends pretesting, except for CLSI guideline QMS02-A6. This guideline mentions document verification as part of the review process (see below), which can be considered as a form of pretesting [2].

### Writing, review and approval of SOPs: Who does what?

An SOP should be written by a person who knows the procedure [2] and, if possible, by the staff that will follow the instructions. The review process should also involve people outside of the writing process, to ensure that the SOP can be used by persons that are not familiar with the topic. CLSI recommends several rounds of reviews by different laboratory staff, focusing on different aspects per review round. CLSI also recommends document verification to ensure that by following the procedure the correct end result is obtained. This can be done, for example, by asking the laboratory staff who were not involved in writing and reviewing to perform the procedure exactly as is written in the SOP [2]. Apart from the review process during the SOP development, revisions are required on a regular basis (e.g., annual or biannual review), while updates may occur at any time, when needed.

Finally, the SOP is approved by the laboratory management, e.g., by circulating a signature page together with the SOP or by an electronic sign-off. SOP approval ensures that the content of the SOP is known to the managers and that they approve the use of the SOP by the staff. SOP approval also allows coordinated and timely implementation of the SOP on-site. An approval procedure should be established, specifying the individuals involved (by their positions or functions) and the order in which approvals are given. [2]

### SOP management

A controlled document master list should be kept by a dedicated document manager, specifying the SOP names and identification numbers, the versions in use, and the effective dates and locations of controlled copies. Copies of SOPs need to be clearly labelled as approved and current versions, while out-of-date versions must be removed from site. The SOP master file contains the current and previous versions of the SOP and serves as the source for generating working copies of current SOPs as well as a historic record, which is useful for audit and inspection purposes. Obsolete versions should be clearly labeled as such, e.g., by means of a notation or stamp. All SOPs need to be stored in a manner that prevents loss, damage, or unauthorized access and should promote easy retrieval. The retention duration of SOPs is defined by regulations, accreditation requirements, study protocol requirements, and the sponsor’s quality system [2].

### Training of involved laboratory staff

Training takes place after approval and before distribution and implementation of the SOP. Group training can be effective, if it allows time for discussion, questions, and answers. Individual hands-on training is recommended for more challenging or unfamiliar techniques and for new laboratory staff. Training records (e.g., sign-in sheets) must be kept either in the SOP master file or a group training file, but also in the individual’s training file [2].

### On-site accessibility and visibility of SOPs and job aids

SOPs must be available where used [2]. For swift retrieval, consulting, and use, SOPs related to laboratory work must be visible and accessible close to the bench rather than in a cupboard in the quality manager’s office. A controlled copy of the original SOPs can be displayed (marked as “copy”).

As to accessibility and visibility, so-called job aids are a valuable adjunct. Job aids are instructions, lists, or quick reference materials derived from the main document and are used when the full procedure is not needed at the time the task is performed [2]. They are designed for direct use at the testing site and are meant to supplement but not replace SOPs [1]. Clear job aids improved health workers’ performance during malaria rapid diagnostic tests (RDTs) [17–19]. Job aids are also subject to document control and can be included or referred to in the “related documents” section of the SOP template [2]. Job aids should be posted in a place that is clearly visible from the work space [1] (e.g., on the wall or on a dedicated display system/document holder).

## What are best practices for writing and implementing laboratory SOPs in low-resource settings?

In low-resource settings, specific factors that may interfere with writing and understanding of SOPs should be anticipated and addressed.

### Language and terminology

Most laboratory staff in low-resource settings are not native English (or French) speakers and are often not expert in the particular domain of care or research; therefore, their literacy level may be lower than anticipated. This language barrier is often not overcome by simple translation into the local language, as words in different languages are not always identical in meaning and function [20]. In addition, producing a high-quality translation is labor intensive [21]. Web-based translation machines may be inaccurate: as an example, Google Translate only had an accuracy of 57.7% for the translation of common medical statements to 26 languages [22]. Apart from interlanguage differences, there is also the issue of terminology. For instance, the “buffer” used for RDTs is also called “blood lysis buffer,” “clearing buffer,” “assay (or sample) diluent,” or “reagent,” and the “specimen transfer device” may be named “tube,” “straw,” or “pipette” [23–24].

In this context, extra care should be taken to adhere to the aforementioned requirements of legibility, readability, and comprehensibility of the SOP and to consistently use simple terms and words. Graphics should be used to simplify the overall message, with preference for drawings [12,14].

### Cultural background

Concepts and symbols can be interpreted differently in different cultures [20,25]. A person’s perception of a symbol varies across cultures [25] but also depends on training, educational level, and professional experience [26]. Furthermore, even if quality systems have become a dominant feature in industrial societies, this is not always the case elsewhere. In particular, a QMS cannot hinge solely on written instructions in an environment with a strong oral culture.

### Barriers to correct use and application of SOPs

Barriers to the correct application of SOPs include misunderstandings because of language or jargon that’s too technical, lack of familiarity with written guidelines, lack of belief that SOPs will improve practice, and lack of motivation to change practice [27]. The number, length, and complexity of SOPs can also be a barrier to writing and implementation, as well as the language issues. In addition, presbyopia (i.e., loss of eye lens accommodation that results in an inability to focus at near distances) tends to occur frequently and at an early age in Africa [28]; it may pass unnoticed and may affect reading, particularly in low light conditions.

To overcome these barriers, ownership by and dialogue with local users is crucial in SOP development and during periodic reviews. Moreover, it is the ethical principle of “collaborative partnership” to engage local researchers and to share the responsibilities within a study [29]. SOPs should be developed on-site to produce best practices in accordance with the available resources [27]. Adequate budget and staff should be allocated to pretest the draft SOPs and to implement them once finalized. A training period should be foreseen [2], and continuous support should be available. Regular exchange with the local users and supportive site visits are indispensable for guaranteeing correct use of SOPs.

## What did we learn about SOP writing and implementation during the NIDIAG study on neurological disorders?

### Outlines and examples of SOP writing

All SOPs were prepared in compliance with a “SOP-on-SOP” and were based on the NIDIAG SOP template. Examples of NIDIAG SOPs are given as supporting information (S1 Appendix, S1 and S2 Figs). The NIDIAG SOP template includes a standardized header, title box, and five section headings: (i) Scope and application, (ii) Responsibilities, (iii) Procedures, (iv) Records and archives, and (v) Document history. For clarity and simplicity, we opted for these five sections only, rather than for the full list of 17 sections recommended by CLSI (Table 2); we also considered that these five sections were the most relevant at the time for all concerned SOPs (also for those SOPs outside the laboratory domain). Of note, we explicitly added “safety precautions” (at the beginning of the procedure and before each step whenever appropriate) and “waste management” as separate headings.

### Training and implementation

During the prestudy phase, the feedback of the study site team helped to refine and adapt the SOPs to the local setting. Once finalized, the SOPs were used for on-the-job training of all staff involved during the on-site pilot of the study. The compilation and version management of the SOPs were handled by the NIDIAG Good Clinical Practice (GCP) focal point, who ensured availability of the most recent versions on the NIDIAG website, allowing for timely distribution to all concerned individuals.

### Feedback from the study site: Challenges and opportunities

With hindsight, setting up and using this extensive SOP system in the NIDIAG project required more time and effort than anticipated. This is in line with the observations on QMS implementation in low-resource settings recently compiled by Luman and coworkers [30]. The large number of SOPs proved to be impractical, and it was difficult to comply with every single one of them. By contrast, job aids were perceived to be very useful in day-to-day research practice. They were provided as a supplement to a number of SOPs and were displayed on the walls in plastic covers (S3 Fig).

Timely review of SOPs proved to be challenging in our study because of unforeseen events (e.g., changed kit contents that had an effect on test procedures). Due to the difficult internet and telephone communication with the remote site, some procedural changes could pass unnoticed. Because of the high number of SOPs, small errors slipped in, causing a series of (minor) revisions, which complicated document control and on-site SOP management. Also, on-site implementation of SOPs did not always happen in time.

Most QMS documents (e.g., CLIA regulations 2011 and CLSI guideline QMS01-A4 2011 [31,8]) have been developed for high-resource settings, hence anticipating fast-track documentation and distribution systems (e.g., by using specific software systems). As these are not available in low-resource settings, one could reconsider some of the QMS requirements. For instance, it could be allowed to adapt SOPs on-site, after discussion with the study quality manager and the sponsor, and the site quality manager’s sign-off. The compilation of small changes could then be adapted at once during the planned periodic revisions of SOPs, resulting in official new versions only at specific time points, thereby facilitating distribution and implementation of these new versions. The key learning points about SOP writing and implementation during the NIDIAG study are summarized in Box 2.

**Box 2. Key learning points**

1. When writing SOPs, think about:
  - Layout: use a template with standardized sections, with a maximum of two levels of headings, and bulleted or numbered lists (the latter for chronological steps)
  - Legibility and readability: use a clear font and font size, simple words, simple sentences, add drawings
  - Comprehensibility: pretest SOPs before finalization
  - Use job aids and display them on/near the bench
2. When considering writing and implementing SOPs for a study in low-resource settings:
  - Engage local staff to develop the SOPs
  - Think about the language of the SOP (English, French, local language)
  - Take extra care in adhering to requirements of legibility, readability, and comprehensibility
  - Take cultural differences into account: different meaning of words, terms, and symbols, the use of written documents in a setting with a strong oral culture
  - Think about possible impaired vision of the user and low light conditions (e.g., use larger print)
3. When implementing the SOPs:
  - Make sure SOPs are distributed in a timely fashion and are accessible to all staff involved
  - Provide training on SOPs for all users and other implicated staff
  - Plan periodic revisions and updating of SOPs in use
  - Provide continuous support and regular site visits

## Conclusion

The development of a set of SOPs is essential for the good conduct of a clinical study, such as the NIDIAG study on neurological disorders. SOPs should be based on a template and kept simple and short, while still including the minimal essential information to perform the task correctly. Efforts should be made to ensure their legibility, readability, and comprehensibility. Graphics (preferably drawings) should be added to aid comprehension. When relevant, safety precautions and waste management should be included as separate sections. The use of job aids is recommended (e.g., displayed on the wall), as they are often more practical than fully detailed SOPs.

The users should be involved in SOP writing, and local development of SOPs—together with the site team—is encouraged. The language barrier, differences in terminology, and the user’s cultural background have to be taken into account. Pretesting of SOPs and staff training need to take place before distribution and implementation of SOPs. Continuous support of local staff and regular site visits are needed to ensure SOP compliance and to allow timely revisions and implementation of SOPs.

## Supporting Information

S1 Appendix SOP Manual—Neurological Syndrome. Set of 50 SOPs used for the NIDIAG study on neurological disorders.
(PDF)

S2 Appendix Overview of the laboratory QMS and other documents assessed for the writing and implementation of SOPs. (PDF)

S1 Fig Example of a NIDIAG SOP using the NIDIAG SOP template, indicating some important characteristics. (PDF)

S2 Fig Extract of SOP-WP2-LAB-35-V2.0-11Apr2014 on how to perform the SD Bioline Malaria Ag PF/Pan RDT (SD 05FK60), showing the interpretation section. Clear drawings, a flowchart, and a decision table are used. The SOP is based on the generic WHO job aid of a malaria RDT (http://www.who.int/malaria/areas/diagnosis/rapid-diagnostic-tests/generic_PfPan_training_manual_web.pdf).
(TIF)

S3 Fig Job aid on the specimen types to be collected during the study. This job aid represents a table summarizing the NIDIAG study specimen numbering information extracted from SOP-WP6-DOC-02-V02.1-18Sep2012, and was put as its annex five. The job aid has a clear title linking it to an approved SOP, thus making it subject to document control. Font type Calibri, and type sizes of 18 and 24 points were used for table text and headers, respectively, allowing the text to be easily read when printed out and posted on a wall. The “bold” font style was appropriately used to highlight the column titles and abbreviations.
(TIF)
