# Supplementary material for: The Art of Writing and Implementing Standard Operating Procedures (SOPs) for Laboratories in Low-Resource Settings: Review of Guidelines and Best Practices
Source: PLoS Negl Trop Dis. 2016 Nov 3;10(11):e0005053. doi: 10.1371/journal.pntd.0005053 (PMC5094690; doi:10.1371/journal.pntd.0005053)
Supplement: S1 Appendix — Set of 50 SOPs used for the NIDIAG study on neurological disorders. (PDF) [file pntd.0005053.s001.pdf]

# SOP Manual

## - Neurological Syndrome –

# Table of contents


| Nr | Syndrome | SOP number | SOP title | Annexes | Page |
|---|---|---|---|---|---|
| **Clinical SOPs** | | | | | |
| 1 | Neuro | SOP-WP2-CLIN-01-V1-18Jun2012 | SOP pour le screening et l'inclusion des patients | No | 1 |
| 2 | Neuro | SOP-WP2-CLIN-02-V1-18Jun2012 | SOP pour l'examen et le suivi clinique | No | 6 |
| 3 | Neuro/Fever | SOP-WP2-CLIN-03-V1-18Jun2012 | SOP pour la réalisation d'une ponction lombaire | No | 14 |
| 4 | Neuro | SOP-WP2-CLIN-04-V02-18Feb2013 | SOP pour les protocoles thérapeutiques | No | 21 |
| **Laboratory SOPs** | | | | | |
| 5 | Neuro/Fever | SOP-WP2-LAB-01-V03-18Sep2012 | Mini anion exchange centrifugation technique (mAECT) | No | 28 |
| 6 | Neuro/Fever | SOP-WP2-LAB-02-V1-30Mar2012 | Simple centrifugation modifiée (MSC) | No | 32 |
| 7 | Neuro/Fever | SOP-WP2-LAB-03-V04-27Dec2012 | Test d'agglutination sur carte *T.b. gambiense* (CATT/*T.b. gambiense*) | No | 35 |
| 8 | Neuro/Fever | SOP-WP2-LAB-04-V1-30Mar2012 | La technique de centrifugation en tube capillaire (CTC) | No | 42 |
| 9 | Neuro/Fever | SOP-WP2-LAB-08-V3-19Mar2014 | Test diagnostic rapide (TDR) de la trypanosomiase humaine Africaine: SD Bioline HAT test | No | 45 |
| 10 | Neuro/Fever | SOP-WP2-LAB-09-V02-11Feb2013 | Test diagnostic rapide (TDR) de la trypanosomiase sur sang total: Gambiense-Sero-K-Set (Coris BioConcept) | No | 49 |
| 11 | Neuro/Fever | SOP-WP2-LAB-20-V02-18Sep2012 | Confection de la goutte épaisse, coloration de Giemsa et examen microscopique | Yes (2) | 53 |
| 12 | Neuro/Fever | SOP-WP2-LAB-21-V01-12Jul2012 | CareStart Malaria pLDH | No | 62 |
| 13 | Neuro | SOP-WP2-LAB-22-V1-12Jun2012 | Comptage de cellules blanches dans le LCR avec la chambre de comptage "Uriglass" (Menarini) | No | 66 |
| 14 | Neuro | SOP-WP2-LAB-23-V02.1-18Sep2012 | Détermination de l'hémoglobine méthode HemoCue Hb 301 | No | 70 |
| 15 | Neuro | SOP-WP2-LAB-24-V01.1-18Jun2012 | Numération et différentiation des globules blancs Système HemoCue WBC DIFF | No | 74 |
| 16 | Neuro | SOP-WP2-LAB-25-V01-12Jul2012 | SD Syphilis 3.0 - SD Bioline | No | 79 |
| 17 | Neuro | SOP-WP2-LAB-26-V01-12Jul2012 | Encre de Chine - Recherche de Cryptocoques dans le LCR | No | 82 |
| 18 | Neuro | SOP-WP2-LAB-27-V01-17Sep2012 | Coloration de Gram | Yes (1) | 84 |
| 19 | Neuro | SOP-WP2-LAB-28-V01-13Aug2012 | Coloration de Ziehl-Neelsen à chaud et lecture microscopique des lames. Détection des mycobactéries sur LCR | No | 94 |
| 20 | Neuro | SOP-WP2-LAB-29-V02-18Sep2012 | Système de détection pour l'antigène latex - Cryptococcose (IMMY) | No | 99 |
| 21 | Neuro | SOP-WP2-LAB-30-V01-17Sep2012 | Syphilis - Test RPR sur cartes Macro-Vue - BD | No | 105 |
| 22 | Neuro | SOP-WP2-LAB-31-V02-18Sep2012 | CrAg Lateral Flow Assay. Système de détection de l'antigène des Cryptocoques (IMMY) | No | 111 |
| 23 | Neuro | SOP-WP2-LAB-32-V01-12Jul2012 | Determine HIV-1/2 | No | 115 |
| 24 | Neuro | SOP-WP2-LAB-33-V01-12Jul2012 | Uni-Gold HIV | No | 119 |
| 25 | Neuro | SOP-WP2-LAB-34-V03-26Dec2012 | DoubleCheckGold HIV 1&2 | No | 123 |
| 26 | Neuro | SOP-WP2-LAB-35-V2.0-11Apr2014 | Test diagnostic rapide (TDR) du paludisme: SD BIOLINE Ag Pf/Pan (SD 05FK60) | No | 127 |

| Nr | Syndrome | SOP number | SOP title | Annexes | Page |
|---|---|---|---|---|---|
| 27 | Common | SOP-WP2-LAB-36-V01-13Aug-2012 | Le prélèvement de l'urine à mi-jet | No | 131 |
| 28 | Neuro/Fever | SOP-WP2-LAB-37-V01-24sep2012 | Liste des tests NIDIAG-Neuro | Yes (5) | 133 |
| 29 | Neuro | SOP-WP2-LAB-38-V2.0-12Sep2013 | Utilisation du Reflotron Plus (Roche) - Analyseur biochimique | No | 154 |
| 30 | Neuro | SOP-WP2-LAB-39-V02-27Dec2012 | Mise en culture du LCR en milieu trans-isolate (T-I) | No | 159 |
| 31 | Neuro | SOP-WP2-LAB-40-V02-27Dec2012 | Mise en culture du LCR en flacons Bact/Alert pédiatriques | No | 162 |
| 32 | Neuro | SOP-WP2-LAB-41-V01-17Sep2012 | Culture des mycobactéries du LCR en milieu BBL MGIT (BD) additionné du supplément d'enrichissement OADC et d'un complexe d'antibiotiques PANTA | No | 164 |
| 33 | Neuro | SOP-WP2-LAB-42-V01-17Sep2012 | Différentiation leucocytaire sur frottis de LCR, coloré au Giemsa | No | 168 |
| 34 | Neuro/Fever | SOP-WP2-LAB-43-V01-17Sep2012 | Recherche de trypanosomes à frais dans le liquide ganglionnaire | No | 171 |
| 35 | Common | SOP-WP2-LAB-44-V01-18Sep2012 | Prélèvements sanguins (hémocultures, sérum, sang héparine et sang EDTA) | Yes (1) | 175 |
| 36 | Neuro | SOP-WP2-LAB-45-V01-17Sep-2012 | Comptage des lymphocytes CD4+ (Dynal T4 Quant Kit/Invitrogen) | No | 181 |
| 37 | Neuro | SOP-WP2-LAB-46-V01-17Sep2012 | Test de glucose (glycémie) - HumaSens glucomètre | No | 186 |
| **Data management SOPs** | | | | | |
| 38 | Common | SOP-WP6-DATA-01-V1.1-18Jun2012 | Procédure pour remplissage des Case Report Forms | No | 189 |
| 39 | Neuro/Fever | SOP-WP6-DATA-02-V1.0-02Dec2013 | Procédure pour la gestion des données en RDC | Yes (6) | 191 |
| 40 | Common | SOP-WP6-DOC-01-V01.1-18Jun2012 | Procédure d'obtention du consentement éclairé | No | 213 |
| 41 | Neuro/Fever | SOP-WP6-DOC-02-V02.1-18Sep2012 | Système de numérotation à utiliser dans les études NIDIAG WP2 | Yes (5) | 217 |
| 42 | Common | SOP-WP6-DOC-03-V01.1-18Jun2012 | Gestion des documents de l'étude | No | 228 |
| **Quality control SOPs** | | | | | |
| 43 | Common | SOP-WP6-QUAL-01-V01.1-09July2012 | Comment écrire les procédures opératoires standards (SOP) | No | 234 |
| 44 | Common | SOP-WP6-QUAL-02-V2-05Dec2012 | External monitoring | Yes (1) | 238 |
| 45 | Common | SOP-WP6-QUAL-03-V1.1-14Jul2014 | Activités de controle de qualité interne | Yes (1) | 252 |
| 46 | Common | SOP-WP6-QUAL-04-V1.1-23Feb2014 | Visites de supervision du laboratoire (GCLP) | No | 264 |
| 47 | Neuro/Fever | SOP-WP6-QUAL-05-V01.1-20Aug2013 | Manipulation et conservation des tests rapides | Yes (2) | 274 |
| 48 | Neuro | SOP-WP6-QUAL-06-V01-24Sep2012 | Comment installer et utiliser le thermomètre "Min/Max"? | Yes (1) | 280 |
| 49 | Neuro | SOP-WP6-QUAL-07-V01-24Sep2012 | Gestion du stock | Yes (2) | 284 |
| 50 | Common | SOP-WP6-QUAL-08-V1.1-05Mar2014 | Gestion des produits périmés et déclassés | Yes (1) | 288 |

| | |
|---|---|
| | **SOP titre :** SOP pour le screening et l'inclusion des patients |
| | **Project/study:** Evaluation of Rapid Diagnostic Tests in association with clinical and laboratory predictors for the diagnosis of Neglected Tropical Diseases in patients presenting with neurological disorders in rural hospitals of Bandundu, Democratic Republic of Congo (NIDIAG WP2-01-NEU) |

# 1. Domaine et application

Les troubles neurologiques peuvent être causés par de nombreuses étiologies (infectieuse, traumatique, vasculaire, ...) et peuvent présenter des manifestations cliniques diverses. Nous définissons ici "troubles neurologiques» la présence de tout symptôme ou signe (seul ou en combinaison) qui suggère une maladie neurologique sous-jacente. Des symptômes ou signes non-neurologiques (par exemple fièvre, ...) peuvent être présents également. Dans cette étude, certaines neuro-infections seront spécifiquement ciblées parce que considérées comme graves mais néanmoins traitables dans un contexte à faible ressource. Les autres étiologies de troubles neurologiques ne seront pas étudiées systématiquement, n'étant pas immédiatement pertinentes dans le cadre de cette recherche. Les patients se présentant avec des troubles neurologiques aux "Hôpitaux généraux de référence" (HGR) participant à l'étude peuvent être éligibles ou non, et peuvent être consentants ou non. Toute une série de symptômes et signes neurologiques a été choisis comme critères d'inclusion pour leur pertinence clinique. Ils sont volontairement assez larges pour tenir compte de la réalité clinique dans les structures de soins primaires et pour capturer la plupart des maladies-cibles. Un patient suspect de trouble neurologique doit être évalué pour son éligibilité dans l'étude au moyen de la « Fiche de Screening » et cela nécessite une anamnèse et un examen clinique succincts. Etant donné que les critères d'inclusion sont très divers et d'évaluation parfois subjective, des définitions précises et des explications détaillées sont fournies pour chacun d'eux dans ce SOP. Le patient ne pourra être inclus dans l'étude que si 1) au moins un des critères d'inclusion est présent et si 2) tous les critères d'exclusion sont absents. Les raisons de non-inclusion dans l'étude seront également analysées. Finalement, comme les troubles neurologiques à investiguer sont le plus souvent sévères et requièrent une ponction lombaire pour une évaluation correcte ainsi qu'un suivi quotidien, seuls les patients acceptant d'être hospitalisés pourront être inclus dans l'étude.

# 2. Responsabilités

| Fonction | Activités |
|---|---|
| *Investigateur Principal du Pays* | ▪ Organisation de la formation des chercheurs du site pour la compréhension de la liste des critères d'inclusion et d'exclusion (avec l'appui du neurologue expert NIDIAG)<br>▪ Suivi de la compréhension adéquate des critères d'inclusion/exclusion<br>▪ Surveillance que le « Registre de Suivi des Patients» (screening/inclusion) est complété régulièrement et lui est envoyé chaque semaine ainsi qu'au Coordinateur de l'Etude |
| *Investigateur Principal du site* | ▪ Screening systématique pour inclusion / exclusion (selon les critères énumérés dans la « Fiche de Screening ») chez tout patient se présentant avec un trouble neurologique<br>▪ Décision d'inclure ou d'exclure le patient dans l'étude |

| | |
|---|---|
| | ▪ Supervision et coaching des co-investigateurs concernant la conformité de leurs décisions avec les critères de sélection |
| *Investigateur(s) du site* | ▪ Screening systématique pour inclusion /exclusion (selon les critères énumérés dans la « Fiche de screening ») chez tout patient se présentant avec un trouble neurologique<br>▪ Décision d'inclure ou d'exclure le patient dans l'étude (en accord avec l'investigateur principal du site) |
| *Moniteur* | ▪ Vérification que seuls les patients répondant aux critères d'inclusion (et sans critère d'exclusion) sont inclus dans l'étude<br>▪ Vérification que les « Fiches de screening » sont correctement remplies et conservées dans le classeur Screening pour une analyse plus approfondie |

# 3. Procédures

## 3.1 Screening des patients suspects de trouble neurologique par l'investigateur du site

- Le personnel médical dans chaque HGR de l'étude et dans les structures de santé primaire en dépendant seront informés à propos des objectifs de l'étude et invités à appeler les investigateurs (ou à leur référer) chaque fois qu'ils seront confrontés à un patient présentant des symptômes/signes évocateurs de trouble neurologique.
- Le screening systématique pour inclusion / exclusion (suite dans ce document) doit être exécuté uniquement par l'investigateur (principal) du site
- Lors de chaque appel auprès du patient, évaluez son éligibilité pour l'étude (avec l'anamnèse et l'examen physique) dans les services ou aux urgences.
- Attribuez au candidat à l'étude un numéro de screening et remplissez les informations requises dans la « Fiche de Screening ».
- Cochez les cases oui/non correspondantes aux critères d'inclusion et d'exclusion. Afin d'assurer un dépistage standardisé et cohérent, les critères sont définis et décrits en détail dans la section suivante.

## 3.2 Critères d'inclusion et d'exclusion

### 3.2.1 Critères d'inclusion

1. <u>Altération de l'état de conscience</u>

Pragmatiquement divisé en quatre stades:

- confusion («altération qualitative»): alerte mais inadéquat dans les réponses simples; discours insensé; désorientation lieu/temps /personne (échelle de Glasgow 14-15/15)
- somnolence («altération quantitative»): idéation anormalement lente, léthargique, mais facilement réveillable avec une stimulation légère (échelle de Glasgow 13-14/15)
- stupeur («altération quantitative»): endormi, difficulté à réveiller, seulement avec une stimulation robuste, mais non douloureuse (échelle de Glasgow 9/15-13/15)
- coma («altération quantitative»): réactif à la douleur ou non (échelle de Glasgow inférieure à 8/15)

La plupart de ces symptômes sont aigus ou subaigus et sont principalement signalés par l'entourage.

2. <u>Changements dans la structure du sommeil</u>

Le patient présente une insomnie la nuit, alors que le sommeil était normal auparavant. Il peut également être endormi, léthargique pendant la journée. Il n'y a pas de restriction spécifique quant à la durée de ces symptômes avant de consulter (plusieurs semaines ou mois), à condition que le symptôme soit toujours présent au moment du screening. Le symptôme peut aussi excéder un an comme on peut le voir dans la THA. Il ne faut pas inclure le patient si le trouble du sommeil est CLAIREMENT lié à un stress familial/social récent (voir critère d'exclusion 3).

3. Déclin cognitif

Le patient développe des troubles cognitifs chroniques qu'il ne présentait auparavant, tels que l'apathie, l'indifférence, des troubles de la mémoire, jusqu'à la démence, ... La plupart du temps, ces symptômes sont rapportés par l'entourage inquiet. Il n'y a pas de restriction spécifique quant à la durée de ces symptômes avant de consulter. Il ne faut pas inclure le patient si le trouble cognitif CLAIREMENT apparu suite à un stress familial/social récent (voir critère d'exclusion 3).

4. Changements dans la personnalité/ comportement

Les changements dans la personnalité du patient, qu'ils soient rapides ou progressifs, sont surtout observés par la famille et sont sources de préoccupation (euphorie anormale, dépression sévère, agitation, des comportements étranges, ...). Il ne faut pas inclure le patient si le trouble du comportement est CLAIREMENT apparu suite à un stress familial/social récent (voir critère d'exclusion 3).

5. Crise(s) convulsive(s) récente(s) (survenues dans les 2 semaines précédant le screening)

Apparition soudaine de rigidité et/ou de mouvements saccadés des bras et des jambes, localisés ou généralisés (avec perte de conscience), avec la dernière crise ayant eu lieu dans les 2 semaines avant le screening, chez un patient qui n'a jamais rencontré ce problème avant l'âge de 6 ans (voir les critères d'exclusion). Morsure de langue, incontinence urinaire et/ou fécale peuvent être observées. En outre, le patient peut présenter des blessures suite à la crise convulsive.

6. Céphalées récentes, sévères et en aggravation

Le patient se plaint de maux de tête qui restent présents tous les jours, qui sont sévères (c.-à-d. empêchant les activités quotidiennes) et qui ne se résolvent pas avec un traitement symptomatique et même dont la gravité augmente (avec ou sans autres symptômes) ; céphalées d'intensité telle qu'elles justifieraient une ponction lombaire aux yeux d'un clinicien pour exclure un processus intracrânien.

7. Méningisme

Les signes classiques de méningisme comprennent des maux de tête sévères et en aggravation (soulagés par la position couchée), une raideur de la nuque (douleur et rigidité nucale, généralement moins marquée à la rotation) et des signes d'hypertension intracrânienne ou d'irritation (nausées / vomissements; phono-photophobie).

8. Lésions des nerfs crâniens d'apparition récente

Apparition récente d'un ou plusieurs symptômes ou signes, pour la plupart localisés à la tête ou au visage tels que troubles auditifs ou visuels, anomalies des mouvements oculaires, anomalies des pupilles, paralysie faciale, ... suggérant une lésion sous-jacente d'un nerf crânien. L'apparition « récente » signifie arbitrairement ayant commencé dans l'année précédant le screening, mais des exceptions (plus longue durée des symptômes) sont acceptables si jugées pertinentes.

9. Déficits sensori-moteurs ou autres signes neurologiques focaux (par exemple dysphasie, ataxie, dystonie,…) d'apparition récente

Le patient se plaint de troubles sensoriels ou moteurs (hypoesthésie, parésie, paralysie, ...) causant des difficultés pour avaler, parler, marcher, saisir des objets ou exécuter des activités physiques. .

L'apparition « récente » signifie arbitrairement ayant commencé dans l'année précédant le screening, mais des exceptions (plus longue durée des symptômes) sont acceptables si jugées pertinentes.

10. Trouble neurologique de la marche (par exemple spastique/ataxique/parétique) d'apparition récente

Le patient a des difficultés qu'il n'avait pas auparavant pour se lever ou marcher, à cause d'une diminution de l'équilibre, de la coordination, de la force motrice ou de la sensation. L'apparition « récente » signifie arbitrairement ayant commencé dans l'année précédant le screening, mais des exceptions (plus longue durée des symptômes) sont acceptables si jugées pertinentes. A noter que le patient ne sera pas inclus si le trouble de la marche a une origine orthopédique évidente.

### 3.2.2 Critères d'exclusion

1. **Enfant de moins de 6 ans**
   Etablissez la date de naissance (certaine ou approximative) du patient en l'interrogeant lui ou un parent. Le profil de morbidité des troubles neurologiques est très différent chez les jeunes enfants et a été davantage investigué. Les jeunes enfants ne représentent donc pas le groupe-cible de cette étude.
2. **Symptôme/signe neurologique clairement lié à un traumatisme récent (accident, ...**): étiologie non-infectieuse évidente.
3. **Symptôme neuropsychiatrique clairement lié à un stress récent social/familial**: les symptômes sont dus à une dépression évidente post-stress ou à une psychose réactive selon l'investigateur. En cas de doute, incluez le patient de toute façon (pour peu qu'il présente un des critères d'inclusion évidemment).
4. **Symptôme neurologique correspondant à une séquelle d'un vieil événement neurologique bien défini** (p. ex accident vasculaire cérébral, ...): l'étude ne vise pas à explorer les séquelles neurologiques à long terme.
5. **Première crise (épileptique) avant l'âge de 6 ans**: une épilepsie idiopathique ou faisant suite à un problème neurologique précoce est dans ce cas beaucoup plus probable.
6. **Incapacité selon l'avis du médecin à se conformer aux exigences de l'étude** (par exemple refus de l'admission à l'hôpital ou d'un suivi clinique régulier).
7. **Refus de donner un consentement éclairé écrit** (soit du patient, soit du tuteur)

***Si au moins un des critères d'inclusion est présent, et que tous les critères d'exclusion sont absents, le patient est éligible pour l'inclusion. Expliquez de façon adéquate les objectifs de l'étude et la procédure (au moyen du « Formulaire d'Information ») Demandez au patient (ou au tuteur) son consentement éclairé (il doit alors signer le «Formulaire de Consentement Eclairé », ou y apposer son empreinte digitale)***

## 3.3 Inclusion des patients dans l'étude

- Si le patient a signé le « Formulaire de consentement éclairé »
  - Complétez la « Fiche de screening »
  - Attribuez un **numéro d'étude** (se terminant par "N", voir SOP-WP6-DOC-02)
  - Remplissez le **formulaire d'identification du patient** (qui restera dans le dossier du patient)
  - **Ouvrez un CRF pour l'étude neuro**

- Archivez la “Fiche de Screening” dans le classeur Screening
- Introduisez le cas dans le Registre de Suivi des patients (répondre « oui » dans colonne « inclus » et compléter comme demandé)

➢ Si le patient n’est pas éligible (pas de critère d’inclusion et/ou au moins un critère d’exclusion, y compris refus de donner son consentement)
  - Complétez aussi la “Fiche de Screening”
  - Archivez la “Fiche de Screening” dans le classeur Screening
  - Introduisez le cas dans le Registre de Suivi des patients (répondre « non » dans colonne « inclus » et donner la raison comme demandé)

**Remarque**: si le patient inclus présente une fièvre prolongée (> 7 jours), il est éligible aussi pour l'étude NIDIAG fièvre à condition qu’il consente à y participer. Attribuez-lui un numéro d'étude « fièvre » (terminé par "F"), remplissez un formulaire d'identification du patient et ouvrez un CRF conçu pour l'étude de la fièvre.

## 4. Définitions et abréviations

- Troubles neurologiques (ou syndrome neurologique): présence de tout symptôme ou signe (seul ou en combinaison) qui suggère une maladie neurologique sous-jacente
- Symptôme: plainte subjective rapportée par le patient (par exemple des maux de tête, sommeil trouble, ..)
- Signe: anomalie objective observée par le clinicien lors de l'examen physique (raideur de la nuque par exemple, ...)
- THA : trypanosomiase humaine africaine
- CRF: “Case Report Form”

## 5. Registres et archives

| **Appendices & Formulaires à compléter** | |
|---|---|
| **Numéro** | **Titre** |
| 1 | Fiche de screening |
| 2 | Registre de Suivi du Patient |
| 3 | Formulaire d’identification du patient |

## 6. Historique du document

*Indiquez les versions précédentes de la SOP et les modifications apportées*

| **Révisions** | |
|---|---|
| *n.a.* | *Première version* |
| | |

| **Nom et fonction** | **Date** | **Signature** |
|---|---|---|
| *Auteurs* | | |
| *Emmanuel Bottieau (coordinateur de l’étude)* | 05 June 2012 | |
| *Revu par* | | |
| *Andrea Winkler (consultante neurologue)* | 14 June 2012 | |
| *Approuvé par* | | |
| *Veerle Lejon* | 18 June 2012 | |

| | |
|---|---|
| | **SOP titre :** SOP pour l'examen et le suivi clinique |
| | **Project/study:** Evaluation of Rapid Diagnostic Tests in association with clinical and laboratory predictors for the diagnosis of Neglected Tropical Diseases in patients presenting with neurological disorders in rural hospitals of Bandundu, Democratic Republic of Congo (NIDIAG WP2-01-NEU) |

# 1. Domaine et application

Les patients inclus dans l'étude NIDIAG syndrome neuro bénéficieront d'un examen clinique approfondi et d'une évaluation ciblée neurologique. En fait, le but-même de l'étude est d'identifier plusieurs prédicteurs cliniques et biologiques permettant de discriminer les maladies tropicales négligées et les maladies infectieuses prioritaires contribuant au syndrome neurologique. Par conséquent, un examen clinique complet et standardisé, en particulier neurologique, est crucial dans cette recherche. Comme la plupart des médecins généralistes se sentent mal à l'aise avec l'évaluation neurologique précise, cette section a été particulièrement développée dans ce SOP sous forme de définitions et d'explications détaillées. Le but est de diminuer autant que possible la subjectivité et les approximations dans la collecte des données cliniques et dans l'interprétation de celles-ci par des chercheurs différents. Pour être complet, ce SOP décrit aussi brièvement le reste de l'évaluation clinique initiale (anamnèse, examen non-neurologique, tests de laboratoire) et le suivi clinique. La CRF a été développé suivant la logique de la démarche clinique et est dans l'ensemble conçu de la manière la plus « auto-explicite » possible. **Il est cependant évident que les soins au patient ont la plus haute priorité ! Si un patient se présente avec des manifestations graves ou potentiellement mortelles, tous les efforts cliniques doivent être faits pour le stabiliser avant d'entreprendre toute la documentation administrative ou de recherche**.

# 2. Responsabilités

| **Fonction** | **Activités** |
|---|---|
| *Investigateur Principal du Pays* | ▪ Organisation de la formation des chercheurs du site concernant l'exécution de l'examen clinique et neurologique et du suivi selon cette SOP (avec le concours d'un expert neurologue)<br>▪ Surveillance du remplissage adéquat des CRFs conformément à cette SOP |
| *Investigateur Principal du site* | ▪ Réalisation d'un examen clinique et neurologique complet et standardisé de tout patient enrôlé dans l'étude, en conformité avec cette SOP<br>▪ Enregistrement des données cliniques initiales et de suivi, tel que prévu dans cette SOP<br>▪ Supervision de l'exécution adéquate de l'examen clinique/neurologique par les co-investigateurs |
| *Investigateur(s) du site* | ▪ Réalisation d'un examen clinique et neurologique complet et standardisé de tout patient enrôlé dans l'étude, en conformité avec cette SOP<br>▪ Enregistrement des données cliniques initiales et de suivi, tel que prévu dans cette SOP |
| *Moniteur* | ▪ Vérification que l'examen clinique et neurologique et le suivi des patients inclus sont effectués en conformité avec cette |

| | |
|---|---|
| | SOP<br>▪ Vérification que les bonnes pratiques cliniques sont respectées<br>▪ Vérification que le CRF est correctement rempli |

# 3. Procédures

## 3.1 Structure du CRF

Le bilan clinique et de laboratoire initial est bien sûr essentiel dans cette étude qui recherche des prédicteurs diagnostiques. Le suivi clinique est tout aussi important car la réponse clinique aux traitements empiriques (ou ciblés) peut aussi constituer un «argument diagnostique » a posteriori en pratique clinique. Ce SOP décrit cette démarche clinique classique.

**La première partie du CRF** (pages 3-16) décrit l'évaluation clinique initiale (Evaluation 1 : démographie, anamnèse détaillée, examen clinique/neurologique complet), que doivent réaliser les investigateurs cliniques. L'examen neurologique sera particulièrement développé ici. En outre, une section spécifique du CRF donne un résumé des principales raisons neurologiques et non-neurologiques d'admission pour permettre à tout clinicien appelé au chevet du malade d'appréhender rapidement la situation du patient (le CRF servant de dossier pour le patient).

**La deuxième partie** (pages 17-26) correspond toujours à l'Evaluation 1 initiale mais rapporte les résultats des tests de laboratoire initiaux (inscrits directement par l'investigateur du laboratoire). Les informations complémentaires venant par la suite des laboratoires de référence seront transmises par texto dès que possible et adjointes au CRF papier.

**La troisième partie** (page 27 de l'Evaluation 1) résume le diagnostic à l'admission, qui peut être soit déjà confirmé (si démonstration d'un agent pathogène) soit le plus souvent encore présomptif. Cette section est également utile pour tout clinicien nécessitant une information rapide sur l'état du patient. L'investigateur clinique doit aussi ouvrir au moment de l'admission une « Fiche de Suivi Clinique » (où tout problème clinique en cours ou survenant par la suite doit être enregistré) et une «Fiche de Médication » (où toutes les données thérapeutiques seront mentionnées), ces deux documents étant essentiels aussi pour le dossier du patient.

**La quatrième partie** (pages 28-36) porte sur les évaluations cliniques par les investigateurs lors du suivi:

- Evaluation 2 : à la sortie de l'hôpital
- Evaluation 3 endéans le mois après la sortie (un contact téléphonique peut suffire si le patient va bien)
- Evaluation 4 six mois après la sortie (ce dernier uniquement pour les patients souffrant de maladies chroniques (THA, VIH, TB) et de séquelles neurologiques encore présentes à l'Evaluation 3)

L'investigateur doit compléter à chaque évaluation le « Fiche de Suivi Clinique » et la « Fiche de Médication » ; il existe aussi dans le CRF un « Formulaire de Visite Non-planifiée » pour toute consultation ou admission imprévue lors de la période de suivi. Une « Fiche de Conclusion de Fin d'Etude » (page 37-39) est à remplir, dater et signer quand l'étude est complétée chez un patient.
A la demande des investigateurs, un formulaire de résumé clinique et de suivi quotidien standardisé à l'hôpital sera disponible dans le dossier papier patient, mais sans faire partie du CRF. Ce document sera toutefois utile au chercheur pour remplir correctement l'Evaluation 2 (à la sortie d'hôpital).

## 3.2 Evaluation clinique initiale (Evaluation 1 dans CRF)

### 3.2.1 Anamnèse

Cette section ne contient pas de difficultés particulières pour l'interprétation par les médecins de l'étude (données démographiques, antécédents médicaux et maladies actives, plaintes principales, trajectoire de référence et traitement reçus/entamés avant l'inclusion, examen clinique général et neurologique détaillés). Pour la partie neurologique, la plupart des signes sont très détaillées ci-dessous (3.2.3 Examen neurologique) pour la raison déjà invoquée.

### 3.2.2 Signes vitaux et examen clinique général

Les signes vitaux et de l'examen général ne posent pas de problèmes spécifiques. Ci-dessous quelques définitions/descriptions des signes cliniques à rechercher, à toute fin utile (dans l'ordre d'apparition du CRF).

| | |
|---|---|
| Déshydratation | Bouche sèche, yeux enfoncés, pli cutané lent; spécifiez sévérité comme requis dans CRF |
| Cachexie | Estimez le "Body Mass Index" (BMI) avec la formule: poids (kg)/taille au carré (m); BMI < 17 kg/m² correspond à cachexie |
| Ictère | Coloration jaunâtre des sclérotiques et/ou peau |
| Pâleur | Aspect pâle des conjonctives, paumes et lits d'ongle |
| Cyanose | Coloration bleuâtre peau et muqueuses |
| Adénopathie localisée | Adénopathie > 2 cm dans une aire; spécifiez localisation et taille |
| Adénopathies généralisées | Adénopathies > 2 cm dans 2 aires ou plus |
| Eruption localisée | Eruption limitée en surface (spécifiez localisation et type: maculaire (même plan que peau) ; papulaire (surélevé) ; vésiculaire (avec contenu liquide) |
| Eruption generalisée | Eruption disséminée sur > 50% du corps (spécifiez type) |
| Oedème localisé | Oedème limité à un ou 2 côtés; spécifiez localisation |
| Oedème generalisé | Oedème (prise du godet) aux jambes, sacrum, paroi abdominal, visage (anasarque) |
| Infection peau/tissus mous | Toute plaie infectée/piqûres d'insecte/abcès/érysipèle/ulcérations |
| Utilisation muscles respiratoires accessoires | Battement des ailes du nez, rétraction intercostale |
| Auscultation pulmonaire anormale | Spécifiez et localisez: sibilances, crépitations, ronchi, hypoventilation<br>Mentionnez percussion si anormale |
| Distension veines du cou | Jugulaires turgescentes en position à 30° |
| Anomalie cardiaque | Présence de bruits anormaux (spécifiez, localisez: soufflé, gallop) |
| Hépatomegalie | Foie palpé en dessous du rebord costal (taille en travers de doigts) |
| (Autre) masse abdominale | Présence d'une tuméfaction abdominale anormale (taille et localisation) |
| Palpation abdominale douloureuse | Spécifiez la localisation et l'intensité de la douleur |

### 3.2.3 Examen neurologique

Comme mentionné précédemment, cette partie est plus élaborée et destinée particulièrement aux investigateurs qui ne sont pas familiers avec la neurologie. Chaque signe clinique est décrit et expliqué dans l'ordre d'apparition du CRF, avec pour but d'uniformiser l'interprétation.

| Fonctions supérieures | Définitions/explications |
|---|---|
| Niveau de conscience | Pragmatiquement divisé en quatre stades:<br>• confusion («altération qualitative»): alerte mais inadéquat dans les réponses simples; discours insensé; désorientation lieu/temps /personne (échelle de Glasgow 14-15/15)<br>• somnolence («altération quantitative») : idéation anormalement lente, léthargique, mais facilement réveillable avec une stimulation légère (échelle de Glasgow 13-14/15)<br>• stupeur («altération quantitative»): endormi, difficulté à réveiller, seulement avec une stimulation robuste, mais non douloureuse (échelle de Glasgow 9/15-13/15)<br>• coma («altération quantitative»): réactif à la douleur ou non (échelle de Glasgow inférieure à 8/15) |
| Orientation (temps, lieu, personne) | Le patient connaît-il la date d'aujourd'hui? Le patient sait-il où il est et pourquoi il est là? Le patient sait-il dans quel village il habite ? |
| Comportement et discours/action | Le patient se comporte-t-il dans la norme culturelle ou a-t-il une attitude étrange? |
| Cognitive: simple arithmétique (?) | Demandez au patient de compter jusque 10, et de revenir à zéro (dépend du niveau d'éducation). |
| Cognitive: mémoire ancienne | Demandez au patient quand il est allé à l'école, quand il s'est marié (événements du passé). |
| Cognitive: mémoire récente (5 mots) | Demandez au patient ce qu'il a mangé ce matin, comment il a voyagé jusque l'hôpital,… (événements récents). Prononcez 5 mots et demandez au patient de les répéter quelques minutes après. |
| Examen tête et cou | |
| Raideur nuque flexion | Prenez la tête du patient dans vos mains et inclinez-la doucement vers l'avant. Observez la résistance et la réaction du patient. |
| Raideur nuque rotation | Prenez la tête du patient dans vos mains et tournez-la doucement de gauche à droite et inversément. Observez la résistance et la réaction du patient. |
| Examen nerfs crâniens | |
| Acuité visuelle (compter doigts) | Des formes (ou lettres) standards doivent être lues à haute voix, à différents distance. Ou demandez de dire le nombre de doigts que vous montrez. |
| Champs visuels | Asseyez-vous en face du patient et demandez-lui de se couvrir un oeil. Placez un doigt tendu à mi-chemin entre vous et le patient, et déplacez-le dans les 4 quadrants du champ visuel de la périphérie vers le centre. Demandez au patient d'indiquer quand il voit apparaître le doigt. Votre propre champ visuel sert de contrôle. |
| Mouvements yeux: paralysie nerfs (III, IV, VI) | Notez la localisation des yeux dans la position neutre (oeil vers le bas ? vers le haut ?) Demandez ensuite au |

| | |
|---|---|
| | patient de suivre votre doigt avec ses yeux dans les 8 directions (haut, bas, gauche, droite, et les diagonales dans les 4 quadrants) et de vous indiquer quand il voit le doigt en double. Notez la position des yeux quand le patient se plaint de double vision. |
| Mouvements yeux: paralysie regard (haut, bas, latéral) | Demandez au patient de suivre votre doigt avec ses yeux dans les 4 directions principals (haut, bas, gauche, droit), et regardez ses yeux. Est-ce que la poursuite douce est interrompue ? Y a-t-il un paralysie du regard (des 2 yeux) ou un nystagmus (yeux oscillants) ? Si oui notez la position. |
| Mouvements yeux: poursuite douce | Voir ci-dessus |
| Pupilles de taille égale? | Regardez les pupilles à la lumière du jour et notez leur taille, forme et éventuelle différence entre les 2 yeux. |
| Pupilles réactive lumière (directe)? | Utilisez une lumière et regardez si la pupille ipsilatérale se contracte. |
| Pupilles réactives lumière (indirecte)? | Utilisez une lumière et regardez si la pupille contratérale se contracte. Assurez- vous que la lumière éclaire seulement une pupille en protégeant l'oeil opposé. |
| Accomodation des pupilles? | Dites au patient de regarder votre doigt à une distance que vous rapprochez, et regardez si les pupilles se contractent. |
| Ptose | Affaissement de la paupière. Demandez au patient de regarder vers le plafond. Comparez toujours avec l'autre côté. |
| Hypoesthésie faciale | Caressez le visage du patient de la tempe vers le front, de la joue vers l'angle de la bouche et de l'angle de la mâchoire vers le menton. Comparez avec l'autre côté. |
| Réflexe masseter | Posez votre doigt sur le milieu du menton et tapez sur votre doigt avec un marteau réflexe. |
| Paralysie faciale (haute, centrale) | Demandez au patient de faire un sourire "forcé" et regardez s'il y a une asymétrie musculaire. Le mouvement des muscles du front est intact. |
| Paralysie faciale (basse, périphérique) | Demandez au patient de faire un sourire "forcé" et regardez s'il y a une asymétrie musculaire. Le mouvement des muscles du front n'est pas intact; il y a asymétrie. Les paupières ne se referment pas complètement sur l'oeil. |
| Trouble de l'audition | Frottez le pouce contre l'index juste à proximité de l'oreille du patient et demandez s'il entend. Notez s'il y a une asymétrie. |
| Nystagmus (spontané) | Présence de mouvements en saccades des yeux en position neutre (patient regardant en face de lui). |
| Nystagmus (provoqué) | Voir ci-dessus (mouvement des yeux et paralysie du regard) |
| Dysarthrie | Est-ce que la prononciation est laborieuse quand vous demandez au patient son nom (comme s'il avait bu)? |
| Dysphonie | Est-ce que le patient a une voix enrouée ou particulièrement aiguë ? |
| Dysphagie | Le patient a-t-il des difficultés pour avaler? S'étrangle-t-il quand on lui donne du liquide? |
| Réflexe luette | Est-ce que le patient a la nausée quand vous touchez |

| | |
|---|---|
| | le pharynx postérieur avec une spatule? |
| Mouvement langue | Quand le patient tire la langue, est-elle déviée sur le côté ? Peut-il la bouger d'un côté à l'autre ? |
| Examen moteur | |
| Atrophie musculaire | Examinez le patient sans habit et recherchez si certains muscles sont fins. L'atrophie peut être focale ou généralisée. |
| Hémiparésie/hémiplégie | Vérifiez si le patient peut bouger les 4 membres et notez toute difficulté pour bouger. Si c'est d'un côté uniquement, on parle d'hémiparésie. S'il n'y a aucun mouvement possible d'un côté, on parle d'hémiplégie. Dans tous les cas, effectuez toujours un testing de la force muscle par muscle. |
| Quadriparésie/quadriplégie | Vérifiez si le patient peut bouger les 4 membres et notez toute difficulté pour bouger. Si les 4 membres sont atteints on parle de quadriparésie ou de quadriplégie (paralysie complète). Dans tous les cas, effectuez toujours un testing de la force muscle par muscle. |
| Déficits moteurs localisés | Vérifiez si le patient peut bouger les 4 membres et notez toute difficulté pour bouger. Si un seul groupe de muscles est affecté, on parle de déficit moteur. La force musculaire doit être quantifiée pour chaque muscle (voit Tableau dans CRF). |
| Tonus | |
| Hypotonie | Bouger passivement les bras et jambes du patient au niveau des articulations et jugez le tonus (= résistance aux mouvements passifs). Est-il diminué? |
| Hypertonie (spasticité, rigidité) | Bouger passivement les bras et jambes du patient au niveau des articulations et jugez le tonus (= résistance aux mouvements passifs). Est-il augmenté? |
| Examen des réflexes primitifs | |
| Palmo-mentonnier | Grattez l'éminence thénar du patient et regardez la présence de contraction au niveau du menton. |
| Moue | Placez votre index sur la bouche du patient et tapez sur votre doigt avec le marteau réflexe. Y a-t-il un mouvement vers l'avant exagéré? |
| Réflexes tendineux profonds/plantaires | Utilisez la Figure pour grader chaque réflexe |
| Réflexe tricipital | Placez votre doigt sur le tendon du triceps juste au-dessus de l'épicondyle médian et frappez dessus avec le marteau |
| Réflexe bicipital | Placez votre doigt sur le tendon du biceps dans la fosse antécubitale et frappez dessus avec le marteau. |
| Réflexe radial | Placez votre doigt sur le radius latéral distal et frappez dessus avec le marteau. |
| Réflexe rotulien | Localisez le tendon du quadriceps entre le tibia et la rotule et frappez avec le marteau. |
| Réflexe achilléen | Localisez le tendon d'Achilles juste au-dessus du talon et frappez dessus avec le marteau. |
| Réflexe plantaire | Grattez doucement la partie latérale de la plante du pied du talon jusqu'au petit orteil et puis vers le gros orteil. Observez si le gros orteil s'étend ou se fléchit et si les orteils se recourbent |
| Extension des orteils | Durant la manoeuvre du réflexe plantaire, observez si les orteils s'étendent. |

| Coordination et examen de la marche | |
|---|---|
| Marche | Observez la marche du patient. Est-elle instable? Le patient tombe-t-il d'un côté? Balance-t-il les bras? |
| Test doigt-nez | Avec les yeux fermés, le patient doit toucher son nez avec l'index. Y a-t-il hésitation? |
| Test talon-genou | Avec les yeux fermés, le patient doit toucher son genou avec son talon et le descendre vers la cheville. Y a-t-il hésitation ? |
| Tremblement | Regardez si les patient tremble des mains, pieds ou tête. Si oui, cela survient-il au repos, lorsqu'il bouge, ou lorsqu'il veut saisir un objet? |
| Examen sensibilité | |
| Hypo/anesthésie localisée | Glissez votre doigt sur différentes parties des bras et jambes du patient et comparez avec la même zone du côté oppose (ex: bras latéral supérieur, partie supérieure et inférieure de la jambe et du pied). Au niveau du tronc et de l'abdomen, comparez gauche et droite. Recherchez les niveaux sensitifs en glissant le doigt du pelvis jusqu'au cou et demandez au patient quand la sensation redevient normale. |

### 3.3 Evaluation de suivi

Selon les cas, 2 ou 3 évaluations de suivi (clinique seulement) sont prévues durant l'étude

- A la sortie de l'hôpital
- Endéans un mois après la sortie
- Six mois après la sortie

Ces évaluations sont destinées à investiguer l'évolution et l'issue des patients étudiés ainsi que tout nouvel événement, durée des symptômes et réponse aux traitements successifs. Il n'y a pas de difficulté particulière dans cette section du CRF.

## 4. Définitions et abréviations

BMI: Body Mass Index
CRF: Case Report Form
GCS: Glasgow Coma Scale

## 5. Registres et archives

| **Appendices & Formulaires à compléter** | |
|---|---|
| **Numéro** | **Titre** |
| 1 | Case Report Form |

## 6. Historique du document

*Indiquez les versions précédentes de la SOP et les modifications apportées*

| **Révisions** | |
|---|---|
| *n.a.* | *Première version* |
| | |

| Nom et function | Date | Signature |
|---|---|---|
| *Auteurs* | | |
| *Emmanuel Bottieau (coordinateur de l'étude)* | 14 June 2012 | |
| *Revu* | | |
| *Andrea Winkler (consultante neurologue)* | 14 June 2012 | |
| *Approuvé par* | | |
| *Veerle Lejon* | 18 June 2012 | |

| | |
|---|---|
| nidiag Better Diagnosis of Neglected Infectious Diseases | **SOP titre :** SOP pour la réalisation d'une ponction lombaire |
| | **Project/study:** Evaluation of Rapid Diagnostic Tests in association with clinical and laboratory predictors for the diagnosis of Neglected Tropical Diseases in patients presenting with neurological disorders in rural hospitals of Bandundu, Democratic Republic of Congo (NIDIAG WP2-01-NEU) |

## 1. Domaine et application

Les patients présentant des troubles neurologiques requièrent la plupart du temps un examen du liquide céphalo-rachidien (LCR), car celui peut être perturbé en cas de pathologies cérébrales et/ou méningées. Dans les contextes à ressources limitées, cet examen est parfois le seul accessible pour évaluer une maladie neurologique. L'analyse du LCR peut fournir de précieux renseignements pour les cliniciens grâce à la mesure directe du taux de glucose ou de protéines, ou la démonstration de cellules sanguines (avec quantification et différentiation), de parasites (trypanosomes) ou de divers agents pathogènes (via différentes colorations spécifiques). Dans des contextes plus favorisés, des examens tels que des cultures du LCR, des analyses moléculaires ou histologiques peuvent fournir des informations supplémentaires. A noter enfin que la mesure de la pression d'ouverture du LCR peut aussi avoir une valeur ajoutée pronostique et thérapeutique.
Le prélèvement du LCR et la mesure de la pression d'ouverture ne peut se faire que par ponction lombaire (PL). Cette technique peut être très sûre, voire même relativement confortable si une expertise minimale et des conditions adéquates sont réunies. Il existe toutefois aussi certains risques, parfois sévères. Dans l'étude NIDIAG neuro, cette investigation sera nécessaire pour la décision thérapeutique chez presque tous les patients enrôlés, sauf s'il y a des contre-indications spécifiques (voir ci-dessous). Parce que la qualité du prélèvement du LCR est cruciale, ce SOP est complètement dédié à cette technique, qui ne pourra être appliquée aux patients de l'étude NIDIAG neuro que par les investigateurs faisant partie de l'équipe NIDIAG.

## 2. Responsabilités

| Fonction | Activités |
|---|---|
| *Investigateur Principal du Pays* | ▪ Organisation de cours de recyclage pour les cliniciens de l'étude qui effectueront les PL, la mesure de la pression d'ouverture et le prélèvement de LCR sur les sites d'étude<br>▪ Surveillance du respect de ce SOP la de la réalisation des PL |
| *Investigator Principal du site* | ▪ Réalisation adéquate (contre-indications, techniques) de la PL, de la mesure de pression d'ouverture et du prélèvement du LCR selon les recommandations de ce SOP<br>▪ Supervision du caractère adéquat des procédures opératoires réalisées par les co-investigateurs |
| *Investigateur(s)* | ▪ Réalisation adéquate (contre-indications, techniques) de la PL, de la mesure de pression d'ouverture et du prélèvement du LCR selon les recommandations de ce SOP |
| *Moniteur* | ▪ Vérification du respect de ce SOP (vis-à-vis de la réalisation de la PL et de ses contre-indications, de la mesure de la pression d'ouverture et du prélèvement de |

| | |
|---|---|
| | LCR)<br>▪ Vérification que les bonnes pratiques cliniques sont respectées (par les investigateurs et les infirmiers assistants |

# 3. Procédure

## 3.1 Vérifier d'abord les contre-indications à la ponction lombaire (PL)

➢ La ponction lombaire (PL) **ne doit pas** être effectuée dans les situations suivantes:

- Risque imminent d'engagement cérébral
  Une PL en présence d'une hypertension intracrânienne peut provoquer une herniation cingulaire, uncale ou foraminale (selon la localisation de la lésion cérébrale) avec arrêt cardio-respiratoire. C'est pourquoi en contexte favorisé, un CT Scan cérébral et un examen ophtalmoscopique sont obligatoires avant toute PL. Dans le contexte limité de l'étude, la décision ne pourra être basée que sur des critères cliniques et ophtalmoscopiques. En principe donc une PL est contre-indiquée formellement si un patient présente
  - ➢ une altération de l'état de conscience se dégradant rapidement
  - ➢ une altération de l'état de conscience ET un œdème papillaire à l'ophtalmoscopie
  - ➢ dans le décours immédiat d'une crise convulsive (< 30 minutes)
  - ➢ lors d'état de mal épileptique.
- Diathèse hémorragique (saignements diffus)
- Infection cutanée au site de ponction
- Etat de choc ou dyspnée sévère
- Graves déformations vertébrales

Un traitement empirique d'emblée est dans ces cas préférable.
La raison pour laquelle une PL n'est pas effectuée chez un patient inclus doit être mentionnée dans le CRF (page)

La ponction lombaire (PL) doit être effectuée **avec prudence,** en décidant au cas par cas, et seulement après consultation avec l'investigateur principal dans les situations suivantes:

- Altération de l'état de conscience (surtout si coma aréactif)
- Œdème papillaire à l'examen ophtalmoscopique
- Signes neurologiques focaux (déficit moteur, convulsions)
- Autres symptômes suggérant une hypertension intracrânienne (vomissements, céphalées intenses,..)

**Toutefois, chaque fois qu'une méningite est suspectée (fièvre, signes méningés,...), même en la présence d'un des signes/symptômes mentionnés ci-dessus, le rapport bénéfice-risque justifie quand même une PL.**

## 3.2 Phase préparatoire

Seuls les investigateurs NIDIAG peuvent effectuer des PL chez des participants de l'étude. Il faut dans ce cas respecter les consignes suivantes:
- Expliquer la procédure au patient et/ou à son tuteur, y compris les éventuels effets

secondaires post-PL (et leur prévention et traitement)
- Obtenir le consentement oral avant de procéder.
- Demander l'assistance d'un infirmier
- Contrôler avec l'assistant que l'unique tube de collecte en plastique (15 cc), stérile et avec bouchon à vis est correctement étiqueté (voir SOP sur l'étiquetage des échantillons)
- Se laver les mains avec du savon et de l'eau, ou un désinfectant si disponible

## 3.3 Matériel à rassembler

Matériel pour prélèvement stérile (gants stériles, masque et lunettes en plastique pour l'opérateur)
Aiguille à PL, stérile calibre 20 ou 22 (voir Figure 1 ci-dessous)
Trousse de perfusion stérile (mesure pression d'ouverture)
Alcool éthanol à 70%
Povidone Iodine à 10% (ou alcool iodé à 2%)
Eventuellement aiguille SC (calibre 22 ou 25 g) avec seringue de 5 cc et lidocaïne 1% pour anesthésie locale
Compresses stériles 10 x 10 cm
Pansement adhésif
Conteneur à « biohazard »

Figure 1: aiguille à PL (avec stylet)

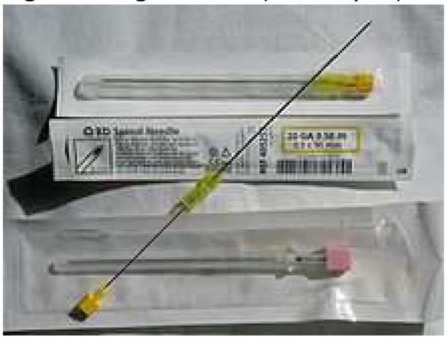

## 3.4 Technique/procédure

- **<u>Placez le patient</u>** en décubitus latéral, dos en face de vous, dans une position genu-pectorale (cou en flexion - position fœtale), la tête installée sur un oreiller (Figure 2). La position assise est également possible pour les patients pas trop malades (moins de risque de hernie), mais la pression du LCR ne peut pas être mesurée dans ce cas (Figure 3). L'infirmier assistant explique aussi et aide le patient à se mettre en bonne position.
- Trouver les crêtes iliaques postérieures, tracez une ligne fictive, palper **<u>l'apophyse épineuse de L4</u>**, et marquez l'endroit d'un trait d'ongle.

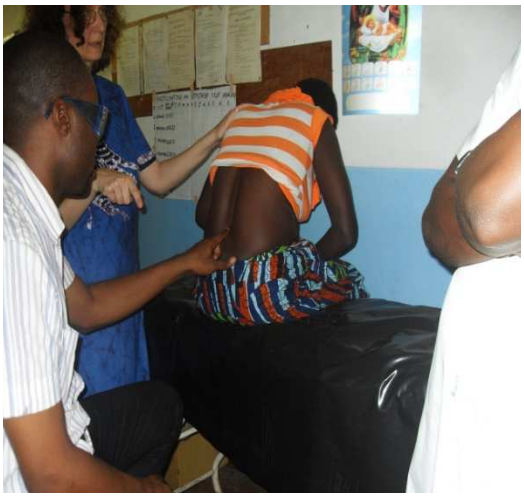

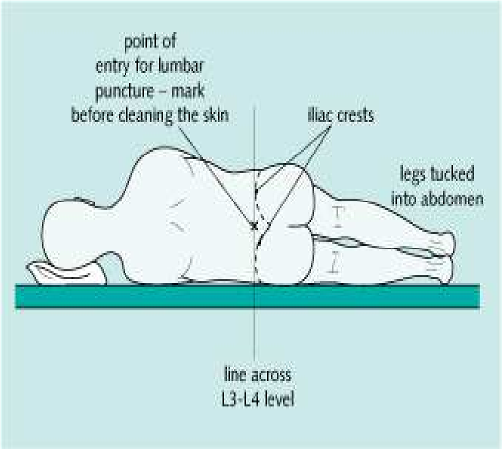


Figure 3

Figure 2

- **<u>Désinfectez la peau</u>** (ou demandez à l'infirmier assistant de le faire) avec une compresses imbibée d'alcool éthanol à 70% pendant une minute puis avec une autre compresse imprégnées de povidone iodine à 10% pendant 2 minutes (procédure identique au prélèvement d'hémoculture, dans un but de standardisation). Commencez par le site de ponction et effectuez des cercles concentriques de plus en plus étendus jusqu'aux crêtes iliaques (Figure 4).

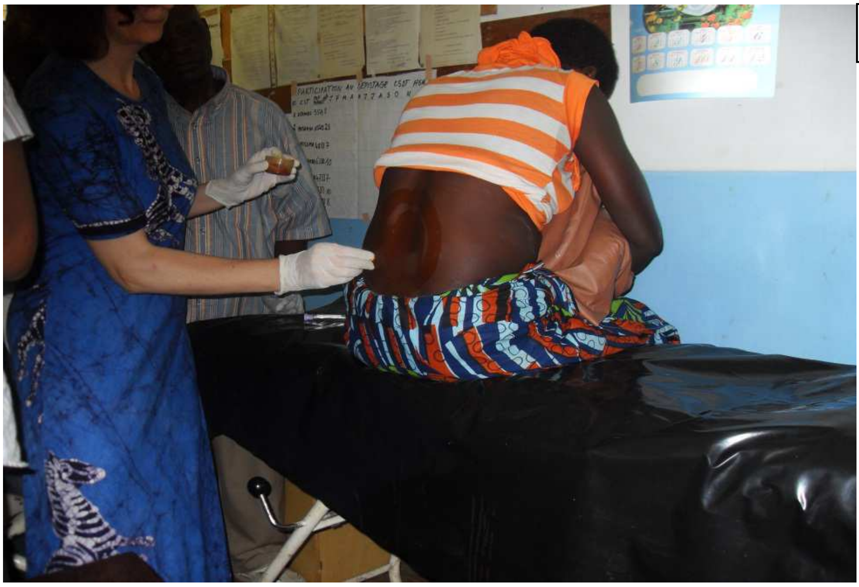

Figure 4

- Mettez les **<u>gants stériles</u>** et les lunettes de protection ; installez le paquet inversé des gants sur le lit derrière le patient pour disposer d'un champ stérile.
- Dans le cas où la PL pourrait être malaisée (agitation, anxiété,…), envisagez une **<u>anesthésie locale</u>** ; demandez à l'infirmier assistant dans ce cas de déposer une aiguille sous-cutanée (25 g), une aiguille puiseuse et une seringue de 5 cc. Aspirez de la lidocaine 1% d'un flacon tendu par l'assistant et désinfecté au préalable (avec l'iode povidone). Anesthésiez localement la peau et l'apophyse épineuse, et attendez 2-3 minutes.

- Demandez à l'infirmier assistant de déposer l'aiguille à PL. **<u>Insérez l'aiguille à PL</u>** dans la ligne médiane et la pointe dirigée vers l'ombilic du patient (Figures 5 et 6)

Figure 5

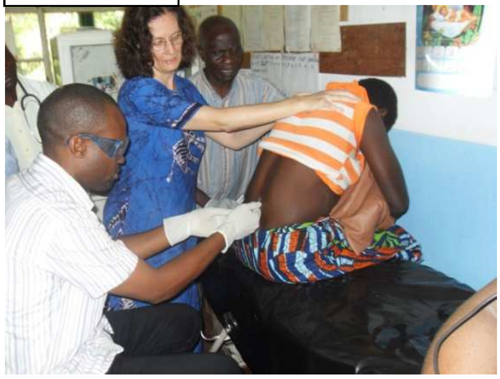

Figure 6

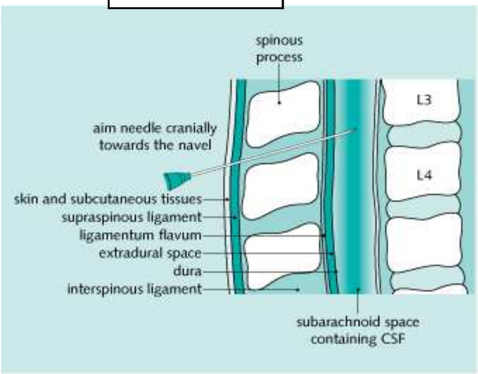


- Faites progresser lentement l'aiguille spinale vers l'avant, pendant environ 2 cm ou jusqu'à ce qu'un **<u>« pop » soit ressenti</u>** (correspondant à la perforation du ligament jaune et de la dure-mère). Avancez ensuite en retirant le stylet tous les 2-3 mm pour vérifier le retour du LCR (Figure 7)

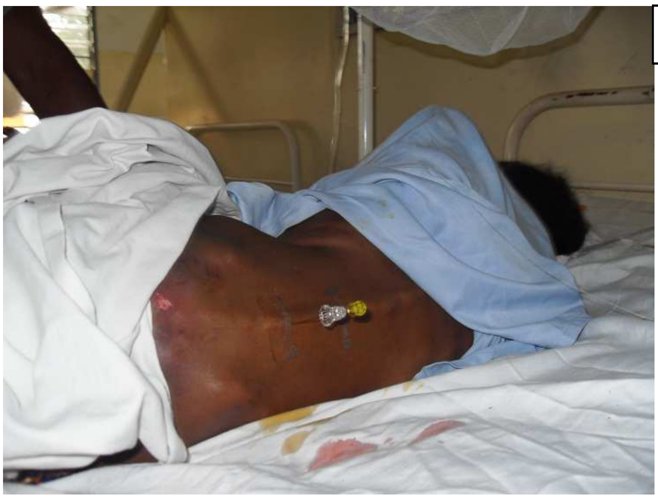

Figure 7

- Si l'aiguille rencontre l'os, retirez-la de quelques mm lentement et essayez à nouveau. Si du sang apparaît (ponction traumatique), retirez l'aiguille et comprimez le point de ponction. Réessayez avec une nouvelle aiguille dans l'espace inter-épineux sous-jacent. Si une nouvelle ponction est jugée non-faisable, prenez soin d'envoyer seulement le dernier tube pour la numération cellulaire.
- Lorsque le LCR commence à couler de l'aiguille (parfois lent s'il est épais et purulent), écartez les premières gouttes avant de mesurer la pression. Ne jamais aspirer le LCR car une racine nerveuse peut être blessée.
- Mesurez la **<u>pression d'ouverture</u>** au moyen d'une trousse à perfusion stérile. Vissez l'embout sur l'aiguille à PL (avec le robinet d'abord fermé). Etirez le tube verticalement (Figure 8 et 9) et ouvrez le robinet. Laissez le LCR monter dans le tuyau et se stabiliser. Demandez qu'on mesure avec une latte le niveau atteint, c.-à-d. la «distance» en cm entre le niveau d'insertion et le niveau atteint par le LCR (normale <20 cm d'eau).

Figure 9

Figure 8

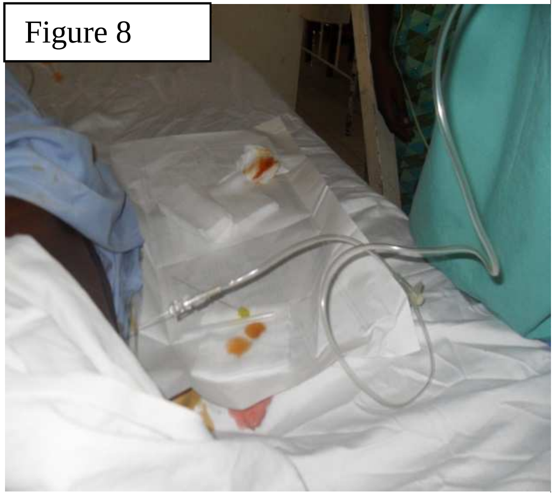

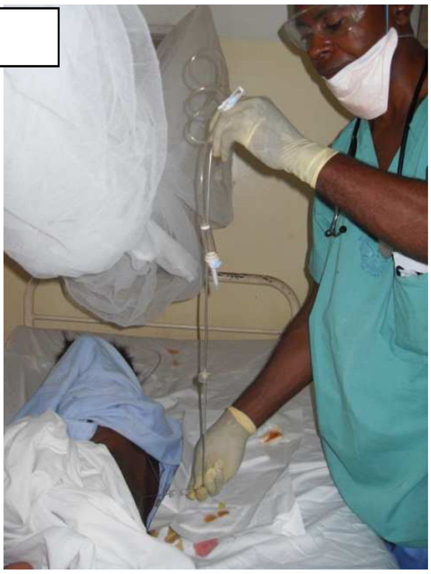

- ➢ **<u>Faites couler le LCR</u>** de la trousse de perfusion vers le tube collecteur stérile (de 10 à 15 cc). Complétez si nécessaire avec des gouttes supplémentaires de LCR.
- ➢ **<u>Retirez l'aiguille à PL</u>** sans replacer le stylet et appuyez légèrement avec du coton. Jetez l'aiguille dans le conteneur à objet tranchant/aiguille (appelé internationalement à « biohazard ») ; ne re-capuchonnez jamais l'aiguille !
- ➢ **<u>Apposez un pansement</u>** sur le point de ponction.
- ➢ **<u>Envoyez ou portez-vous-même DIRECTEMENT</u>** le tube au laboratoire (crucial pour la numération des cellules du LCR et la détection de trypanosomes).
- ➢ **<u>Notez dans le CRF</u>** le jour et l'heure de la PL, la qualité du prélèvement (traumatique ou non), le volume prélevé, la mesure de la pression d'ouverture et tout incident survenu lors du prélèvement.
- ➢ Demandez au patient de **<u>rester allongé</u>** pendant au moins 4 heures.

## 4. Risques et complications de la PL

- **<u>Céphalées post-PL</u>**
  Ces maux de tête peuvent se produire chez 10% à 30% des patients endéans 1 à 3 jours après la PL et peuvent durer de 2 à 7 jours (rarement plus). Il s'agit d'une douleur céphalique pulsatile, avec ou sans nausée, soulagée par la position couchée et aggravée par la position debout et les manœuvres de « Valsalva » telles que la toux et les efforts de défécation. Elle est toujours temporaire. Le traitement consiste en un repos au lit avec hydratation, ainsi que des analgésiques simples (type paracétamol). Un épidurale avec sang autologue est rarement nécessaire (sauf si l'on suspecte une fuite de LCR persistante).
- **<u>Traumatisme d'une branche nerveuse radiculaire</u>**
  Cela peut causer une douleur temporaire ou des picotements dans la peau innervée par la racine nerveuse touchée (parfois dans la jambe). Cet effet secondaire est sans danger et il en faut en avertir le patient au préalable pour diminuer l'anxiété, s'il devait se produire. Une faiblesse persistante ou une perte de sensation sont extrêmement rare car l'extrémité de la moelle épinière se situe bien au-dessus du niveau de la ponction lombaire.
- **<u>Hernie cérébrale</u>**
  Cette complication fatale (engagement suivi d'arrêt cardio-respiratoire) ne survient que

lorsque la pression intracrânienne est augmentée. C'est la raison pour laquelle les contre-indications potentielles doivent être soigneusement examinées avant une PL. Certains experts estiment toutefois que de tels événements ne représentent qu'une que pure coïncidence dans le temps, liée à la pathologie sous-jacente plutôt qu'à la ponction lombaire elle-même.

- **Infection cutanée ou épidurale**
  C'est extrêmement rare, si les contre-indications locales ainsi qu'une stricte asepsie sont respectées. Le traitement consiste en la désinfection de la plaie une antibiothérapie systémique couvrant les bactéries cutanées.
- **Saignement médullaire ou épidural**
  C'est aussi une complication extrêmement rare si la procédure est correctement effectuée. Le traitement est en principe supportif.

Au cours de l'étude NIDIAG, toute complication liée à une ponction lombaire doit être signalée dans le CRF (dans la partie « Fiche de Suivi Clinique).

## 5. Définitions & abréviations

- PL: ponction lombaire
- LCR: liquide céphalo-rachidien
- CT: Computed Tomography
- CRF: Case Report Form
- SC: sous-cutané

## 6. Registres et archives

| **Appendices & Formulaire à compléter** | |
|---|---|
| **Nombre** | **Titre** |
| 1 | Case Report Form |

## 7. Historique du document

*Indiquez les versions précédentes du SOP et les modifications apportées*

| **Révisions** | |
|---|---|
| *n.a.* | *Première version* |
| | |

| **Nom et fonction** | **Date** | **Signature** |
|---|---|---|
| *Authors* | | |
| *Emmanuel Bottieau (coordinateur de l'étude)* | 14 June 2012 | [illegible] |
| *Revu par* | | |
| *Andrea Winkler (consultante neurologue)* | 14 June 2012 | [illegible] |
| *Approuvé par* | | |
| *Veerle Lejon* | 18 June 2012 | [illegible] |

| | |
|---|---|
| | **SOP titre :** SOP pour les protocoles thérapeutiques |
| | **Project/study:** Evaluation of Rapid Diagnostic Tests in association with clinical and laboratory predictors for the diagnosis of Neglected Tropical Diseases in patients presenting with neurological disorders in rural hospitals of Bandundu, Democratic Republic of Congo (NIDIAG WP2-01-NEU) |

# 1 Domaine et application

Parmi les troubles neurologiques observés chez les patients admis dans les HGR de l'étude, une certaine proportion (non encore connue) sera causée par une ou plusieurs maladies graves et traitables spécifiquement ciblées par l'étude NIDIAG. Il s'agit pour rappel (1) de la **trypanosomiase humaine africaine** (THA), (2) de la **malaria cérébrale**, (3) des **méningites bactériennes**,(4) de la **neurotuberculose**, (5) de la **neurosyphilis**, (6) de l'**infection VIH** et (7) de la **cryptococcose méningée (liée au VIH)**. La difficulté réside dans le fait que beaucoup de ces pathologies peuvent se présenter sous n'importe quel symptôme neurologique d'inclusion, même si certains tableaux cliniques et biologiques (résultats de ponction lombaire) sont parfois davantage évocateurs de telle ou telle étiologie. Certaines autres maladies non investiguées prioritairement (comme la toxoplasmose cérébrale liée au VIH) seront également abordées quand il est possible de les traiter dans le contexte de l'étude.

Ce SOP est destiné à standardiser l'approche THERAPEUTIQUE des pathologies-cibles. Il ne s'agit pas de réécrire des guidelines complets déjà existants, mais bien de les adapter au contexte rural congolais, en fonction des disponibilités, monitoring nécessaire, coûts,… Il y a 3 raisons pour élaborer ce SOP de standardisation thérapeutique: (1) une volonté d'utiliser la meilleure approche thérapeutique actuelle, y compris coût-bénéfice, en fonction des (maigres) données épidémiologiques (2) l'importance d'évaluer la réponse clinique à des (mono)thérapies spécifiques car ce sont des arguments diagnostiques importants a postériori en pratique clinique et (3) la nécessité éthique pour NIDIAG de participer aux coûts des traitements des pathologies-cibles. A noter que pour les problèmes neurologiques fréquents (convulsion,…), les protocoles (« ordinogrammes ») congolais seront utilisés.

Il est toutefois clair que l'étude ne pourra prendre en charge financièrement que les pathologies cibles diagnostiquées chez les patients répondant aux critères d'inclusion (qu'ils aient consenti ou non à participer) ainsi que leur complication neurologique immédiate.

Signalons enfin qu'il est impossible en clinique d'envisager toutes les situations qui peuvent se présenter. Des traitements différents ou complémentaires restent bien entendu possibles selon le jugement du clinicien chercheur. Tout écart thérapeutique important avec les protocoles devra néanmoins être mentionné dans la rubrique conçue à cet effet dans le CRF (faisant directement suite à la rubrique diagnostics présomptifs/confirmés).

# 2 Responsabilités

| Fonction | Activités |
|---|---|
| *Investigateur Principal du Pays* | ▪ Organisation de la formation et surveillance des chercheurs du site pour l'utilisation et le respect du SOP thérapeutique concernant les pathologies-cibles du NIDIAG |
| *Investigateur Principal du Site* | ▪ Respect des protocoles thérapeutiques élaborés et justification écrite dans le CRF de tout écart ou ajout important |

| | |
|---|---|
| | ▪ Enregistrement adéquat des traitements instaurés (dans la « Fiche de Suivi Clinique » et la « Fiche de Médication »<br>▪ Supervision des co-investigateurs concernant le respect de ce SOP |
| *Investigateur(s) du Site* | ▪ Respect des protocoles thérapeutiques élaborés et justification écrite dans le CRF de tout écart ou ajout important<br>▪ Enregistrement adéquat des traitements instaurés (dans la « Fiche de Suivi Clinique » et la « Fiche de Médication » |
| *Moniteur* | ▪ Vérification que les protocoles thérapeutiques sont respectés (CRF) et que les « Fiches de Suivi Clinique » et « Fiches de Médication » sont correctement remplies |

# 3 Procédures

## 3.1 Approche syndromique

Les patients se présentent avec des plaintes et pas des diagnostics. La recherche NIDIAG va permettre une démarche diagnostique standardisée et approfondie pour tout patient de plus de 5 ans avec un trouble neurologique.

Le Tableau ci-dessous résume (très sommairement) les grands syndromes de présentation neurologique et les étiologies les plus fréquentes (parmi les pathologies prioritaires NIDIAG ; la toxoplasmose cérébrale est ajoutée car son traitement présomptif est simple). Ce Tableau ne donne pas un ordre de probabilité ( !) mais est purement indicatif pour la démarche

| **Syndromes** | **Etiologies possibles (parmi les pathologies-cibles)** |
|---|---|
| Déficits sensorimoteurs (origine cérébro-pontine, atteinte nerfs crâniens, myélite, radiculite, névrite) | Neurotuberculose<br>THA<br>Toxoplasmose cérébrale (SIDA)<br>Abcès cérébral bactérien/méningite compliquée<br>Cryptococcose (SIDA)<br>(Infection VIH)<br>(Neurosyphilis) |
| Crise convulsive | Malaria cérébrale<br>Méningite bactérienne/abcès cérébral<br>Neurotuberculose<br>Toxoplasmose cérébrale (SIDA)<br>Cryptococcose (SIDA)<br>(THA)<br>(Neurosyphilis) |
| Syndrome méningé | Méningite bactérienne<br>Neurotuberculose<br>Cryptococcose (SIDA) |
| Encéphalite | THA<br>Malaria cérébrale<br>Toxoplasmose cérébrale (SIDA)<br>(Neurosyphilis)<br>(Encéphalite herpétique) |

| | |
|---|---|
| Détérioration cognitive (démence)/ troubles comportement | THA<br>Neurotuberculose<br>Infection VIH (démence SIDA)<br>Neurosyphilis (tardive)<br>Cryptococcose (SIDA) |
| Céphalées sans anomalie neurologique somatique | THA<br>Malaria (cérébrale)<br>Meningite/encephalite<br>Neurotuberculose<br>Cryptococcose (SIDA)<br>Toxoplasmose cérébrale (SIDA)<br>(Neurosyphilis) |

On constate que presque chaque pathologie peut se présenter avec presque tous les syndromes (et c'est d'autant plus vrai pour les cas atypiques). A noter que l'absence de fièvre n'exclut pas les neuro-infections. Aussi le coma peut se présenter à un stade préterminal dans toutes les pathologies-cibles…

Il est néanmoins possible de construire une certaine approche probabiliste, avec le tableau de présentation, les résultats de laboratoire initiaux (pléiocytose neutrophilique/lymphocytaire) et les résultats des tests rapides (malaria, VIH, syphilis,…)

La conséquence en est qu'il n'est pas nécessaire de donner tous les traitements à tout le monde. Si l'état du patient n'est pas trop inquiétant une approche séquentielle des traitements est souhaitable pour observer les réponses aux (mono)thérapies spécifiques. En cas de situation alarmante (coma), en particulier chez le patient VIH, plusieurs traitements concomitants sont cliniquement justifiés, mais la réponse clinique au traitement sera malaisée, voire impossible.

## 3.2 Pathologies-cibles et protocoles de traitement NIDIAG

- Les protocoles suivants ont été élaborés en incorporant les recommandations nationales des programmes du Ministère de la Santé et la connaissance épidémiologique locale (quand disponible). Quand des recommandations nationales n'étaient pas disponibles, les protocoles ont été basés sur des ouvrages de référence suivants : Oxorf Handbook of Tropical Medicine, 3rd Edition 2008 ; District Clinician Manual , IMAI-WHO, draft 2009 ; Clinical HIV/AIDS Care Guidelines for Resource-poor Settings, MSF-OCB 2006 ; Clinical Guideline, MSF Edition 2010. Ils ont ensuite été adaptés après une visite préparatoire approfondie sur le site d'étude (faisabilité, disponibilité et coût).

- Le prix par protocole thérapeutique (tel que payé par le patient) est mentionné à titre indicatif, sur la base des prix des médicaments obtenus à Mosango en Mai 2012 (lors de la vite préparatoire). Chaque aiguille à ponction lombaire coûte 800 francs congolais (FC), c.-à-d. 1 USD.

- Comme mentionné, ces protocoles ne peuvent couvrir toutes les situations potentielles qu'on observe en clinique. Les événements particuliers et atypiques devront être gérés avec le jugement clinique des chercheurs.

| Pathologies prioritaires | Diagnostic présomptif (P) ou confirmé (C) | Protocole NIDIAG | Prix indicatif total pour patient (avec cathéter,…) |
|---|---|---|---|
| THA second stade | C: Présence de trypano dans LCR, OU dans | Traitement du PNLTHA: NECT c.-à-d. | Gratuit via le PNLTHA |

| | | | |
|---|---|---|---|
| | sang/ganglions avec leucocytes > 5/µL LCR<br><br>P :<br><br>Soit CATT + ET leucocytes > 5/µL LCR ET pas d'autre cause<br><br>Soit trypano dans sang/ganglion MAIS LCR normal (leucocytes < 5/µL<br><br>(A DISCUTER au cas par cas avec superviseurs pays et/ou IMT) | Eflornithine (DFMO) 200 mg/kg 2x/jour IV en 2 h. pendant 7 jours et Nifurtimox 15 mg/kg en 3 doses par jour pendant 10 jours<br><br>NB1 : si symptômes neuro, CATT + et LCR anormal (sans démonstration de trypanosomes), faire titration du CATT : au-delà de 1/8, pas de traitement immédiat à Mosango (à discuter toutefois avec superviseurs en fonction gravité des symptômes) MAIS suivi médical et biologique rapproché ( toutes les deux semaines au début puis tous les trois mois).<br><br>NB2 : si symptômes neuro, présence de trypanosomes dans le sang et LCR normal, en principe traitement comme premier stade (pentamidine) MAIS discussion préalable avec superviseurs pays et/ou IMT ; décision clinique en fonction sévérité des symptômes<br><br>NB3 : A partir de février 2013, possibilité de référer des patients éligibles pour l'étude fexinidazole de DNDI à Masi-Manimba, dans le cadre d'une convention avec l'équipe de recherche DNDI | A Mosango et/ou à Masi-Manimba (30 min voiture ; USD) |
| Malaria cérébrale | P : Diagnostic malaria par goutte épaisse ou TDR du PNLP : ET signes dysfonction cérébrale (altération conscience, convulsions,..) ET pas d'autres cause, à l'exception du VIH (définition OMS) | Quinine 20 mg/kg en dose de charge puis 10 mg/kg 3x par jour IV lente G5% en 4h. (adulte 500 mg 3x/jour) jusque prise orale possible ; relais PO (même doses) pour compléter 7 jours de traitement en tout<br><br>NB : si suspicion insuffisance hépatique/rénale, réduire dose de moitié après 48 h.<br><br>NB : relais par Artesunate-Amiodaquine (AS-AQ) du PNLP si disponible ; pas de Fansidar ( !) : insuffisant | 40 USD (7 jours quinine dont 4 en IV et en comptant les perfusions)<br><br>Artesunate-Amiodaquine gratuit en principe<br><br>+ 5-7 USD pour forfait hospitalisation 2 semaines |
| Méningite bactérienne | C : Présence de leucocytes (neutro) > 5/µL LCR ET coloration de Gram positive LCR ; culture pos INRB (tardif)<br><br>P : Présence de leucocytes (neutro) > 5/µL LCR ET signes méningés | A TOUS les patients suspects, donner immédiatement (après PL et en attendant comptage leuco LCR et avant Gram) : Ceftriaxone 2 gr IV (50 mg/kg)<br><br>Si présence de leucocytes (neutro) > 5/µL LCR, poursuivre Ceftriaxone 2 x 2 gr IV (ou IM) pendant 10 jours (14 jours si évolution lente)<br><br>Considérer Déxamethasone 3 x 4 mg IV par jour pendant 3 jours (chez VIH | Environ 60 USD (en comptant 4 jours de perfusion et 5 cathéters en tout)<br><br>+ 5-7 USD pour forfait hospitalisation 2 semaines |

| | | | |
|---|---|---|---|
| | | –) si forte suspicion pneumocoques (à la coloration de Gram) | |
| Neurotuberculose | P : Présence de leucocytes (lympho) > 5/µL LCR ET signes neuro ET évolution subaiguë, non réponse traitement AB empirique, signes d'autres localisation TB, contage, etc..<br>C : coloration Ziehl + sur LCR | Schéma RHZE du PNLT<br>Donner toujours dexamethasone 3x 4 mg/jour IV ou Prednisolone 30 mg/jour PO pendant 2 semaines (et réduction progressive) | Gratuit<br>Déxaméthasone (10 USD, 4 jours) et Prednisolone (6 USD/2 semaines)<br>Hospitalisation gratuite |
| Neurosyphilis | P : signes neuro ou LCR anormal ET RPR (ou TDR-syphilis) + et pas d'autre cause | Pénicilline G 5000000 UI 5x/jour IV pendant 14 jours | 50 USD (flacon péni G 5000000 IU = 800 FC)<br>+ 5-7 USD pour forfait hospitalisation 2 semaines |
| Infection VIH | C : TDR Determine ET TDR UniGold ET TDR Double Check tous positifs | Schéma PLNS (première ligne : Duovir-N : zidovudine-lamivudine-névirapine)<br>Si TB concomitant ou intolérance névirapine : efavirenz | Gratuit<br>+ 5-7 USD pour forfait hospitalisation 2 semaines |
| Cryptococcose (SIDA) | C: VIH positif ET Encre de Chine + dans LCR OU présence de leucocytes (lympho) > 5/µL LCR ET Antigène Crypto LATEX + dans sang | Soit patient en état d'avoir un traitement oral : fluconazole 1200 mg PO pendant 3 jours puis 800 mg/jour pendant 8 semaines (puis prophylaxie secondaire 200 mg/jour)<br>Soit état grave et/ou incapacité d'ingestion : amphotéricine liposomale (AmBisome amp 50 mg) à administrer à raison de 3 mg/kg/jour dans du G5% en IV lente (30 minutes minimum) ; FAIRE d'abord test avec 1 mg IV pour voir si allergie ! pas de monitoring labo particulier par la suite! ; puis relais avec fluconazole 800 mg/jour per os dès que possible, comme indiqué ci-dessus)<br>Ponctions lombaires itératives (jusque normalisation neurologique et/ou pression d'ouverture < 20 cm d'eau) | 30-50 USD selon gravité (perfusion-cathéter)<br>Fluconazole IV indisponible dans le pays ; amphotéricine liposomale recommandée par le programme pour formes graves (obtenue via MSF) |
| Toxoplasmose cérébrale (SIDA) | P: VIH positif ET lésions neuro focale | Cotrimoxazole 960 mg (= 2 tab simples) 3x par jour PO 4 semaines (10/50 mg/kg/jour)<br>Si état grave : Cotrimoxazole IV aux mêmes doses<br>Considérer Déxamethasone si grave<br>Si allergie (non menaçante) rechallenge | 5 USD (700 FC pour 28 compr)<br>Forme IV obtenue via MSF |

Note : La toxoplasmose cérébrale a été ajoutée car le traitement est simple (mais elle ne sera pas investiguée comme telle dans le NIDIAG car aucune certitude n'est possible sans imagerie cérébrale)

Les autres médicaments pris en charge par l'étude concernent le traitement aigu des complications neurologiques, en particulier les crises convulsives et psychotiques et la prise en charge de la douleur

***Médicaments disponibles à Mosango et prix***

| | | | |
|---|---|---|---|
| **Anticonvulsifs** | | | |
| Diazepam | IV | 5 mg | 200 FC |
| Diazepam | PO | 10 mg | 100 FC |
| Phenobarbital | IV | 130 mg | 900 FC |
| Phenobarbital | PO | 100 mg | 500 FC/10 co |
| **Vitamines neurotropes** | | | |
| Vitamine B1 | IV | 50 mg | 200 FC |
| Vitamine B1 | PO | 50 mg | 100 FC/10 co |
| Vitamine B6 | PO | 50 mg | 100FC /10 co |
| **Antipsychotique** | | | |
| Chlorpromazine | IV | 50 mg | 200 FC |
| **Antidouleurs** | | | |
| Paracétamol | PO | 500 mg | 100 FC/10 co |
| Aspirine | PO | 500 mg | 100 FC/10 co |
| Tramadol | IV | 100 mg | 700 FC |
| Tramadol | PO | 50 mg | 700 FC |
| Diclofenac | PO | 50 mg | 200 FC/10 co |
| Morphine | IV | 10 mg | 500 FC |

# 4 Définitions & abréviations

- CRF: "Case Report Form"
- THA: trypanosomiase humaine africaine
- LCR: liquide cépaholo-rachidien
- RHZE: rifampicine-isoniazide-pyrazinamide-éthambutol
- PNLTHA: Programme National de Lutte contre la THA
- PNLM: Programme National de Lutte contre le Paludisme
- PNLT: Programme National de Lutte contre la Tuberculose
- PNLS: Programme National de Lutte contre le SIDA
- FC : Francs Congolais
- IV : Intraveineux
- IM : Intramusculaire
- PO : Per os
- TDR : Test Rapide Diagnostique
- NECT : « Nifurtimox-Eflornithine Combination Therapy »

## 5 Registres et archives

| **Appendices & Formulaires à remplir** | |
|---|---|
| **Numéro** | **Titre** |
| 1 | Fiche de Suivi Clinique |
| 2 | Fiche de Médication |

## 6 Historique du document

*Indiquez les versions précédentes du SOP et les modifications apportées*

| **Révision** | |
|---|---|
| *18 février 2013* | *La version 1 approuvée le 22 juin 2012 a été modifiée sur les 3 points suivants :*<br>1. *Traitement de la cryptococcose méningée* ***<u>grave</u>*** *: le fluconazole IV n'étant pas/plus disponible en RDC, un stock d'amphotéricine liposomale a été rendu disponible (via MSF) à Mosango car il constitue, malgré son coût, le traitement de premier choix vu son efficacité et sa faible toxicité, sans nécessité de monitoring laboratoire. Le traitement proposé correspond aux guidelines OMS et MSF en vigueur (attention il ne s'agit pas d'amphotéricine « classique », donc dosage différent).*<br>2. *Pour éviter des ponctions lombaires trop fréquentes, l'investigateur peut dorénavant évaluer le patient cliniquement (disparition des symptômes neurologiques) pour décider d'arrêter les ponctions de « décharge » dans la cryptococcose méningée.*<br>3. *Etant donné la mise en place en février 2013 de l'étude fexinidazole (traitement oral de la THA) par le DNDI à proximité de Mosango (Masi-Manimba 30-45 minutes en voiture), et la volonté des équipes de recherche ITM et DNDI de favoriser les synergies, il est possible de référer pour ce traitement les patients éligibles diagnostiqués à Mosango (voir point NB3 : traitement THA), selon une procédure établie dans une convention* |

| **Nom et fonction** | **Date** | **Signature** |
|---|---|---|
| *Auteurs* | | |
| *Emmanuel Bottieau (coordinateur de l'étude) and Andrea Winkler (consultante neurologue)* | 19 Février 2013 | 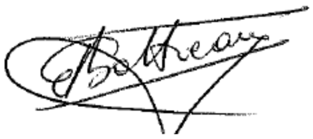 |
| *Revu par* | | |
| *Pascal Lutumba* | 19 Février 2013 | 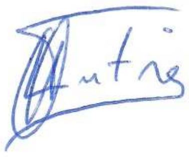 |
| *Approuvé par* | | |
| *Emilie Alirol* | 21 Février 2013 | 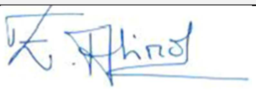 |

| | |
|---|---|
| | **SOP titre:** Mini anion exchange centrifugation technique (mAECT) |
| | **Projet/étude:** Evaluation de tests rapides en association avec des prédicteurs cliniques et de laboratoire pour le diagnostic de maladies tropicales négligées dans des patients se présentant avec des troubles neurologiques dans les hôpitaux ruraux au Bandundu, République Démocratique du Congo. |

# 1. Domaine et application

La technique de "mini-anion exchange centrifugation (mAECT)" est une méthode qui permet de séparer et de concentrer les trypanosomes du sang de patients infectés..
Les trypanosomes sont isolés à partir du sang veineux par chromatographie échangeuse d'anions. Ils sont concentrés au fond d'un tube collecteur par centrifugation à faible vitesse. Après centrifugation, la pointe du tube collecteur est examinée au microscope révélant la présence de trypanosomes mobiles. Le volume de sang utilisé (500 µl) permet la détection du parasite à une concentration de moins de 30 trypanosomes/ml et donc d'atteindre une sensibilité diagnostique élevée.
Cette SOP est basée sur la notice qui est livrée avec les kits mAECT. Les kits mAECT sont disponibles à l'Institut National de Recherche Biomédicale à Kinshasa, R.D. Congo.

# 2. Responsabilités

| Fonction | Activités |
|---|---|
| Technicien de labo / infirmier | Prise de sang **sur héparine (Li ou K)** |
| Technicien de labo | Exécution du test mAECT |

# 3. Procédures

## 3.1 Conservation de la mAECT

- De préférence, conserver les mini-colonnes mAECT à 4°C. **Ne pas congeler**.

## 3.2 Précautions

- Ne pas ouvrir les tubes avant de les utiliser (ils sont stériles).
- Les trypanosomes sont fragiles le sang doit donc être examiné dès que possible, au plus tard une demi-heure après le prélèvement.
- Les trypanosomes sont infectieux, utilisez donc des gants en latex lors de la manipulation des échantillons sanguins

## 3.3 Matériel et échantillons

### 3.3.1 Matériel fournis dans les kits

- Brochure explicative
- Tube collecteur 4 ml en plastique avec bouchon bleu (1)
- Mini-colonne mAECT en plastique avec bouchon supérieur en caoutchouc et bouchon inférieur blanc (2)
- Pipette graduée en plastique de 3 ml (pipette de transfert) (3)
- Tube de centrifugation de 14 ml (4)

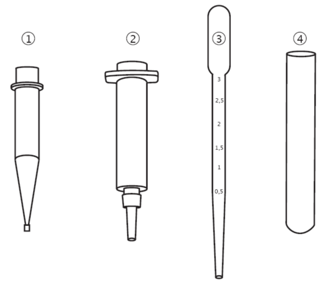

### 3.3.2 Matériels supplémentaires requis

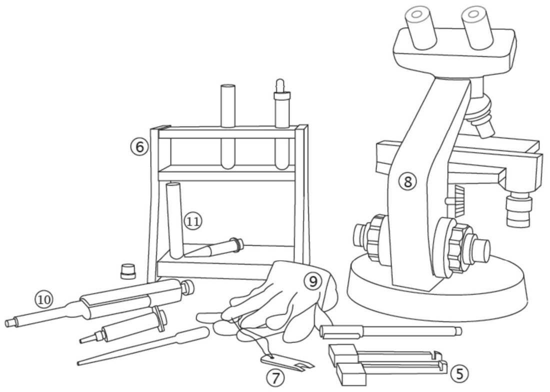


- Chambre de lecture mAECT (5)
- Portoir à 2 étages pour mini-colonnes mAECT (6)
- Clé pour ouvrir la colonne mAECT (7)
- Microscope avec oculaires 10x ou 15x et objectif 10x (8)
- Gants de protection en latex (9)
- Micropipette 500 µl avec pointes bleues à usage unique (10)
- Papier absorbant
- Container à déchet
- Liquide désinfectant
- Centrifugeuse

### 3.3.3 Echantillon de sang à examiner

- Du sang fraîchement prélevé sur héparine. Ne pas utiliser l'EDTA ou citrate comme anticoagulant!

## 3.4 Mode opératoire

### 3.4.1 Préparation de la colonne

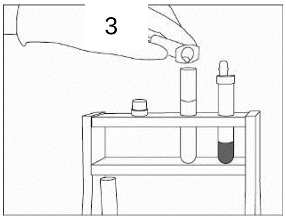


1. Placer un tube de centrifugation de 14 ml sur l'étage du haut du portoir et un autre sur l'étage du bas.
2. Ôter le bouchon supérieur en caoutchouc de la mini-colonne.
3. Verser le tampon surnageant de la colonne dans le tube de 14 ml placés sur l'étage du haut.
4. Inscrire le numéro de patient sur la mini-colonne et la placer sur le portoir

### 3.4.2 Dépôt de l'échantillon de sang

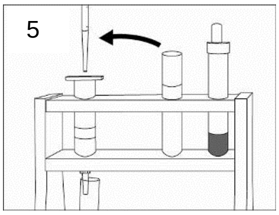


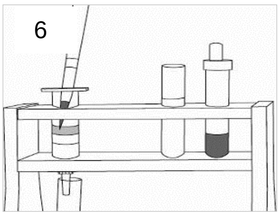


5. À l'aide de la pipette de transfert, déposer 500 µl de tampon sur la colonne.
6. À l'aide d'une micropipette, mélanger soigneusement 500 µl de sang hépariné avec le tampon dans la colonne.

### 3.4.3 Élution

7. Ouvrir un tube collecteur et mettez le bouchon bleu de côté. Noter le numéro de patient de l'échantillon sur la surface prévue à cet effet sur le tube collecteur.
8. Insérer le tube collecteur dans le tube de centrifugation de 14 ml se trouvant sur l'étage du bas du portoir.
9. Enlever le bouchon blanc inférieur de la colonne en plaçant la clé entre la base de la colonne et le haut du bouchon. La colonne commence à couler.
10. Immédiatement, remettre la colonne sur le portoir en haut du tube collecteur. S'assurer que la pointe de la colonne pénètre dans le tube collecteur.

11. Laisser la colonne écouler à sec dans le tube collecteur
12. Verser tout le reste du tampon dans la colonne et laisser écouler à sec dans le tube collecteur.
13. Déposez la colonne mAECT dans un conteneur à déchets contaminés

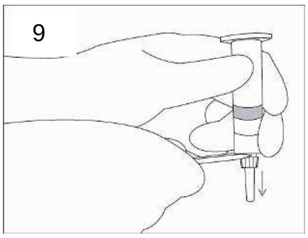


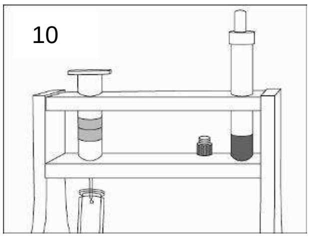


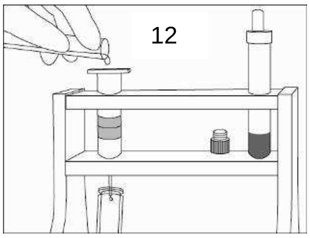


### 3.4.4 Centrifugation du tube collecteur

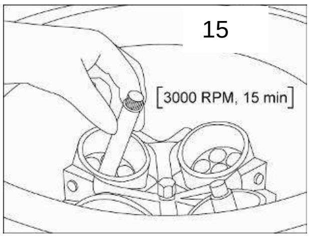


14. Remettre le bouchon bleu sur le tube collecteur
15. Ré-insérer le tube collecteur dans le tube de centrifugation de 14 ml (si on l'avait enlevé pour remettre le bouchon)
16. Centrifuger pendant 10-15 min à 3000 rpm (Centrifugeuse AML A8 en position 8).

### 3.4.5 Examen microscopique du sédiment

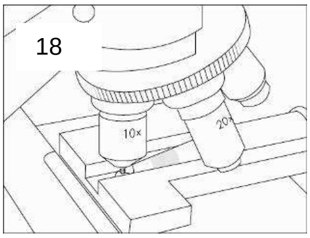


17. Monter la chambre de lecture sous l'objectif 10x du microscope.
18. Glisser le tube collecteur dans la chambre de lecture avec son rebord dans la rainure transversale et sa pointe au-dessus de l'ouverture, une face large de la pointe dirigée vers le haut.
19. Ajuster le microscope pour avoir un contraste maximale (descendre le condensateur, fermer le diaphragme, diminuer la lumière).
20. Les trypanosomes sont visibles comme des petits organismes s'agitant dans le fond du tube. Prendre soin de bien balayer sur toute la profondeur de la pointe du tube.
21. Parfois des particules de débris peuvent passer la colonne et peuvent rendre difficile la lecture.  Dans ce cas tourner le tube de 180°.

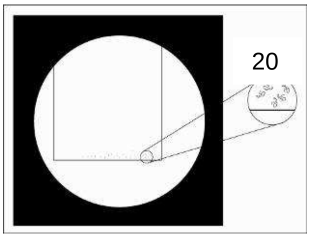


## 3.5 Interprétation et enregistrement du résultat

- Si un trypanosome est détecté: confirmer sa présence par une deuxième personne.  Si confirmé, le résultat est positif.  Si non-confirmé : cherchez un autre trypanosome et confirmer avec un deuxième personne.
- Si aucun trypanosome n'a été détecté après examen de toute la pointe, le résultat est négatif.
- Noter le résultat (positif, négatif, non-fait) dans le cahier de laboratoire en face / sous le numéro de patient

## 3.6 Déchets, nettoyage

- Déposez la colonne mAECT, le tube collecteur et les pointes utilisées dans un conteneur pour déchets contaminés.
- Nettoyez le portoir avec une solution de 0.2% de chlore.

## 4. Définitions & abréviations

- mAECT : Technique de mini anion exchange centrifugation
- rpm : Rotations par minute

## 5. Enregistrements et archives

| Appendices & formulaires à compléter | |
|---|---|
| **Numéro** | **Titre** |
| 1 | Cahier de laboratoire |

## 6. Références


- Lumsden et al. (1979) Trans. R. Soc. Trop. Med. Hyg. 73: 312-317


## 7. Historique du document

| Révision | |
|---|---|
| SOP-WP2-LAB-01-V1-30Mar2012 | Version initiale |
| SOP-WP2-LAB-01-V02-31Jul2012 | Volume de sang changé de 350µl à 500µl et seuil de détection à 30 trypanosomes/µl |
| SOP-WP2-LAB-01-V03-18Sep2012 | 3.4.4, 16 Vitesse centrifugeuse AML A8 ajouté |

| Nom et fonction | Date | Signature |
|---|---|---|
| Auteur | | |
| Philippe Büscher | 16/Jul/2012 |  |
| Révision par | | |
| | | |
| Approuvé par | | |
| Veerle Lejon | 31/Jul/2012 | 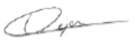 |

| | |
|---|---|
| nidiag Better Diagnosis of Neglected Infectious Diseases | **SOP titre:** Simple Centrifugation Modifiée (MSC) |
| | **Projet/étude:** Evaluation de tests rapides en association avec des prédicteurs cliniques et de laboratoire pour le diagnostic de maladies tropicales négligées dans des patients se présentant avec des troubles neurologiques dans les hôpitaux ruraux au Bandundu, République Démocratique du Congo. |

# 1. Domaine et application

Dans la méthode MSC ("Modified Simple Centrifugation") les trypanosomes du liquide céphalorachidien (LCR) sont concentrés dans un tube collecteur, par centrifugation à faible vitesse (3,000 – 3,500 rpm). Après centrifugation, la pointe du tube collecteur est examinée au microscope révélant la présence de trypanosomes mobiles.

# 2. Responsabilités

| Fonction | Activités |
|---|---|
| Technicien de labo / infirmier ou investigateur | ▪ Ponction lombaire |
| Technicien de labo | ▪ Exécution du test MSC<br>▪ Validation du test MSC |

# 3. Procédures

## 3.1 Précautions

- Examinez le LCR dès que possible, au plus tard 15 minutes après le prélèvement (les trypanosomes sont fragiles).
- utilisez des gants lors de manipulations du LCR (les trypanosomes sont infectieux).

## 3.2 Matériel et échantillons

### 3.2.1 Matériel fournis dans le kit MSC

- Brochure explicative
- Tube collecteur en plastique avec bouchon
- Pipette graduée en plastique (pipette de transfert)
- Tube de centrifugation de 14 ml

### 3.2.2 Matériels supplémentaires requis

- Chambre de lecture pour mini-colonnes mAECT
- Microscope avec oculaires 10x ou 15x et objectif 10x
- Gants de protection
- Container à déchets contaminés
- Centrifugeuse

### 3.2.3 Echantillon à examiner

- 4 ml de LCR fraîchement prélevé

## 3.3 Mode opératoire

- Ouvriez le tube collecteur.
- Y transférez 4 ml de LCR à l'aide de la pipette de transfert.
- Refermez le tube collecteur avec son bouchon
- Insérez le tube collecteur dans un tube de centrifugation (figure 1)
- Centrifugez pendant 10 minutes à 3,500 rpm ou pendant 15 minutes à 3,000 rpm.
- Montez la chambre de lecture sous l'objectif 10x du microscope (figure 2).
- Après centrifugation, glissez le tube collecteur dans la chambre de lecture : Le rebord du tube doit être dans la rainure transversale et sa pointe au-dessus de l'ouverture. Une face large de la pointe doit être dirigée vers le haut.
- Ajustez le microscope pour avoir un contraste maximale (descendez le condensateur, fermez le diaphragme, diminuer la lumière).

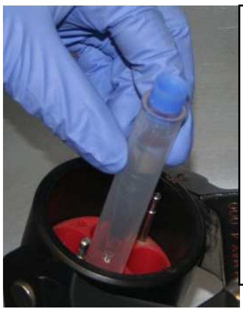

Figure 1 : Le tube collecteur est inséré dans un tube de centrifugation pour protéger la pointe pendant la centrifugation

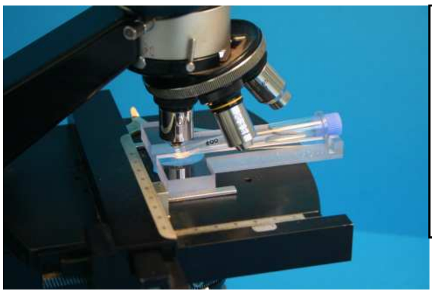

Figure 2 : Montage de la chambre de lecture et tube collecteur sous le microscope

- Les trypanosomes sont visibles comme des petits organismes s'agitant dans le fond du tube parmi les globules blancs. Prenez soin de bien balayer sur toute la profondeur de la pointe du tube afin de ne pas rater les trypanosomes.
- S'il y a de débris qui rendent difficile la lecture, tournez le tube 180°.

## 3.4 Interprétation du résultat

- Si un trypanosome est détecté: confirmer sa présence par une deuxième personne. Si confirmé, le résultat est positif. Si non-confirmé : cherchez un autre trypanosome et confirmer avec une deuxième personne.
- Si aucun trypanosome n'a été détecté après avoir examiné toute la pointe, le résultat est négatif.
- Noter le résultat (positif, négatif, non-fait) dans le cahier de laboratoire en face / sous le numéro de patient

## 3.5 Déchets, nettoyage

Après lecture microscopique :

- Déposez le tube collecteur et la pipette de transfert dans un conteneur à déchets contaminés.
- Nettoyez la chambre de lecture et la tube de centrifugation avec une solution de 0.2% de chlore.

# 4. Définitions & abréviations

- LCR : Liquide CéphaloRachidien
- MSC : Simple centrifugation modifiée
- mAECT : Technique de mini anion exchange centrifugation
- rpm : Rotations par minute

## 5. Registres et archives

| Appendices & formulaires à compléter | |
|---|---|
| **Numéro** | **Titre** |
| 1 | Cahier de laboratoire |

## 6. Références


- Miézan T.W., Meda H.A., Doua F., Djè N.N., Lejon V., Büscher P. (2000) Single centrifugation of cerebrospinal fluid in a sealed pasteur pipette for simple, rapid and sensitive detection of trypanosomes. Trans. R. Soc. Trop. Med. Hyg. 94, 293.
- Büscher P, Mumba Ngoyi D, Kaboré J, Lejon V, Robays J, Jamonneau V, Bebronne N, Van der Veken W, Biéler S. (2009) Improved Models of Mini Anion Exchange Centrifugation Technique (mAECT) and Modified Single Centrifugation (MSC) for sleeping sickness diagnosis and staging. PLoS Negl Trop Dis. 3(11):e471.


## 7. Historique du document

| Révision | |
|---|---|
| | Version initiale |

| Nom et fonction | Date | Signature |
|---|---|---|
| Auteur | | |
| Veerle Lejon | 13/Feb/2012 | |
| Révision par | | |
| Philippe Büscher | 30/Mar/2012 | |
| Approuvé par | | |
| Emilie Alirol | 27 Feb 2012 | |

| | |
|---|---|
| | **SOP titre:** Test d'agglutination sur carte *T.b. gambiense* (CATT/*T.b. gambiense*) |
| | **Projet/étude:** Evaluation de tests rapides en association avec des prédicteurs cliniques et de laboratoire pour le diagnostic de maladies tropicales négligées dans des patients se présentant avec des troubles neurologiques dans les hôpitaux ruraux au Bandundu, République Démocratique du Congo. |

# 1. Domaine et application

L'infection par *Trypanosoma brucei gambiense* (*T.b. gambiense*) donne lieu à la production d'anticorps circulants dirigés contre plusieurs antigènes de surface du parasite. Ces anticorps dans le sang, le plasma ou le sérum de l'hôte infecté peuvent être détectés par le test d'agglutination sur carte *T.b. gambiense* (CATT).
L'antigène CATT est une suspension lyophilisée de trypomastigotes sanguicoles purifiés, fixés et colorés exprimant un sérotype prédominant de *T.b. gambiense*. Après le test, la présence d'anticorps est visible sous forme d'agglutinats. Le test CATT existe sous 2 formats différents : 1° CATT-R250 qui contient 50 tests par flacon ; 2° CATT-D10 qui contient 10 tests par flacon.

# 2. Responsabilités

| Fonction | Activités |
|---|---|
| Technicien de labo / infirmier / investigateur | ▪ Prend de sang |
| Technicien de labo | ▪ Exécute le test CATT<br>▪ Valide le test CATT<br>▪ Enregistre les résultats |

# 3. Procédures

## 3.1 Précautions

- Les trypanosomes sont infectieux, utilisez donc des gants lors des manipulations du sang, sérum ou plasma.

## 3.2 Matériel et échantillons

### 3.2.1 Matériel fournis dans les kits (CATT-R250 ou CATT-D10) et conservation

**CATT-R250**

- Antigène CATT (**2,5** ml / flacon) : Suspension lyophilisée de trypanosomes purifiés, fixés et colorés (bleu). Conservation: au frigo (+2°C / +8°C) ou au congélateur (-20°C). Après reconstitution, l'antigène peut être utilisé pendant 1 semaine à condition de le conserver au frigo (+2°C à +8°C), ou pendant 8 heures à +37°C.
- Tampon CATT (**30** ml / flacon) : Solution tampon salin (phosphate pH 7.2) utilisée pour la reconstitution de l'antigène CATT, des contrôles et pour la préparation des dilutions des échantillons. Conservation: au frigo (+2°C / +8°C). NE PAS CONGELER !

**CATT-D10**

- Antigène CATT (**0,75** ml / flacon) : Suspension lyophilisée de trypanosomes purifiés, fixés et colorés (bleu). Conservation: Température ambiante. Si possible, au frigo (+2°C / +8°C) ou au congélateur (-20°C). Après reconstitution, l'antigène peut être utilisé pendant 1 semaine à condition de le conserver au frigo (+2°C à +8°C), ou pendant 8 heures à +37°C.

- Tampon CATT (**2,5** ml / flacon) : Solution tampon salin (phosphate pH 7.2) utilisée pour la reconstitution de l'antigène CATT, des contrôles et pour la préparation des dilutions des échantillons. Conservation: Température ambiante. Si possible, au frigo (+2°C / +8°C). NE PAS CONGELER !

- Contrôle positif (0,5 ml / flaco$^{2}$n) : Antisérum de chèvre dilué et lyophilisé. Conservation: au frigo (+2°C / +8°C) ou au congélateur (-20°C).
- Contrôle négatif (0,5 ml / flacon) : Solution lyophilisée d'albumine bovine. Conservation: au frigo (+2°C / +8°C) ou au congélateur (-20°C).
- Tubes capillaires héparinés
- Cartes plastifiées
- Tiges
- Mini-poire
- 1 seringue (CATT-R250 : 2,5 ml ; CATT-D10 : 1 ml)
- Compte-gouttes

### 3.2.2 Matériel supplémentaire requis

- Gants
- Agitateur électrique
- Carte « Scores de référence » (sur demande)
- Papier absorbant

Pour le test sur sang total :

- Prise de sang capillaire : coton & désinfectant, lancettes, portoir pour micro capillaires
- Prise de sang veineux : Pipette 25 µl, embouts jaunes

Pour le test sur dilutions de plasma :

- Micropipette P100 ou P200 avec embouts jaunes
- Microplaque de 96 puits (8 x 12)

### 3.2.3 Echantillon à examiner

- Sang sur anticoagulant (sang capillaire ou sang veineux)
- Titration : sérum ou plasma

## 3.3 Mode opératoire CATT

### 3.3.1 Placement et vitesse de l'agitateur

- Avant de commencer le test, assurez-vous que l'agitateur est placé sur une surface horizontale, ensuite ajustez la vitesse de l'agitateur à 60 rotations par minute. A cause de différentes intensités du courant, la vitesse peut s'altérer. Vérifiez périodiquement la vitesse de l'agitateur.

### 3.3.2 Reconstitution de l'antigène

- **CATT-R250 :** A l'aide de la seringue, ajouter **2,5 ml** de tampon CATT au flacon d'antigène lyophilisé.
- **CATT-D10 :** A l'aide de la seringue, ajouter **0,75 ml** de tampon CATT au flacon d'antigène lyophilisé.
- Mélangez manuellement pendant quelques secondes afin d'obtenir une suspension bien homogène (couleur bleu). Evitez les bulles d'air.
- Placez un compte-gouttes sur le flacon.
- L'antigène est prêt à l'emploi.

### 3.3.3 Contrôle de qualité

Effectuer un test de qualité chaque semaine:

- Identifiez les cercles et notez la date sur la carte.

- A l'aide de la seringue, dissolvez le contrôle négatif avec 0,5 ml de Tampon CATT.
- A l'aide de la seringue, dissolvez le contrôle positif avec 0,5 ml de Tampon CATT.
- Placez un compte-gouttes sur chacun des flacons contrôle.
- Prenez une carte plastifiée neuve.
- Déposez 1 goutte de l'antigène CATT (couleur bleu) dans le premier cercle, et 1 goutte dans le deuxième cercle de la carte, en tenant le flacon verticalement. Laisser tomber la goutte librement.
- Déposez 1 goutte du contrôle négatif dans le premier cercle de la carte.
- Dans le deuxième cercle de la carte, déposez 1 goutte du contrôle positif.
- Mélangez la goutte de réactif et la goutte de contrôle à l'aide de la tige, en l'étalant dans toute la zone de réaction du cercle; prenez soin de bien essuyer la tige entre 2 mélanges avec du papier absorbant pour éviter les contaminations.
- Mettez la carte sur l'agitateur et laissez-la tourner pendant 5 minutes.
- Après les 5 minutes, lisez les réactions, en ouvrant le couvercle de l'agitateur mais sans déplacer l'agitateur ni la carte.
- Le contrôle positif, qui contient des anticorps, doit donner des agglutinations.
- Le contrôle négatif, qui ne contient pas d'anticorps, n'a pas d'agglutinations.

### 3.3.4 CATT sur sang total

#### 3.3.4.1 Prise de sang capillaire par piqûre du doigt

- Piquez avec une micro-lancette le doigt préalablement désinfecté.
- Essuyez la première goutte de sang.
- Remplissez un tube capillaire hépariné sur environ ¾ de sa longueur en évitant les bulles d'air.
- Inclinez le tube capillaire à plusieurs reprises dans les 2 sens afin de bien mélanger le sang à l'héparine.
- Déposez le tube capillaire horizontalement dans un portoir.

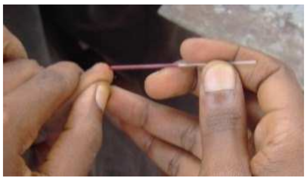

Prise de sang en tube capillaire

#### 3.3.4.2 Prise de sang intraveineux

- Du sang veineux peut également être utilisé à la place du sang capillaire. Pour la prise de sang, voir procédure approprié.

#### 3.3.4.3 Réaction d'agglutination

- Notez le numéro de patient et la date sur la carte
- Déposez 1 goutte d'antigène CATT (couleur bleu) bien homogénéisé dans chaque cercle de la carte selon le nombre de tests à effectuer.
- Afin d'avoir des gouttes de volume constant, tenez le compte-gouttes verticalement et laissez tomber la goutte librement sans toucher la carte.
- Ajoutez ensuite 1 goutte de sang du capillaire avec la micro-poire (Figure 1). Afin d'avoir des gouttes de volume constant tenez le capillaire verticalement et laissez tomber la goutte librement sans toucher la carte. A la place du sang capillaire, 25 µl de sang veineux peuvent être utilisés.
- Mélangez l'antigène CATT et le sang avec la tige en étalant le mélange à l'intérieur du cercle jusqu'à un millimètre avant le bord du cercle (figure 2).
- Prenez soin de bien essuyer la tige de mélange entre deux mélanges avec du papier absorbant pour éviter la contamination entre les échantillons.
- Mettez la carte sur l'agitateur et laissez-la tourner pendant 5 minutes.
- Afin d'éviter le séchage, fermez toujours le couvercle de l'agitateur et mettez un tampon d'ouate humide sous le couvercle.

- Après les 5 minutes quand l'agitateur s'arrête : lisez les réactions, en ouvrant le couvercle de l'agitateur mais sans déplacer l'agitateur ni la carte (figure 3).

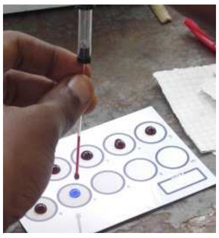

Figure 1 : Dépôt d'une goutte de sang du capillaire avec la micro-poire

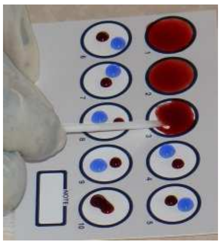

Figure 2 : Mélange de l'antigène et sang

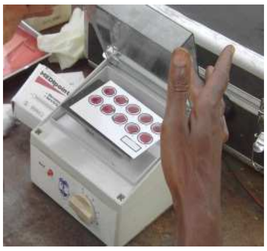

Figure 3 : Lecture du résultat

### 3.3.4.3 Lecture de la réaction d'agglutination et interprétation

- Lisez les résultats de la façon suivante (voir aussi la carte « Scores de référence »):

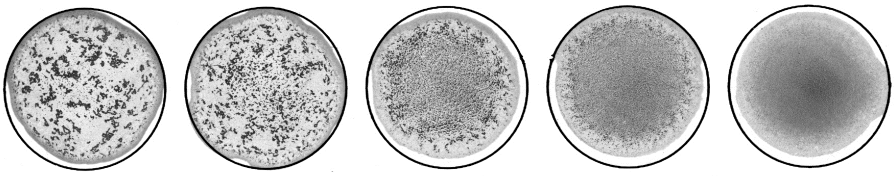

| +++ | ++ | + | +/- | - |
|---|---|---|---|---|

+++ = **Fortement positif** (Agglutination très forte)
++ = **Positif** (Agglutination forte)
+ = **Positif** (Agglutination modérée)
± = **Faiblement positif** (Agglutination faible)
– = **Négatif** (Absence d'agglutination)

## 3.3.5 CATT sur sérum ou plasma dilué

### 3.3.5.1 Préparation des dilutions de plasma

- Prenez une microplaque de 96 puits (8 x 12)
- Montez une pointe jaune (200 µl) sur une micropipette P200
- Vérifiez que la pointe est bien enfoncée sur la micropipette.
- Prélevez 50 µl de Tampon CATT
- Déposez les 50 µl prélevés dans le puits H1 de la microplaque (1)
- Sans changer d'embout procédez de même pour les cinq puits suivants (G1 à C1)
- Au total 6 puits dans une rangée contiendront chacun 50 µl de tampon

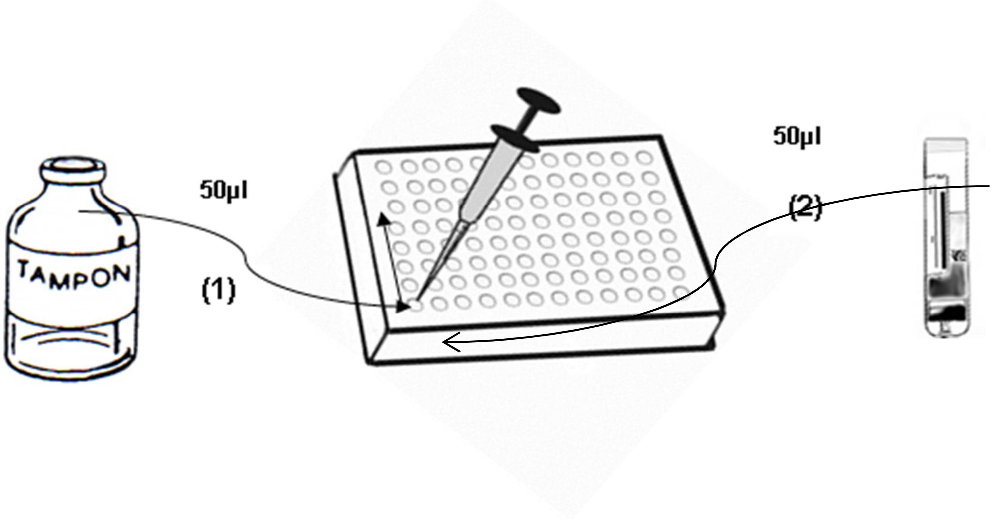


- Changez la pointe jaune de la micropipette
- Prélevez 50 µl de plasma ou sérum dans le tube de prélèvement (2)
- Déposez ces 50 µl de plasma dans le puits H1

- Mélangez en prélevant et en redéposant avec la pipette plusieurs fois (5 à 6 fois)
- Sans changer de pointe, prélevez 50 µl du mélange dans le puits H1 et déposez-les dans le puits G1.
- Mélangez en prélevant et en redéposant le liquide plusieurs fois à l'aide de la micro pipette. Une fois bien mélangé, prélevez 50 µl du mélange du puits G1 et déposez-les dans le puits F1. Mélangez à l'aide de la micro pipette.
- Procédez de la sorte jusqu'au puits C1 (3).
- Ce dernier puits contiendra plus de liquide que les autres.
- Jetez l'embout jaune.
- Les 6 puits contiennent chacun les dilutions suivantes :
    - Le premier puits H1 contient une dilution du plasma au demi (1/2)
    - Le 2ème puits G1 contient une dilution du plasma au quart (1/4)
    - Le 3ème puits F1 contient une dilution du plasma au huitième (1/8)
    - Le 4ème puits E1 contient une dilution du plasma au seizième (1/16)
    - Le 5ème puits D1 contient une dilution du plasma au trente-deuxième (1/32)
    - Le 6ème puits C1 contient une dilution du plasma au soixante quatrième (1/64)
- Les dilutions sont prêtes pour réaliser le test CATT sur dilution du plasma.
- Placez le couvercle sur la microplaque pour éviter toute évaporation.

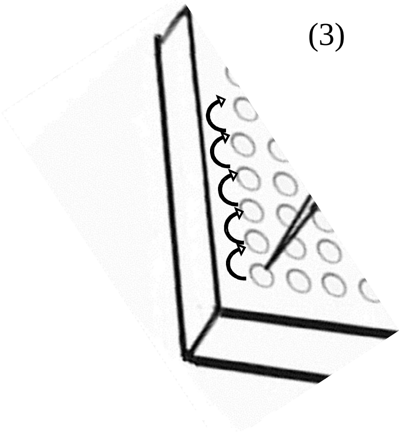

(3)

### 3.3.5.2 CATT sur plasma dilué (figure 4)

- Sur une carte plastifiée neuve déposez une goutte d'antigène CATT (couleur bleu) sur chacun des 5 cercles d'une ligne (4)
- Montez une pointe jaune sur une micropipette de **25 µl.** A l'aide de la micropipette
    - mélangez la dilution **1/64** (puits C1)) en prélevant et redéposant le liquide plusieurs fois à l'aide de la micropipette. Prélevez 25 µl de la dilution **1/64** et déposez-les sur le **cinquième cercle** de la carte (dernier cercle à droite), sans toucher le réactif CATT.
    - Sans changer d'embout, et en mélangeant chaque fois la dilution
    - prélevez **25 µl** de la dilution **1/**32 (puits D1) et déposez-les sur le **quatrième cercle**, puis
    - prélevez **25 µl** de dilution **1/16** (puits E1) et déposez sur le **3ème cercle**, puis
    - prélevez **25 µl** de dilution **1/8** (puits F1) et déposez sur le **2ème cercle**, puis
    - prélevez **25 µl** de dilution **1/4** (puits G1) et déposez sur le **1er cercle**.
- Jetez la pointe jaune

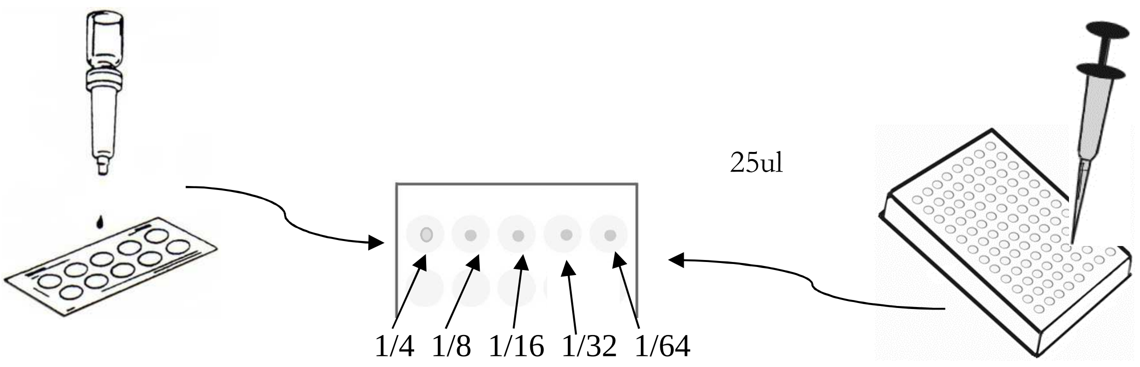


(4) (5)

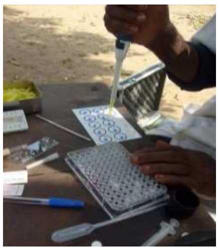

Figure 4 : Dépôt des dilutions sur la carte

- Mélangez l'antigène CATT et le plasma dilué avec la tige plastique en étalant le mélange à l'intérieur du cercle jusqu'à un millimètre des limites. Prenez soin de bien essuyer la tige de

mélange entre deux mélanges avec du papier absorbant pour éviter la contamination entre les différentes dilutions.

- Mettez la carte sur l'agitateur et laissez-la tourner pendant 5 minutes.
- Afin d'éviter le séchage, toujours fermer le couvercle de l'agitateur et mettez un tampon d'ouate humide sous le couvercle.

### 3.4 Lecture de la réaction d'agglutination, interprétation et enregistrement des résultats

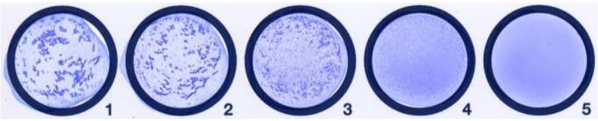


| +++ | +++ | ++ | - | - |
|---|---|---|---|---|
| 1/4 | 1/8 | 1/16 | 1/32 | 1/64 |

Dans cet exemple, le plasma est positif aux dilutions 1/4, 1/8 et 1/16, négatif aux dilutions 1/32 et négatif au 1/64. Son titre est donc de 1/16.

- Après les 5 minutes, quand l'agitateur s'arrête, lire les réactions, en ouvrant le couvercle de l'agitateur mais sans déplacer l'agitateur ni la carte.
- Le titre du plasma correspond à la dernière dilution qui est positive.
- La lecture et le titre doivent être confirmés par une deuxième personne
- Noter le résultat (positif, négatif, si positif notez le titre auquel le plasma est positif) dans le CRF de laboratoire sous le numéro de patient

### 3.4.2 Déchets, nettoyage

- Eliminez les cartes CATT, les pointes et les microplaques utilisées dans un conteneur pour déchets contaminés.
- Eliminez les lancettes et capillaires dans un conteneur pour aiguilles.
- Nettoyez les tiges et le portoir avec une solution de 0.2% de chlore. Les micro-poires et l'agitateur peuvent être nettoyés avec 0.2% de chlore s'ils sont contaminés.

## 4. Définitions & abréviations

CATT: Test d'agglutination sur carte
*T.b. gambiense*: *Trypanosoma brucei gambiense*
rpm: Rotations par minute

## 5. Registres et archives

| **Appendices & formulaires à compléter** | |
|---|---|
| **Numéro** | **Titre** |
| 1 | Cahier de laboratoire |

## 6. Références

Magnus E, Vervoort T, and Van Meirvenne N (1978). A card-agglutination test with stained trypanosomes (C.A.T.T.) for the serological diagnosis of *T.b. gambiense* trypanosomiasis. Ann Soc Belg Méd Trop 58: 169-176.

## 7. Historique du document

| **Révision** | |
|---|---|
| SOP-WP2-LAB-03-V01-30Mar2012 | Version initiale |
| SOP-WP2-LAB-03-V02-18Sep2012 | 3.3.3 Fréquence contrôle changé et identification des cercles ajouté. |

| | |
|---|---|
| | 3.3.4.3 Identification des cercles ajouté |
| SOP-WP2-LAB-03-V03-24Sep2012 | CATT-D10 ajouté dans l'introduction et paragraphes 3.2.1 et 3.3.2 |
| SOP-WP2-LAB-03-V04-27Dec2012 | Addition de la couleur de la suspension antigène CATT (bleu). |

| **Nom et fonction** | **Date** | **Signature** |
|---|---|---|
| Auteur | | |
| Barbara Barbé | 27/12/2012 | 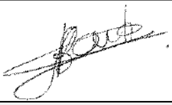 |
| Révision par | | |
| Philippe Gillet | 28/12/2012 | 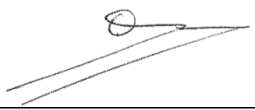 |
| Approuvé by | | |
| Emilie Alirol | 02/01/2013 | 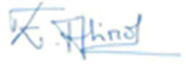 |

| | |
|---|---|
| | **SOP titre:** La technique de centrifugation en tube capillaire (CTC) |
| | **Projet/étude:** Evaluation de tests rapides en association avec des prédicteurs cliniques et de laboratoire pour le diagnostic de maladies tropicales négligées dans des patients se présentant avec des troubles neurologiques dans les hôpitaux ruraux au Bandundu, République Démocratique du Congo. |

# 1. Domaine et application

La technique de centrifugation en tube capillaire (CTC) aussi appelée technique de centrifugation en tube capillaire micro-hématocrite (mHCT) ou test de Woo, est une méthode pour concentrer les trypanosomes présents dans le sang des patients infectés.
Les tubes capillaires contenant de l'anticoagulant sont remplis avec du sang. Par centrifugation à haute vitesse, les trypanosomes sont concentrés au niveau des globules blancs, entre le plasma et les érythrocytes.

# 2. Responsabilités

| **Fonction** | **Activités** |
|---|---|
| Technicien de labo / infirmier | ▪ Prise de sang sur anticoagulant |
| Technicien de labo | ▪ Exécution du test CTC, Validation du test CTC |

# 3. Procédures

## 3.1 Précautions

- Les trypanosomes sont fragiles le sang doit donc être examiné dès que possible, au plus tard une demi-heure après le prélèvement.
- Les trypanosomes sont infectieux, utilisez donc des gants lors des manipulations du sang.

## 3.2 Matériel et échantillons

### 3.2.1 Matériel

- Tubes capillaires
- Plasticine
- Eau (l'eau du robinet peut être utilisée si elle ne contient pas de particules visibles au microscope à magnification 100x)
- Chambre de lecture
- Lamelle couvre objet de 24 x 24 mm
- Centrifugeuse à hématocrite

### 3.2.2 Echantillon de sang à examiner

- Du sang pris du doigt (sang capillaire) ou du sang veineux fraîchement prélevé sur anticoagulant.

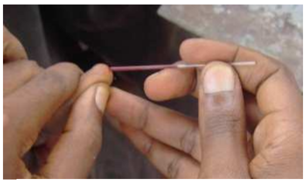

Etape 2 : Prise de sang en tube capillaire

## 3.3 Mode opératoire

1. Prendre un tube capillaire contenant un anticoagulant (héparine ou EDTA) neuf
2. Remplir le tube capillaire au trois quarts avec du sang frais (photo).
3. Répéter l'opération : il faut au moins 4 capillaires par personne.
4. Fermer le coté sec des capillaires avec de la plasticine (figure).
5. Placer les capillaires dans une centrifugeuse à hématocrite, avec le côté du

plasticine à l'extérieur (figure)

6. Centrifuger les capillaires à vitesse élevée (12.000 rpm) pendant 5 min pour séparer les globules rouges du plasma
7. Monter les capillaires entre lame et lamelle. De préférence, utiliser une chambre de lecture spéciale adaptée à cet usage.
8. Remplir l'espace vide entre la lame et la lamelle d'eau afin de réduire la diffraction (figure).
9. Examiner les 4 capillaires pour la présence de trypanosomes mobiles entre les globules blancs et le plasma (figure) à faible grossissement (10x10 ou 10x20).
10. Ajuster le microscope pour avoir un contraste maximale (descendre le condensateur, fermer le diaphragme, diminuer la lumière).

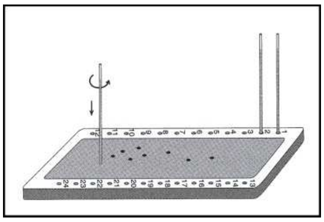

Etape 4: fermer le capillaire

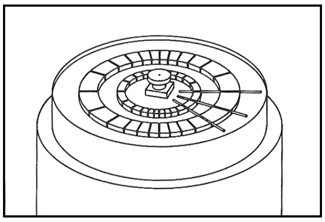

Etape 5: capillaires dans centrifugeuse

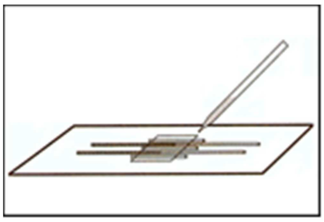

Etape 8: montage capillaire entre lame et lamelle

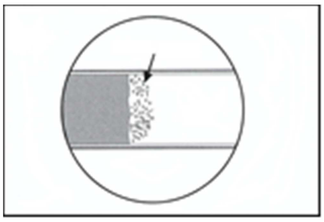

Etape 9: position des trypanosomes

- Interprétation du résultat :
  - o Si un trypanosome est détecté dans au moins 1 capillaire : demader à une deuxième personne de confirmer sa présence. Si confirmé, le résultat est positif. Si non-confirmé : cherchez un autre trypanosome.
  - o Si aucun trypanosome n'a été détecté après avoir examiné 4 capillaires, le résultat est négatif.

- Après lecture microscopique :
  - o Déposez les capillaires dans un conteneur à aiguilles.
  - o Nettoyez la chambre de lecture avec une solution de 0.2% de chlore.

## 4. Documentation des Résultats

Notez le résultat (positif, négatif, non-fait) du CTC dans le cahier de laboratoire.

## 5. Définitions & abréviations

CTC : Centrifugation sur tube capillaire
mHCT : Technique de centrifugation sur tube capillaire micro-hématocrite
rpm : Rotations par minute

## 6. Registres et archives

| **Appendices & formulaires à compléter** | |
|---|---|
| **Numéro** | **Titre** |
| 1 | Cahier de laboratoire |

## 7. Références


- Woo PTK (1970). The haematocrit centrifuge technique for the diagnosis of African trypanosomiasis. Acta Tropica 27: 384-386.

## 8. Historique du document

| **Révision** | |
|---|---|
| | Version initiale |

| **Nom et fonction** | **Date** | **Signature** |
|---|---|---|
| Auteur | | |
| Veerle Lejon | 13/Feb/2012 | |
| Révision par | | |
| Philippe Büscher | 13/Feb/2012 | |
| Approuvé par | | |
| Emilie Alirol | 04/Apr/2012 | |

| | |
|---|---|
| | **SOP titre:** Test diagnostic rapide (TDR) de la trypanosomiase humaine Africaine : SD Bioline HAT test |
| | **Projet/Étude :** Evaluation de tests rapides en association avec des prédicteurs cliniques et de laboratoire pour le diagnostic de maladies tropicales négligées dans des patients se présentant avec des troubles neurologiques dans les hôpitaux ruraux au Bandundu, République Démocratique du Congo. |

## 1. Domaine et application

Ce document donne les instructions pour exécuter le test de diagnostic rapide de la trypanosomiase humaine Africaine SD Bioline HAT test.
Le test SD Bioline HAT est un test rapide de détection d'anticorps spécifiques pour *T.b. gambiense* dans le sang. Le test est à utiliser comme un outil de diagnostic de première ligne pour la détection de la maladie du sommeil à *gambiense.* Un résultat positif doit impérativement être confirmé par un test de détection du parasite. Le test peut être fait sur sang capillaire ou sang veineux.

## 2. Responsabilités

| Fonction | Activités |
|---|---|
| Technicien de laboratoire | ▪ Prélève le sang<br>▪ Exécute le test<br>▪ Interprète les tests<br>▪ Enregistre les résultats |

## 3. Procédures

### 3.1 Précautions

- Tous les échantillons de sang sont potentiellement infectieux. Respectez les précautions universelles. METTEZ DES GANTS À USAGE UNIQUE PENDANT TOUTE LA PROCÉDURE **!**

### 3.2 Matériel et échantillons

#### 3.2.1 Matériel fourni dans le kit et conservation

- <u>La cassette SD BIOLINE HAT</u> :
  Une bandelette de test comprenant :
  - o Le conjugué
  - o 2 lignes de test : T1 et T2
  - o Ligne de contrôle

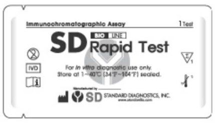


  *Conservation* : à température ambiante (1 - 40°C). NE PAS REFRIGERER !
  La cassette est sensible à l'humidité et à la chaleur. Utilisez les tests immédiatement après ouverture du sachet. N'UTILISEZ PAS le test si le sachet est endommagé.
  Voir **SOP-WP6-QUAL-05** pour la manipulation et le stockage des TDR.

- <u>Diluant (tampon)</u> :
  *Conservation* : à température ambiante (1 - 40°C).
  NE PAS REFRIGERER !
  <u>Remarque</u> : Utilisez uniquement le tampon inclus pour tous les tests appartenant au même kit.

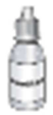

- Brochure explicative

### 3.2.2 Matériel supplémentaire requis

- Gants non stériles, à usage unique
- Minuterie
- Marqueur (crayon, stylo)
- Micropipette de 20 µl
- Des embouts pour micropipette
- Conteneur « bio-hazard »

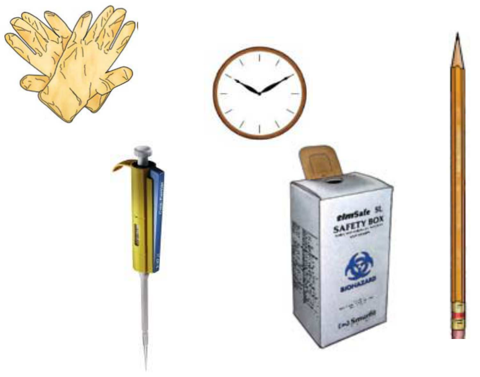

### 3.2.3 Echantillon à examiner

- Sang veineux (prélevé sur héparine) : 20 µl

*Conservation*: Exécutez le test immédiatement ou réfrigérez l'échantillon à 2-8°C pendant 3 jours maximum.
Remarque : Laissez revenir les échantillons à température ambiante avant d'exécuter le test.

## 3.3 Mode opératoire

### 3.3.1 Contrôle de qualité interne

Une ligne de contrôle « C » est inclue dans le système afin d'assurer la validité du test. Si la ligne de contrôle n'apparait pas à la fin du test, le résultat n'est pas valide.

### 3.3.2 Procédure

**1**. Laissez revenir l'échantillon et le TDR à température ambiante

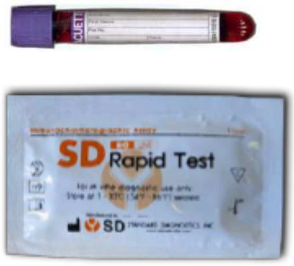


**2**. Vérifiez la date de péremption sur le sachet (NE PAS UTILISER au-delà de la date d'expiration!)

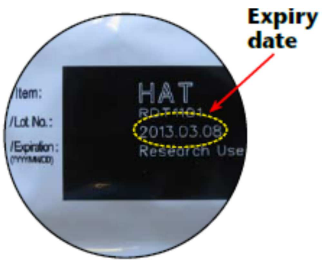


**3**. Mettez les gants

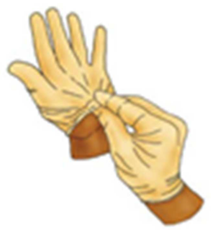

---

**4.** Ouvrez le paquet
Prenez la cassette

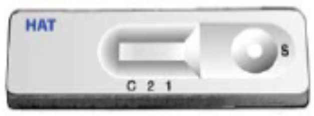


**5**. Ecrivez la date et le numéro du patient sur la cassette

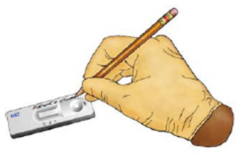

**6**. Aspirez 20 µl de sang à l'aide d'une micropipette (Utilisez un embout neuf pour chaque échantillon)

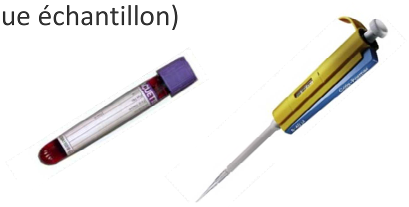

**7**. Déposez les 20 µl de sang dans le puits rond

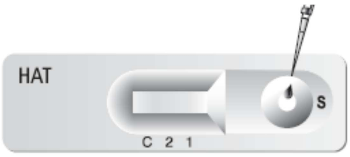


**8**. Ajoutez 4 gouttes de tampon dans le puits rond

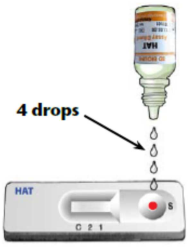


**9**. Lancez la minuterie (15 minutes). Attendez 15 min avant de lire le résultat (NE LISEZ PAS après 30 minutes)

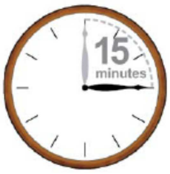


**10.** Prenez une photo du résultat.

**11.** Notez dans le CRF laboratoire les résultats du test, y compris l'intensité des bandes du test, le N° de lot du Kit et le N° de la photo.

## 3.4 Interprétation du test

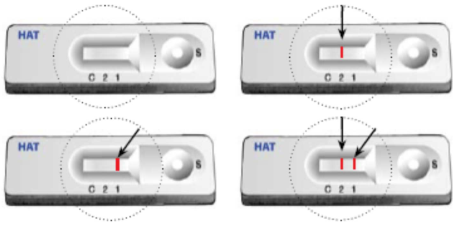

Ligne de contrôle « C » absente

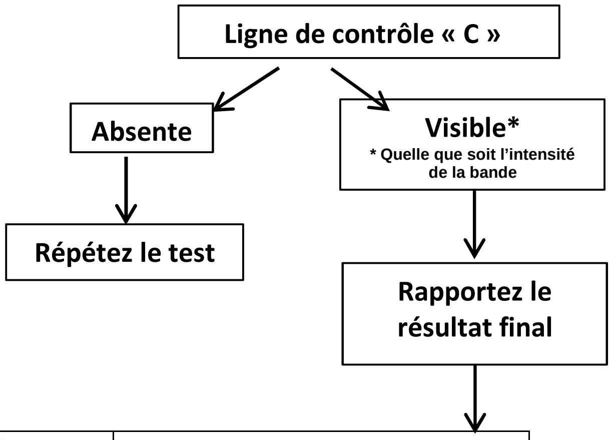


| | | Ligne de contrôle « C » visible | Ligne T2 visible | Ligne T1 visible | Résultat final à rapporter |
|---|---|---|---|---|---|
| 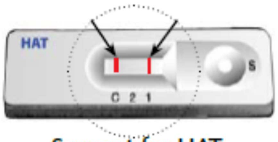  | 1 | Oui | Non | Oui | Positif, suspect pour THA |
| 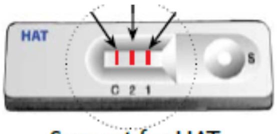  | 2 | Oui | Oui | Oui | Positif, suspect pour THA |
| 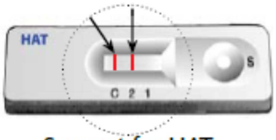  | 3 | Oui | Oui | Non | Positif, suspect pour THA |
| 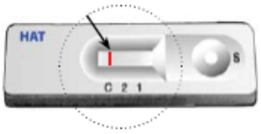  | 4 | Oui | Non | Non | Négatif |

### 3.5 Déchets et nettoyage

- Jetez les cassettes utilisées dans un conteneur « bio-hazard ».
- Jetez l'excès du diluant (après avoir utilisés tous les tests du kit) dans un conteneur « bio-hazard ».

## 4. Registres et archives

| **Appendices & formulaires à completer** | |
|---|---|
| **Numéro** | **Titre** |
| 1 | CRF laboratoire |

## 5. Références

Instruction du producteur, version 2011.03

## 6. Historique du document

| **Révision** | |
|---|---|
| SOP-WP2-LAB-08-V01-26Sep2012 | Version initiale |
| SOP-WP2-LAB-08-V02-11Feb2013 | Ajout de la référence SOP-QP6-QUAL-05 |
| SOP-WP2-LAB-08-V03-19Mar2014 | - Adaptation de la conservation du sang veineux<br>- Correction du temps de lecture maximal |

| **Nom et fonction** | **Date** | **Signature** |
|---|---|---|
| *Auteur* | | |
| Barbara Barbé | 19/03/2014 | |
| *Révisé par* | | |
| | | |
| *Approuvé par* | | |
| Ninon Horié | 19/03/2014 | |

| | |
|---|---|
| nidiag — Better Diagnosis of Neglected Infectious Diseases | **SOP titre:** Test diagnostic rapide (TDR) de la trypanosomiase sur sang total: Gambiense-Sero-K-Set (Coris BioConcept ). |
| | **Projet/Étude** : Evaluation de tests rapides en association avec des prédicteurs cliniques et de laboratoire pour le diagnostic de maladies tropicales négligées dans des patients se présentant avec des troubles neurologiques dans les hôpitaux ruraux au Bandundu, République Démocratique du Congo. |

# 1. Domaine et application

Le test Gambiense-Sero-K-Set est un test rapide de détection d'anticorps spécifiques pour *T.b. gambiense* dans le sang. Le test est à utiliser comme un outil de diagnostic de première ligne pour la détection de la maladie du sommeil à *gambiense.* Un résultat positif doit impérativement être confirmé par un test de détection du parasite. Le test peut être fait sur sang digital ou sang veineux.
Cette SOP est uniquement applicable aux analyses d'échantillons sanguins réalisées dans le cadre de la validation en phase III du test Gambiense-Sero-K-Set de Coris BioConcept.

# 2. Responsabilités

| Fonction | Activité |
|---|---|
| Technicien de labo / infirmier | ▪ Prise de sang **sur héparine (Li ou K)** |
| Technicien de labo | ▪ Exécution du test Gambiense-Sero-K-Set<br>▪ Interprétation du résultat<br>▪ Enregistrement des résultats |

# 3. Procédures

## 3.1 Conservation du test Gambiense-Sero-K-Set

- De préférence, conserver le test à 4°C. **Ne pas congeler**.
- Si conservation à température ambiante, garder à l'abri du soleil.
- Voir **SOP-WP6-QUAL-05** pour la manipulation et le stockage des TDR.

## 3.2 Précautions

- Ne pas ouvrir l'enveloppe de protection de la cassette avant de l'utiliser.
- Utiliser le tampon spécifique au kit.
- Respecter la date de péremption.
- Tous les échantillons de sang sont potentiellement infectieux. Respecter les précautions universelles. METTRE DES GANTS À USAGE UNIQUE PENDANT TOUTE LA PROCÉDURE !
- Respecter les BPL.

## 3.3 Matériel et échantillons

### 3.3.1 Matériels fournis dans le kit

- 20 cassettes Gambiense-Sero-K-Set, emballées individuellement dans une enveloppe de protection avec un déssicant.
- 20 tubes capillaires héparinés.
- 1 micro-poire pour capillaires
- 20 lancettes de prélèvement sanguin.
- Un flacon de 15 ml avec compte-goutte contenant le tampon de migration spécifique au Gambiense-Sero-K-Set.

### 3.2.2 Matériels supplémentaires requis

- Gants
- Papier absorbant
- Cotons & désinfectant
- Test sur sang veineux : Pipette 30 µl, embouts jaune

### 3.2.3 Echantillon à examiner

<u>Test sur sang veineux :</u>

- Sang total, prélevé sur héparine: 30 µl

  *Conservation : Sang total sur héparine* : Exécuter le test immédiatement, si ce n'est pas possible, réfrigérer l'échantillon à 2-8°C pour 3 jours maximum.

  *Remarque* : Laisser revenir les échantillons à température ambiante avant d'exécuter le test. Congélations et décongélations répétitives peuvent affecter les résultats du test.

<u>Test sur sang digital :</u>

- Piquer avec une micro-lancette le doigt du patient préalablement désinfecté.
- Essuyer la première goutte de sang.
- Remplir à moitié un tube capillaire hépariné en évitant les bulles d'air (fig 1).
- Incliner le tube capillaire à plusieurs reprises dans les 2 sens afin de bien mélanger le sang à l'héparine.

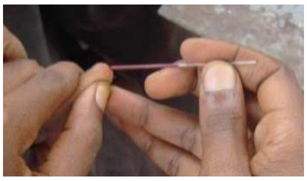

Fig 1 : Prise de sang en tube capillaire

## 3.4 Mode opératoire

### 3.4.1 Procédure

- Ouvrir l'enveloppe de protection de la cassette en la déchirant à partir des encoches prévues à cet effet.

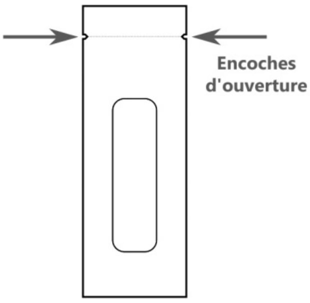


- Vérifier que deux lignes VERTES sont visibles dans la fenêtre de lecture (une au niveau de la ligne contrôle notée «C» et l'autre au niveau de la ligne test notée «T»). Dans le cas contraire, la cassette n'est pas valide. Il faut alors la jeter et recommencer la procédure avec une nouvelle cassette.
- Ecrire la date et le numéro du patient sur la cassette, au niveau de la zone prévue à cet effet (zone rugueuse située entre les fenêtres de dépôts et de lecture).
- Repousser partiellement la cassette dans l'enveloppe de protection de telle sorte que la fenêtre de dépôt soit visible mais que la fenêtre de lecture soit cachée.

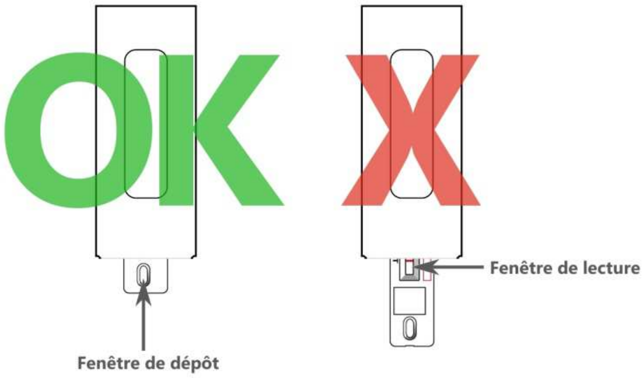


<u>Test sur sang digital :</u>

- Monter la micro-poire sur le tube capillaire et déposer une goutte de sang (30 µl) dans la fenêtre de dépôt de la cassette. Afin d'avoir des gouttes de volume constant, bien tenir le capillaire à la verticale pour laisser tomber la goutte librement et sans toucher la fenêtre de dépôt.

<u>Test sur sang veineux :</u>

- Distribuer avec une micropipette 30 µl d'échantillon sur la zone de dépôt de l'échantillon (flèche).
- Déposer **immédiatement 2 gouttes de tampon** par-dessus le sang, dans la fenêtre de dépôt.
- Refermer le bouchon du flacon de tampon.
- **Repousser immédiatement la cassette dans l'enveloppe** de protection jusqu'à ce que la fenêtre dépôt soit cachée dans l'enveloppe.

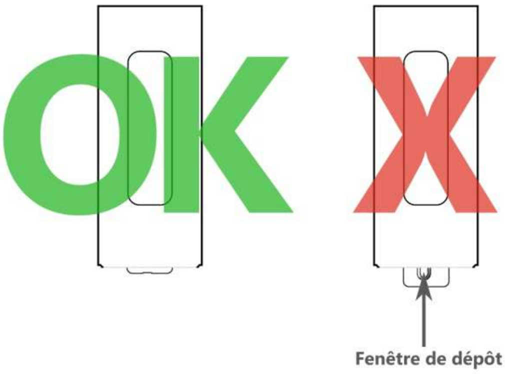


- Laisser réagir le test 15 minutes.
- Après les 15 minutes de réaction, sortir complètement la cassette de l'enveloppe, lire le résultat dans la fenêtre de lecture et prendre une photo de la cassette entière.

### 3.4.2 Interprétation du test

- L'interprétation du résultat se fait de la façon suivante:
  1. Une ligne ROUGE au niveau de la ligne contrôle («C») et une ligne rouge au niveau de la ligne test («T») = test valide positif.
  2. Une seule ligne ROUGE au niveau de la ligne contrôle («C») = test valide négatif.
  3. PAS de ligne ROUGE au niveau de la ligne contrôle («C») avec une ligne rouge au niveau de la ligne test = test INVALIDE.
  4. PAS de ligne ROUGE au niveau de la ligne contrôle («C») sans ligne rouge au niveau de la ligne test = test INVALIDE.

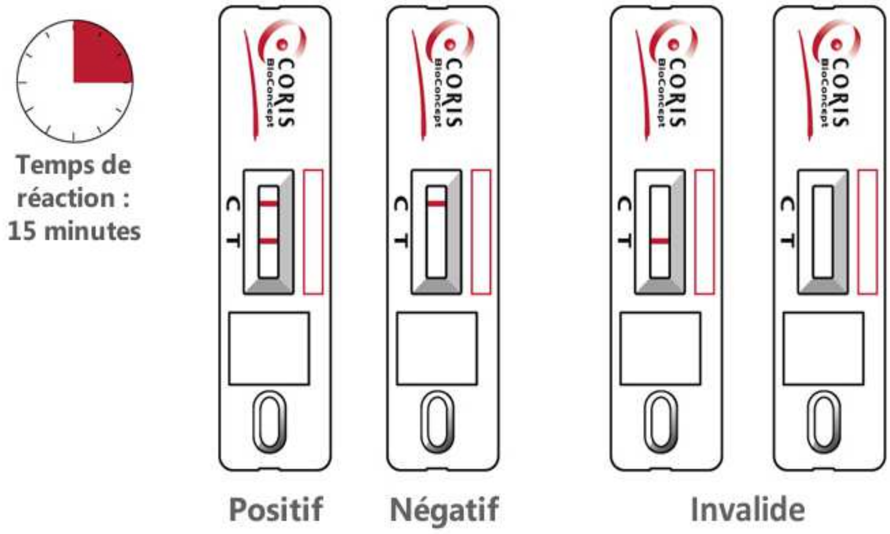


- Dans les configurations 3 et 4, où le résultat est invalide, on ne peut rien conclure. Il faut alors **jeter la cassette** et recommencer la procédure avec une nouvelle cassette.
- *Remarque :* **Tout signal positif** doit être considéré comme tel, **quelle que soit l'intensité** de la ligne test (celle-ci peut être extrêmement faible)

## 4. Définitions & abréviations

- RDT = Rapid Diagnostic Test.
- HAT = Human African Trypanosomiasis.
- SOP = Standard Operating Procedure.
- *T.b.gambiense* = *Trypanosoma brucei gambiense*.
- BPL = Bonnes Pratiques de Laboratoire.

## 5. Registres et archives

| **Appendices & formulaires à compléter** | |
|---|---|
| **Numéro** | **Titre** |
| 1 | CRF laboratoire |

## 6. Historique du document

| **Révision** | |
|---|---|
| SOP-WP2-LAB-09-V01-13aug2012 | Version initiale |
| SOP-WP2-LAB-09-V02-11Feb2013 | Ajout de la référence SOP-WP6-QUAL-05 |

| **Nom et fonction** | **Date** | **Signature** |
|---|---|---|
| *Auteur* | | |
| Quentin Gilleman (Coris BioConcept) | 11/02/2013 | |
| *Révisé par* | | |
| Barbara Barbé | 11/02/2013 | [signature] |
| *Approved by* | | |
| Emilie Alirol | 11/02/2013 | [signature] |

| | |
|---|---|
| | **SOP titre :** Confection de la goutte épaisse, coloration de Giemsa et examen microscopique. |
| | **Projet/Étude :** NIDIAG |

# 1. Domaine et application

Ce document fournit des instructions pour préparer une goutte épaisse, pour faire une coloration de Giemsa et pour faire la lecture microscopique.
La goutte de sang est utilisée dans cette étude pour la détection de la malaria (Plasmodium spp.), des fièvres récurrentes (Borrelia spp.) et de la maladie du sommeil (Trypanosoma spp.)

# 2. Responsabilités

| Fonction | Activités |
|---|---|
| Technicien de laboratoire | ▪ Prélève le sang<br>▪ Exécute le test<br>▪ Prépare la dilution de Giemsa et vérifie le pH.<br>▪ Stocke correctement le Giemsa concentré et le Giemsa dilué<br>▪ Interprète le résultat<br>▪ Enregistre les résultats<br>▪ Entretien le microscope |

# 3. Procédures

## 3.1 Précautions

- Tous les échantillons de sang sont potentiellement infectieux. Respectez les précautions universelles. METTEZ DES GANTS À USAGE UNIQUE PENDANT TOUTE LA PROCÉDURE **!**
- Le Giemsa est inflammable, manipulez ce produit loin d'une flamme.

## 3.2 Matériel et échantillons

### 3.2.2 Matériel requis

- Gants non-stériles, à usage unique
- Lames de porte-objet neuves
- Giemsa concentré (Merck N° 1.09204)
- Comprimés pH 7.2 (Merck N° 9468)
- pH-mètre et solution de calibration
- Bouteille à 1000 ml
- Eau propre ou courante
- Flacon compte-gouttes
- Pipettes pasteur
- Cylindre gradué de 10 ml
- Becher à 200 ml
- Support de coloration
- Râtelier à lames
- Minuterie
- Microscope (objectif 100 x)
- Huile à immersion
- 2 compteurs à 1 touche
- Boîte de conservation pour lames de porte-objet
- Tissu

### 3.2.3 Echantillon à examiner

- Sang anti-coagulé veineux prélevé sur EDTA : ± 10 µl
- *Stabilité* : au maximum une heure à température ambiante (pour éviter des déformations morphologiques des parasites de *Plasmodium*)

## 3.3 Mode opératoire

### 3.3.1 Préparation de la goutte épaisse

1. Trempez les lame de porte-objet neuves dans l'alcool dénaturée pendant 30 minutes et essuyez avec un chiffon propre.
2. Mettez des gants à usage unique non stériles.
3. Ecrivez le code du patient sur la lame porte-objet.
4. Déposez une petite goutte de sang (±10 µl) au centre de la lame avec une pipette pasteur.
5. Mélangez le sang immédiatement pendant 20 – 30 secondes à l'aide du coin d'une autre lame
6. Etalez le sang sur une surface de 1 à 2 cm de diamètre.
   Bonne épaisseur de l'étalement = quand il est possible de lire les caractères d'un texte à travers la goutte épaisse.
7. Laissez sécher la lame sur une surface plane à température ambiante SANS CHAUFFAGE et SANS EXPOSITION AU SOLEIL pour éviter la fixation des globules rouges qui empêcherait leur hémolyse.

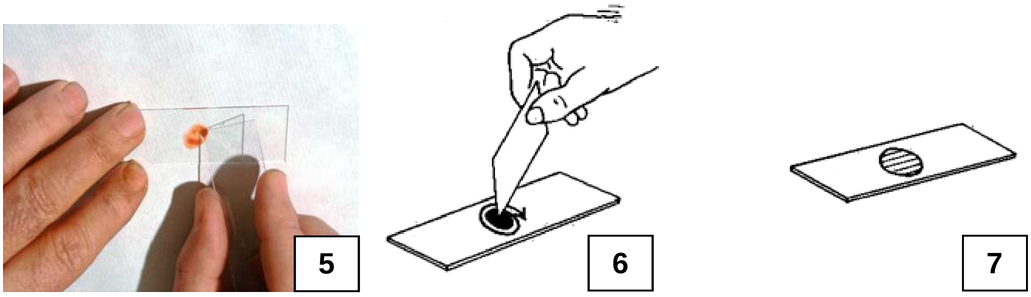


### 3.3.2 Coloration de la goutte épaisse au colorant de Giemsa 3,5 %

#### 3.3.2.1 Préparation de l'eau tamponnée

- Dissolvez un comprimé de pH 7,2 dans 1 litre d'eau propre.
- Vérifiez le pH de la solution préparée (voir Procédure pH-mètre : annex 1 et annex 2). Le pH doit être compris entre 7,0 et 7,4.
- Ecrivez « Eau tamponnée, la date de préparation et la date d'expiration » sur la bouteille.

➔ *Stabilité* : un mois à température ambiante. Remplacez la solution si elle est légèrement trouble après agitation.

#### 3.3.2.2 Préparation du Giemsa dilué à 3,5 % (à faire juste avant la coloration)

- Versez la quantité de Giemsa concentré nécessaire pour environ une semaine travail dans un petit flacon compte-gouttes. Utilisez ce flacon pour faire les dilutions fraîches de Giemsa à 3,5% avant chaque coloration.
- Préparez la quantité de Giemsa dilué suffisante pour le nombre de lames à colorer. : par lame :
    - Versez 4 ml d'eau tamponnée dans un cylindre gradué de 10 ml
    - Ajoutez 5 gouttes de Giemsa concentré avec une pipette pasteur

- o Mélangez bien
- → *Stabilité :* une heure à température ambiante.

#### 3.3.2.3 Coloration de la goutte épaisse au colorant de Giemsa 3,5 %

1. Attendez le séchage complet de la goutte épaisse avant de la colorer (± 20 minutes)
2. Déposez la lame sur un support de coloration. Les lames ne peuvent pas se toucher. Remarque : N'utilisez pas de cuve de coloration pour éviter une contamination entre les lames.
3. Diluez la quantité de Giemsa nécessaire (voir « 3.3.2.2 Préparation du Giemsa dilué à 3,5% »)
4. Couvrez complètement chaque lame de Giemsa dilué à 3,5 %
5. Laissez agir pendant 20 minutes.
6. Egouttez le colorant.
7. Rincez prudemment chaque lame dans un bécher contenant de l'eau propre.
8. Laissez sécher la goutte épaisse sur un râtelier à lames.

### 3.3.3 Examen microscopique de la goutte épaisse

#### 3.3.3.1 Détection des parasites/bactéries sanguin(e)s

- Enlevez les gants pour faire l'examen microscopique (objectif 100x, à immersion).
- Parcourez la lame champ par champ et comptez le nombre de champs examinés.
- Après 100 champs sans détection d'un parasite ou d'une bactérie → Rapportez comme recherche négative.
- Si un parasite ou bactérie est trouvé :
  - o Faites l'identification d'espèce (Voir « 3.3.3.2 Identification de l'espèce ») et rapportez l'espèce dans le cahier de laboratoire.
  - o Pour *Plasmodium spp*, rapportez aussi les stades de parasites présents dans la goutte épaisse (trophozoïtes, schizontes, gamétocytes).
  - o Pour *Plasmodium spp.* → Calculez la densité parasitaire (Voir « 3.3.3.3 Densité parasitaire »).

| Élément sanguin | Couleur après la coloration de Giemsa |
|---|---|
| Globules rouges | Hémolysés (non visibles) |
| Globules blancs | Magenta (rose-pourpre) |
| Plaquettes | Rose pâle léger |
| Cytoplasme de *Plasmodium spp.* | Bleu – mauve |
| Chromatine de *Plasmodium spp.* | Rouge foncé - pourpre |
| Granulation de Schüffner | Pourpre – rouge |
| Pigment malarien (hémozoïne) | Jaune/brun ou noir |
| *Borrelia* spp. | Bleu |
| Cytoplasme de *Trypanosoma spp.* | Bleu – mauve |
| Noyau et kinétoplaste de *Trypanosoma spp* | Rouge – pourpre |
| Membrane ondulante et flagelle de *Trypanosoma spp.* | Bleuté |

#### 3.3.3.2 Identification de l'espèce

- *Plasmodium spp.*

Les caractéristiques les plus importantes de chaque espèce de *Plasmodium* :

| ***P. falciparum*** | | ***P. vivax*** | |
|---|---|---|---|
| – Image uniforme<br>– Trophozoïtes : fins, formes de bague, 1-2 grains de chromatine<br>– Pas de schizontes (excepté en cas grave)<br>– Gamétocytes : forme de banane | 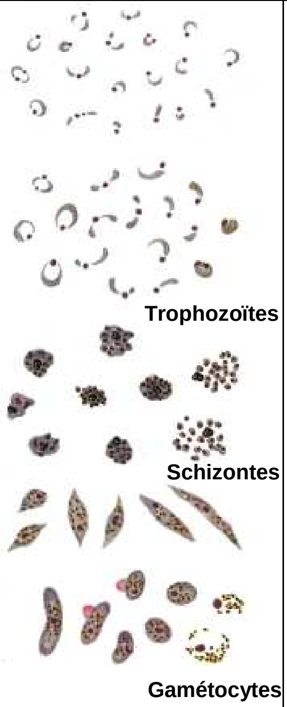 | – Tous les stades<br>– Forme amiboïde (trophozoïtes âgés)<br>– Schizontes mûrs ≥ 16 noyaux<br>– Fantômes de globules rouges (granulation de Schüffner fines) | 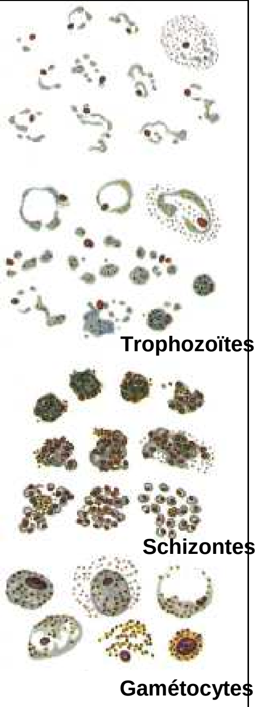 |

| ***P. ovale*** | | ***P. malariae*** | |
|---|---|---|---|
| – Tous les stades<br>– 1 grain de chromatine « lourd »<br>– Parasites compactes<br>– Schizontes mûrs à 8 noyaux<br>– Fantômes de globules rouges (granulation de Schüffner) | 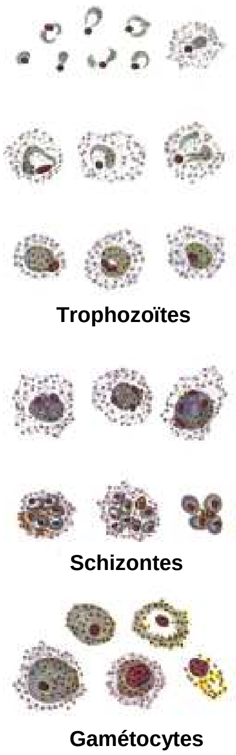 | – Tous les stades<br>– Parasites petits, compactes, foncés<br>– Schizontes mûrs à 6-12 noyaux (forme de marguerite)<br>– Pigment malarien précoce même dans les trophozoïtes jeunes | 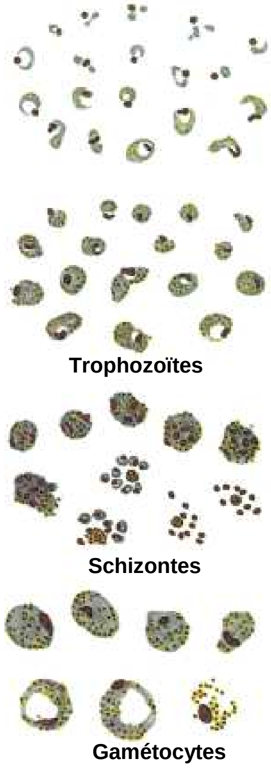 |

➔ Consultez les planches pour le diagnostic du paludisme de l'OMS !

➔ Rapportez l'espèce et les stades présents dans la goutte épaisse dans le cahier de laboratoire.

**Les caractéristiques du paludisme grave (*P. falciparum*) :**

- Hyper parasitémie : > 250.000 parasites/µl de sang (zone endémique)
- Présence de schizontes dans le sang circulant (et donc dans la goutte épaisse)
- Prédominance des trophozoïtes matures (pigment malarien présent en > 20% des trophozoïtes)
- Hémozoïne phagocytée dans les globules blancs

➔ Rapportez les caractéristiques du paludisme graves dans le CRF laboratoire et prévenez immédiatement le médecin prescripteur.

**Borrelia spp. en goutte épaisse**

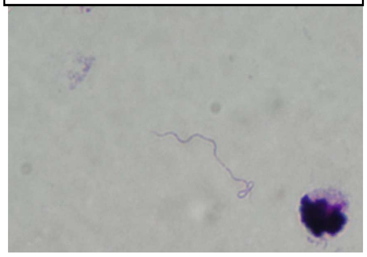

- *Borrelia spp.*
  - Bactérie Gram-négative (spirochète)
  - Fine et longue : 10-20 µm x 0,5 µm
  - Forme hélicoïdale
  - Couleur bleu (au Giemsa)
- *Trypanosoma spp.*
  - *Trypanosoma brucei gambiense* : Afrique de l'Ouest et Centrale
    *Trypanosoma brucei rhodesiense* : Afrique de l'Est
  - 4 des 5 caractéristiques suivantes doivent être présentes:
    - Taille entre 15 et 25 µm
    - Cytoplasme : fusiforme, bleuâtre
    - Noyau : assez gros, rougeâtre
    - Kinétoplaste : petit et souvent central. De couleur rougeâtre.
    - Membrane ondulante : de couleur bleu, partant du kinétoplaste (sub-terminal) et se terminant par le flagelle
  - Dimorphes : des formes longues (en multiplication) et courtes (infectantes pour le vecteur)
  - Pas de différence morphologique entre les deux espèces (différence géographique)

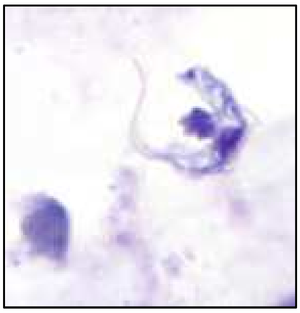

***Trypanosoma spp.* en goutte épaisse**

### 3.3.3.3 Densité parasitaire *(Plasmodium spp.)*

- Prenez 2 compteurs à touche (un dans chaque main)
- Parcourez la lame champ par champ (objectif 100 x)
- Comptez ± 200 globules blancs et en même temps les parasites asexuées de *Plasmodium* rencontrées. Ne comptez pas les gamétocytes de *P. falciparum* (= parasites sexuées).
  - Si aucune parasite n'est rencontrées dans 100 champs → résultat est négatif.
  - Si > 100 parasites par champs → comptez 5 champs et calculez la densité parasitaire
  - Si avoir compté 200 globules blancs et
    ≥ 100 parasites comptés → calculez la densité parasitaire
    < 100 parasites comptés → continuez le comptage jusqu'à 500 globules blancs et calculez la densité parasitaire

- Utilisez la formule suivante pour calculer la densité parasitaire :

$$\frac{\text{Nombre de parasites asexués comptés}}{\text{Nombre de globules blancs comptés}} \text{ x concentration leucocytaire} = \text{Nombre de parasites/}\mu\text{l de sang}$$

➔ Utilisez la numération leucocytaire obtenue par le HemoCue WBC DIFF. Multipliez le résultat par 1000 pour obtenir l'unité par µl.
Remarque : 50.000 parasites par µl de sang correspondent à 1% d'hématies parasitées.

### 3.4 Stockage et conservation des lames pour relecture

- Déposez délicatement les lames sur un papier toilette pour éliminer l'huile à immersion.
- Rangez les lames nettoyées dans des boîtes de rangement pour lames porte-objet et étiquetez clairement les boîtes (Nom de l'étude, numéro de la boîte, type de prélèvement et coloration, numéro du premier et du dernier patients).

## 4. Définitions & abréviations

EDTA : acide éthylène diamine tétra-acétique
spp. : species

## 5. Registres et archives

| Appendices & formulaires à compléter | |
|---|---|
| **Numéro** | **Titre** |
| 1 | CRF "laboratoire" |
| SOP-WP2-LAB-20 –annex 1 | Bench Aid pH mètre |
| SOP-WP2-LAB-20 –annex 2 | Etalonnage pH mètre |

## 6. Historique du document

| Révision | |
|---|---|
| SOP-WP2-LAB-20-V01-13Aug2012 | Version initiale |
| SOP-WP2-LAB-20-V02-18Sep2012 | 3.3.3.3 Numération leucocytaire par HemoCue WBC DIFF<br>Annex 1 et annexe 2 ajouté. Formulaires à compléter : Annex2 ajouté |

| Nom et fonction | Date | Signature |
|---|---|---|
| *Auteur* | | |
| Barbara Barbé | 25/05/2012 | |
| *Révisé par* | | |
| Philippe Gillet | 16/09/2012 | |
| *Approuvé par* | | |
| Veerle Lejon | 18/09/2012 | |

# B E N C H A I D : Correction pH

***pH10 pH/Temperature Pen, VWR International***

## MATERIELS:

- pH mètre
- Tampon 4,1 – 7 – 10
- Solution NaOH à 1N et 0,1N
- Solution HCl à 1N et 0,1N
- Papier tissue
- Eau distillée (pissette)

## ETAPE 1: AVANT LECTURE

1. Si pas utilisée depuis longtemps, plongez l'électrode dans le tampon pH4 pendant 10 min avant de l'allumer.
2. Appuyez le bouton marche/arrêt (ON/OFF) pour 2 secondes.
3. Rincez la sonde avec l'eau distillée et essuyez avec papier douce.
4. Testez le fonctionnement de l'appareil en mesurant le pH du tampon pH7 (ou pH4).
5. Inscrire la valeur sur la feuille de contrôle.
6. Si la valeur ne correspond pas avec celui attendu faire l'étalonnage.
7. Au moins une fois par semaine faire la calibration.

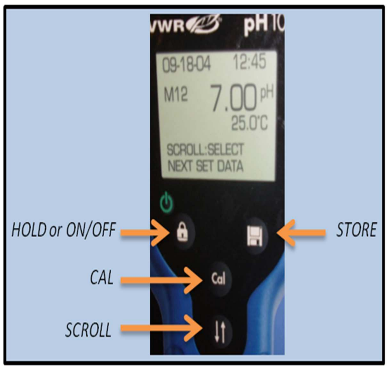


***Toujours rincer avec de l'eau distillée et essuyer la sonde entre les solutions et tampons différents.***

## ETAPE 2: ETALLONAGE

1. Appuyer « CAL » pendant 2 secondes.
2. Immergez la sonde dans le tampon pH7.
3. Attendez jusqu'à ce que « WAIT » ait disparu de l'écran.
4. Immergez la sonde dans le tampon pH4 (l'écran affiche : « Buffer 7/4 ») .
5. Attendez jusqu'à ce que « WAIT » ait disparu de l'écran.
6. Immergez la sonde dans le tampon pH10.
7. Après « WAIT », le mètre change dans le mode « MEASURE » (= lecture)

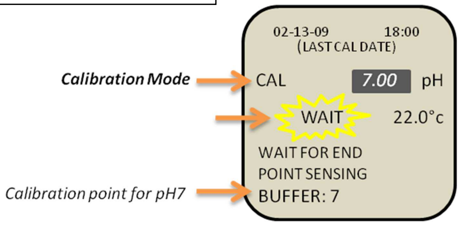

SOP-WP2-LAB-20, **Annex 1**

# B E N C H A I D : Correction pH

***pH10 pH/Temperature Pen, VWR International***

## ETAPE 3: LECTURE

1. Immergez la sonde dans la solution.
2. Attendez que la valeur pH sur l'écran soit stable.
3. Appuyer « **HOLD** » (=**BLOQUER**) pour bloquer la valeur pH.
4. Notez/vérifiez la valeur.
5. Si nécessaire, adaptez le pH avec la solution.
   - 1N ou 0,1N NaOH pour un pH plus haut
   - 1N ou 0,1N HCl pour un pH plus bas
6. Appuyez « ***HOLD*** » ***pour débloquer***.
7. Rincez avec l'eau distillée et séchez avec un tissu.
8. Répétez les points 1-5 pour la prochaine lecture.
9. Appuyez ON/OFF pour 2 secondes pour éteindre l'appareil.

**Valeur bloqué**
**Mode "Lecture"**

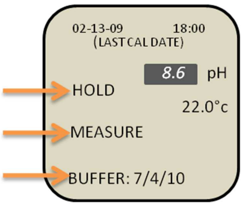


## MAINTENANCE

- Rincez l'électrode après usage avec l'eau distillée.
- Gardez l'électrode dans un environnement humide (eau robinet) en utilisant l'éponge dans le capuchon.
- Pour un stockage à longue durée, plongez dans le tampon pH4.

## Resolution problèmes

| | |
|---|---|
| Si l'écran affiche « OVER » : | - Renouvelez le tampon<br>- Vérifiez la sonde ou l'électrode |
| Si rien ne s'affiche à l'écran : | - Vérifiez les batteries |

Mise à jour 14/02/2011
INRB Service de Microbiologie

## NIDIAG SOP-WP2-LAB-20-V02-18Sep2012-annex2

### Etallonage pH mètre

| | | |
|---|---|---|
| **ID Appareil:** | | |

| | | | |
|---|---|---|---|
| **Tampon pH 4,01** | | | |
| Date: | | Lot: | Valeur Cible: |
| **Tampon pH7** | | Date | |
| Date: | | Lot: | Valeur Cible: |
| **Tampon pH10** | | Date | |
| Date: | | Lot: | Valeur Cible: |

| Date | pH | | | Etallonage | Resp | Commentaires |
|---|---|---|---|---|---|---|
| | 4.01 | 7 | 10 | | | |
| | | | | | | |
| | | | | | | |
| | | | | | | |
| | | | | | | |
| | | | | | | |
| | | | | | | |
| | | | | | | |
| | | | | | | |
| | | | | | | |
| | | | | | | |
| | | | | | | |
| | | | | | | |
| | | | | | | |
| | | | | | | |
| | | | | | | |
| | | | | | | |
| | | | | | | |
| | | | | | | |
| | | | | | | |
| | | | | | | |
| | | | | | | |
| | | | | | | |
| | | | | | | |
| | | | | | | |
| | | | | | | |
| | | | | | | |
| | | | | | | |
| | | | | | | |
| | | | | | | |
| | | | | | | |

| | |
|---|---|
| | **SOP titre :** CareStart Malaria pLDH |
| | **Projet/Étude** : NIDIAG : Evaluation de tests rapides en association avec des prédicteurs cliniques et de laboratoire pour le diagnostic de maladies tropicales négligées dans des patients se présentant avec des troubles neurologiques dans les hôpitaux ruraux au Bandundu, République Démocratique du Congo. |

# 1. Domaine et application

Ce document fournit les instructions pour exécuter le test diagnostic rapide du paludisme CareStart Malaria pLDH. Ce test rapide détecte le présence d'antigènes spécifiques à *Plasmodium falciparum* (Pf-pLDH, parasite lactate déhydrogénase et à toutes les espèces de Plasmodium (Pan-pLDH : parasite lactate déhydrogénase) : *P. falciparum, P. vivax, P. ovale* et *P. malariae*. Ce test apporte une aide pour le diagnostic du paludisme. Attention : Ce test ne permet pas toujours de différencier une infection à *Plasmodium falciparum* d'une infection mixte (*P. falciparum* associé à une ou plusieurs autres espèces de *Plasmodium*).

# 2. Responsabilités

| Fonction | Activités |
|---|---|
| Technicien de laboratoire | ▪ Prélève le sang<br>▪ Exécute le test<br>▪ Interprète le résultat<br>▪ Enregistre les résultats |

# 3. Procédures

## 3.1 Précautions

- Tous les échantillons de sang sont potentiellement infectieux. Respectez les précautions universelles. METTEZ DES GANTS À USAGE UNIQUE PENDANT TOUTE LA PROCÉDURE **!**

## 3.2 Matériel et échantillons

### 3.2.1 Matériel fourni dans le kit et conservation

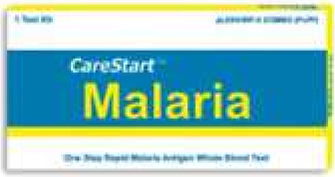


- Cassette CareStart Malaria pLDH
  *Conservation* : entre 4 et 30°C. NE PAS CONGELER !
  Utilisez les tests immédiatement après ouverture du sachet.
  NE PAS UTILISER le test si le sachet est endommagé.
- Tampon (diluant)
  *Conservation* : entre 4 et 30°C. NE PAS CONGELER !
  Remarque : Utilisez seulement le tampon inclus dans le kit pour les tests appartenant à ce kit.
- pipette de 5 µl
- Brochure explicative

### 3.2.2 Matériel supplémentaire requis

- Gants, non stérile, à usage unique
- Marqueur
- Minuterie
- Micropipette de 5 µl
- Embouts de micropipette
- Conteneur « bio-hazard »

### 3.3.3 Echantillon à examiner

- Sang total, prélevé sur EDTA: 5 µl

  *Conservation : Sang total sur EDTA* : Exécutez le test immédiatement, si ce n'est pas possible, réfrigérez l'échantillon à 2-8°C pour 3 jours maximum, ou congelez à -20°C pour des périodes plus longues.

Remarque : Laissez revenir les échantillons à température ambiante avant d'exécuter le test. Congélations et décongélations répétitifs peuvent affecter les résultats du test.

## 3.4 Mode opératoire

### 3.4.1 Contrôle de qualité interne

Une ligne de contrôle « C » est inclue dans le système afin d'assurer la validité du test. Si la ligne de contrôle n'apparait pas à la fin du test, le résultat n'est pas valide.

### 3.4.2 Procédure

1. Laissez revenir tous les réactifs et les échantillons à température ambiante.
2. Mettez des gants à usage unique.
3. Vérifiez la date de péremption. NE JAMAIS utiliser après la date de péremption.
4. Ouvrez l'emballage et enlevez la cassette.
5. Identifiez la cassette avec le numéro du patient et la date.
6. Ajoutez avec une micropipette 5 µl d'échantillon dans le puits de l'échantillon « S ».
7. Ajoutez 2 gouttes de tampon (~ 60 µl) dans le puits du tampon « A ».
8. Lancez la minuterie (20 minutes).
9. Lisez le test après 20 minutes.
10. Prenez une photo du résultat.
11. Notez dans le CRF laboratoire les résultats du test, y compris l'intensité des bandes du test, le N° de lot du Kit et le N° de la photo

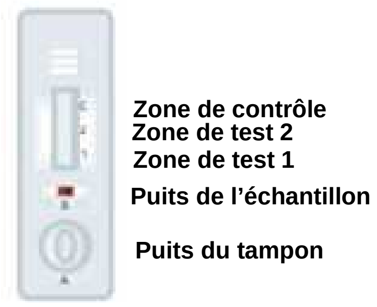

## 3.5 Interprétation du test

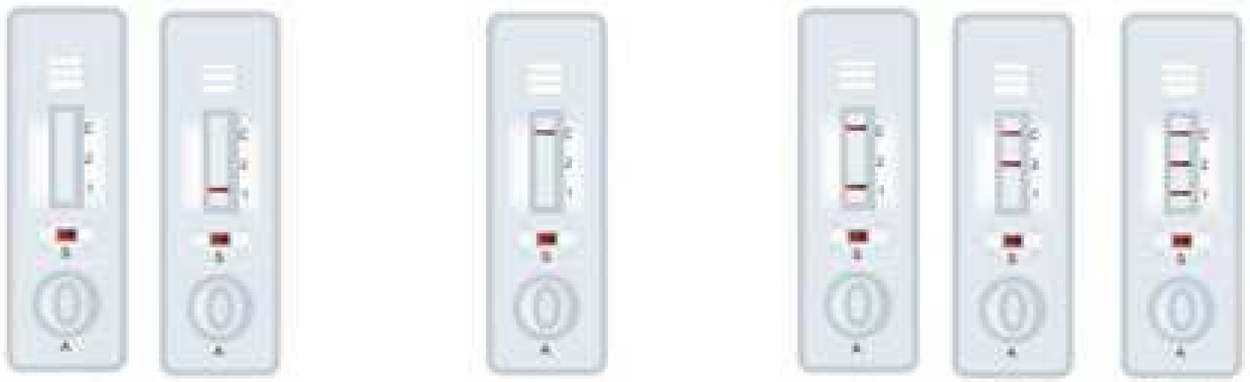

**Invalide** — **Négatif** — **Positif** *P. falciparum* — **Positif** *Plasmodium Non-falciparum* — **Positif** *P. falciparum ou infection mixte*

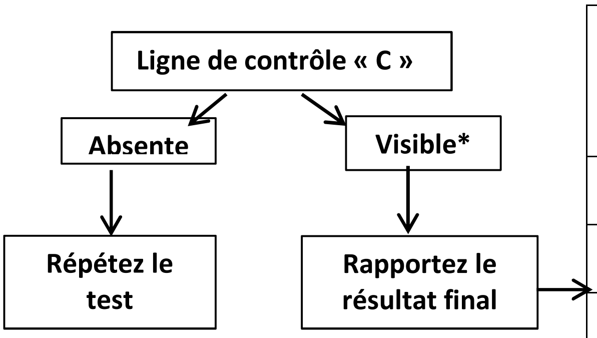


| | Ligne de contrôle « C » visible | Ligne de zone 1 visible | Ligne de zone 2 visible | Résultat final à rapporter |
|---|---|---|---|---|
| 1 | Oui | Non | Non | Négatif |
| 2 | Oui | Oui | Non | Infection *à P. falciparum* |
| 2 | Oui | Non | Oui | Infection à Plasmodium non-*falciparum* |
| 3 | Oui | Oui | Oui | *Infection à P. falciparum* infection mixte possible (*P. falciparum* avec un *Plasmodium* non-*falciparum*) |

## 3.6 Déchets, nettoyage

- Jetez les cassettes utilisées dans un conteneur « bio-hazard ».
- Jetez l'excès du tampon d'essai (après avoir utilisé tous les tests du kit) dans un conteneur « bio-hazard ».

# 4. Registres et archives

| **Appendices & formulaires à compléter** | |
|---|---|
| **Numéro** | **Titre** |
| 1 | CRF laboratoire |

# 5. Références

Instruction du producteur, version I0014, July 2005

# 6. Historique du document

| **Révision** | |
|---|---|
| | Version initiale |

| **Nom et fonction** | **Date** | **Signature** |
|---|---|---|
| *Auteur* | | |
| Barbara Barbé | 07/06/2012 | |
| *Révisé par* | | |
| Philippe Gillet | 12/06/2012 | |
| *Approuvé par* | | |
| Veerle Lejon | 12/07/2012 | |

| | |
|---|---|
| | **SOP titre :** Comptage de cellules blanches dans le LCR avec la chambre de comptage "Uriglass" (Menarini) |
| | **Projet/Étude** : NIDIAG : Evaluation de tests rapides en association avec des prédicteurs cliniques et de laboratoire pour le diagnostic de maladies tropicales négligées dans des patients se présentant avec des troubles neurologiques dans les hôpitaux ruraux au Bandundu, République Démocratique du Congo. |

# 1. Domaine et application

La cellule de numération (ou chambre de comptage) "Uriglass" est utilisée pour compter le nombre de globules blancs dans l'urine, le LCR, le sperme ou d'autres liquides corporels de façon standardisée. Cette procédure décrit la façon dont elle doit être utilisée.

# 2. Responsabilités

| Fonction | Activités |
|---|---|
| Technicien de labo/infirmier ou investigateur | ▪ Ponction lombaire |
| Technicien de labo | ▪ Comptage de cellules<br>▪ Documentation des résultats dans le cahier de laboratoire |

# 3. Procédures

## 3.1 Précautions

- Le LCR doit être examiné dès que possible, au plus tard 15 minutes après le prélèvement.
- Les trypanosomes qui peuvent être présents dans le LCR sont infectieux, utilisez donc des gants lors des manipulations du LCR.
- Déposez le matériel à usage unique contaminé dans un conteneur jaune (pour matériel contaminé).

## 3.2 Matériel

- Microscope avec oculaires 10x et objectif 10x et 40x
- La lame "Uriglass" qui comprend les éléments suivants:
  - o 10 chambres de comptage
  - o Chaque chambre de comptage contient une grille à compter d'un volume de 1 µl.

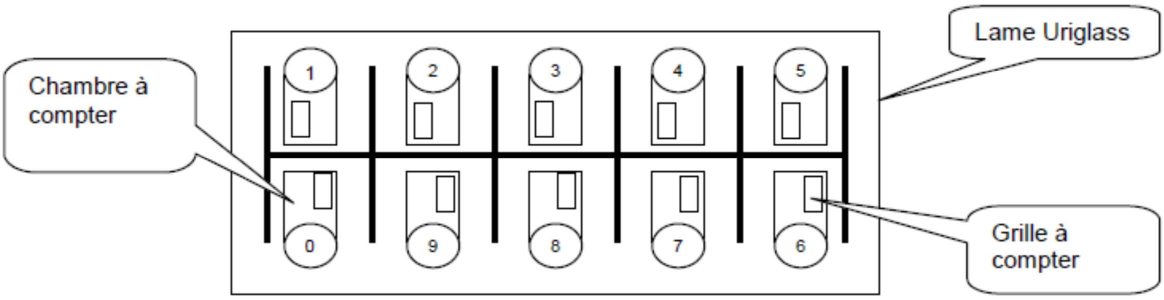

### Description de la grille de comptage

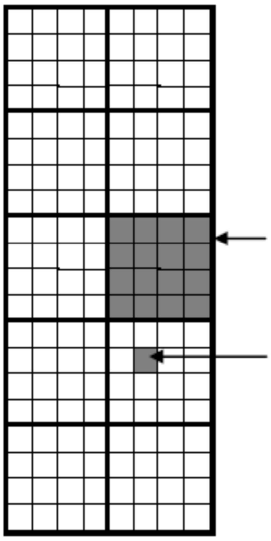

Volume de la grille de comptage:

Largeur = 2 mm
Hauteur = 5 mm
Profondeur = 0,1 mm

**Volume total de la grille de comptage = 1 µl**
( L x H x P = 2 mm x 5 mm x 0,1 mm= 1 mm³ = 1 µl )
La grille est composée de 10 grands carrés.

**Le volume de 1 grand carré = 0,1 µl**
( 1 mm x 1 mm x 0,1 mm = 0,1 mm³ = 0,1 µl )
Chaque grand carré est divisé en 16 petits carrés (secteurs).

**Le volume de 1 petit carré = 0,00625 µl**
(L = 0,25 mm, H = 0,25 mm and P = 0,1 mm.
L x H x P = 0,25 x 0,25 x 0,1 = 0,00625 µl. )

La grille complète consiste en
10 x 16 = 160 petits carrés, et donc un volume total de 1 µl.

## 3.3 Mode opératoire

- Mélangez le LCR doucement en inversant le tube ou avec une pipette.
- Déposez 20 µl de LCR dans la chambre de comptage à l'aide d'une micropipette. La chambre se remplira automatiquement par capillarité.
- Mettez la lame Uriglass sous le microscope.
- Laissez les cellules se sédimenter pendant 1 à 2 minutes.
- Comptez le nombre de globules blancs présents sur la totalité de la grille (objectif 10x). En cas de doute pour différencier les globules blancs des globules rouges, confirmez à l'objectif 40x. Le nombre de globules blancs comptés correspond au nombre de globules blancs par µl de LCR.
- Dans le cas où le nombre de globules blancs est ≤ 20/µl, répétez le comptage en remplissant une nouvelle chambre de comptage.

Si le nombre de globules blancs est élevé (> 1 globule blanc par petit carré), compter le nombre de globules blancs présents dans un grand carré. Le nombre de globules blancs comptés doit alors être multiplié par 10 pour correspondre au nombre de globules blancs par µl de LCR.

| Nombre moyen de globules blancs pour | | Compter | Résultat en globules blancs / µl de LCR |
|---|---|---|---|
| 1 **petit** carré | 1 **grand** carré | | |
| >1 | >16-32 | 1 grand carré | Nombre de globules blancs comptés x 10 |
| ≤1 | ≤ 16 cellules | grille complète | Nombre de globules blancs comptés |

## 3.4 Remarques générales

- Pour mieux différentier les globules blancs des globules rouges, utilisez l'objectif 40 x.
- Les globules blancs qui se trouvent sur les bords d'un carré, doivent être comptés comme indiqué sur la figure: Les globules blancs sur les lignes en haut et à droite, doivent être comptés dans le carré que vous êtes en train de compter, ceux qui se trouvent à gauche et en bas seront comptés dans d'autres carrés.
- Barrez la chambre de comptage au marqueur après comptage pour éviter de la réutiliser

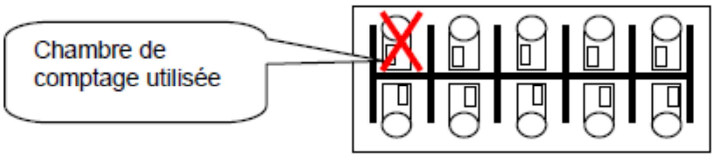


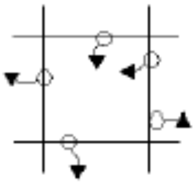

## 4. Documentation des résultats

Notez le résultat (nombre de globules blancs dans le LCR) dans le cahier de laboratoire.

## 5. Définitions & abréviations

LCR : liquide céphalorachidien

## 6. Registres et archives

| Appendices & formulaires à compléter | |
|---|---|
| **Numéro** | **Titre** |
| 1 | Cahier de laboratoire |

## 7. Distributeurs Menarini

- Afrique Francophone:
  ODIFIC, Immeuble Natalys, B.P. 1093, Cotonou - BeninMedical Information : Benin, Burkina Faso, Cameroun, Congo, Côte d'Ivoire, Gabon, Togo.Tel. 00229 310896, Fax 00229 310915.
  PIEX – PHARMACIE IMPORTATION EXPORTATION, 1 Av. du Cap Horn, B.P. 224 Les Ulis, 91942 Courtaboeuf Cedex, France, Distribution au: Benin, Burkina Faso, Cameroun, Congo, Côte d'Ivoire, Gabon, Togo. Tel. 0033 1 64462343 , Fax 0033 1 69299922
- Soudan: HOUSE OF MEDICINE & INVESTMENT, P. O. Box 11201, Khartoum, E-mail: HMI@sudanmail.net, Tel. 00249 11 772537, Tel. 00249 11 782838, Fax 00249 11 784686
- Kenya: PHILLIPS PHARMACEUTICALS LTD, Power Technics Complex, Near Sahara City, P.O. Box 46662, Nairobi - Mombasa Road, Tel. 00254 2 82 36 60, Tel. 00254 2 82 43 24, Tel. 00254 2 82 36 60, Fax00254 2 82 43 66
- South Africa: MENARINI SUDAFRICA, c/o ADCOCK INGRAM GROUP, Adcock Ingram Park, 17 Harrison Av., Bryanston 2021, REP. OF SOUTH AFRICA, E-mail: restan@adcock.co.za, Tel. 0027 117099300, Tel. 0027 119211511, Tel. 0027 117099320, Fax 0027 117099332
- Italie: A. MENARINI IND. FARM. RIUNITE S.r.l., Via Sette Santi, 1, 50131 Florence , Tel. 0039 055 56801, Fax 0039 055 582771
- Autriche: A. MENARINI PHARMA GmbH, Hietzinger Kai 169, Top 102, A-1130 Wien, E-mail: knikitsch@menarini-pharma.at, Tel. +43-1-879 95 85-0, Fax +43-1-879 95 85-50
- Belgique: MENARINI BENELUX SA/NV, Belgicastraat 4, 1930 Zaventem, Tel. 0032 2 7214545 – 7209540, Fax 0032 2 7209292
- France: MENARINI FRANCE S.A., 1-7 Rue du Jura 528SILIC , 94633 Rungis Cedex, Tel. 0033 1 45607720, Fax 0033 1 46879431
- Allemagne: BERLIN-CHEMIE AG, Glienicker Weg 125-127, 12489 Berlin, Tel. 0049 30 67070 , Fax 0049 30 67072600
- Pays-bas: MENARINI BENELUX, Divisie Farma Nederland, De Bleek 173447-GV-Woerden, E-mail: menarini.nl@wxs.nl, Tel. 0031 348 432360, Fax 0031 348 432962
- Espagne: LABORATORIOS MENARINI S.A., Alfonso XII 587, (Apartado 215), 08918 Badalona, Barcelona, Tel. 0034 93 4628800, Fax 0034 93 4628820
- Suisse: A. MENARINI AG, Eggbühlstrasse 14, Postfach - 8052 Zürich, Tel. 0041 1 3074050 , Tel. 0041 1 3074051, Fax 0041 1 3074054
- Royaume-Uni: A. MENARINI Pharmaceuticals U.K. Ltd, Menarini House, Mercury Park, Wycombe Lane, Wooburn Green, Buckinghamshire, HP10 OHH, E-mail: Inquiries@Menariniuk.com, Tel. 0044 1628 856400, Fax 0044 1628 856402
- **Pour distributeurs dans d'autres parties du monde: http://www.menarini.com/english/mondo/mercati.htm**

## 8. Historique du document

| **Révision** | |
|---|---|
| | Version initiale |

| **Nom et fonction** | **Date** | **Signature** |
|---|---|---|
| Auteur | | |
| Veerle Lejon | 15/May/2012 | 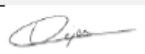 |
| Révision par | | |
| Philippe Gillet | 23/May/2012 | 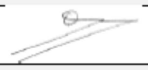 |
| Approuvé par | | |
| Emilie Alirol | 12 June 2012 | 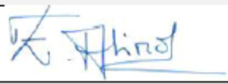 |

| | |
|---|---|
| Better Diagnosis of Neglected Infectious Diseases | **SOP titre :** Détermination de l'hémoglobine méthode HemoCue Hb 301 |
| | **Projet/Étude :** NIDIAG : Evaluation de tests rapides en association avec des prédicteurs cliniques et de laboratoire pour le diagnostic de maladies tropicales négligées dans des patients se présentant avec des troubles neurologiques dans les hôpitaux ruraux au Bandundu, République Démocratique du Congo. |

# 1. Domaine et application

Ce document fournit les instructions pour assurer la sécurité et la précision de la mesure de l'hémoglobine en utilisant le système HemoCue 301. Dans cet appareil, l'absorbance du sang total est mesurée au point isobestique Hb/$HbO_2$. sur 506 nm et aussi sur 880 nm pour compenser la turbidité de l'échantillon. L'absorbance, corrigée pour la turbidité est proportionnelle à la quantité d'hémoglobine présente dans le sang.

# 2. Responsabilités

| Fonction | Activités |
|---|---|
| Technicien de laboratoire | ▪ Suit la procédure<br>▪ Enregistre toutes les données dans le cahier de laboratoire<br>▪ Suit les règles d'assurance de qualité et tient un registre de tous les contrôles de qualité<br>▪ Suit les procédures d'entretien |

# 3. Procédures

## 3.1 Précautions

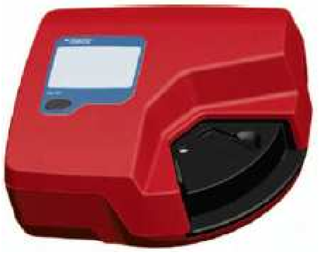

Tous les échantillons de sang sont potentiellement infectieux.
METTEZ DES GANTS pendant toute la procédure.

## 3.2 Matériel et échantillons

### 3.2.1 Matériel fourni et conservation

- Analyseur HemoCue Hb 301 (IMP code 5400049):

*Stockage* : Entre 0 – 50°C. La température de fonctionnement normale est 10 - 40 °C, à une humidité relative 5 - 90% sans condensation.

- Adaptateur secteur
- Microcuvettes HemoCue Hb 301 (IMP code 5400041) :

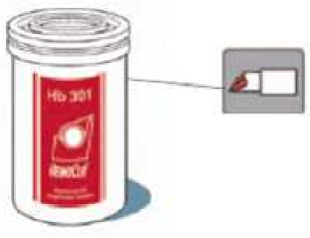


*Conservation* : À température ambiante (15 - 40°C)
NE PAS METTRE AU REFRIGERATEUR !

*Stabilité* : Utilisez avant la date de péremption. Refermez-le flacon. Après ouverture du flacon, les microcuvettes sont stables pendant 3 mois. Écrivez la date d'ouverture sur le flacon.

### 3.2.2 Matériel supplémentaire requis

- Gants non-stériles, à usage unique
- piles de type AA (1,5 V)
- Pipette Pasteur
- Lames porte-objet en verre

- Solutions de contrôle (Eurotrol Hb 301 control Low (IMP code 5400039) /Normal (IMP code 5400040 /High)
- Tampons nettoyant HemoCue (IMP code 5400019)

### 3.2.3 Echantillon à examiner

- Sang anti-coagulé veineux (EDTA) : 10 µl
- Conservation de l'échantillon : L'hémoglobine est stable pendant plusieurs jours si le prélèvement était stérile. Après conservation au réfrigérateur, laisser revenir le sang à température ambiante avant analyse.
- Critères de rejet : échantillons hémolysés ou coagulés, sang recueilli sur citrate de sodium ou dans un tube contenant un gel séparateur, échantillons contaminés.

## 3.3 Mode opératoire

### 3.3.1 Contrôle de qualité

- Un autocontrôle électronique interne, qui vérifie l'unité optique, est effectué chaque fois que l'analyseur est allumé et régulièrement pendant qu' il reste allumé.
- Faites un contrôle de qualité chaque semaine avec les solutions de contrôle recommandées:

Eurotrol Hb 301 control Low/Normal/High. Suivez « 3.3.2 Procédure » pour faire le contrôle de qualité.

### 3.3.2 Procédure

Vérifiez la date de péremption des microcuvettes et la date d'ouverture du flacon. Jetez les microcuvettes quand elles sont expirées. Prenez un nouveau lot de microcuvettes pour faire les mesures.

1. Allumez l'analyseur: Appuyez sur la touche gauche jusqu'à ce que l'écran s'allume.
2. L'unité optique est contrôlée automatiquement.
3. L'écran montre trois tirets clignotants: l'analyseur est prêt à l'emploi.
4. Tirez le support de cuvette.
5. Le résultat le plus récent est affiché sur l'écran.
6. Mélangez bien le sang veineux sur EDTA : retournez le tube doucement 8 -10 fois.
7. Placez une goutte de sang sur une lame porte-objet en verre avec une pipette
8. Remplissez la microcuvette complètement d'un seul trait.
9. Le volume exact est aspiré dans la microcuvette (10 µl). NE REMPLISSEZ JAMAIS la même microcuvette une seconde fois!!

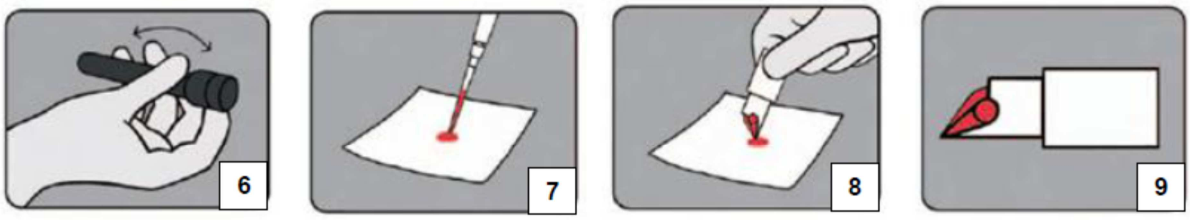


10. Essuyez le surplus de sang à l'extérieur de la microcuvette avec un tissu.
11. Vérifiez que la microcuvette remplie ne contient pas de bulles d'air. S'il y a des bulles d'air, recommencez la procédure avec une nouvelle microcuvette.
12. Mettez la microcuvette remplie dans son support (au maximum 40 secondes après le remplissage!)
13. Poussez le support de cuvette en position de mesure.
14. Après 10 secondes, le taux d'hémoglobine s'affiche.

15. Lisez et rapportez le résultat. Enlevez et jetez la microcuvette dans un conteneur approprié. Poussez le support de cuvette de retour dans l’analyseur.

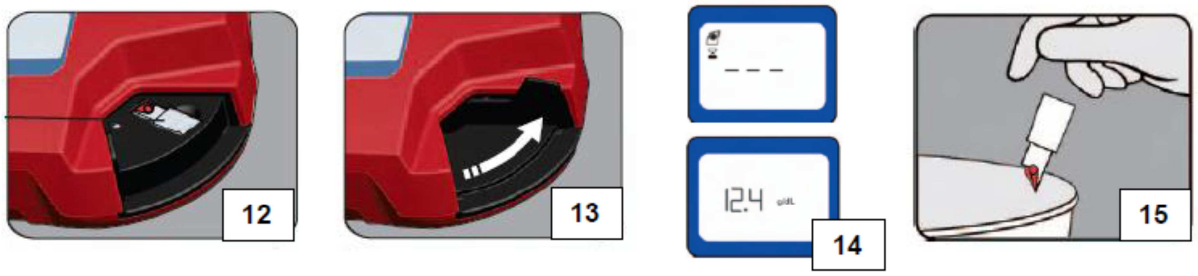


Remarques :

- Le numéro et les identifiants du patient ne peuvent pas être introduits dans l’analyseur.
- Les résultats ne sont pas mémorisés dans l’analyseur.

### 3.4 Interprétation des résultats

- Les résultats sont affichés en g/dl ou en g/l
- Plage de mesure: 0 – 25,6 g/dl
- Conversion en unités SI: g / dl x 0,6206 = mmol/l
- Les valeurs de référence d’hémoglobine selon des sources différentes:

  (Les valeurs varient en fonction de l’altitude/de la région. Vérifiez donc les données locales.)
  o **Adulte masculin:** 14 - 18 g/dl
  o **Adulte féminin:** 12 - 16 g/dl
  o **Nourrisson:** 17 - 23 g/dl
  o **Enfant:** 11 - 14 g/dl

### 3.5 Entretien

1. Vérifiez que l’analyseur est éteint.
2. Nettoyez le support de cuvette chaque jour avec de l’alcool ou un détergent doux (consultez le manuel d’utilisation).
3. Nettoyez l’unité optique avec un tampon nettoyant HemoCue une fois par mois, après 50 mesures ou quand un message d’erreur est affiché
(consultez le manuel d’utilisation).
4. Attendez 15 minutes avant de remplacer le support de cuvette.
5. Nettoyez la coque avec l’alcool ou un détergent doux.

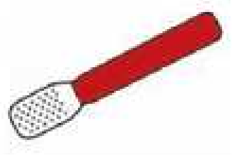

### 3.6 Causes d’erreur

- Remplissage insuffisant ou non uniforme de la microcuvette (Rouleaux, agglutination). Recommencez la procédure avec une nouvelle microcuvette
- Bulles d’air dans la microcuvette.
- Homogénéisation incorrect du sang.
- Consultez le guide de dépannage dans le manuel d’utilisation.

## 4. Définitions & abréviations

- EDTA : l'acide éthylène diamine tétra-acétique
- Hb : Hémoglobine

## 5. Références

- Manuel d’utilisation de HemoCue 301
- www.hemocue.com

- Morris LD, et al. Evaluation of the utility of the HemoCue 301 haemoglobinometer for blood donor screening. Vox Sanguinis 2007;93:64-69.


## 6. Enregistrements et archives

| **Appendices & formulaires à compléter** | |
|---|---|
| **Numéro** | **Titre** |
| 1 | Cahier de laboratoire |

## 7. Histoire de document

| **Révision** | |
|---|---|
| SOP-WP2-LAB-23-V01-18Jun2012 | Version Française de SOP-WP2-LAB-23-V01-18Jun2012 |
| SOP-WP2-LAB-23-V02.1-19Jun2012 | Fréquence Contrôle de qualité changé à chaque semaine |

| **Nom et fonction** | **Date** | **Signature** |
|---|---|---|
| *Auteur* | | |
| Barbara Barbé | 22/05/2012 | |
| *Révisé par* | | |
| Philippe Gillet | 16/09/2012 | |
| *Approuvé par* | | |
| Veerle Lejon | 18/Sep/2012 | |

| | |
|---|---|
| | **SOP titre :** Numération et différenciation des globules blancs Système HemoCue WBC DIFF |
| | **Projet/Étude :** NIDIAG : Evaluation de tests rapides en association avec des prédicteurs cliniques et de laboratoire pour le diagnostic de maladies tropicales négligées dans des patients se présentant avec des troubles neurologiques dans les hôpitaux ruraux au Bandundu, République Démocratique du Congo. |

## 1. Domaine et application

Ce document fournit les instructions pour assurer la sécurité et la précision de la numération et différentiation des globules blancs en utilisant le système HemoCue WBC DIFF. Les globules rouges sont lysés et les noyaux des globules blancs sont colorés. 37 images des noyaux colorés sont prises et analysés pour identifier et compter les globules blancs.

## 2. Responsabilités

| **Fonction** | **Activités** |
|---|---|
| Technicien de laboratoire | ▪ Suit la procédure<br>▪ Enregistre toutes les données dans le cahier de laboratoire<br>▪ Suit les règles d'assurance de qualité et tient un registre de tous les contrôles de qualité<br>▪ Suit les procédures d'entretien |

## 3. Procédures

### 3.1 Précautions

Tous les échantillons de sang sont potentiellement infectieux.
METTEZ DES GANTS pendant toute la procédure.

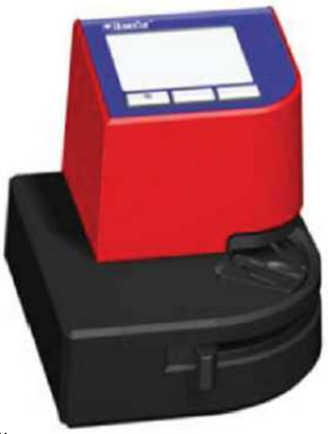

### 3.2 Matériel et échantillons

#### 3.2.1 Matériel fourni et conservation

- Analyseur HemoCue WBC DIFF :

*Stockage* : Entre 4 – 50°C. La température de fonctionnement normal est 15 - 35 °C, à une humidité < 90% sans condensation.

- Adaptateur secteur
- Microcuvettes HemoCue WBC DIFF :

*Conservation* : À température ambiante (15 - 35°C), humidité relative < 90%
NE PAS METTRE AU REFRIGERATEUR !

*Stabilité* : Utilisez avant la date de péremption.
Utilisez la microcuvette dans les 10 minutes qui suivent l'ouverture d'un emballage unique.

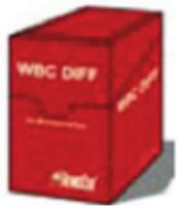


#### 3.2.2 Matériel supplémentaire requis

- Gants non-stériles, à usage unique
- 6 piles de type C (1,5 V)
- Pipette Pasteur
- Lames porte-objet en verre
- Tampons nettoyant WBC DIFF
- Solutions de contrôle (R&D Systems HemoCue WBC control solutions, en projet)

### 3.2.3 Echantillon à examiner

- Sang anti-coagulé veineux (EDTA) : 10 µl
- Conservation de l'échantillon : Conservez les échantillons à température ambiante (15 - 35°C). Analysez les échantillons au maximum 8 heures après le prélèvement de sang.
- Critères de rejet : échantillons coagulés ou contaminés, présence de cryo-globulines.

## 3.3 Mode opératoire

### 3.3.1 Contrôle de qualité

Un autocontrôle électronique interne, qui vérifie l'unité optique, est effectué chaque fois que l'analyseur est allumé et avant chaque mesure. Des que disponible, les solutions de contrôles devront être analysées journellement.

### 3.3.2 Procédure

Vérifiez la date de péremption des microcuvettes et la date d'ouverture du flacon. Jetez les microcuvettes si elles sont expirées ou si le flacon est depuis plus de 4 semaines et prenez un nouveau lot de microcuvettes pour faire les mesures.

1. Mettez le bras mobile de la cuvette en position de charge.
2. Allumez l'analyseur: Appuyez sur la touche de gauche jusqu'à ce que l'écran s'allume.
3. L'analyseur effectue un autotest : un sablier s'affiche sur l'écran.
4. Le menu principal s'affiche.
5. L'analyseur est prêt à l'emploi.

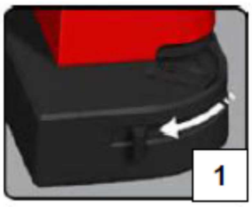

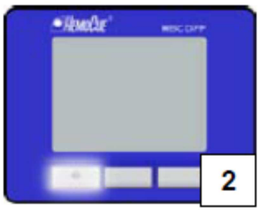

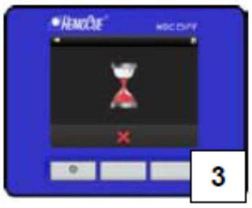

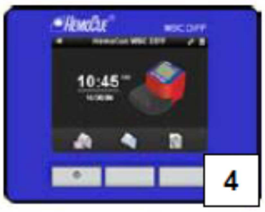


6. Appuyez sur la deuxième touche en dessous du symbole « Test patient » et utilisez le clavier pour saisir les données du patient.
7. Appuyer la touche droite pour confirmer les données saisies.
Appuyer la touche gauche pour changer les données saisies.
8. Le symbole « Insérer cuvette » s'affiche.

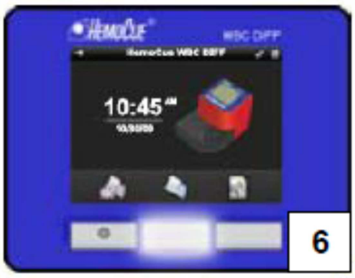

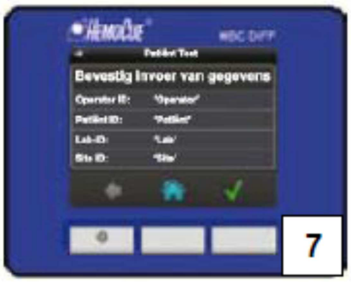

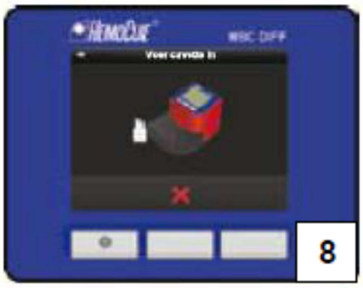


9. Mélangez le sang veineux prélevé sur EDTA : retournez doucement le tube 10 à 20 fois.
10. Placez une goutte de sang sur une lame porte-objet avec une pipette.
11. Remplissez la microcuvette complètement en une seule fois.
12. Le volume exact est aspiré dans la microcuvette (10 µl). NE REMPLISSEZ JAMAIS la même microcuvette une seconde fois!!

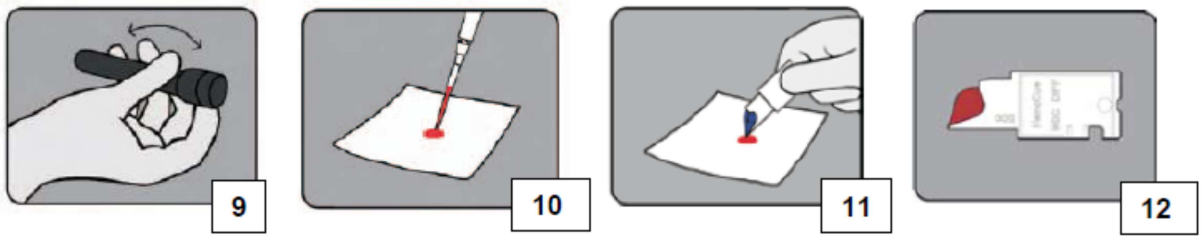


13. Essuyez le surplus de sang à l'extérieur de la microcuvette avec un tissu.
14. Vérifiez que la microcuvette remplie ne contient pas de bulles d'air. S'il y a des bulles d'air, recommencez la procédure avec une nouvelle microcuvette.
15. Placez la microcuvette remplie dans le support de la cuvette (au **maximum 40 secondes** après le remplissage!) et poussez le bras mobile en position de mesure.
16. La fenêtre « Mesure » s'affiche.
17. Le résultat s'affiche après 5 minutes :
    a. La fenêtre des résultats WBC DIFF
    b. La fenêtre des données saisies

Appuyez sur la deuxième touche pour basculer entre les 2 fenêtres.
18. Rapportez le résultat. Appuyez sur la première ou la troisième touche pour confirmer ou refuser le résultat. Le résultat est enregistré. NB : Tous les résultats sont enregistrés dans la mémoire (jusqu'à 600 résultats). Les résultats rejetés sont aussi enregistrés, mais marqués par un drapeau.
19. L'écran affiche le menu principal.
20. Enlevez et jetez la microcuvette dans un conteneur approprié. Repoussez le bras mobile de la cuvette dans l'analyseur.

<u>Remarque</u> : NE REMESUREZ PAS la microcuvette remplie.

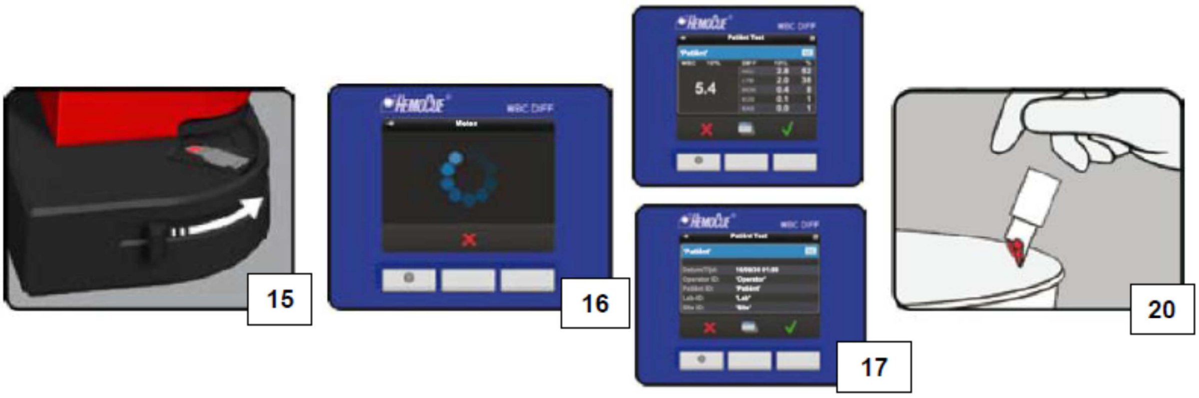


## 3.4 Interprétation des résultats

- Les résultats sont affichés en 10$_9$/L et %
- Plage de mesure: 0.3 à 30 x10$_9$/L. Les résultats supérieurs sont rapportés come « HHH », les résultats inférieurs comme « LLL ».
- La formule leucocytaire est affichée quand la numération des globules blancs est dans la plage de 1 à 30 x10$_9$/L
- Les valeurs de référence sont :

(Les valeurs varient en fonction du genre, stress, tabagisme, conditionnement physique, indigestion, variation quotidienne)

**Enfants (moyenne ± 2SD)**

| | 3-6 mois | 1 an | 2-6 ans | 6-12 ans |
|---|---|---|---|---|
| WBC (x $10^9$/L) | 12 ± 6 | 11 ± 5 | 10 ± 5 | 9 ± 4 |
| Neutrophiles (x $10^9$/L) | 1 - 6 | 1 - 7 | 1.5 - 8 | 2 - 8 |
| Lymphocytes (x $10^9$/L) | 4 - 12 | 3.5 - 11 | 6 - 9 | 1 - 5 |
| Monocytes (x $10^9$/L) | 0.2 - 1.2 | 0.2 - 1.0 | 0.2 - 1.0 | 0.2 - 1.0 |
| Eosinophiles (x $10^9$/L) | 0.1 - 1.0 | 0.1 - 1.0 | 0.1 - 1.0 | 0.1 - 1.0 |

**Adultes (moyenne ± 2SD)**

| | Adulte | Adulte % |
|---|---|---|
| WBC (x $10^9$/L) | 4.0 - 10.0 | |
| Neutrophiles (x $10^9$/L) | 2.0 - 7.0 | 40 - 80 |
| Lymphocytes (x $10^9$/L) | 1.0 - 3.0 | 20 - 40 |
| Monocytes (x $10^9$/L) | 0.2 - 1.0 | 2 - 10 |
| Eosinophiles (x $10^9$/L) | 0.02 - 0.5 | 1 - 6 |
| Basophiles (x $10^9$/L) | 0.02 - 0.1 | <1 - 2 |

### 3.5 Entretien

1. Vérifiez que l'analyseur est éteint.
2. Nettoyez le support de cuvette chaque jour avec de l'alcool ou un détergent doux (consultez le manuel d'utilisation).
3. Nettoyez l'unité optique avec un tampon nettoyant HemoCue une fois par mois. (consultez le manuel d'utilisation).
4. Attendez 15 minutes avant de remplacer le support de cuvette.
5. Nettoyez la coque avec l'alcool ou un détergent doux.

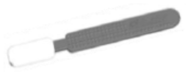

### 3.6 Causes d'erreur

- Remplissage insuffisant ou non uniforme de la microcuvette (Rouleaux, agglutination). Recommencez la procédure avec une nouvelle microcuvette
- Bulles d'air dans la microcuvette.
- Homogénéisation incorrect du sang.
- Consultez le guide de dépannage dans le manuel d'utilisation.

## 4. Définitions & abréviations

- DIFF : differential (différentiel)
- EDTA : acide éthylène diamine tétra-acétique
- WBC : white blood cell (globules blancs)

## 5. Références

- Osei-Bimpong A, et al. Point-of-care method for total white cell count: an evaluation of the HemoCue WBC device. Int Jnl Lab Hem 2009;31:657-664.

- De Jonge R. Evaluation of the HemoCue WBC analyzer to count leucocytes in body fluids. Ned Tijdschr Klin Chem Labgeneesk 2011;36:35-36.

HemoCue WBC DIFF User Manual

## 6. Enregistrements et archives

| **Appendices & formulaires à compléter** | |
|---|---|
| **Numéro** | **Titre** |
| 1 | Cahier de laboratoire |

## 7. Historique du document

| **Révision** | |
|---|---|
| SOP-WP2-LAB-24-V01-18Jun-2012 | Version Française de SOP-WP2-LAB-24-V01-18Jun-2012 |

| **Nom et fonction** | **Date** | **Signature** |
|---|---|---|
| *Auteur* | | |
| Barbara Barbé | 22/05/2012 | |
| *Révisé par* | | |
| Philippe Gillet | 24/05/2012 | |
| *Approuvé par* | | |
| Veerle Lejon | 18 Juin 2012 | |

| | |
|---|---|
| | **SOP titre :** SD Syphilis 3.0 - SD Bioline |
| | **Projet/Étude** : NIDIAG : Evaluation de tests rapides en association avec des prédicteurs cliniques et de laboratoire pour le diagnostic de maladies tropicales négligées dans des patients se présentant avec des troubles neurologiques dans les hôpitaux ruraux au Bandundu, République Démocratique du Congo. |

# 1. Domaine et application

Ce document fournit les instructions pour l'utilisation du test rapide SD Syphilis 3.0 qui indique la présence d'anticorps contre *Treponema pallidum* (de tous isotypes : IgM, IgG ou IgA). Ce test apporte une aide pour le diagnostic de la syphilis. Attention : Ce test ne différencie pas une infection actuelle d'une infection antérieure.

# 2. Responsabilités

| Fonction | Activités |
|---|---|
| Technicien de laboratoire | ▪ Prélève le sang<br>▪ Exécute le test<br>▪ Interprète le résultat<br>▪ Enregistre les résultats |

# 3. Procédures

## 3.1 Précautions

- Tous les échantillons de sang sont potentiellement infectieux. Respectez les précautions universelles. METTEZ DES GANTS À USAGE UNIQUE PENDANT TOUTE LA PROCÉDURE **!**
- Le diluent contient de l'azide de sodium (0,02% p/v), qui est irritant pour la peau. EVITEZ TOUT CONTACT AVEC LA PEAU !
- Les réactifs contiennent de l'azide de sodium (0.02% p/v). NE JETEZ PAS LES REACTIFS DANS UN EVIER: le contact de l'azide avec du plomb ou du cuivre forme un produit explosif !

## 3.2 Matériel et échantillons

### 3.2.2 Matériel fourni dans le kit et conservation

- Cassette SD BIOLINE Syphilis 3.0 :
  Une bandelette de test comprenant :
  - Le conjugué : antigène recombinant de *Treponema pallidum* – or colloïdal
  - Ligne de test : antigène recombinant de *Treponama pallidum*
  - Ligne du contrôle : anticorps de chèvre anti-*Treponema pallidum*

  *Conservation* : à température ambiante **NE PAS CONGELER !.** La cassette est sensible à l'humidité et la chaleur. Utilisez les tests immédiatement après l'ouverture du sachet. N'UTILISEZ PAS le test si le sachet est endommagé.
- Diluant (tampon):
  Une bouteille contient :
  - 50 mM tampon Tris-HCl buffer
  - Azide de sodium (0,02% w/w)

  *Conservation* : à température ambiante .
  Remarque : Utilisez uniquement le tampon inclus pour tous les tests appartenant au même kit.
- Brochure explicative

### 3.2.2 Matériel supplémentaire requis

- Gants, non stérile, à usage unique
- Marqueur
- Micropipettes 20 µl
- Embouts pour micropipette
- Minuterie
- Conteneur « bio-hazard »

### 3.4.3 Echantillon à examiner

- Sang total sur EDTA (20 µl).
- *Conservation*:
    - *Sang total sur EDTA* : Exécutez le test immédiatement, si ce n'est pas possible, réfrigérez l'échantillon à 2-8°C pour 3 jours maximum, ou congelez à -20°C pour des périodes plus longues.
    - *Sérum/plasma* : Exécutez le test immédiatement, réfrigérez l'échantillon à 2-8°C pendant 2 semaines, ou congelez à -20°C pour des périodes plus longues.

Remarque : Laissez revenir les échantillons à température ambiante avant d'exécuter le test. Assurez-vous que le sérum/plasma ne contient pas de précipité. S'il y en a, centrifugez l'échantillon avant de l'utiliser.

## 3.5 Mode opératoire

### 3.5.1 Contrôle de qualité interne

Une ligne de contrôle « C » est inclus dans le système afin d'assurer la validité du test. Si la ligne de contrôle n'apparait pas à la fin du test, le résultat n'est pas valide.

### 3.5.2 Procédure

12. Mettez des gants à usage unique.
13. Vérifiez la date de péremption au dos du sachet. N'utilisez jamais les tests après la date de péremption.
14. Enlevez la cassette du sachet.
15. Vérifiez la couleur du silicagel. En cas de changement de couleur (bleu qui est devenu rose), prenez un nouveau test !
16. Identifiez la cassette avec le numéro du patient et notez la date.
17. Déposez 20 µl de sang total dans le puits « S ».
18. Ajoutez 4 gouttes diluant (~120 µl) dans le puits « S ».
    Prenez soin de ne pas toucher le puits, pour éviter de contaminer la pointe de la bouteille.
19. Lancez la minuterie (10 minutes).
20. Une couleur pourpre commence à se déplacer le long de la fenêtre de résultat. Lisez le test après 5 – 20 minutes.
21. Prenez une photo du résultat.
22. Notez dans le CRF laboratoire les résultats du test, y compris l'intensité de la bande du test, le N° de lot du Kit et le N° de la photo

**Sang total (20 µl)**

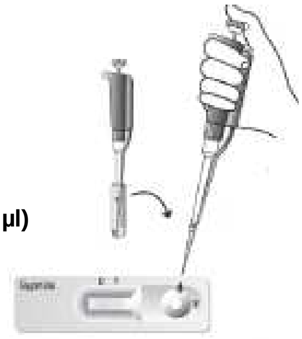

**4 gouttes**

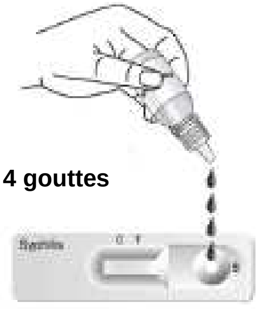

**5 – 20 minutes**

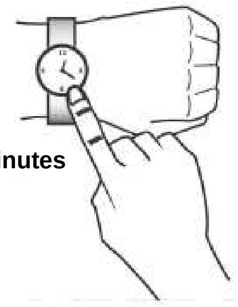

### 3.6 Interprétation du test

**N'INTERPRÉTEZ PAS** les résultats après 20 minutes !

- Pas de bande pourpre de contrôle : **Invalide**
  L'absence d'une bande pourpre sous le « C » indique que le test s'est détérioré ou que la procédure n'est pas correctement suivie. RÉPÉTEZ LE TEST !

- 1 bande pourpre : **Négatif**
  Une seule bande pourpre dans la fenêtre de résultat, en dessous de « C »

- 2 bandes pourpre : **Positif**
  Deux bandes pourpres dans la fenêtre de résultat : 1 sous le « C » et 1 sous le « T »

**Négatif** **Positif** **Invalide**

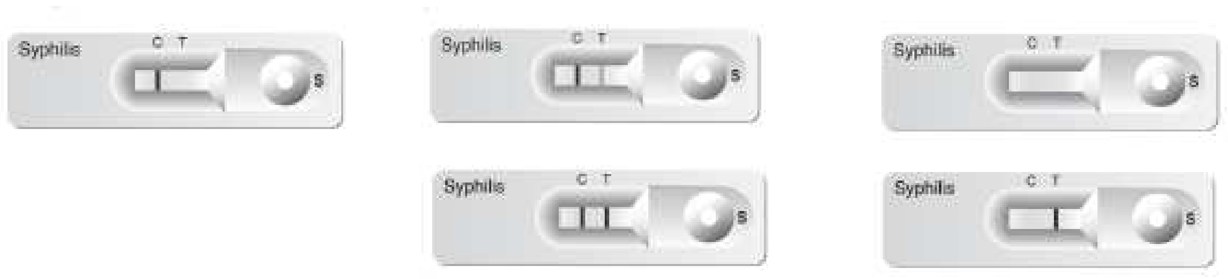


### 3.7 Déchets, nettoyage

- Jetez les cassettes utilisées dans un conteneur « bio-hazard ».
- Jetez l'excès du diluant (après avoir utilisés tous les tests du kit) dans un conteneur « bio-hazard ».

## 4. Enregistrements et archives

| **Appendices & formulaires à completer** | |
|---|---|
| **Numéro** | **Titre** |
| 1 | CRF "laboratoire" |

## 5. Références

- Instruction du producteur, version 06FK10-FR1 2009.02

## 6. Histoire de document

| **Révision** | |
|---|---|
| | Version initiale |

| **Nom et fonction** | **Date** | **Signature** |
|---|---|---|
| *Auteur* | | |
| Barbara Barbé | 29/05/2012 | |
| *Révisé par* | | |
| Philippe Gillet | 07/06/2012 | |
| *Approuvé par* | | |
| Veerle Lejon | 12/07/2012 | |

| | |
|---|---|
| | **SOP titre :** Encre de Chine – Recherche de Cryptocoques dans le LCR |
| | **Projet/Étude :** NIDIAG : Evaluation de tests rapides en association avec des prédicteurs cliniques et de laboratoire pour le diagnostic de maladies tropicales négligées dans des patients se présentant avec des troubles neurologiques dans les hôpitaux ruraux au Bandundu, République Démocratique du Congo. |

# 1. Domaine et application

Ce document fournit les instructions pour l'exécution du coloration du LCR à l'encre de Chine pour détecter des Cryptocoques dans le liquide céphalo-rachidien (LCR). *Cryptococcus neoformans* est un champignon entouré d'une grande capsule non-colorable à l'encre de chine, permettant ainsi sa détection au microscope à l'objectif 40x.

# 2. Responsabilités

| Fonction | Activités |
|---|---|
| Technicien de laboratoire | ▪ Prélève le LCR<br>▪ Exécute le test<br>▪ Interprète le résultat<br>▪ Enregistre les résultats |

# 3. Procédures

## 3.1 Précautions

Tous les échantillons de LCR sont potentiellement infectieux. Respectez les précautions universelles. METTEZ DES GANTS À USAGE UNIQUE PENDANT TOUTE LA PROCÉDURE **!**

## 3.2 Matériel et échantillons

### 3.2.3 Matériel

- Gants, non-stérile, à usage unique
- Encre de Chine
- Lame porte-objet
- Lamelle couvre-objet
- Pipette pasteur en plastique
- Anse de platine
- Lampe à alcool (ou butagaz)
- Microscope, objectif 40x
- Conteneur « bio-hazard ».

### 3.2.2. Echantillon à examiner

LCR frais : une goutte du culot du LCR centrifugé (15 minutes à 3.500 rpm) (UNIQUEMENT pour les patients **VIH positifs**, avec **plus de 5 globules blancs/µl** de LCR)

## 3.3 Mode opératoire

1. Mettez des gants à usage unique.
2. Identifiez une lame porte-objet avec le numéro du patient.
3. Déposez une goutte de l'encre de Chine sur la lame porte-objet avec une pipette pasteur.
4. Flambez l'anse de platine (portez le fil au rouge sur toute sa longueur) puis laissez-la refroidir.
5. Mélangez avec l'anse de platine le culot de centrifugation du LCR

6. Prélevez avec l'anse de platine un goutte du culot du LCR et mélangez-la avec l'encre de Chine sur la lame porte-objet.
7. Flambez l'anse platine (portez le fil au rouge sur toute sa longueur) pour détruire les germes.
8. Recouvrez la lame d'une lamelle couvre-objet.
9. Observez au microscope (objectif 40x, oculaire 10 x)
10. Le champignon se présente sous forme de spores rondes, bourgeonnantes, contenant des granulations grisâtres. Les spores mesurent entre 2 et 10 µm et sont entourées d'une grande capsule non-colorée, qui est visible comme un halo clair autour du champignon. Ils peuvent avoir des tailles différentes. (Figure 1)

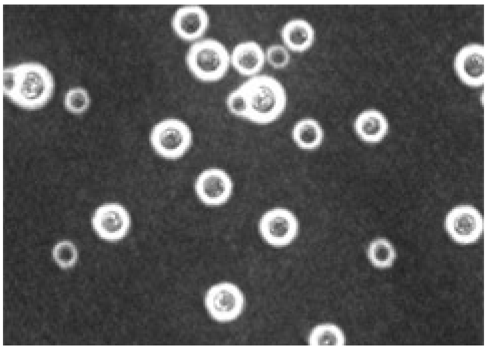

Figure 1. Preparation de LCR avec de l'encre de chine**,** *C. neoformans* , 40x. (Notez la grande capsule autour des champignons)

### 3.4 Rapportage des résultats

Rapportez comme :

- Présence de levures entourées d'une capsule.
- Recherche négative

### 3.5 Déchets, nettoyage

Jetez les lames utilisées dans un conteneur « bio-hazard ».

## 4. Enregistrements et archives

| **Appendices & formulaires à compléter** | |
|---|---|
| **Numéro** | **Titre** |
| 1 | CRF laboratoire |

## 5. Histoire de document

| **Révision** | |
|---|---|
| | Version initiale |

| **Nom et fonction** | **Date** | **Signature** |
|---|---|---|
| *Auteur* | | |
| Hilde De Boeck<br>Barbara Barbé | 31/05/2012 | |
| *Révisé par* | | |
| Philippe Gillet | 12/06/2012 | |
| *Approuvé par* | | |
| Veerle Lejon | 12/07/2012 | |

| | |
|---|---|
| | **SOP titre :** Coloration de Gram |
| | **Projet/Étude :** NIDIAG : Evaluation de tests rapides en association avec des prédicteurs cliniques et de laboratoire pour le diagnostic de maladies tropicales négligées dans des patients se présentant avec des troubles neurologiques dans les hôpitaux ruraux au Bandundu, République Démocratique du Congo. |

# 1. Domaine et application

*Ce document donne les instructions pour effectuer une coloration de Gram sur un frottis de LCR (liquide céphalo-rachidien). Cette coloration différentielle permet de classer les bactéries en deux familles sur base de la perméabilité de leur paroi à l'alcool (et donc de leur sensibilité à une décoloration par l'alcool) : les bactéries Gram-positives et les Gram-négatives.*
*La perméabilité de la paroi dépend de sa composition : les parois constituées d'une couche épaisse de peptidoglycane sont imperméable à l'alcool et ne peuvent pas être décolorées (Gram-positives). Les parois constituées d'une couche mince de peptidoglycane sont perméables à l'alcool et peuvent être décolorées (Gram-négatives).*

# 2. Responsabilités

| Fonction | Activités |
|---|---|
| *Technicien de laboratoire* | ▪ *Prélève le LCR*<br>▪ *Pré-traite l'échantillon*<br>▪ *Exécute le test*<br>▪ *Interprète le test*<br>▪ *Enregistre les résultats* |

# 3. Procédures

## 3.1 Précautions

- Tous les échantillons de LCR sont potentiellement infectieux. Respectez les précautions universelles.
  METTEZ DES GANTS À USAGE UNIQUE PENDANT TOUTE LA PROCÉDURE **!**
  METTEZ UN MASQUE RESPIRATOIRE FFP3 PENDANT TOUTE LA PROCÉDURE **!**
- Tous les alcools sont inflammables, manipulez ces produits loin d'une flamme.

## 3.2 Matériel et échantillons

### 3.2.4 Matériel requis

- Gants, non stérile, à usage unique
- Lame porte-objet neuve
- Crayon diamant
- Anse de platine
- Flacon contenant un mélange de sable et de désinfectant.
- Lampe à alcool
- Méthanol
- Pipettes pasteur
- Cristal violet
- Lugol faible pour Gram
- Alcool à 96°
- Safranine
- Support de coloration des lames
- Support sèche lame

- Microscope (objectif 100 x)
- Huile à immersion
- Minuterie

### 3.2.5 Echantillon à examiner

LCR prélevé depuis moins d'une heure : une goutte du culot de centrifugation du LCR (10 minutes à 3000 rpm, soit pour la centrifugeuse AML A8 en position 8).

## 3.3 Mode opératoire

### 3.3.1 Principe de la coloration

La coloration de Gram utilise 4 réactifs :

- Le **Cristal Violet** qui colore tout en violet.
- Le **Lugol faible**, qui se complexe au cristal violet, formant un colorant violet soluble dans l'alcool.
- L'**Alcool à 96%** qui décolore des bactéries dont la paroi est perméable à l'alcool (Gram-négatives), mais pas celles dont la paroi est imperméable à l'alcool (Gram-positives).
- La **Safranine**, qui colore en rouge/rose les bactéries Gram-négatives qui sont décolorées par l'alcool, sans modifier la couleur violet foncé-bleu des bactéries Gram-positives.

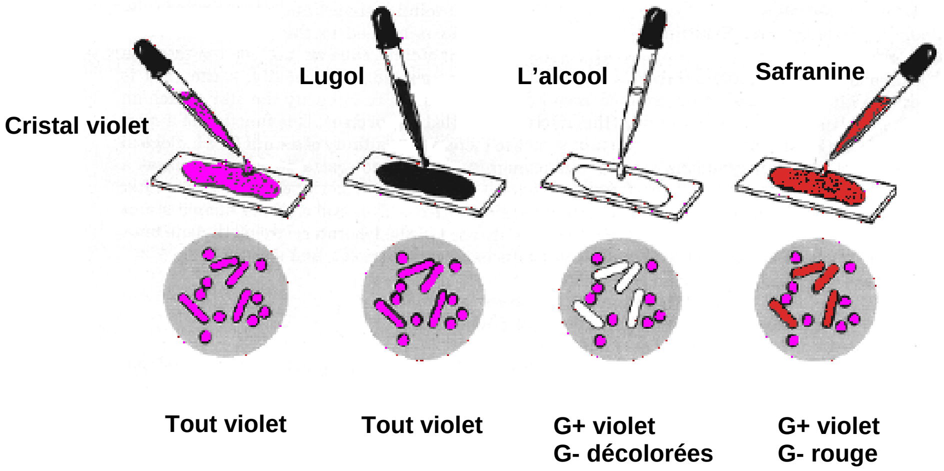


On distingue ainsi deux classes de bactéries :

- **Gram positives** (paroi épaisse de peptidoglycane) qui sont colorées en **violet foncé/bleu.**
- **Gram négatives** (paroi mince de peptidoglycane) qui sont colorées en **rouge/rose.**

### 3.3.2 Procédure

23. Notez au crayon diamant le numéro du patient sur une lame neuve.
24. Flambez la lame en la passant 3 x sur la flamme d'une lampe à alcool.
25. Décontaminez l'anse de platine en l'immergeant dans le flacon contenant un mélange de sable et de désinfectant.
26. Flambez l'anse de platine (porter le fil au rouge sur toute sa longueur) puis laissez-la refroidir.
27. Etalez à l'aide de l'anse de platine une goutte du culot de centrifugation du LCR sur la lame flambée et faites un frottis aussi mince que possible.

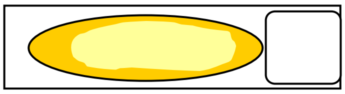

28. Décontaminez l'anse de platine en l'immergeant dans

le flacon contenant un mélange de sable et de désinfectant.

29. Reflambez l'anse de platine (porter le fil au rouge) pour détruire les bactéries.
30. Laissez sécher à l'air, à l'abri des insectes.
31. Recouvrez la lame de méthanol pur pour fixer le frottis.
32. Eliminez le méthanol et laisser sécher.
33. Déposez la lame fixée sur un support de coloration (des barres en position horizontales). Si plusieurs lames sont colorées en même temps, ne les laissez pas se toucher.
34. Couvrez complètement la lame de Cristal violet pendant 1 minute 30 secondes.
35. Rincez la lame à l'eau (propre ou filtrée).
36. Couvrez complètement la lame de Lugol faible (Lugol pour Gram) pendant 1 minute 30 secondes.
37. Rincez **très soigneusement** la lame à l'eau (propre ou filtrée).
38. Décolorez à l'alcool à 96 % :
    - 38.1 Tenez la lame entre le pouce et l'index.
    - 38.2 Laissez couler goutte à goutte de l'alcool à 96 % sur la lame tout en observant la décoloration.
    - 38.3 Arrêtez immédiatement la décoloration, dès que l'alcool n'entraîne plus de violet (2 - 30 secondes), en plongeant la lame dans un bécher contenant de l'eau (propre ou filtrée).
39. Rincez à l'eau (propre ou filtrée).
40. Couvrez complètement la lame de Safranine pendant 1 minute 30 secondes.
41. Rincez à l'eau (propre ou filtrée).
42. Séchez à l'air.
43. Observez la lame au microscope (à l'huile d'immersion, objectif 100 x, oculaire 10 x)

## 3.4 Rendu des résultats

- Recherche négative.
- Présence de :
    - Forme des bactéries : Coques, bacilles.
    - Coloration : Gram positif ou Gram négatif.
    - Disposition des bactéries : Par 2 (= diplo-), en chaînettes, en grappes.
    - Localisation des bactéries : Intra ou extracellulaires.
    - Particularités : Capsules, ...

## 3.5 Interprétation des résultats

L'élément essentiel et critique de la coloration de Gram est la décoloration par l'alcool (ou l'alcool acétone) :

- *Décoloration trop courte* → faux Gram positifs
- *Décoloration trop longue* → faux Gram négatifs

Examinez la couleur des leucocytes pour valider la qualité de la coloration de Gram :
Leurs cytoplasmes doivent être uniformément rouge et leurs noyaux doivent être partiellement bleu et rouge.

- *Décoloration trop courte* → les cytoplasmes des leucocytes restent violets.
- *Décoloration trop longue* → les noyaux des leucocytes sont rouges.

L'interprétation du caractère Gram des bactéries se fait donc dans les environs immédiats d'endroits bien colorés (déterminé sur base de la coloration des leucocytes).

Voir **Annexe 1** pour la classification des bactéries sur base de leurs morphologies et de leurs couleurs au Gram !

## 3.6 Stockage et conservation des lames pour relecture

- Enlevez l'huile à immersion en déposant délicatement la lame sur un papier doux.

- Placez les lames nettoyées dans des boîtes de rangement pour lames porte-objet et étiquetez clairement les boîtes (Nom de l'étude, type de coloration, numéro de la boîte, date de la première lame)

## 4. Enregistrements et archives

| **Appendices & formulaires à completer** | |
|---|---|
| **Numéro** | **Titre** |
| 1 | CRF laboratoire |
| Annexe 1 | Classification des bactéries sur base de leurs morphologies et de leurs couleurs au Gram |

## 5. Histoire de document

| **Révision** | |
|---|---|
| | Version initiale |

| **Nom et fonction** | **Date** | **Signature** |
|---|---|---|
| *Auteur* | | |
| Barbara Barbé | 01/06/2012 | |
| *Révisé par* | | |
| Philippe Gillet | 10/09/2012 | |
| *Approuvé par* | | |
| Veerle Lejon | 17/Sep/2012 | |

| | |
|---|---|
| | **Annexe 1 :** Classification des bactéries sur base de leurs morphologies et de leurs couleurs au Gram |
| | **Projet/Étude :** NIDIAG : Evaluation de tests rapides en association avec des prédicteurs cliniques et de laboratoire pour le diagnostic de maladies tropicales négligées dans des patients se présentant avec des troubles neurologiques dans les hôpitaux ruraux au Bandundu, République Démocratique du Congo. |

## 1. Domaine et application

*Cette annexe donne un aperçu de la classification des bactéries sur base de leurs morphologies et de leurs couleurs au Gram. Cette annexe fait partie de la procédure de la coloration de Gram sur un frottis de LCR (liquide céphalo-rachidien).*

## 2. Responsabilités

| Fonction | Activités |
|---|---|
| *Technicien de laboratoire* | ▪ *Utilise ce schéma pour rapporter les résultats dans le cahier de laboratoire* |

## 3. Classification des bactéries sur base de leurs morphologies et de leurs couleurs au Gram

| **Bactéries à GRAM POSITIF**<br>**(Violet foncé / Bleu)** | **Bactéries à GRAM NEGATIF**<br>**(Rose / Rouge)** |
|---|---|
| **Coques en grappes** | **(Diplo)Coques** |
| Arrondies,<br>Par deux ou en amas.<br><br>Exemples :<br>*Staphylococcus aureus*<br>*Staphylococcus saprophyticus*<br>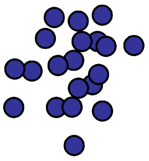 | Arrondies,<br>Par deux ou en amas.<br><br>Exemples :<br><br>[Lautropia mirabilis],<br>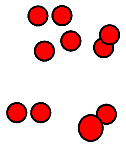 |
| **Coques en chaînes** | **Diplocoques** |
| Ovoïdes,<br>Par deux ou en chaînettes.<br>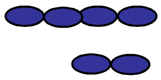<br>Exemples :<br>*Streptococcus pyogenes* (β hémolytique) [A][1]<br>*Streptococcus agalactiae* (β hémolytique) [B]<br>*Enterococcus faecalis* (entérocoques) [D]<br>…<br>Par deux, avec une capsule<br>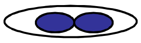<br>Exemples :<br>*Streptococcus pneumoniae* (α hémolytique) | Diplocoques,<br>Réniforme (grains de café ou haricot),<br>intra ou extra cellulaires.<br>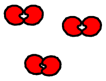<br>Exemples :<br>*Neisseria meningitidis*<br>*Neisseria gonorrhoeae*<br>*Moraxella (Branhamella) catarrhalis*<br>Autres *Neisseria*<br>… |

[1] [ ] Sérogroupes des *Streptococcus* spp., classification de Lancefield basée sur les antigènes polysaccharidiques C de leur paroi. *S. pneumoniae* n'en possède pas.

| Bactéries à GRAM POSITIF<br>(Violet foncé / Bleu) | Bactéries à GRAM NEGATIF<br>(Rose / Rouge) |
|---|---|
| **Gros bacilles** | **Gros bacilles** |
| Gros bacilles,<br>Formant des spores<br><br>**Arrondis** 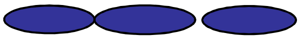<br><br>Exemples :<br>**Bacillus** (*Bacillus cereus, Bacillus subtilis,…*)<br>**Clostridium** (*Clostridium tetani, Clostridium difficile, Clostridium botulinum, Clostridium perfringens…*)<br>…<br><br>**Rectangulaires** 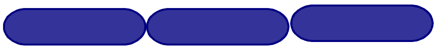<br><br>Exemples :<br>*Bacillus anthracis* | Gros bâtonnets,<br>Courts ou longs<br>Parfois bi polaires (*E. coli*) 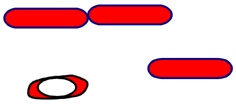<br><br>Exemples :<br>**Entérobactéries** (*E. coli, Shigella, Salmonella, Citrobacter, Klebsiella, Yersinia, Proteus,…*)<br>**Pseudomonas** (*Pseudomonas aeruginosa,…*)<br>**Borrelia**<br>… |
| **Bacilles moyens** | **Bacilles moyens** |
| Taille moyenne, 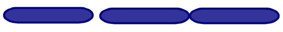<br>Réguliers,<br>Formant des chaînes.<br><br>Exemples<br>*Lactobacillus acidophilus* | Taille moyenne,<br>En forme de virgules<br>polymorphes<br>(Gram labile) 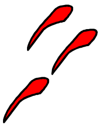<br><br>Exemples :<br>*Vibrio cholerae* |
| **Petits bacilles** | **Petits bacilles** |
| Petits, granulés, irréguliers,<br>Polymorphes, 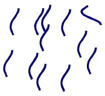<br><br>**« Corynéformes »**<br><br>Exemples<br>*Listeria monocytogenes*<br>*Gardnerella vaginalis*<br>*Bacteroïdes*<br>*Mycobacterium*<br>*Corynebacterium diphteriae*<br>… | Coccobacilles,<br>Fins, polymorphes. 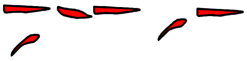<br><br>**« Haemophilus like »**<br><br>Exemples :<br>*Haemophilus influenzae,*<br>*Haemophilus ducreyi*<br>*Brucella*<br>*Bordetella*<br>… |

## GERMES POUVANT ÊTRE RETROUVES DANS UN L.C.R.

| Forme | Morphologie, Disposition et Localisation | Coloration | Dessin | Agent causal Probable |
|---|---|---|---|---|
| 1 Coques | Souvent rares, par 2, en grains de café, intra ou extra-cellulaires. | Gram Négatif | | *Neisseria meningitidis* |
| 2 Bacilles | Souvent rares, fins bâtonnets, coccoïdes, chaîne courte, intra ou extra-cellulaires, souvent entourés d'une capsule. | Gram Négatif | | *Haemophilus influenzae* |
| 3 Coques | souvent abondants, en forme de lance, par 2 ou en courtes chaînes, souvent entourés d'une capsule, toujours extra-cellulaires | Gram Positif | | *Streptococcus pneumoniae* |
| 4 Grands éléments ronds | grands éléments ronds de la taille d'un G.R., souvent avec des bourgeonnements, parfois avec un pseudomycélium [Avec capsule pour *Cryptococcus neoformans]* | Gram Positif<br><br>Encre de chine | | Levure<br><br>*Cryptococcus neoformans* |
| 5 Coques | coques réguliers en amas, extra-cellulaires | Gram Positif | | Staphylocoques (*S. aureus*, *S. epidermidis*,…) |
| 6 Bacilles | petits bâtonnets isolés ou couplés, en forme de V, parfois en palissade, intra ou extra-cellulaires | Gram Positif | | *Listeria monocytogenes* |
| 7 Coques | coques par 2 ou en chaîne assez longue | Gram Positif | | *Streptococcus agalactiae* |
| 8 Bacilles | gros bâtonnets, parfois bipolaires | Gram Négatif | | Entérobactéries<br><br>*(Escherichia coli)* |
| 9 Bacilles | fins bâtonnets | Ziehl BAAR | | *Mycobacterium tuberculosis* |

**GRAM POSITIF = VIOLET FONCE / BLEU**
**GRAM NEGATIF = ROUGE / ROSE**

Les germes 1 à 3 représentent 80 à 90 % des méningites bactériennes. Toute classe d'âge.
Les *Neisseria* sont responsable de la plupart des épidémies.
Le germe 2 se retrouve principalement chez des enfants de 3 mois à 3 ans.
Les germes 4 et 5 ne se retrouvent que chez les immunodéprimés ou en cas de corps étrangers (drains).
Le germe 5 se retrouve aussi chez des personnes de plus de 50 ans.
Les germes 6 à 8 se retrouvent principalement chez des enfants de moins de 6 mois.
Le germe 9 est mis en évidence par microscopie dans moins de 5 % méningites des tuberculoses.
**Occasionnellement, tout autre germe peut être retrouvé (*Pseudomonas spp.*, *Klebsiella spp.*, …)**

## MICROPHOTOGRAPHIE DE QUELQUES BACTERIES D'INTERET MEDICAL

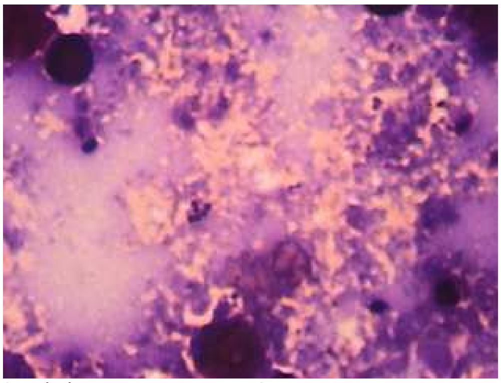

*Staphylococcus* spp., pus, Gram.

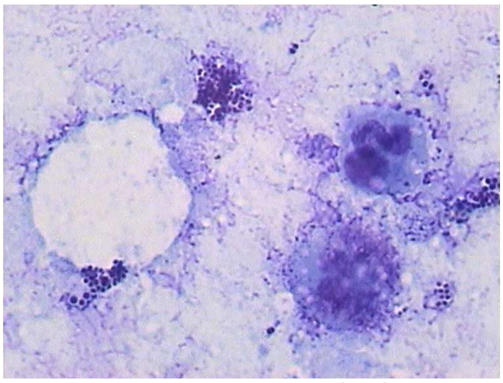

*Staphylococcus* spp., pus, bleu de méthylène.

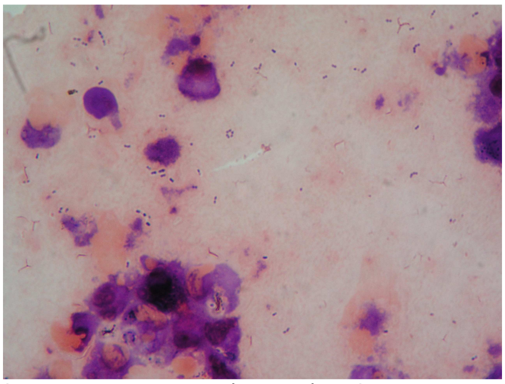

*Streptococcus pneumoniae*, crachat, Gram.

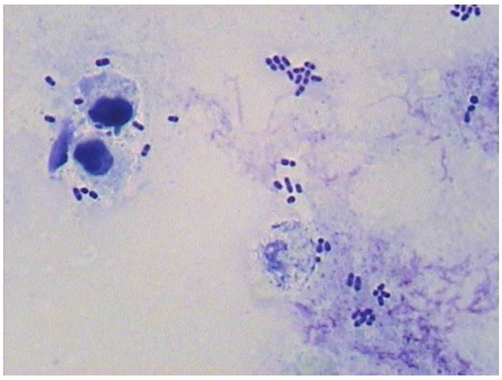

*Streptococcus mutans,* pus, bleu de méthylène.

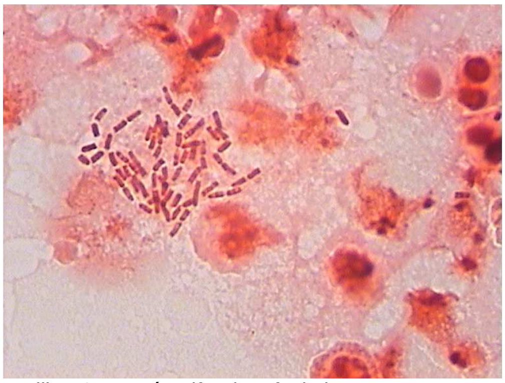

Bacilles Gram négatif, urine, fuchsine.

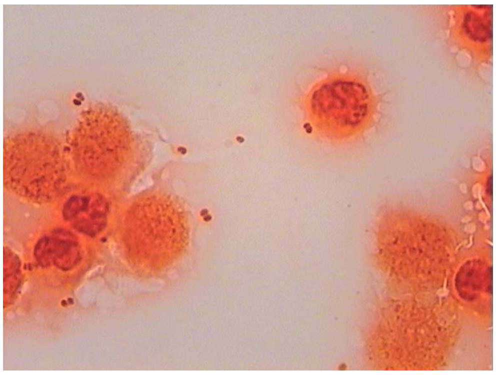

*Neisseria meningitidis,* LCR, fuchsine.

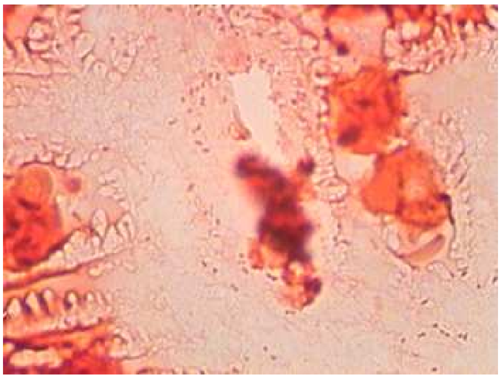

*Haemophilus influenzae*, LCR, fuchsine.

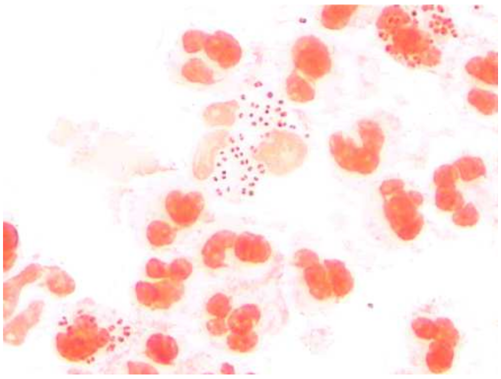

*Neisseria gonorrhoeae*, écoulement urétral, safranine.

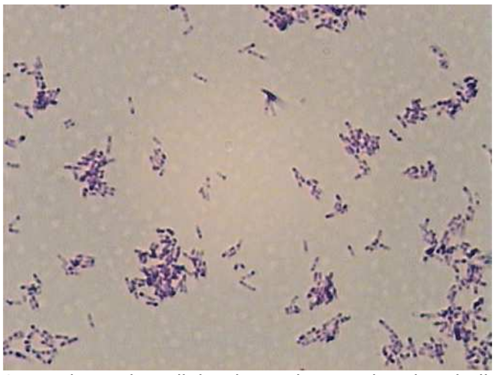

*Corynebacterium diphteriae,* culture, coloration d'Albert.

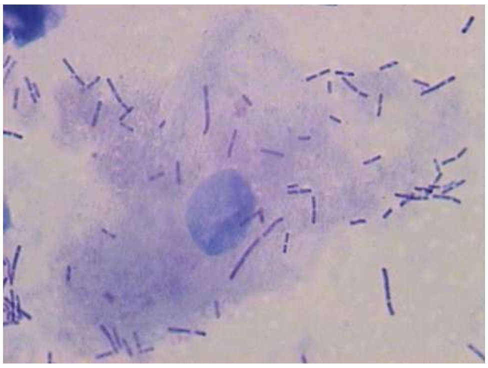

*Lactobacillus acidophilus,* sécrétion vaginale, bleu de méthylène

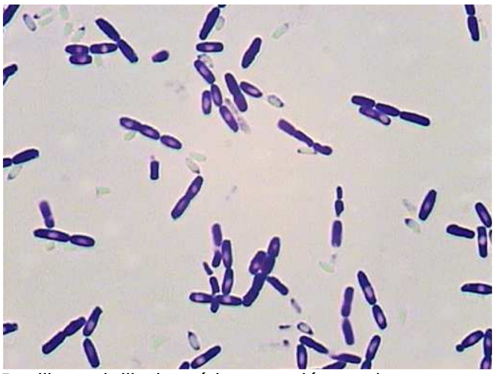

*Bacillus subtilis*, bactéries sporulées*,* culture, cristal violet.

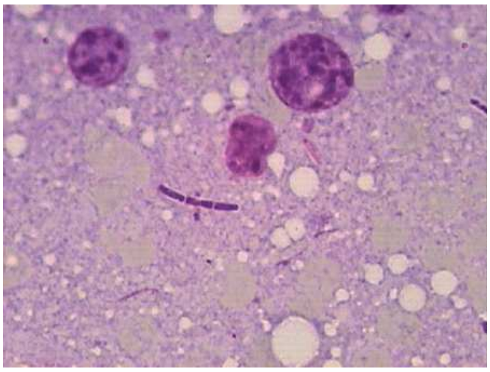

*Bacillus anthracis,* Deps, foie de souris, Giemsa.

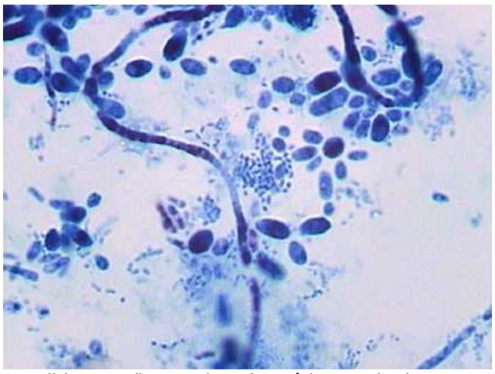

*Candida* spp. (levures) et bactéries vaginales, Ziehl.
Sécrétion vaginale, Bleu de méthylène.

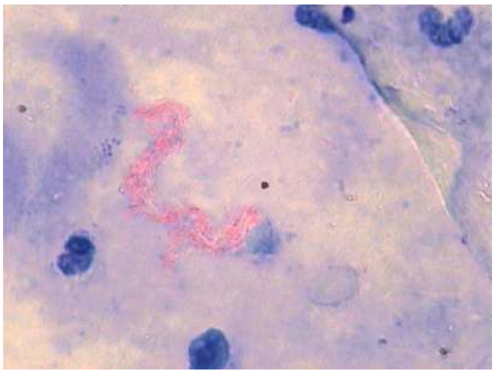

*Mycobacterium tuberculosis*, cordes, crachat,

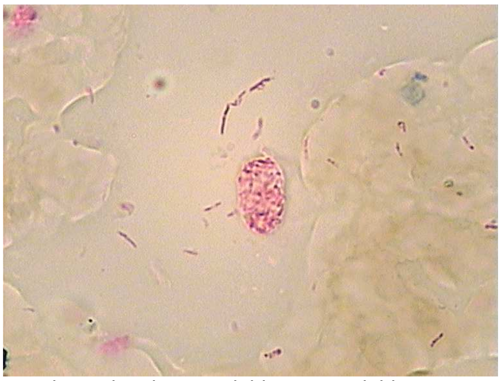

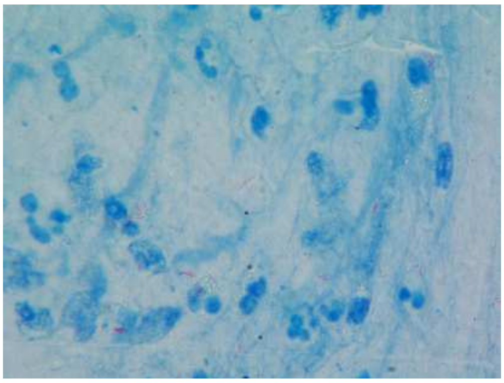

*Mycobacterium leprae,* globie, peau, Ziehl.
*Mycobacterium tuberculosis*, crachat, Ziehl.

## 4. Enregistrements et archives

| **Appendices & formulaires à compléter** | |
|---|---|
| **Numéro** | **Titre** |
| 1 | Cahier de laboratoire |

## 5. Histoire de document

| **Révision** | |
|---|---|
| | Version initiale |

| **Nom et fonction** | **Date** | **Signature** |
|---|---|---|
| *Auteur* | | |
| Barbara Barbé | 01/06/2012 | 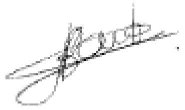 |
| *Révisé par* | | |
| Philippe Gillet | 10/09/2012 | 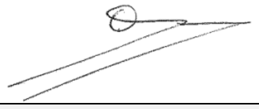 |
| *Approuvé par* | | |
| Veerle Lejon | 17/Sep/2012 | 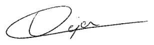 |

| | |
|---|---|
| nidiag – Better Diagnosis of Neglected Infectious Diseases | **SOP titre :** Coloration de Ziehl-Neelsen à chaud et lecture microscopique des lames. Détection des mycobactéries sur LCR |
| | **Projet/Étude :** NIDIAG : Evaluation de tests rapides en association avec des prédicteurs cliniques et de laboratoire pour le diagnostic de maladies tropicales négligées dans des patients se présentant avec des troubles neurologiques dans les hôpitaux ruraux au Bandundu, République Démocratique du Congo. |

# 1. Domaine et application

*Ce document fournit les instructions pour réaliser la coloration de Ziehl-Neelsen à chaud (coloration spécifique pour les mycobactéries) et pour faire la lecture semi-quantitative des lames colorées. La coloration est basée sur l'alcoolo-acido résistance des mycobactéries. Cette coloration est utilisée pour le diagnostic de neuro-tuberculose par la détection du Mycobacterium tuberculosis (bacille de Koch) dans des étalements de LCR (liquide céphalo-rachidien).*

# 2. Responsabilités

| Fonction | Activités |
|---|---|
| *Technicien de laboratoire* | ▪ *Prélève le LCR*<br>▪ *Exécute la coloration*<br>▪ *Réalise la lecture microscopique des lames*<br>▪ *Interprète le résultat*<br>▪ *Enregistre les résultats* |

# 3. Procédures

## 3.1 Précautions

- Tous les échantillons de LCR sont potentiellement infectieux. Respectez les précautions universelles. METTEZ DES GANTS À USAGE UNIQUE PENDANT TOUTE LA PROCÉDURE, **A L'EXCEPTION DE LA COLORATION (Risque de brûlures graves) !**
- L'alcool (éthanol, méthanol et alcool-acide) est inflammable, manipulez ce produit loin d'une flamme.
- L'Acide Chlorhydrique est extrêmement corrosif et ses vapeurs sont toxiques. Manipulez ce produit avec une extrême prudence et avec les fenêtres ouvertes.
- Le Phénol est extrêmement corrosif et toxique. Manipulez ce produit avec grande précaution.

## 3.2 Matériel et échantillons

### 3.2.1 Matériel requis

- Gants, non stérile, à usage unique
- Lame porte-objet neuve
- Crayon diamant
- Anse de platine
- Lampe à alcool
- Méthanol
- Solution de fuchsine de Ziehl (1:10) (du PNLT)
- Alcool-acide (3%) (ou acide sulfurique à 20 %)
- Solution de bleu de méthylène (1:10)
- Pince
- Coton
- Papier filtre
- Entonnoir
- Support de coloration des lames

- Support sèche lames
- Microscope (objectif 100 x)
- Huile à immersion
- Papier doux
- Minuterie

### 3.2.2 Echantillon à examiner

LCR frais : une goutte du culot du LCR centrifugé (tube original centrifugé15 minutes à 3.500 rpm)
(test réalisé uniquement pour les patients, avec plus de 5 globules blancs/µl de LCR)

## 3.3 Mode opératoire

### 3.3.1 Contrôle de qualité

Conservation de toutes les lames colorées. Relecture de toutes les lames lors des visites de supervision laboratoire de l'équipe d'Anvers.

### 3.3.2 Principe de la coloration

La coloration de Ziehl-Neelsen utilise 3 réactifs :

- La Fuchsine de Ziehl qui colore tout en rouge.

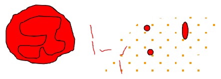

- L'alcool-acide (ou acide dilué) qui décolore tout à l'exception des mycobactéries.

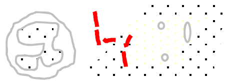

- Le bleu de méthylène qui contre-colore en bleu tout ce qui n'est pas coloré en rouge (pour augmenter le contraste).

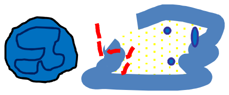

Les **Bacilles Alcoolo-Acido Résistant (BAAR)** sont donc de petits bâtonnets rouge-rosé sur un fond bleu.

### 3.3.3 Préparations des réactifs (uniquement en cas de rupture en réactifs livrés par le PNLT)

- Fuchsine de Ziehl, solution mère (saturée) :
  - Etiquetez une bouteille en verre brun de 250 ml avec « Solution saturée de fuchsine basique » et la date de préparation.
  - Introduisez le contenu d'un flacon de 25 g de Fuchsine basique dans la bouteille.
  - Ajoutez 250 ml d'éthanol à 96%.
  - Agitez la bouteille vigoureusement à 3 reprises dans la même journée.
  - Laissez déposer.
  - La solution est prête à l'emploi dès le lendemain.
  - Ajoutez de l'éthanol quand il reste un dépôt rouge.

  *Conservation* : quelques années dans un flacon brun hermétiquement bouché.

- Fuchsine de Ziehl, solution de travail à 1:10 :
  - Etiquetez un flacon en verre brun de 1000 ml avec « Fuchsine de Ziehl 10 % » et la date de préparation.
  - Filtrez 100 ml de solution saturée de fuchsine basique.
  - Versez le 100 ml de fuchsine basique filtré dans le flacon.
  - Ajoutez 900 ml de solution aqueuse de Phénol à 5 %
  - Filtrez la solution de travail avant l'emploi !

  *Conservation* : Au moins 2 ans dans un flacon en verre brun hermétiquement bouché.

- Alcool Acide de Ziehl à 3 % v/v :
    - Etiquetez un flacon d'un litre avec « Alcool Acide de Ziehl » et la date de préparation.
    - Versez 970 ml d'éthanol à 96% dans le flacon.
    - Ajoutez lentement 30 ml d'Acide Chlorhydrique en le laissent couler le long de la paroi.
      Attention : Le contenu s'échauffera.

  *Conservation* : quelques années dans un flacon brun hermétiquement bouché.

- Bleu de méthylène, solution mère (saturée) :
    - Etiquetez une bouteille brune de 250 ml avec « Bleu de méthylène saturé » et la date de préparation.
    - Introduisez le contenu d'un flacon de 25 g de bleu de méthylène (de qualité analytique) dans la bouteille.
    - Ajoutez 250 ml d'éthanol à 96%.
    - Agitez la bouteille vigoureusement à 3 reprises dans la même journée.
    - Laissez déposer.
    - La solution est prête à l'emploi dès le lendemain.
    - Ajoutez de l'éthanol quand il reste un dépôt de bleu de méthylène.

  *Conservation* : quelques années dans un flacon brun hermétiquement bouché.

- Bleu de méthylène, solution de travail à 1:10:
    - Etiquetez un flacon brun de 1000 ml avec « Bleu de méthylène 10 % » et la date de préparation.
    - Filtrez 100 ml de solution saturée de bleu de méthylène.
    - Versez le 100 ml de bleu de méthylène filtré dans le flacon.
    - Ajoutez 900 ml de l'eau distillée
    - Filtrez la solution de travail avant l'emploi !

  *Conservation* : Au moins 1 an dans un flacon brun hermétiquement bouché.

### 3.3.4 Procédure

44. Notez au crayon diamant le numéro du patient et la date sur une lame neuve.
45. Flambez la lame en la passant 3 x sur la flamme d'une lampe à alcool.
46. Flambez l'anse de platine (portez le fil au rouge sur toute sa longueur) puis laissez-la refroidir.
47. Etalez à l'aide de l'anse de platine une goutte du culot de centrifugation du LCR sur la lame flambée et faites un frottis aussi mince que possible.

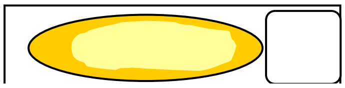

48. Reflambez l'anse de platine (portez le fil au rouge) pour détruire les bactéries.
49. Laissez sécher à l'air, à l'abri des insectes.
50. Recouvrez la lame de méthanol pur pour fixer le frottis.
51. Eliminez le méthanol et laisser sécher.
52. Déposez la lame fixée sur un support de coloration (des barres en position horizontales). Si plusieurs lames sont colorées en même temps, ne les laissez pas se toucher.
53. Recouvrez chaque lame de fuchsine de Ziehl préfiltrée.
54. Chauffez chaque lame individuellement par le dessous (bec bunsen, lampe à alcool ou pince portant un morceau de coton imbibé d'alcool et enflammé), jusqu'à émission de vapeurs. Ne pas faire bouillir.

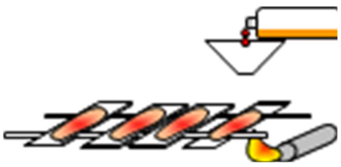

55. Laissez agir le colorant pendant au moins 5 minutes (maximum 10 minutes). Ne laissez pas sécher le colorant pour éviter la précipitation de la fuchsine.

Remarque : Un temps prolongé intensifie la coloration.

56. Rincez délicatement à l'eau courante.
57. Recouvrez la lame d'alcool acide (3 %). Laissez agir 1 minute.
58. Rincez délicatement à l'eau courante
59. Décolorez une seconde fois, si après rinçage la préparation n'est pas encore incolore.

    Remarque : Il est pratiquement impossible de décolorer les mycobactéries sauf *Mycobacterium leprae* et éventuellement des mycobactéries altérées par un traitement tuberculostatique.
60. Recouvrez la lame de bleu de méthylène préfiltré (10 %).
61. Laissez agir le colorant pendant 2 minutes.
62. Rincez délicatement à l'eau courante.
63. Laissez sécher à l'air libre.

    Remarque : Ne séchez pas les lames au soleil car cela diminuera l'intensité de la couleur rouge. Ne séchez pas les lames avec un papier (risque de transfert de bacilles).
64. Mettez une goutte de l'huile à immersion sur le côté gauche de l'étalement coloré.

    Remarque : Nettoyez l'objectif à immersion entre chaque lame pour éviter une contamination croisée.
65. Observez la lame au microscope (à immersion, objectif 100 x, oculaire 10 x).
66. Commencez à lire du côté gauche et bougez la lame longitudinal pour examiner les champs à côté. (100 champs = une longueur de +/- 2cm )
67. Examinez systématiquement la préparation pendant 5 - 10 minutes ou 100 champs microscopiques contigus.

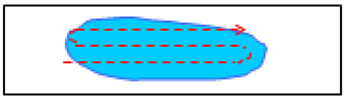

68. Caractéristiques des BAAR sur lame colorée au Ziehl-Neelsen (Figure 1):
    - 68.1 Couleur : Rouge, rosé sur contraste bleu
    - 68.2 Coloration uniforme ou irrégulière (même granulé)
    - 68.3 Taille : 3 - 5 µm
    - 68.4 Forme : fin, bâtonnet (bacilles), légèrement recourbé
    - 68.5 Présentation : isolé ou par deux ou groupés en amas ou en cordes. Immobile, sans formation de spores.

    Ne considérez pas et ne rapportez pas les autres bactéries observées.

    Attention : Ne pas confondre les BAAR avec : les Corynébactéries, les Actinomyces, les *Nocardia* spp., les oocystes de *Cryptosporidium,* …

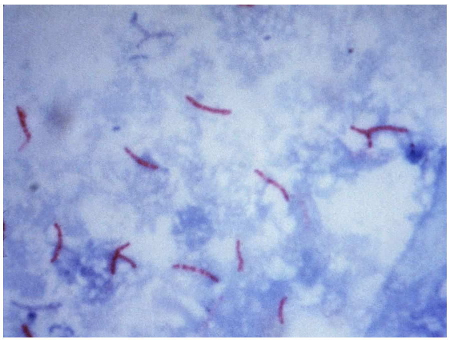

Figure 1. *Mycobacterium tuberculosis* coloration de Ziehl-Neelsen.

## 3.4 Rendu des résultats

- Négatif : Pas de BAAR vu dans 100 champs
- Positif : Détection de BAAR → méthode semi-quantitative (échelle OMS)

| **Nombre de BAAR par champ microscopique** (grossissement 1.000x) | **Nombre minimal de champs microscopiques à observer (objectif 100 x)** | **Résultats** |
|---|---|---|
| Absents | 100 | Négatif |
| 1 - 9 BAAR / 100 champs | 200-300 | Le nombre exact par 100 champs |
| 10 - 99 BAAR / 100 champs | 100 | 1+ |
| 1 - 10 BAAR / champ | 50 | 2+ |
| > 10 BAAR / champ | 20 | 3+ |

### 3.5 Sources d'erreurs

- Non-respect du temps de coloration.
- Surchauffe : décoloration difficile ou destruction de BAAR.
- Etalement trop épais : décoloration et lecture difficile.
- Mauvaise filtration des colorants : présence des dépôts et lecture difficile.
- Contamination des lames via transmission par l'huile à immersion.

### 3.6 Stockage et conservation des lames pour relecture (contrôle de qualité)

- Eliminez l'huile à immersion en déposant les lames sur un papier doux (face colorée sur le papier doux).
- Placez les lames nettoyées dans la boîte de rangement pour lames porte-objet, étiquetez clairement la boîte (Nom de l'étude, date de début de l'étude, type de lame, coloration, numéro de la boîte).

### 3.7 Contrôle de l'infection

Désinfectez avec une solution de Dettol à 10%, car les mycobactéries sont peu sensibles aux désinfectants chlorés (eau de Javel).

## 4. Définitions & abréviations

- BAAR: Bacilles Alcoolo-Acido résistants
- LCR : Liquide Céphalo-Rachidien
- Rpm : tours par minute

## 5. Enregistrements et archives

| **Appendices & formulaires à compléter** | |
|---|---|
| **Numéro** | **Titre** |
| 1 | CRF laboratoire |

## 6. Histoire de document

| **Révision** | |
|---|---|
| | Version initiale |

| **Nom et fonction** | **Date** | **Signature** |
|---|---|---|
| *Auteur* | | |
| Barbara Barbé | 01/06/2012 | |
| *Révisé par* | | |
| Philippe Gillet | 10/08/2012 | |
| *Approuvé par* | | |
| Veerle Lejon | 13/08/2012 | |

| | |
|---|---|
| | **SOP titre :** Système de détection pour l'antigène latex-Cryptococcose (IMMY) |
| | **Projet/Étude :** NIDIAG : Evaluation de tests rapides en association avec des prédicteurs cliniques et de laboratoire pour le diagnostic de maladies tropicales négligées dans des patients se présentant avec des troubles neurologiques dans les hôpitaux ruraux au Bandundu, République Démocratique du Congo. |

# 1. Domaine et application

Ce document fournit les instructions pour l'utilisation du test d'agglutination pour la détection qualitative ou semi-quantitative des antigènes polysaccharides capsulaires de *Cryptococcus neoformans* dans le LCR (liquide céphalorachidien). Ce test peut aider dans le diagnostic de la cryptococcose.

# 2. Responsabilités

| Fonction | Activités |
|---|---|
| Technicien de laboratoire | ▪ Prélève le LCR<br>▪ Exécute le test<br>▪ Interprète le résultat<br>▪ Enregistre les résultats |

# 3. Procédures

## 3.1 Précautions

- Tous les échantillons de LCR sont potentiellement infectieux. Respectez les précautions universelles. METTEZ DES GANTS À USAGE UNIQUE PENDANT TOUTE LA PROCÉDURE **!**
- Les réactifs contiennent de l'azide de sodium (0.095% p/v) qui est irritant pour la peau. EVITEZ TOUT CONTACT AVEC LA PEAU !
- Les réactifs contiennent de l'azide de sodium (0.095% p/v). NE JETEZ PAS LES REACTIFS DANS UN EVIER: le contact de l'azide avec du plomb ou du cuivre forme un produit explosif !
- NE MELANGEZ PAS LES REACTIFS AVEC UN ACIDE (risque de formation d'acide hydrazoïque, gaz très toxique).

## 3.2 Matériel et échantillons

### 3.2.1 Matériel fourni dans le kit et conservation

- <u>Diluant d'échantillon (réf. GB0020), 10 ml</u> : Une solution saline concentrée (10x) tamponnée par de la glycine (pH 8.6), contenant de l'albumine et un conservateur. Conservation : au frigo (2 – 8°C).
- <u>Réactif latex :Cryptococcose en latex (réf. CG0020 ou CG0010), 1,5 ml ou 3,5 ml</u> : Particules de latex sensibilisées par un anticorps de lapin anti-cryptocoques et contenant un conservateur. Conservation : au frigo (2 – 8°C). **NE PAS CONGELER !**
- <u>Contrôle positif d'antigènes de cryptocoques (réf. CB0010), 1 ml</u> : Solution d'antigènes polysaccharides capsulaires purifiés contenant un conservateur. Conservation : au frigo (2 – 8°C).
- <u>Contrôle négatif (réf. N80110), 1 ml</u> : Sérum de chèvre normal contenant un conservateur. Conservation : au frigo (2 – 8°C).
- Plaques de réaction jetables (réf. SC0020).
- Brochure explicative

### 3.2.2 Matériel supplémentaire requis

- Eau distillée

- Micropipettes de 25 µl et 100 µl
- Pipettes de 1 et de 10 ml à usage unique
- Embouts pour micropipette
- Tubes échantillons en verre borosilicate (non-siliconés),10-12 x 75 mm
- Portoir pour tubes échantillons
- Bain marie (56°C – 100°C)
- Bâtonnets applicateurs en bois
- Rotateur (100 rpm)
- Minuterie

### 3.5.3 Echantillon à examiner

- LCR : 150 µl
- Conservation de l'échantillon: Exécutez le test immédiatement. Sinon, réfrigérez ou congelez les échantillons (- 20°C).
  Remarque : Congélations et décongélations successives peuvent affecter les résultats du test.

## 3.6 Mode opératoire

### 3.2.1 Préparations des réactifs

- Diluant d'échantillon: Faites une dilution de 1:10. Prenez 1 ml du diluant et ajoutez 9 ml de l'eau distillé. Notez la date de dilution et la date d'expiration sur le flacon. Conservez au frigo, le réactif dilué est stable pendant 6 mois.
- Contrôle négatif: **Inactivez par chauffage le contrôle négatif (30 minutes à 56°C), avant la première utilisation.**
- Contrôlez l'homogénéité des solutions de latex.

### 3.2.2 Préparations des échantillons

- Liquide céphalorachidien (LCR):
  - Centrifugez 15 minutes à 3500 rpm
  - Transvasez le surnageant dans un tube en verre (borosilicate)
  - Fermez avec un bouchon.
  - **Incubez ce tube contenant le surnageant du LCR 5 minutes à 100°C (bain marie)**
  - L'échantillon ainsi traité est prêt à être analysé

### 3.6.3 Contrôle de qualité

- Contrôle positif et négatif à effectuer en parallèle avec chaque échantillon de patient qui est VIH positif (Determine, Unigold et DoubleCheck positif). Le contrôle positif du kit donne une réaction de 2+ ou supérieure, le contrôle négatif donne une réaction de 1+ ou moins. Interprétez les résultats des contrôles sur base du point 3.4. Interprétation des résultats.
- Contrôlez en parallèle avec chaque échantillon de patient qui est positif pour le test CrAg latex qualitatif la sensibilité du réactif latex : titrez le contrôle positif inclus dans le kit. Son titre doit être de 1:4 ± 1 dilution. Enregistrez les résultats dans le cahier de laboratoire. Changez de kit si ce résultat n'est pas obtenu.

### 3.6.4 Procédure

#### 3.6.4.1 Procédure de dépistage

1. Laissez revenir les réactifs à température ambiante.
2. Écrivez « contrôle positif », « contrôle négatif » et les numéros des patients sous chaque cercle de la plaque de réaction.

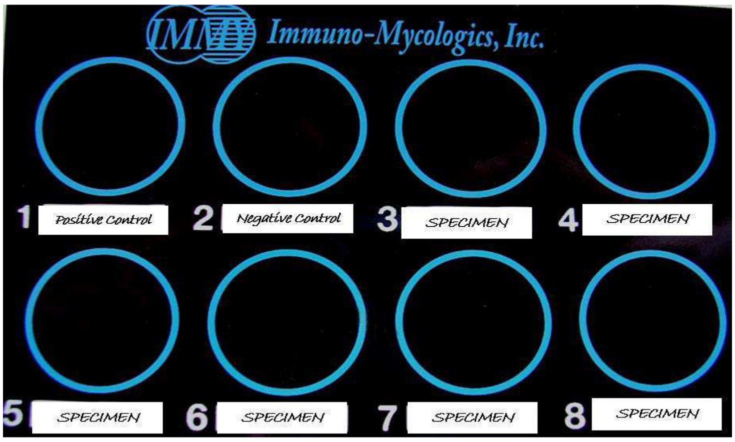


3. Déposez 25 µl du contrôle positif, du contrôle négatif et de chaque échantillon de LCR (traité par chauffage) dans les cercles correspondants. Utilisez un nouvel embout de pipette pour chaque réactif et pour chaque échantillon.
4. Ajoutez 25 µl du réactif latex dans chaque cercle.
5. Mélangez le contenu de chaque cercle, en utilisant un bâtonnet applicateur différent pour chaque cercle.
6. Placez la plaque de réaction sur un rotateur (100 rpm +/- 25) pendant 5 minutes à température ambiante.
7. Lisez les résultats immédiatement (Voir « 3.3.4.3 Lecture des réactions »)
8. Prenez une photo du résultat.
9. Notez dans le CRF laboratoire les résultats du test, y compris l'intensité de la réaction, le N° de lot du Kit et le N° de la photo

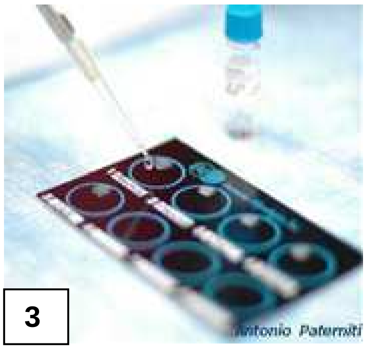


3

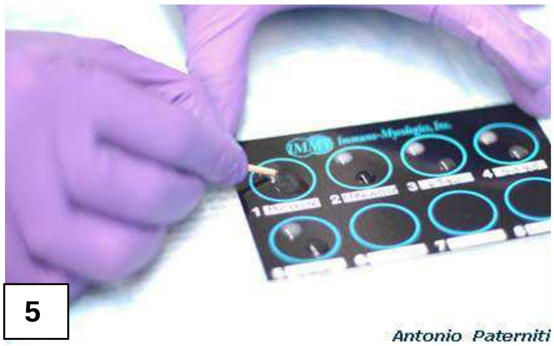


5

### 3.6.4.2 Procédure de titration (pour tous résultats qualitatifs positifs)

1. Pour tous les patients avec une réaction de dépistage positive (intensité 1+ ou supérieur) ! Préparez les dilutions de 2 en 2 en partant de 1 : 2 jusqu'à 1 : 1024

   a. Préparez 10 tubes à hémolyse dans un portoir.
   b. Mettez 100 µl du diluent d'échantillon à chacun des 10 tubes et placez les tubes dans une portoir.
   c. Numérotez les tubes de 1 à 10, inscrivez la dilution (comprise entre 1:2 et 1:1024) et le numéro du patient.
   d. Ajoutez 100 µl de LCR prétraité au tube 1 et homogénéisez (dilution 1:2). Prenez 100 µl du tube 1, ajoutez-le au tube 2 et homogénéisez (dilution 1:4). Continuez cettedilution de 2 en 2 jusqu'au tube 10 (dilution 1:1024).
2. Écrivez « contrôle positif », « contrôle négatif » et le numéros du patient et sa dilution sous chaque cercle de la plaque de réaction

3. Ajoutez 25 µl du contrôle positif, du contrôle négatif et de chaque dilution d'échantillon dans les cercles correspondants. Utilisez un nouveau embout de pipette pour chaque réactif et échantillon.
4. Ajoutez 25 µl du réactif latex dans chaque cercle.
5. Mélangez bien le contenu de chaque cercle, en utilisant un bâtonnet applicateur différent pour chaque cercle.
6. Placez la plaque de réaction sur un rotateur (100 rpm +/- 25) pendant 5 minutes à température ambiante.
7. Lisez les résultats immédiatement (Voir « 3.3.4.3 Lecture des réactions »)
   Remarque : Si l'échantillon est encore réactif à un titre 1:1024, préparez des dilutions additionnelles.
8. Prenez une photo du résultat.
9. Notez dans le CRF laboratoire les résultats du test, y compris le titre (dernière dilution positive), le N° de lot du Kit et le N° de la photo.

#### 3.6.4.3 Lecture des réactions

Lisez les réactions immédiatement contre un fond noir et déterminez l'intensité de la réaction sur une échelle de allant de négative jusqu'à 4+ (cf illustration). NE PAS OBSERVER LES RÉACTIONS AVEC UNE LOUPE!

Pour comparaison : le contrôle positif du kit donne une réaction de 2+ ou supérieure, le contrôle négatif donne une réaction de 1+ ou moins.

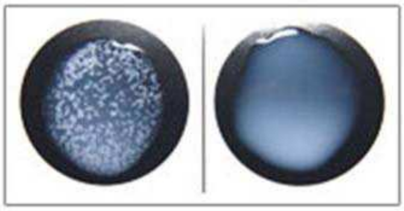

**Positif** **Négatif**

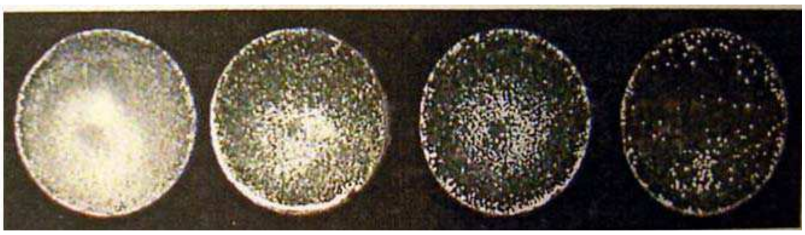

**1+** **2+** **3+** **4+**

Les différentes intensités de réaction sont :
**Négatif (-)** : suspension homogène de particules sans agglutination visible
**Un plus (1+)** : granulations fines sur un fond laiteux
**Deux plus (2+)** : petits amas mais définis sur un fond légèrement nuageux
**Trois plus (3+)** : petits et grands amas sur un fond clair
**Quatre plus (4+)** : grands amas sur un fond très clair

Enregistrez les intensités des réactions pour chaque échantillon. Le titre rapporté est la dilution la plus haute qui donne encore une réaction de 2+ ou supérieure.

### 3.7 Interprétation des résultats

- Contrôles :
  Le contrôle positif du kit doit donner une réaction de 2+ ou supérieure, tandis que le contrôle négatif doit donner une réaction de 1+ ou inférieure. Si l'un des contrôles donne un résultat incorrect, un réactif ou les deux est/sont insatisfaisants ou l'exécution de la procédure était incorrecte. Les résultats des échantillons obtenus ne peuvent être rapportés. Vérifiez votre procédure et recommencez le test. Si ce disfonctionnement persiste, utilisez un nouveau kit.
  N.B. : Une réaction positive du contrôle négatif peut indiquer une contamination ou une congélation du réactif latex, ce qui peut produire des réactions faussement positives pour les échantillons des patients.

- Échantillons des patients :
  - **Négatif** :
    Si la réaction du test de dépistage sur l'échantillon non-diluée est **négative ou 1+**, le résultat du test est négatif. Cependant, une réaction 1+ peut suggérer un effet prozone . Les échantillons qui donnent une réaction faible (1+) doivent être titrer (Voir « 3.3.4.2 Procédure de titration »). Si un effet de prozone est soupçonné, réalisez la procédure de titration (Voir « 3.3.4.2 Procédure de titration »).
  - **Positif** :
    Si la réaction du test de dépistage sur l'échantillon non-diluée est **2+ ou supérieure**, l'échantillon est titré selon la procédure de titration (Voir « 3.3.4.2 Procédure de titration »).
    Le titre rapporté est la dilution la plus haute qui donne une réaction de 2+ ou plus.

- Remarques :
  - Des réactions faussement négatives peuvent se produire en début d'infection, en présence des complexes immuns, suite à un effet de prozone ou pour des souches mal encapsulées (qui produisent peu de polysaccharides).
  - Des réactions faussement positives peuvent se produire en présence de facteur rhumatoïde, de *Capnocytophaga animorsus,* de *Trichosporin beigelii*, ou en cas d'un nettoyage insuffisant de la plaque de réaction.

### 3.5 Déchets, nettoyage

- Jetez l'excès de réactifs dans un conteneur « bio-hazard ». NE JETEZ PAS LES REACTIFS DANS UN EVIER: le contact de l'azide avec du plomb ou du cuivre forme un produit explosif !

## 4. Définitions & abréviations

- g : accélération de l'apesanteur
- LCR : Liquide céphalorachidien
- rpm : rotations par minute
- VIH : Le **virus de l'immunodéficience humaine**

## 5. Enregistrements et archives

| **Appendices & formulaires à completer** | |
|---|---|
| **Numéro** | **Titre** |
| 1 | Cahier de laboratoire |
| 2 | CRF "laboratoire" |

## 6. Références

- Instruction du producteur.

## 7. Historique du document

| **Révision** | |
|---|---|
| SOP-WP2-LAB-29-V01-18Jun2012 | Version initiale |
| SOP-WP2-LAB-29-V02-18Sep2012 | 3.3.3 Controle de qualité: Fréquence adapté |

| **Nom et fonction** | **Date** | **Signature** |
|---|---|---|
| *Auteur* | | |
| Barbara Barbé | 22/05/2012 | |
| *Révisé par* | | |
| Philippe Gillet | 16/09/2012 | |
| *Approuvé par* | | |
| Veerle Lejon | 18/Sep/2012 | |

| | |
|---|---|
| | **SOP titre:** Syphilis – Test RPR sur cartes Macro-Vue-BD |
| | **Projet/Étude** : NIDIAG : Evaluation de tests rapides en association avec des prédicteurs cliniques et de laboratoire pour le diagnostic de maladies tropicales négligées dans des patients se présentant avec des troubles neurologiques dans les hôpitaux ruraux au Bandundu, République Démocratique du Congo. |

# 1. Domaine et application

*Ce document fournit les instructions pour l'exécution du test RPR (Réagine Rapide de Plasma) sur carte Macro-Vue (avec cercle de 18 mm). Ce test est une méthode de diagnostic de la syphilis par sérologie non-tréponémique. La réagine - similaire aux anticorps - présente dans le sérum ou le plasma des patients syphilitiques réagit avec des particules de carbone antigénées sur la carte RPR. Pour les échantillons positifs, une floculation macroscopique se produit sous la forme d'amas noirs sur le fond blanc de la carte RPR, tandis que les échantillons non-réactifs ont une couleur gris clair uniforme.*

# 2. Responsabilités

| Fonction | Activités |
|---|---|
| *Technicien de laboratoire* | ▪ *Prélève le sang*<br>▪ *Exécute le test*<br>▪ *Interprète le résultat*<br>▪ *Enregistre les résultats* |

# 3. Procédures

## 3.1 Précautions

- Tous les échantillons de sang sont potentiellement infectieux. Respectez les précautions universelles.
  METTEZ DES GANTS À USAGE UNIQUE PENDANT TOUTE LA PROCÉDURE **!**
- Le diluent contient de l'azide de sodium, qui est irritant pour la peau.
  EVITEZ TOUT CONTACT AVEC LA PEAU !
- Les réactifs contiennent de l'azide de sodium. NE JETEZ PAS LES REACTIFS DANS UN EVIER:
  le contact de l'azide avec du plomb ou du cuivre forme un produit explosif !

## 3.2 Matériel et échantillons

### 3.2.1 Matériel fourni dans le kit et conservation

- Cartes RPR Macro-Vue :
  *Conservation* : à température ambiante
- Ampoule de suspension d'antigènes (RPR Card Antigen Suspension) :
  *Conservation :* au réfrigérateur (2-8°C). Lors de l'utilisation, versez la suspension d'antigène dans le flacon distributeur, compris dans le kit. Conservez le flacon distributeur rempli au réfrigérateur (2-8°C).
  Laisser revenir à température ambiante avant de l'utiliser.
  *Stabilité :* Lors du transvasement dans le flacon distributeur, la suspension d'antigènes est stable pendant 3 mois, ou jusqu'à la date de péremption (s'il reste moins de 3 mois avant expiration).
- Flacon distributeur
- Capillaires
- Dispositifs « Dispenstirs »
- Aiguilles jaune, 20 G
- Brochure explicative

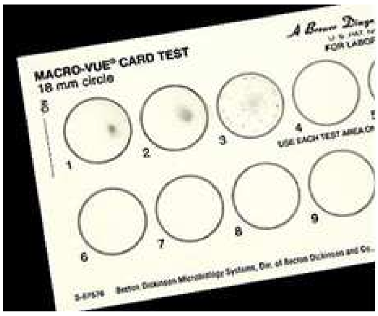

### 3.2.2 Matériel supplémentaire requis

- Contrôles liquides à différentes réactivités (BD Macro-Vue RPR Card Test Liquid Controls) : 3 niveaux de réactivité.
  *Conservation* : au réfrigérateur (2-8°C). Evitez toute contamination microbienne. Après ouverture des flacons, utilisez les réactifs dans les 10 mois ou jusqu'à la date de péremption (s'il reste moins de 10 mois avant expiration). Laissez revenir à température ambiante avant de les utiliser.
- Micropipette pour les volumes 50 µl, 100 µl, 500 et 1000 µl
- Embouts jaunes et bleus pour micropipette
- Pipette ou seringue à 1 ml
- Solution d'eau physiologique (NaCl 0,9%).
- Rotateur mécanique à 100 rpm (± 2 rpm)
- Protège-carte humidificateur (contenant un papier buvard ou une éponge humide)
- Minuterie
- Conteneur« bio-hazard »

### 3.2.2 Echantillon à examiner

- Sérum: 50 à 250 µl
- *Conservation de l'échantillon* :
  *Sérum*: Exécutez le test immédiatement. Sinon, réfrigérez au maximum 5 jours à 2-8°C, ou congelez à - 20°C.
  Remarque : Des congélations et des décongélations successibles peuvent affecter les résultats du test.
- *Critères de rejet* : Echantillons fortement hémolysés, contaminés ou très turbides.

## 3.3 Mode opératoire

### 3.3.1 Préparations des réactifs

- Pour le test qualitatif/quantitatif :
  - Laissez revenir les contrôles liquides et la suspension d'antigènes à température ambiante.
  - Agitez vigoureusement l'ampoule de suspension d'antigènes pendant 10 à 15 secondes pour remettre l'antigène en suspension et pour distribuer toute particule de charbon qui serait dans le goulot de l'ampoule.
  - Attachez l'aiguille au raccord conique du flacon distributeur. Cassez le goulot de l'ampoule à l'aide du dispositif d'ouverture préinstallé et versez la suspension d'antigènes dans le flacon distributeur. Notez le numéro de lot, la date de péremption et la date du transvasement sur le flacon distributeur.
  - Vérifiez le débit de l'aiguille (jaune, 20 G). Placez l'aiguille fermement sur une seringue de 1 ml. Aspirez 0,5 ml de la suspension d'antigène, maintenez la pipette ou la seringue verticalement et comptez le nombre de gouttes distribuées. Le nombre correct de gouttes dans 0,5 ml est 30 ± 1 goutte.
- Pour le test quantitatif :
  - Préparez une dilution à 1:50 du sérum non-réactif (Contrôle -) avec une solution saline (0,9%) : Ajoutez 0,20 ml de sérum non-réactif (4 gouttes du contrôle négatif) à 10 ml de solution saline (NaCl à 0,9%). Mélangez bien.
    Identifiez la solution (nom, date de préparation et date d'expiration) et conservez cette solution au frigo. Cette solution est stable au frigo pendant 3 mois.

### 3.3.2 Contrôle de qualité

- Vérifiez chaque jour la vitesse de rotation du rotateur, elle doit être comprise entre 98 et 102 rpm. Adaptez si nécessaire.

- Vérifiez **chaque semaine** la suspension d'antigènes à l'aide des contrôles liquides à différentes réactivités (BD Macro-Vue RPR Card Test Liquid Controls) par la procédure qualitative:
  - o **Contrôle ++** (1,5 ml) : le Macro-Vue RPR Card Test Contrôle de Sérum Réactif
  - o **Contrôle +** (1,5 ml) : le Macro-Vue RPR Card Test Contrôle de Sérum Réactif Modéré
  - o **Contrôle -** (1,5 ml) : le Macro-Vue RPR Card Test Contrôle de Sérum Négatif

  Voir point « 4.2.4.1 procédure qualitative ».

- Interprétation des résultats de contrôles liquides (procédure qualitative) :
  - o Contrôle ++ doit donner une agglutination caractéristique.
  - o Contrôle + doit donner une agglutination minimale à modérée, inférieure à celle du contrôle ++
  - o Contrôle - doit donner l'aspect gris homogène de particules non-agglutinées.

  ➔ Utilisez seulement les suspensions d'antigènes qui présentent les réactions attendues qui sont décrites ci-dessus.

- Vérifiez **en même temps que chaque patient positif** la suspension d'antigène à l'aide des contrôles liquides à différentes réactivités (BD Macro-Vue RPR Card Test Liquid Controls) par la procédure quantitative (Voir point « 4.2.4.2 procédure qualitative »).

- Interprétation des résultats des dilutions de contrôles liquides (procédure quantitative, chaque semaine) :

| | 1:1 | 1:2 | 1:4 | 1:8 | 1:16 |
|---|---|---|---|---|---|
| Control ++ | R | R | R | Rm | N |
| Control + | R | Rm | N | N | N |
| Control – | N | N | N | N | N |

R = Réactif  Rm = Réactif minimal  N = Non réactif

➔ Utilisez seulement les suspensions d'antigènes qui présentent les réactions attendues décrites ci-dessus.

### 3.3.3 Procédure

#### 3.3.3.1 Procédure qualitative

1. Laissez revenir les réactifs à température ambiante.
2. Humidifiez le protège-carte humidificateur du rotateur.
3. Mettez des gants à usage unique.
4. Identifiez la carte et les cercles (cercle 1 : contrôle ++, cercle 2 : contrôle +, cercle 3 : contrôle -, cercle 4 : numéro du patient, cercle 5 numéro du deuxième patient, …) et notez la date.
   **Remarque :** Évitez de poser les doigts sur les zones de tests (cercles), car les empreintes peuvent entraîner des résultats incorrects.
5. Faites tomber une goutte (50 µl) de Macro-Vue RPR Sérum de contrôle Réactif fort (Contrôle ++) sur le cercle identifié de la carte Macro-Vue. Faites de même pour le Macro-Vue RPR Sérum de contrôle Réactif Modéré (Contrôle +) et pour la contrôle négatif (Contrôle -).
6. Aspirez l'échantillon du patient dans le dispositif « Dispenstirs ».
7. Maintenez le dispositif vertical et laissez tomber une goutte d'échantillon (± 50 µl) sur le cercle identifié de la carte Macro-Vue, sans toucher la carte. Utilisez un dispositif neuf pour chaque échantillon.
8. Retournez le dispositif « Dispenstirs » et étalez l'échantillon en couvrant toute la surface du cercle. Utilisez un dispositif neuf pour chaque échantillon.

Remarque : Évitez de rayer la carte avec le dispositif. Si l'échantillon ne s'étale pas jusqu'au périmètre extérieur du cercle, utilisez un autre cercle de la carte.

9. Agitez doucement le flacon distributeur d'antigènes.
10. Maintenez le flacon distributeur vertical et distribuez plusieurs gouttes dans le bouchon du flacon distributeur pour être sûr que le passage de l'aiguille est dégagé. Ré-aspirez le réactif du bouchon dans le distributeur.
11. Laissez tomber librement une goutte de la suspension d'antigènes sur chaque cercle identifié.
    Remarque : NE PAS REMÉLANGER ! Le mélange de l'échantillon et de l'antigène se produit pendant la rotation.
12. Agitez pendant 8 min, avec le protège-carte humide, sur le rotateur mécanique à 100 rpm.
13. Agitez brièvement par inclinaison manuelle de la carte (3 – 4 mouvements).
14. Lisez la carte immédiatement à l'état humide sous une lampe à incandescence de forte puissance ou sous forte lumière du jour. (Voir « 3.2.3.3 Lecture des réactions »)
15. Prenez une photo du résultat.
16. Si le résultat est « Réactif », poursuivez avec la procédure quantitative. (Voir « 3.2.3.2 Procédure quantitative »). Si le résultat est « Non-réactif », arrêtez et rapportez le résultat dans le CRF laboratoire.

#### 3.3.3.2 Procédure quantitative (en cas de procédure qualitative réactive)

1. Laissez revenir la suspension d'antigènes (dans le flacon distributeur) à température ambiante.
2. Humidifiez protège-carte humidificateur du rotateur.
3. Mettez des gants à usage unique.
4. Notez le numéro de patient et la date sur la carte RPR et notez les dilutions sous les cercles à identifier : cercle 1 (non-dilué), cercle 2 (1:2), cercle 3 (1:4), cercle 4 (1:8), cercle 5 (1:16).
   **Remarque :** Évitez de poser les doigts sur les zones de tests (cercles), car les empreintes peuvent entraîner des résultats incorrects.
5. Pour chaque échantillon à tester, déposez avec un « Dispenstirs » 50 µl de solution saline (NaCl à 0,9 %) dans les cercles 2 à 5. **NE PAS ÉTALER LA SOLUTION SALINE !**
6. Déposez avec un « Dispenstirs » une goutte (50 µl) de l'échantillon sur le cercle 1.
7. Déposez avec un « Dispenstirs » une goutte (50 µl) de l'échantillon sur le cercle 2. Aspirez et refoulez le mélange de solution saline et d'échantillon 5 ou 6 fois.
8. Préparez une série de dilutions :
   Transférez 50 µl du cercle 2 vers le cercle 3, du cercle 3 vers le cercle 4 et du cercle 4 vers le cercle 5. Mélangez après chaque transfert en aspirant et en refoulant le mélange. Eliminez 50 µl du cercle 5 après avoir mélangé son contenu. Evitez la formation de bulles.
   Les dilutions obtenues: cercle 1 (non-dilué), cercle 2 (1:2), cercle 3 (1:4), cercle 4 (1:8), cercle 5 (1:16).
9. Étalez le contenu de chaque cercle avec un dispositif « Dispenstirs ». Commencez par la plus grande dilution (cercle 5) et étalez sur toute la surface du cercle. Déplacez-vous aux cercles 4, 3, 2 et 1 et étalez de la même manière.
10. Agitez doucement le flacon distributeur d'antigènes.
11. Maintenez le flacon distributeur vertical et distribuez plusieurs gouttes dans le bouchon du flacon distributeur pour être sûr que le passage de l'aiguille est dégagé. Ré-aspirez le réactif du bouchon dans le distributeur.
12. Laissez tomber librement une goutte de la suspension d'antigènes sur chaque cercle identifié.
    **Remarque :** NE PAS REMÉLANGER ! Le mélange de l'échantillon et de l'antigène se produit pendant la rotation.

13. Agitez pendant 8 min, avec le protège-carte humide, sur le rotateur mécanique à 100 rpm.
14. Agitez brièvement par inclinaison manuelle de la carte (3 – 4 mouvements).
15. Lisez la carte immédiatement à l'état humide sous une lampe à incandescence de forte puissance ou sous forte lumière du jour. (Voir « 3.2.3.3 Lecture des réactions »).
16. Prenez une photo du résultat.
17. Rapportez la dilution la plus élevée présentant une réaction « Réactif », y compris des réactions minimales à modérées, dans le CRF de laboratoire.

**Si la dilution la plus élevée (1:16) est réactive, procédez comme suit :**

1. Préparez une dilution à 1:16 de l'échantillon en ajoutant 100 µl de l'échantillon à 1,5 ml de solution saline (NaCl à 0,9 %). Mélangez bien.
2. Prenez le sérum non-réactif dilué à 1:50. (Voir « 3.2.1 Préparations des réactifs »)
3. Humidifiez protège-carte humidificateur du rotateur.
4. Mettez des gants à usage unique.
5. Notez le numéro de patient et la date sur la carte RPR et notez les dilutions sous les cercles à identifier : cercle 1 (1 :16), cercle 2 (1:32), cercle 3 (1:64), cercle 4 (1:128), cercle 5 (1:256).
6. **Remarque :** Évitez de poser les doigts sur les zones de tests (cercles), car les empreintes peuvent entraîner des résultats incorrects
7. Déposez avec un « Dispenstirs » 50 µl de sérum non-réactif à 1:50 dans les cercles 2, 3, 4 et 5 avec une micropipette.
8. Déposez avec un « Dispenstirs » 50 µl de la dilution à 1:16 de l'échantillon dans le cercle 1 avec un capillaire.
9. Déposez avec un « Dispenstirs » 50 µl de la dilution à 1:16 de l'échantillon dans le cercle 2.
10. Faites une dilution sériée. Suivez la procédure décrite dessus (points 7 à 17). Les dilutions obtenues : cercle 1 (1:16), cercle 2 (1:32), cercle 3 (1:64), cercle 4 (1:128), cercle 5 (1:256).

#### 3.3.3.3 Lecture des réactions

- Lisez la réaction immédiatement après avoir agité 8 minutes, à l'état "humide" sous une lampe à incandescence de forte puissance ou sous forte lumière du jour.
- Interprétation des réactions (procédure qualitative):
  - **Réactif** : une agglutination caractéristique allant de légère mais définie (minimale à modérée) jusqu'à marquée et intense.
  - **Non-réactif** : Aucune agglutination, aspect gris homogène.

Si le résultat est « Réactif », continuez avec la procédure quantitative.

**Remarques** ***:***

- Rapportez une réaction légèrement granuleuse ou d'apparence rugueuse comme « indéterminée ».
- Un effet de prozone peut survenir avec les échantillons montrant une telle réaction (dans 1 - 2% des patients avec une syphilis secondaire). Diluez ces échantillons pour exclure ou montrer l'effet de prozone. (Voir « 3.2.3.2 Procédure quantitative »)

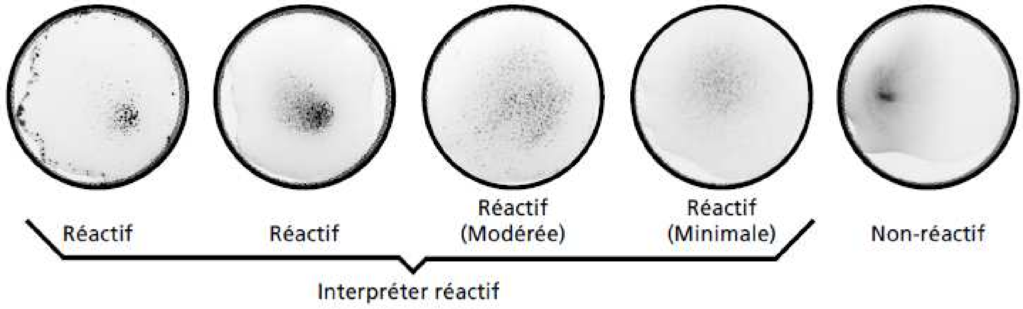


- Interprétation des réactions (procédure quantitative) :

  Interprétez les dilutions comme « Réactif » ou « Non-réactif ». Rapportez comme résultat la dilution la plus élevée présentant une réaction « Réactif », y compris minimale à modérée.
  Exemple :

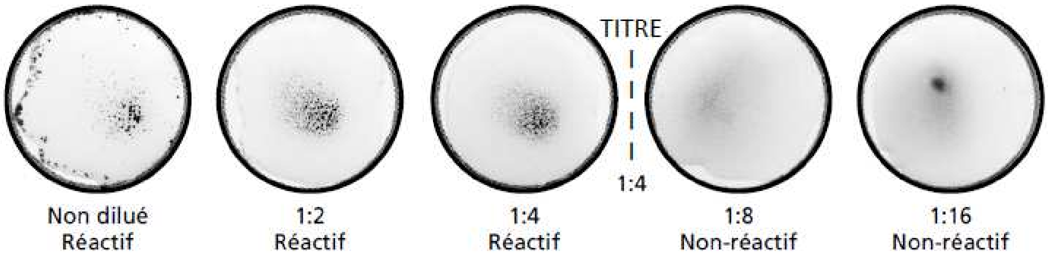


### 3.4 Déchets, nettoyage

- Jetez les cartes complètement utilisées dans un conteneur « bio-hazard ».
- Jetez l'aiguille et le flacon distributeur dans un conteneur « bio-hazard » lorsque le kit est utilisé complètement.

## 4. Définitions & abréviations

- RPR : Rapid Plasma Reagin (Réagine Rapide de Plasma)
- RPM : Rounds per minute (tours par minute)

## 5. Enregistrements et archives

| **Appendices & formulaires à compléter** | |
|---|---|
| **Numéro** | **Titre** |
| 1 | CRF de laboratoire |

## 6. Histoire de document

| **Révision** | |
|---|---|
| | Version initiale |

| **Nom et fonction** | **Date** | **Signature** |
|---|---|---|
| *Auteur* | | |
| Barbara Barbé | 30/05/2012 | |
| *Révisé par* | | |
| Philippe Gillet | 09/09/2012 | |
| *Approuvé par* | | |
| Veerle Lejon | 17/Sep/2012 | |

| | |
|---|---|
| | **SOP titre** : CrAg Lateral Flow Assay: Système de détection de l'antigène des Cryptocoques (IMMY) |
| | **Projet/Étude :** NIDIAG : Evaluation de tests rapides en association avec des prédicteurs cliniques et de laboratoire pour le diagnostic de maladies tropicales négligées dans des patients se présentant avec des troubles neurologiques dans les hôpitaux ruraux au Bandundu, République Démocratique du Congo. |

# 1. Domaine et application

Ce document fournit les instructions pour l'utilisation du CrAg lateral flow assay (LFA) pour la détection qualitative des antigènes polysaccharides capsulaires du complexe des espèces de *Cryptococcus* (*Cryptococcus neoformans* et *Cryptococcus gattii*) dans le sérum, l'urine et le liquide céphalorachidien (LCR). C'est un test immunochromatographique sur bandelette qui peut aider dans le diagnostic de la cryptococcose.

# 2. Responsabilités

| Fonction | Activités |
|---|---|
| Technicien de laboratoire | ▪ Prélève le sang, l'urine et le LCR<br>▪ Sépare le sérum<br>▪ Exécute le test<br>▪ Interprète le résultat<br>▪ Enregistre les résultats |

# 3. Procédures

## 3.1 Précautions

- Tous les échantillons de sang et de LCR sont potentiellement infectieux. Respectez les précautions universelles. PORTEZ DES GANTS À USAGE UNIQUE PENDANT TOUTE LA PROCÉDURE **!**
- Certains réactifs contiennent un conservateur (de l'azide de sodium à 0.095% p/v), qui est irritant pour la peau. EVITEZ TOUT CONTACT AVEC LA PEAU !
- Certains réactifs contiennent un conservateur (de l'azide de sodium à 0.095% p/v), NE JETEZ PAS LES REACTIFS DANS UN EVIER: le contact de l'azide avec le plomb ou le cuivre des canalisation forme un produit explosif !

## 3.2 Matériel et échantillons

### 3.2.1 Matériel fourni dans le kit et conservation

- <u>Diluant d'échantillon LF (réf. GLF025), 2,5 ml</u> : Solution saline tamponnée par la glycine et contenant des agents bloquants et un conservateur. Conservation : à température ambiante.
- <u>[Diluant de titration LF (réf. EI0010), 6 ml</u> : Solution saline tamponnée par la glycine et contenant un conservateur. Conservation : à température ambiante]. **REACTIF A UTILISER UNIQUEMENT POUR LA PROCEDURE QUANTITATIVE! NE PAS A UTILISER DANS L'ETUDE NIDIAG!**
- <u>Contrôle positif CrAg (réf. CB1020), 1 ml</u> : Solution saline tamponnée par la glycine surchargée en antigènes de cryptocoque (souche 184A – isolat clinique de l'Université de Tulane). Conservation : à température ambiante.
- <u>Bandelettes CrAg LF (réf. LFCR50), 50 bandelettes</u> dans un flacon hermétique. Conservation : à température ambiante. **NE PAS CONGELER !** Conservez les bandelettes dans ce flacon spécifique. Refermez le flacon après usage (le bouchon contient du silicagel pour absorber l'humidité).
- Brochure explicative

#### 3.2.2 Matériel supplémentaire requis

- Micropipettes à 40 µl et 80 µl
- Embouts pour micropipette
- Tubes à hémolyse
- Minuterie

#### 3.2.3 Echantillon à examiner

- LCR, sérum (non-hémolysé) ou urine : 40 µl
  Le sérum hémolysé cause un arrière fond rougeâtre sur la bandelette, ce qui peut donner des résultats faussement négatifs
- Conservation de l'échantillon: Exécutez le test immédiatement. Sinon, réfrigérez l'échantillon à 2-8°C pour maximum 72 heures ou congelez les échantillons à - 20°C pour des périodes plus longues.
  Remarque : Les congélations et décongélations successives peuvent affecter les résultats du test.
- Critères de rejet: Rejetez les échantillons lipémiques et les échantillons qui contiennent des bactéries, ou qui montrent d'autres signes de contamination.

### 3.3 Mode opératoire

#### 3.3.1 Contrôle de qualité (une fois par semaine)

- Contrôle positif :
  - Mettez une goutte de diluant d'échantillon LF dans un tube à hémolyse,
  - Ajoutez une goutte de contrôle positif CrAg.
  - Homogénéisez.
  - Immergez l'extrémité blanche de la bandelette CrAg LF dans le tube.
  - Lancez la minuterie (10 minutes).
  - Lisez le résultat après 10 minutes (Voir « 3.3.4 Lecture du test »).
  - Enregistrez les résultats dans le cahier de laboratoire.

- Contrôle négatif :
  - Mettez deux gouttes de diluant d'échantillon LF dans un tube.
  - Homogénéisez.
  - Immergez l'extrémité blanche de la bandelette CrAg LF dans le tube.
  - Lancez la minuterie (10 minutes).
  - Lisez le résultat après 10 minutes (Voir « 3.3.4 Lecture du test »).
  - Enregistrez les résultats dans le cahier de laboratoire.

#### 3.3.2 Procédure qualitative

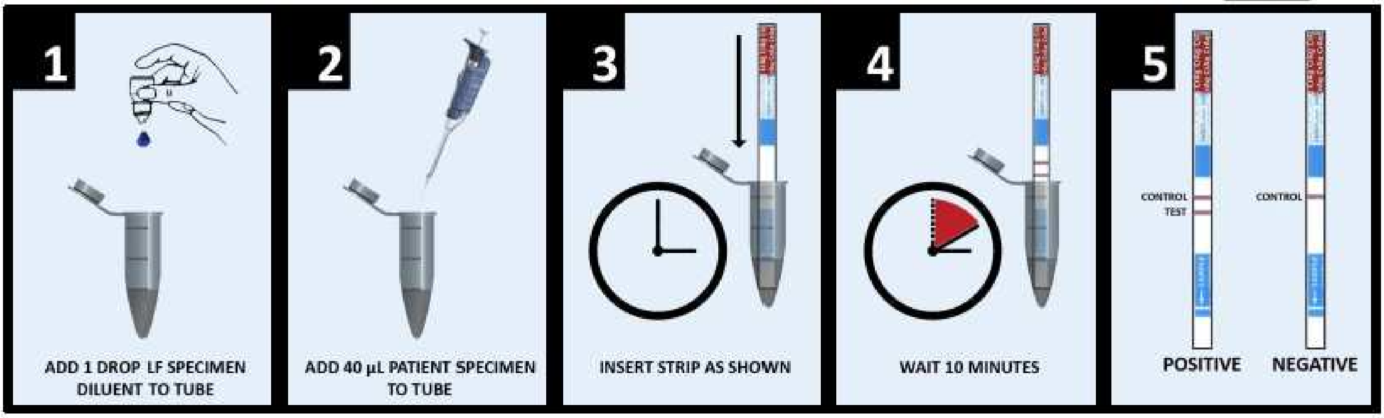


1. Mettez une goutte de diluant d'échantillon LF dans tube à hémolyse.
2. Ajoutez 40 µl d'échantillon (sérum, urine ou LCR).
3. Homogénéisez.

4. Immergez l'extrémité blanche de la bandelette CrAg LF dans l'échantillon.
5. Lancez la minuterie (10 minutes)
6. Lisez le résultat après 10 minutes (Voir « 3.3.4 Lecture du test »).
7. Prenez une photo du résultat.
8. Notez dans le CRF laboratoire les résultats du test, y compris l'intensité de la bande du test, le N° de lot du Kit et le N° de la photo

### 3.3.3 Lecture du test

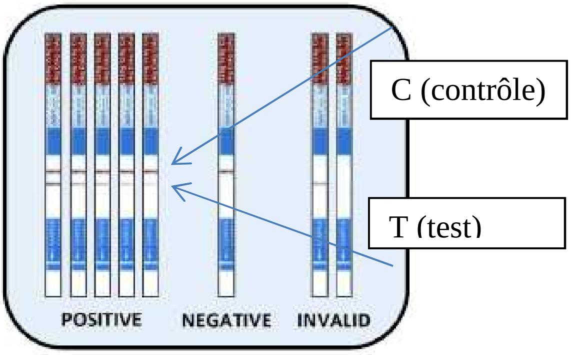


- Absence de la ligne de contrôle : **Invalide**
  RÉPÉTEZ LE TEST !

- Présence des deux lignes (test et contrôle), quel que soit l'intensité des lignes : **Positif**

- Présence d'une seule ligne (contrôle). **Négatif**

## 3.4 Interprétation des résultats

- La présence de la ligne de contrôle est obligatoire pour avoir un résultat valide.
- Un résultat négatif n'exclut pas le diagnostic de la maladie. L'échantillon peut avoir été prélevé avant que les antigènes ne soient détectables.
- L'intensité de la bande de test ne correspond pas à la quantité totale d'antigènes présents.
- Des réactions faussement négatives peuvent se produire pour des échantillons contenant un grande quantité d'antigènes (suite à un effet de prozone).

## 3.5 Déchets, nettoyage

Jetez les bandelettes et l'excès de réactifs dans un conteneur « bio-hazard »..

# 4. Définitions & abréviations

- CrAg : Cryptococcal antigen
- LCR : Liquide céphalorachidien
- LF : Lateral flow
- LFA : Lateral flow assay

# 5. Enregistrements et archives

| Appendices & formulaires à completer | |
|---|---|
| **Numéro** | **Titre** |
| 1 | Cahier de laboratoire |
| 2 | CRF "laboratoire" |

# 6. Références

- Lindsley MD, et al (2011). Evaluation of a newly developed lateral flow immunoassay for the diagnosis of cryptococcosis. Clin Infect Dis;53(4):321-325.
- Instruction du producteur, version 10/14/2011

# 7. Historique du document

| Révision | |
|---|---|
| SOP-WP2-LAB-31-V01-12Jul2012 | Version initiale |
| SOP-WP2-LAB-31-V02-18Sep2012 | Procédure semi-quantitative enlevé |

| **Nom et fonction** | **Date** | **Signature** |
|---|---|---|
| *Auteur* | | |
| Barbara Barbé | 22/05/2012 | |
| *Révisé par* | | |
| Philippe Gillet | 16/09/2012 | |
| *Approuvé par* | | |
| Veerle Lejon | 18/09/2012 | |

| | |
|---|---|
| nidiag Better Diagnosis of Neglected Infectious Diseases | **SOP titre :** Determine HIV-1/2 |
| | **Projet/Étude :** NIDIAG : Evaluation de tests rapides en association avec des prédicteurs cliniques et de laboratoire pour le diagnostic de maladies tropicales négligées dans des patients se présentant avec des troubles neurologiques dans les hôpitaux ruraux au Bandundu, République Démocratique du Congo. |

# 1. Domaine et application

Ce document fournit les instructions pour l'exécution du test rapide immuno-chromotgraphique Determine HIV-1/2. Ce test détecte les anticorps anti-VIH-1 et anti-VIH-2 dans le sérum, le plasma ou le sang total et constitue ainsi une aide sur le diagnostic de VIH.
**Remarque** : Un résultat positif doit toujours être confirmé.

# 2. Responsabilités

| Fonction | Activités |
|---|---|
| Technicien de laboratoire | ▪ Prélève le sang<br>▪ Exécute le test<br>▪ Interprète le résultat<br>▪ Enregistre les résultats |

# 3. Procédures

## 3.1 Précautions

Tous les échantillons de sang sont potentiellement infectieux. Respectez les précautions universelles. METTEZ DES GANTS À USAGE UNIQUE PENDANT TOUTE LA PROCÉDURE **!**

## 3.2 Matériel et échantillons

### 3.6.1 Matériel fourni dans le kit et conservation

- Tests Determine HIV-1/2 recouvert d'antigènes VIH-1/2 recombinant et de peptides synthétiques.
  *Conservation* : entre 2 et 30°C.
  Remarque : Le tampon n'est pas inclus dans le kit. Commandez donc le tampon si vous utilisez le test sur sang total.
- Brochure explicative

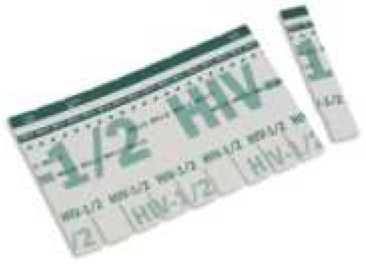


### 3.2.2 Matériel supplémentaire requis

- Tampon de fixation, 2,5 ml (à utiliser avec le sang total)
  *Conservation* : entre 2 et 30°C.
- Gants, non stérile, à usage unique
- Marqueur
- Micropipettes à 50 µl
- Embouts de pipette
- Minuterie
- Conteneur « bio-hazard »

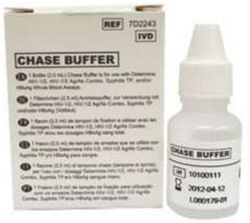


### 3.7.3 Echantillon à examiner

- Sang total prélevé sur EDTA: 50 µl
- *Conservation*:
  *Sang total* : Exécutez le test immédiatement ou réfrigérez à 2-8°C pendant 7 jours. NE CONGELER pas les échantillons de sang total. Congélations et décongélations successives peuvent affecter les résultats du test.
  Remarque : Laissez revenir les échantillons à température ambiante avant d'exécuter le test.

## 3.8 Mode opératoire

### 3.7.1 Contrôle de qualité interne

Une ligne de contrôle « C » est inclue dans le système afin de valider le test. Le résultat est invalide si la ligne de contrôle n'apparait pas à la fin du test.

### 3.7.2 Procédure

69. Laissez revenir tous les réactifs et échantillons à température ambiante.
70. Mettez des gants à usage unique.
71. Vérifiez la date de péremption. N'utiliser jamais un test après sa date de péremption.
72. Ouvrez l'emballage et enlevez le carton de 10 tests.
73. Vérifiez la date de péremption sur les tests. Détachez le nombre de tests nécessaire du carton de 10 tests, en commençant par la droite afin de préserver le numéro de lot et la date de péremption sur la gauche du carton.
74. Refermez l'emballage immédiatement.
75. Identifiez le test avec le numéro du patient et notez la date.
76. Enlevez la protection plastique de chaque test
77. Distribuez avec une micropipette 50 µl d'échantillon sur la zone de dépôt de l'échantillon (flèches).
78. Pour **le sang total** :
    - 78.1 Attendez une minute
    - 78.2 Ajoutez une goutte de tampon de fixation sur la zone de dépôt de l'échantillon
79. Lancez la minuterie (15 minutes).
80. Lisez le résultat après 15 minutes (maximum 60 minutes) (Voir « 3.4 interprétation du test »).
81. Prenez une photo du résultat.
82. Notez dans le CRF laboratoire les résultats du test, y compris l'intensité de la bande du test, le N° de lot du Kit et le N° de la photo.

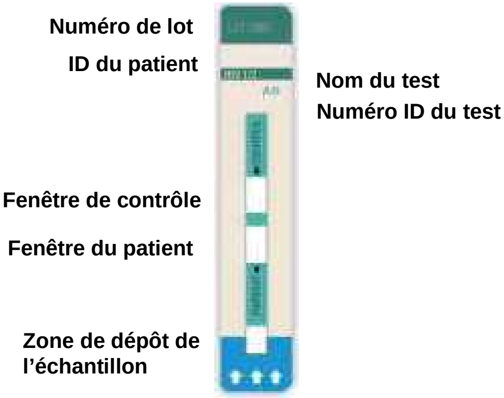


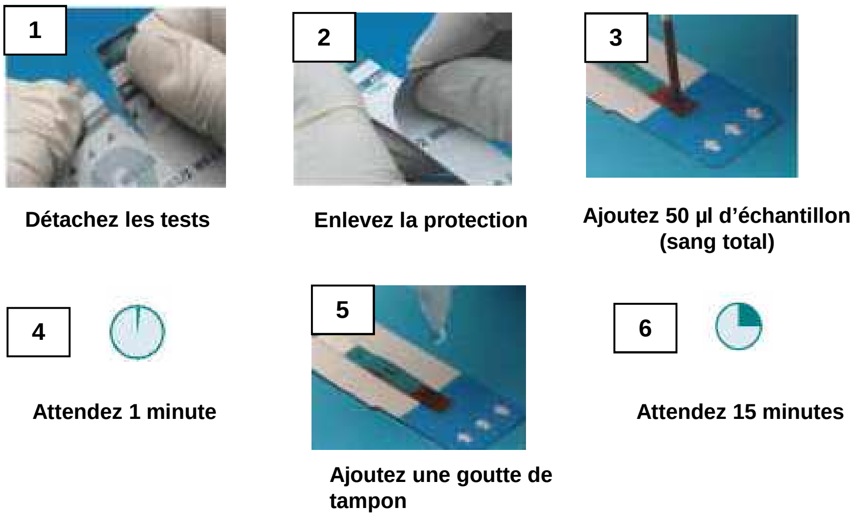


## 3.8 Interprétation du test

N'INTERPRÉTEZ PAS le test après plus de 60 minutes !

- **Négatif** – 1 ligne rouge
  Une seule ligne rouge dans la fenêtre de contrôle (annotée « Control »).
- **Positif** – 2 lignes rouges
  Deux lignes rouges : 1 dans la fenêtre de contrôle (annotée « Control ») et 1 dans la fenêtre du patient (annotée « Patient ») .
  L'intensité de la couleur et l'ordre de l'apparition des lignes n'est pas important. Toute couleur rouge dans la zone de test est interprétée comme un résultat positif.

- **Invalide** – pas de ligne rouge de contrôle
  L'absence d'une ligne rouge dans la fenêtre de contrôle (annotée « Control ») indique que le test s'est détérioré ou que la procédure n'a pas été correctement suivie.
  RÉPÉTEZ LE TEST !

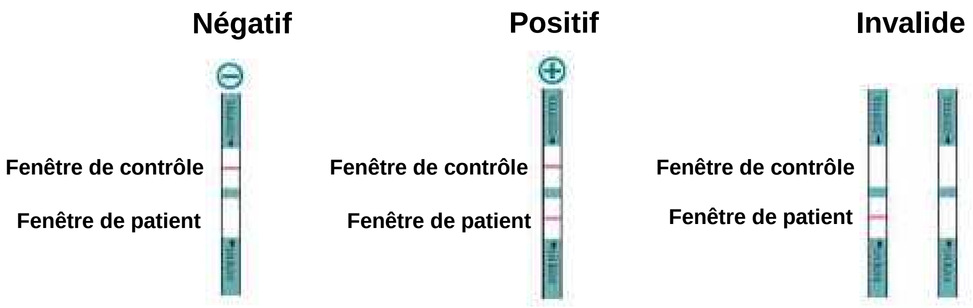


| Résultat | Conduite à tenir |
|---|---|
| Négatif | Rendez le résultat négatif. |
| Positif | Voir l'organigramme des tests VIH. Confirmez la positivité avec deux tests en parallèle : Unigold et DoubleCheckGold ! |
| Résultat invalide | Répétez le test. |

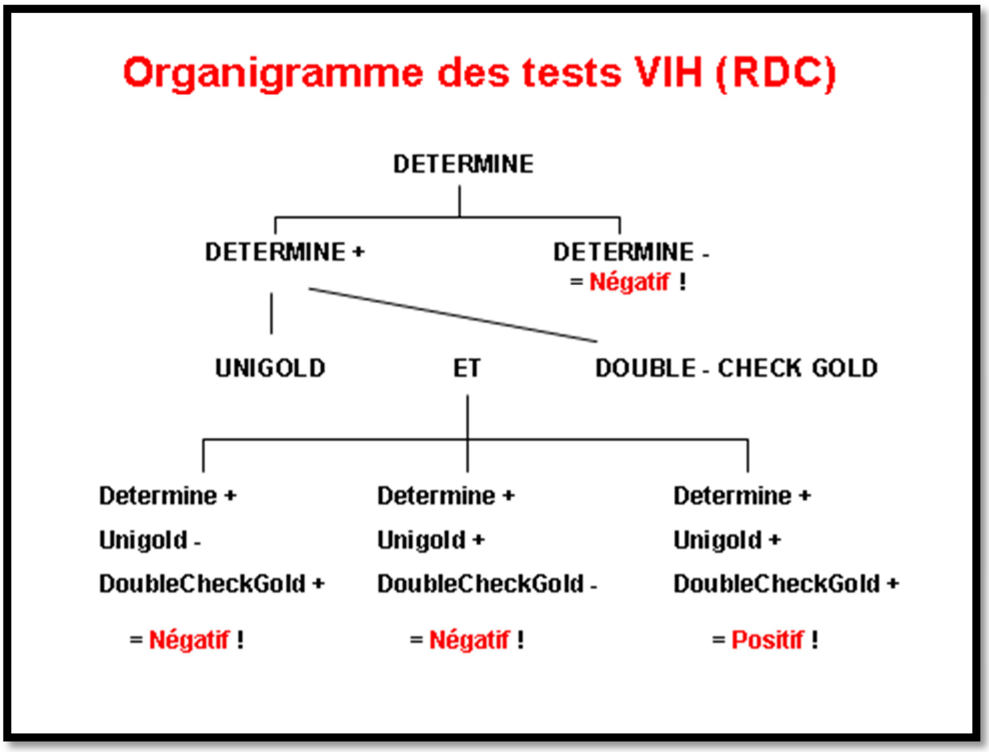


### 3.9 Déchets, nettoyage

- Jetez les tests utilisés dans un conteneur « bio-hazard ».

## 4. Enregistrements et archives

| Appendices & formulaires à compléter | |
|---|---|
| **Numéro** | **Titre** |
| 1 | CRF laboratoire |

## 5. Histoire de document

| **Révision** | |
|---|---|
| | Version initiale |

| **Nom et fonction** | **Date** | **Signature** |
|---|---|---|
| *Auteur* | | |
| Barbara Barbé | 05/06/2012 | |
| *Révisé par* | | |
| Philippe Gillet | 02/07/2012 | |
| *Approuvé par* | | |
| Veerle Lejon | 12/07/2012 | |

| | |
|---|---|
| | **SOP titre :** Uni-Gold HIV |
| | **Projet/Étude :** NIDIAG : Evaluation de tests rapides en association avec des prédicteurs cliniques et de laboratoire pour le diagnostic de maladies tropicales négligées dans des patients se présentant avec des troubles neurologiques dans les hôpitaux ruraux au Bandundu, République Démocratique du Congo. |

# 1. Domaine et application

Ce document fournit Les instructions pour l'exécution du test rapide immuno-chromotgraphique Uni-Gold HIV. Ce test détecte les anticorps anti-VIH-1 et anti-VIH-2 dans le sérum, le plasma ou le sang total et constitue ainsi une aide sur le diagnostic de VIH. **Remarque** : Un résultat positif doit toujours être confirmé.

# 2. Responsabilités

| Fonction | Activités |
|---|---|
| Technicien de laboratoire | ▪ Prélève le sang<br>▪ Exécute le test<br>▪ Interprète le résultat<br>▪ Enregistre les résultats |

# 3. Procédures

## 3.1 Précautions

Tous les échantillons de sang sont potentiellement infectieux. Respectez les précautions universelles. METTEZ DES GANTS À USAGE UNIQUE PENDANT TOUTE LA PROCÉDURE **!**

## 3.2 Matériel et échantillons

### 3.2.1 Matériel fourni dans le kit et conservation

- <u>Cassette Uni-Gold HIV</u> :
  Chaque test contient :
  - L'or conjugué : protéines du VIH recombinantes – colloïde – or
  - Ligne de test : protéines du VIH recombinantes immobilisées
  - Ligne du contrôle

  *Conservation* : entre 2 et 27°C. NE PAS CONGELER !
  Utilisez les tests immédiatement après l'ouverture du sachet.
  NE PAS UTILISER si le sachet est endommagé.
- <u>Réactif de lavage, 2 ml</u> (pour sérum, plasma et sang total)
  *Conservation* : entre 2 et 27°C. NE PAS CONGELER !
  <u>Remarque</u> : Utilisez seulement le réactif de lavage inclus pour les tests appartenant à ce kit.
- Pipettes à usage unique
- Brochure explicative

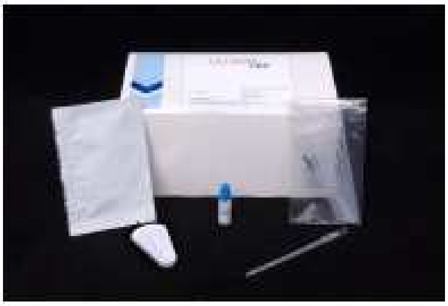

### 3.2.2 Matériel supplémentaire requis

- Gants, non stérile, à usage unique
- Marqueur
- Minuterie
- Conteneur « bio-hazard »

### 3.2.3 Echantillon à examiner

- Sang total : ~ 60 µl
- *Conservation*:

- *Sang total* : Exécutez le test immédiatement ou réfrigérez à 2-8°C pendant 3 jours. NE CONGELER pas les échantillons de sang total.

Remarque : Laissez revenir les échantillons à température ambiante avant d’exécuter le test. Congélations et décongélations successives peuvent affecter les résultats du test.

## 3.3 Mode opératoire

### 3.3.1 Contrôle de qualité interne

Une ligne de contrôle « C » est inclue dans le système afin de valider le test. Le résultat est invalide si la ligne de contrôle n’apparait pas à la fin du test.

### 3.3.2 Procédure

83. Laissez revenir tous les réactifs et échantillons à température ambiante.
84. Mettez des gants à usage unique.
85. Vérifiez la date de péremption. . N’utiliser jamais un test après sa date de péremption.
86. Ouvrez l’emballage et enlevez le nombre de tests nécessaire.
87. Refermez l’emballage immédiatement.
88. Identifiez le test avec le numéro du patient et notez la date.
89. Remplissez une des pipettes à usage unique fournies avec l’échantillon.
90. Distribuez avec cette pipette 2 gouttes (~ 60 µl) de sang total dans le puits d’échantillon (« Sample »).
91. Ajoutez 2 gouttes de réactif de lavage (~ 60 µl) dans le puits d’échantillon (« Sample »).
92. Lancez la minuterie (10 minutes)
93. Lisez le résultat après 10 minutes (au maximum 20 minutes). (Voir « 3.4 interprétation du test »).
94. Prenez une photo du résultat.
95. Notez dans le CRF laboratoire les résultats du test, y compris l’intensité de la bande du test, le N° de lot du Kit et le N° de la photo.

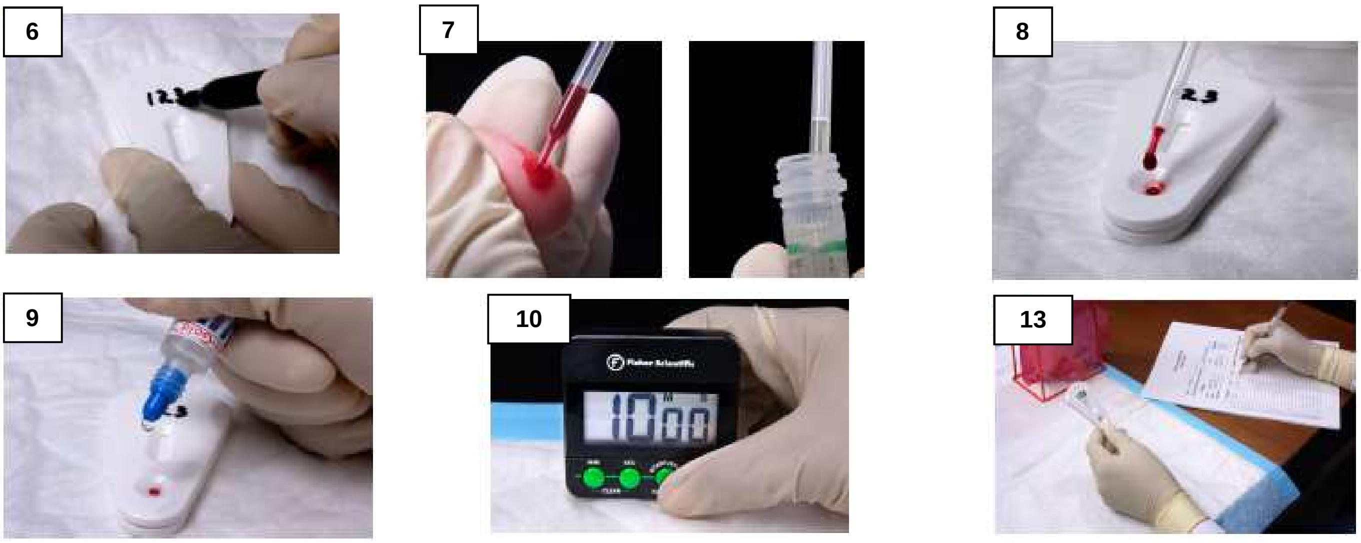

## 3.4 Interprétation du test

N'INTERPRÉTEZ PAS le test après plus de 20 minutes !

- **Négatif** – 1 ligne rouge
  Une seule ligne rouge dans la zone de contrôle « C ».

- **Positif** – 2 lignes rouges
  Deux lignes rouges : 1 dans la zone de contrôle « C » et 1 dans la zone de test « T ». L'intensité de la couleur et l'ordre de l'apparition des lignes n'est pas important. Toute couleur rouge dans la zone de test est interprétée comme un résultat positif.

- **Invalide** – pas de ligne rouge de contrôle
  L'absence d'une ligne rouge dans la zone de contrôle « Control » indique que le test s'est détérioré ou que la procédure n'a pas été suive correctement. RÉPÉTEZ LE TEST !

Remarque : Rarement, la ligne de contrôle ou la ligne de test peut apparaître "brisée". Répétez le test pour cet échantillon avec un autre test Uni-Gold HIV.

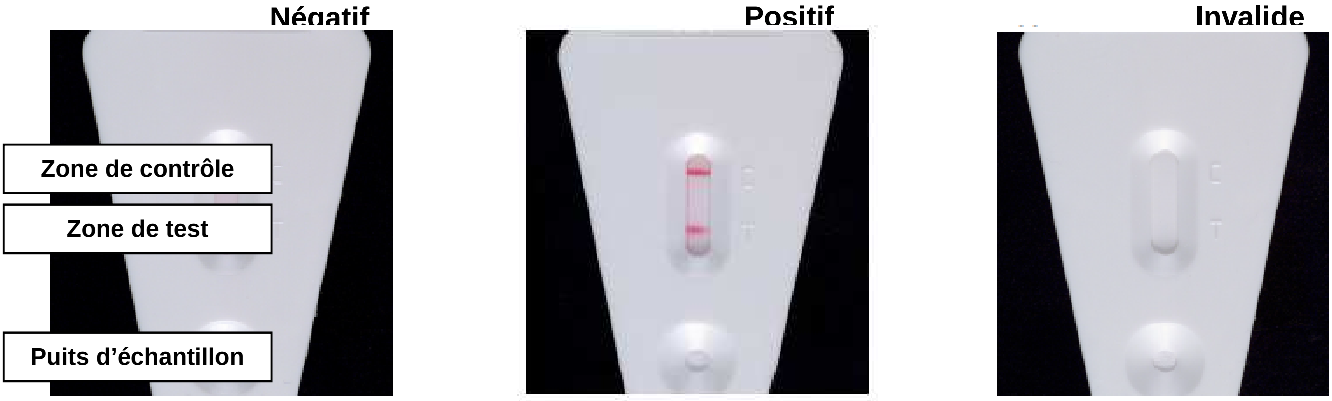


| Résultat | Conduite à tenir |
|---|---|
| Négatif | Rendez le résultat négatif. |
| Positif | Voir l'organigramme des tests VIH.<br>Confirmez la positivité avec le résultat du TDR DoubleCheckGold ! |
| Résultat invalide | Répétez le test. |

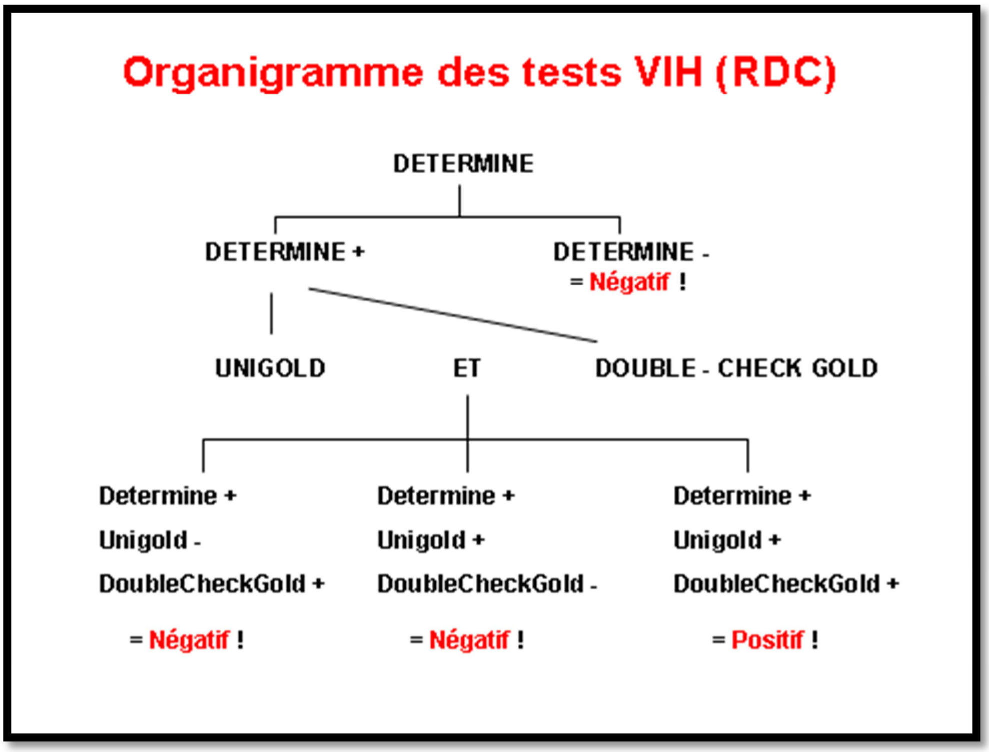


### 3.5 Déchets, nettoyage

- Jetez les cassettes utilisées dans un conteneur « bio-hazard ».
- Jetez l'excès du réactif de lavage (après avoir utilisé le kit complètement) dans un conteneur « bio-hazard ».

## 4. Enregistrements et archives

| **Appendices & formulaires à compléter** | |
|---|---|
| **Numéro** | **Titre** |
| 1 | CRF laboratoire |

## 5. Histoire de document

| **Révision** | |
|---|---|
| | Version initiale |

| **Nom et fonction** | **Date** | **Signature** |
|---|---|---|
| *Auteur* | | |
| Barbara Barbé | 05/06/2012 | |
| *Révisé par* | | |
| Philippe Gillet | 02/07/2012 | |
| *Approuvé par* | | |
| Veerle Lejon | 12/07/2012 | |

| | |
|---|---|
| nidiag Better Diagnosis of Neglected Infectious Diseases | **SOP titre :** DoubleCheckGold HIV 1&2 |
| | **Projet/Étude** : NIDIAG : Evaluation de tests rapides en association avec des prédicteurs cliniques et de laboratoire pour le diagnostic de maladies tropicales négligées dans des patients se présentant avec des troubles neurologiques dans les hôpitaux ruraux au Bandundu, République Démocratique du Congo. |

# 1. Domaine et application

Ce document fournit les instructions pour l'exécution du test rapide immuno-chromatographique DoubleCheckGold HIV 1&2. Ce test détecte des anticorps anti-VIH-1 (type O y compris) et anti-VIH-2 dans le sang total et constitue ainsi une aide sur le diagnostic de VIH.
**Remarque** : Un résultat positif doit toujours être confirmé.

# 2. Responsabilités

| Fonction | Activités |
|---|---|
| Technicien de laboratoire | ▪ Prélève le sang<br>▪ Exécute le test<br>▪ Interprète le résultat<br>▪ Enregistre les résultats |

# 3. Procédures

## 3.1 Précautions

Tous les échantillons de sang sont potentiellement infectieux. Respectez les précautions universelles. PORTEZ DES GANTS À USAGE UNIQUE PENDANT TOUTE LA PROCÉDURE **!**

## 3.2 Matériel et échantillons

### 3.5.1 Matériel fourni dans le kit et conservation

- Cassettes DoubleCheckGold HIV 1&2:
  Chaque test contient :
  - conjugué : conjugué de protéine du VIH – colloïde – or
  - Ligne de test : protéines du VIH recombinantes immobilisées
  - Ligne du contrôle : réactif de liaison d'anticorps

  *Conservation* : entre 2 et 30°C.
  Utilisez les tests immédiatement après l'ouverture du sachet.
  N'UTILISEZ PAS LE TEST si le sachet est endommagé.
- Réactif de lavage, 10 ml
  *Conservation* : entre 2 et 30°C.
  Remarque : Utilisez uniquement le réactif de lavage inclus pour les tests appartenant à ce kit.
- Dispositifs applicateur de sang total:
  Ces coton-tiges prétraités sont utilisés pour collecter et appliquer le sang total sur le puits échantillon de la cassette de test.
- Brochure explicative.

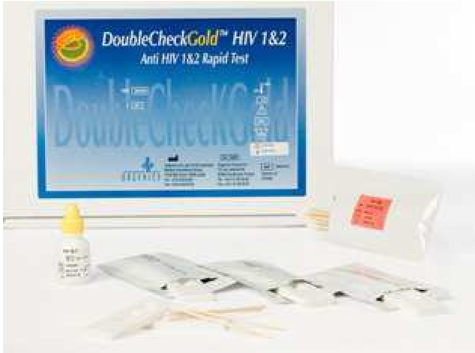


### 3.2.2 Matériel supplémentaire requis

- Gants, non stérile, à usage unique
- Micropipette de 50 µl
- Embouts pour micropipette
- Marqueur

- Minuterie
- Conteneur « bio-hazard »

### 3.5.1 Echantillon à examiner

- Tube EDTA de sang total : au minimum 50 µl
  - *Conservation*: Exécutez le test immédiatement ou réfrigérez l'échantillon à 2-8°C pour 3 jours maximum.
  - Remarques : Laissez revenir les échantillons à température ambiante avant d'exécuter le test.

## 3.6 Mode opératoire

### 3.5.1 Contrôle de qualité interne

Une ligne de contrôle « C » est inclue dans le système afin de valider le test. Le résultat est invalide si la ligne de contrôle n'apparait pas à la fin du test.

### 3.5.2 Procédure

11. Laissez revenir tous les réactifs et échantillons à température ambiante (15 – 30°C).
12. Mettez des gants à usage unique.
13. Vérifiez la date de péremption. N'utiliser jamais un test après sa date de péremption.
14. Ouvrez l'emballage et enlevez le cassette.
15. Identifiez le test avec le numéro du patient et la date.
16. Mélangez doucement la tube de sang total sur EDTA.
17. Trempez le coton tige du dispositif applicateur à fond dans le tube de sang total sur EDTA et faites le tournoyer.
    Remarque : Ne laissez pas tremper le dispositif applicateur dans le sang pendant plus d'1 minute.
18. Enlevez le dispositif applicateur du tube de sang total sur EDTA.
19. APRES 10 SECONDES, appliquez le tige du dispositif applicateur en appuyant DOUCEMENT sur le puits échantillon de la cassette de test. La quantité suffisante de sang total est transférée sur la cassette.
    Remarque : Le transfert de sang est réussi quand une tache rouge est visible sur le puits échantillon après avoir enlevé le coton-tige.
20. Jetez le dispositif applicateur utilisé dans un conteneur « bio-hazard ».

21. Ajoutez 3 gouttes de réactif de lavage (~ 100 µl) dans le puits échantillon.
22. Lancez la minuterie (15 minutes)
23. Lisez le résultat après 15 minutes (Voir « 3.3.4 interprétation du test »).
24. Prenez une photo du résultat.
25. Notez dans le CRF laboratoire les résultats du test, y compris l'intensité de la bande du test, le N° de lot du Kit et le N° de la photo.

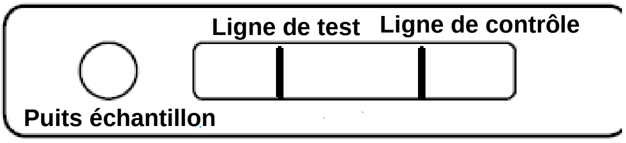


## 3.6 Interprétation du test

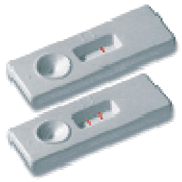

N'INTERPRÉTEZ PAS le test après plus de 25 minutes !

- **Négatif** – 1 ligne rouge
  Une seule ligne rouge dans la zone de contrôle « C ».

- **Positif** – 2 lignes rouges
  Deux lignes rouges : 1 dans la zone de contrôle « C » et 1 dans la zone de test « T ».
  L'intensité de la couleur et l'ordre de l'apparition des lignes n'est pas important. Toute couleur rouge dans la zone de test est interprétée comme un résultat positif.

- **Invalide** – pas de ligne rouge de contrôle
  L'absence d'une ligne rouge dans la zone de contrôle « C » indique que le test s'est détérioré ou que la procédure n'a pas été correctement suivie. RÉPÉTEZ LE TEST !

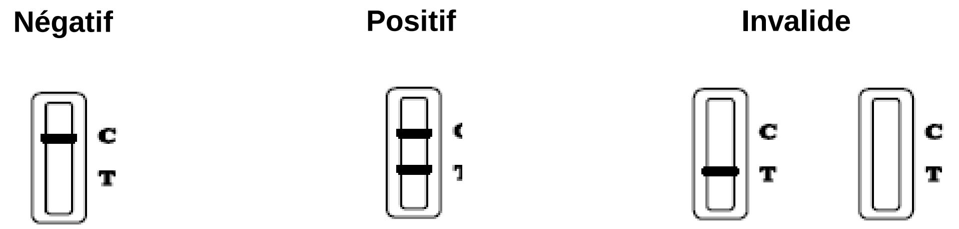


| Résultat | Conduite à tenir |
|---|---|
| Négatif | Rendez le résultat négatif. |
| Positif | Voir l'organigramme des tests VIH.<br>Confirmez la positivité avec le résultat du TDR Uni-Gold ! |
| Résultat invalide | Répétez le test. |

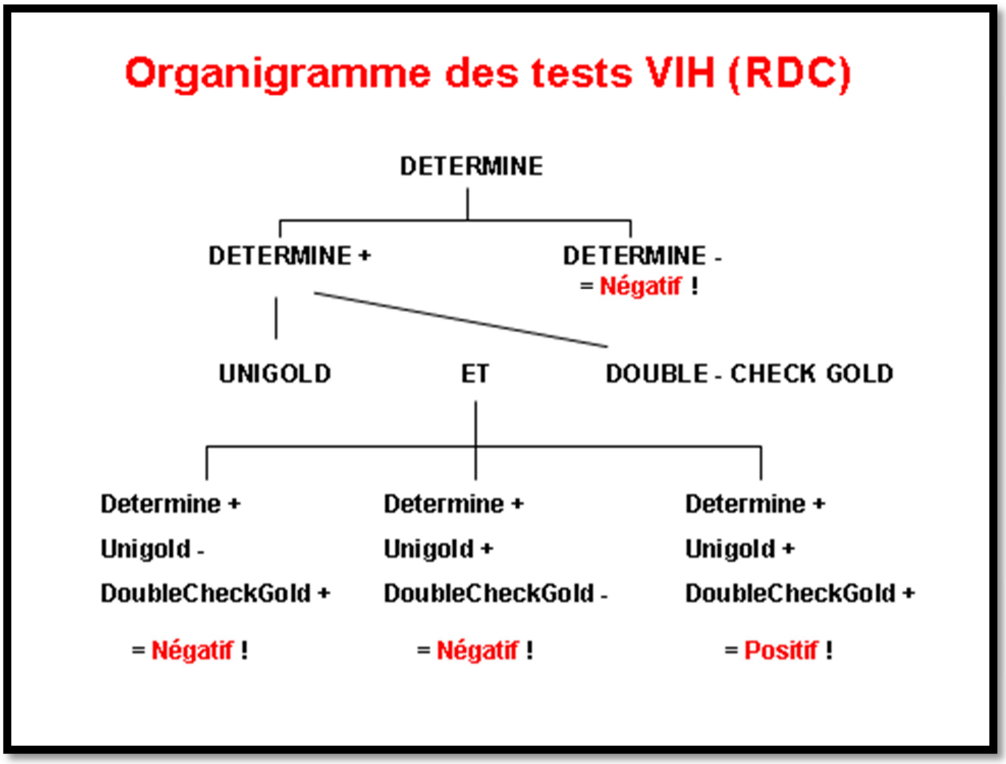

### 3.7 Déchets, nettoyage

- Jetez les cassettes et les dispositifs applicateurs utilisés dans un conteneur « bio-hazard ».
- Jetez l'excès du réactif de lavage (après avoir utilisé le kit complètement) dans un conteneur « bio-hazard ».

## 4. Registres et archives

| **Appendices & formulaires à compléter** | |
|---|---|
| **Numéro** | **Titre** |
| 1 | CRF laboratoire |

## 5. Historique du document

| **Révision** | |
|---|---|
| SOP-WP2-LAB-34-V01-12Jul2012 | Version initiale |
| SOP-WP2-LAB-34-V02-18Sep2012 | Volume de sang changé à 50 µl |
| SOP-WP2-LBA-34-V03-26Dec2012 | Changement de l'application de l'échantillon (avec dispositif applicateur) |

| **Nom et fonction** | **Date** | **Signature** |
|---|---|---|
| *Auteur* | | |
| Barbara Barbé | 26/12/2012 | |
| *Révisé par* | | |
| Philippe Gillet | 28/12/2012 | |
| *Approuvé par* | | |
| Emilie Alirol | 02/01/2013 | |

| | |
|---|---|
| | **SOP titre :** Test diagnostic rapide (TDR) du paludisme: SD BIOLINE Ag Pf / Pan (SD 05FK60) |
| | **Projet/Étude :** Evaluation de tests rapides en association avec des prédicteurs cliniques et de laboratoire pour le diagnostic de maladies tropicales négligées chez les patients se présentant avec des troubles neurologiques dans les hôpitaux ruraux au Bandundu, République Démocratique du Congo. |

## Domaine et application

Ce document fournit les instructions pour exécuter le test diagnostic rapide du paludisme SD 05FK60. Ce test rapide détecte le présence d'antigènes spécifiques à *Plasmodium falciparum* HRP-II (Protéine II, riche en histidine) et à toutes les espèces de *Plasmodium* (Pan-pLDH : Parasite lactate déhydrogénase) : *P. falciparum, P. vivax, P. ovale* et *P. malariae*. Ce test apporte une aide pour le diagnostic du paludisme. Attention : Ce test ne permet pas toujours de différencier une infection à *Plasmodium falciparum* d'une infection mixte (*P. falciparum* associé à une ou plusieurs autres espèces de Plasmodium).

## 2. Responsabilités

| Fonction | Activités |
|---|---|
| Technicien de laboratoire | ▪ Prélève le sang<br>▪ Exécute le test<br>▪ Interprète le résultat<br>▪ Enregistre les résultats |

## 3. Procédures

### 3.1 Précautions

- Tous les échantillons de sang sont potentiellement infectieux. Respectez les précautions universelles. METTEZ DES GANTS À USAGE UNIQUE PENDANT TOUTE LA PROCÉDURE !

### 3.2 Matériel et échantillons

#### 3.2.1 Matériel fourni dans le kit et conservation

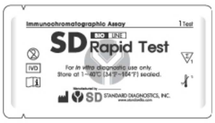


- La cassette SD BIOLINE Ag Pf / Pan :
  Une bandelette de test comprenant :
  - o Le conjugué : Anticorps monoclonaux de souris spécifiques à la P.f. HRP-2 – or colloïdal
    Anticorps polyclonaux de souris spécifiques à la pan pLDH - or colloïdal
  - o Ligne de test P.f. : Anticorps monoclonaux de souris spécifiques à P.f. HRP-II
  - o Ligne de test Pan : Anticorps polyclonaux de souris spécifiques à la pan-pLDH
  - o Ligne du contrôle : Anticorps de chèvre anti-anticorps de souris

  *Conservation* : à température ambiante (1 - 40°C). NE PAS REFRIGÉRER !

La cassette est sensible à l'humidité et la chaleur. Utilisez les tests immédiatement après ouverture du sachet. N'UTILISEZ PAS le test si le sachet est endommagé.

.

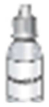

- Diluant (tampon) :
  Une bouteille contient :
  - o Albumine bovine
  - o Triton-X 100

  *Conservation* : à température ambiante (1 - 40°C). NE PAS REFRIGÉRER !

- Remarque : Utilisez uniquement le tampon inclus pour tous les tests appartenant au même kit.
- Brochure explicative

### 3.2.2 Matériel supplémentaire requis

- Gants non stériles, à usage unique
- Minuterie
- Marqueur (crayon, stylo)
- Micropipette de 5 µl
- Des embouts pour micropipette
- Conteneur « bio-hazard »

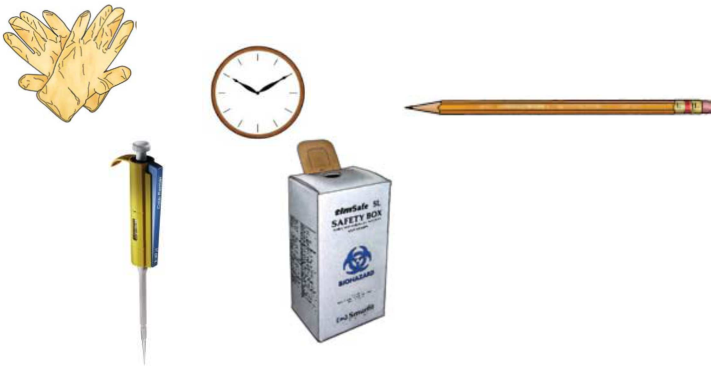

### 3.2.3 Echantillon à examiner

- Sang veineux prélevé sur EDTA : 5 µl
- *Conservation*: Exécutez le test immédiatement ou réfrigérez l'échantillon à 2-8°C pour 3 jours maximum . Pour des périodes plus longues, congelez l'échantillon à -20°C.
- Remarque : Laissez revenir les échantillons à température ambiante avant d'exécuter le test.

## 3.3 Mode opératoire

### 3.3.1 Contrôle de qualité interne

Une ligne de contrôle « C » est inclue dans le système afin d'assurer la validité du test. Si la ligne de contrôle n'apparait pas à la fin du test, le résultat n'est pas valide.

### 3.3.2 Procédure

**1**. Laissez revenir l'échantillon et le TDR à température ambiante

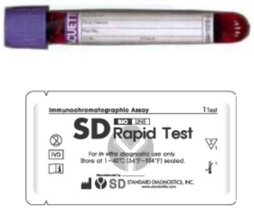


**2**. Vérifiez la date de péremption sur le sachet (NE PAS UTILISER au-delà de la date d'expiration!)

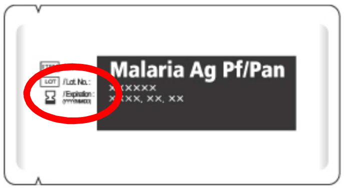


**3**. Mettez les gants

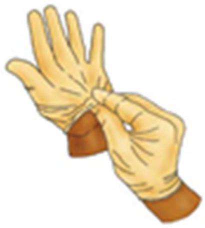

---

**4.** Ouvrez le paquet

a) Prenez la cassette

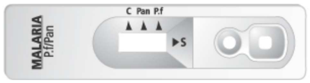


b) Vérifiez la couleur du gel de silice

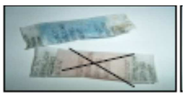

En cas de changement de couleur, (bleu qui est devenu rose) utilisez un autre test !

**5**. Ecrivez la date et le numéro du patient sur la cassette

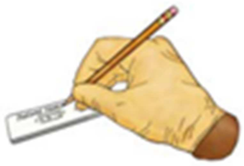

**6**. Aspirez 5µl de sang EDTA à l'aide d'un micropipette Utilisez un embout neuf pour chaque échantillon)

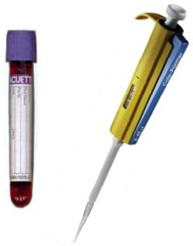

**7**. Déposez les 5 µl de sang dans le puits rond

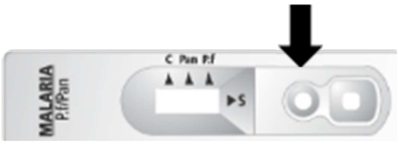


**8**. Ajoutez 4 gouttes de tampon dans le puits carré

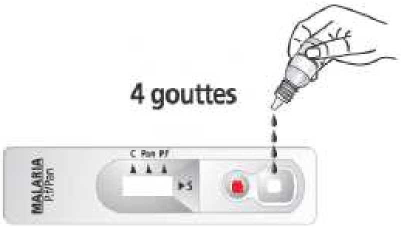


**9**. Lancez la minuterie (15 minutes) Attendez 15 minutes avant de lire le résultat. Si négatif, répéter la lecture à 30 minutes ! (NE LISEZ PAS après 30 minutes)

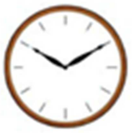

10. Prenez une photo du résultat.

11. Notez dans le CRF laboratoire les résultats du test, y compris l'intensité des bandes du test, le N° de lot du Kit et le N° de la photo

## 3.4 Interprétation du test

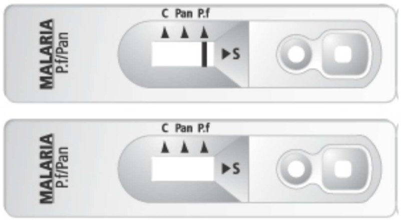


Ligne de contrôle « C » absente

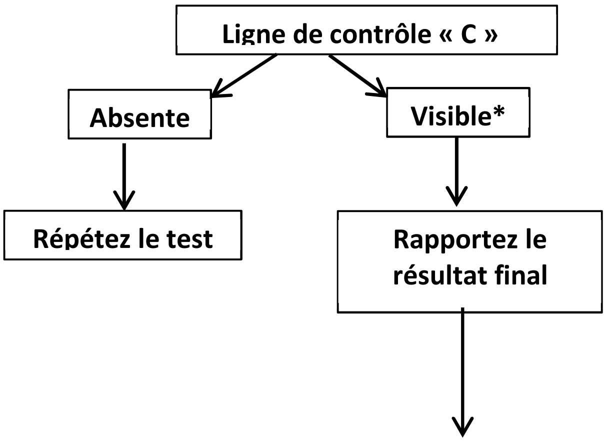


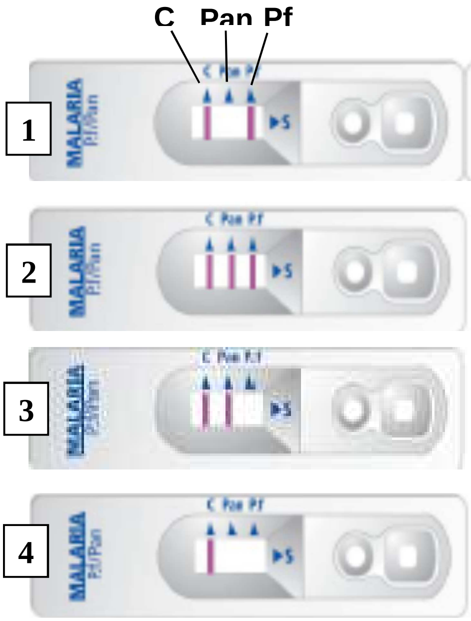


| | Ligne de contrôle « C » visible | Ligne de Pan visible | Ligne de Pf visible | Résultat final à rapporter |
|---|---|---|---|---|
| 1 | Oui | Non | Oui | Infection à *P. falciparum* |
| 2 | Oui | Oui | Oui | *Infection à P. falciparum* infection mixte possible (*P. falciparum* avec un *Plasmodium* non-*falciparum*) |
| 3 | Oui | Oui | Non | Infection à *Plasmodium* non-*falciparum* |
| 4 | Oui | Non | Non | Négatif |

*** Quel que soit l'intensité de la bande**

### Déchets, nettoyage

- Jetez les cassettes utilisées dans un conteneur « bio-hazard ».
- Jetez l’excès du diluant (après avoir utilisés tous les tests du kit) dans un conteneur « bio-hazard ».

## 4. Enregistrements et archives

| **Appendices & formulaires à completer** | |
|---|---|
| **Numéro** | **Titre** |
| 1 | CRF laboratoire |

## 5. Références

- Instruction du producteur, version 05FK60-FR-14 2013.04

## 6. Histoire de document

| **Révision** | |
|---|---|
| SOP-WP2-LAB-35-V01-12Jul2012 | Version initiale |
| SOP-WP2-LAB-35-V02-11Apr2014 | Changement de temps de lecture |

| **Nom et fonction** | **Date** | **Signature** |
|---|---|---|
| *Auteur* | | |
| Barbara Barbé | 11/04/2014 | |
| *Révisé par* | | |
| | | |
| *Approuvé par* | | |
| Ninon Horié | 11/04/2014 | |

| | |
|---|---|
| nidiag Better Diagnosis of Neglected Infectious Diseases | **SOP titre:** Le prélèvement de l'urine à mi-jet |
| | **Projet/Étude :** Evaluation de tests rapides en association avec des prédicteurs cliniques et de laboratoire pour le diagnostic de maladies tropicales négligées chez les patients se présentant avec des troubles neurologiques dans les hôpitaux ruraux au Bandundu, République Démocratique du Congo. |

## 1. Domaine et application

Ce document fournit les instructions pour prélever l'urine à mi-jet

## 2. Responsabilités

| Fonction | Activités |
|---|---|
| Technicien de laboratoire | ▪ Prélève l'urine<br>▪ Suit la procédure |

## 3. Procédures

### 3.1 Précautions

- Tous les échantillons d'urine sont potentiellement infectieux. Respectez les précautions universelles. PORTEZ DES GANTS À USAGE UNIQUE PENDANT TOUTE LA PROCÉDURE **!**

### 3.2 Matériel requis

- Récipient pour urine en plastique, à usage unique
- Gants, non stérile, à usage unique
- Marqueur

### 3.3 Procédure

- Mettez des gants à usage unique
- Identifiez le récipient avec le numéro du patient et notez la date et l'heure.
- Donnez le récipient au patient.
- Expliquez au patient comment prélever une urine à mi-jet.
- Laissez le patient prélever l'urine dans les toilettes locales:
    - o au minimum 50 ml
    - o à mi-jet
- Utilisez l'urine au maximum 4 heures après le prélèvement.

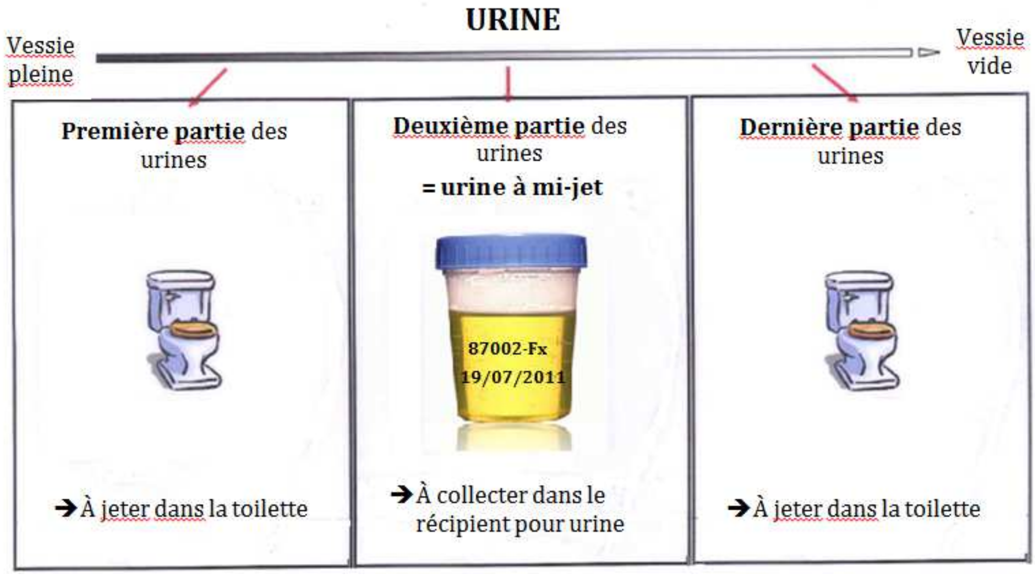

## 4. Registres et archives

| **Appendices & formulaires à compléter** | |
|---|---|
| **Numéro** | **Titre** |
| | Pas nécessaire |

## 5. Historique du document

| **Révision** | |
|---|---|
| | Version initiale |

| **Nom et fonction** | **Date** | **Signature** |
|---|---|---|
| *Auteur* | | |
| Barbara Barbé | 19/07/2012 | |
| *Révisé par* | | |
| Philippe Gillet | 10/08/2012 | |
| *Approuvé par* | | |
| Veerle Lejon | 13/08/2012 | |

| | |
|---|---|
| | **SOP titre :** Liste des tests NIDIAG-Neuro |
| | **Projet/Étude :** Evaluation de tests rapides en association avec des prédicteurs cliniques et de laboratoire pour le diagnostic de maladies tropicales négligées chez les patients se présentant avec des troubles neurologiques dans les hôpitaux ruraux au Bandundu, République Démocratique du Congo. |

## 1. Domaine et application

Cette procédure décrit les échantillons à prélever, les tests à réaliser et l'ordre des tests dans l'étude NIDIAG, syndrome neurologique.

## 2. Responsabilités

| Fonction | Activités |
|---|---|
| Technicien de laboratoire<br>Infirmier | ▪ Suit la procédure<br>▪ Réalise les prélèvements<br>▪ Analyse les échantillons comme décrits dans les SOPs |
| Médecins | • Demande les prélèvements et les analyses<br>• Supervise les prélèvements |

## 3. Procédures

| ORDRE £ | PRELEVEMENT * | TESTS A REALISER (ou actions à faire) | PRIORITE pour la réalisation des tests $ | TESTS SUPPLEMENTAIRES | ACTIONS SUPPLEMENTAIRES |
|---|---|---|---|---|---|
| **1.0°** | **Sang total (10 ml)** | Hémoculture ATTENTION NON FAIT POUR LES ENFANT DE MOIS DE 14 ANS | Immédiatement | | Incubez et envoyez à l'INRB 1 x/semaine |
| | | | | | |
| **1.1°** | **Sérum (7 ml) (5 ml)** | CENTRIFUGATION | | SEPARATION sérum et mise dans 1 tube et 2 cryotube (bouchons incolores) | Cryotube en azote liquide, envoyez à l'INRB 1x/3 semaines |
| | ATTENTION, POUR LES ENFANTS DE MOINS DE 14 ANS, LE TUBE SERUM SERA PRIS EN 2.01) | RPR SYPHILIS Sérum | | Si RPR SYPHILIS positif, titration | |
| | | RDT Cr Ag LFA (qualitatif) | | | |
| | | | | si CATT SANG TOTAL +: CATT dilution | |
| | | GLYCEMIE (reflotron) | | | |
| | | FONCTION RENALE: créatinine | | | |
| | | FONCTION HEPATIQUE: GPT | | | |
| | | | | | |

| ORDRE [£] | PRELEVEMENT * | TESTS A REALISER (ou actions à faire) | PRIORITE pour la réalisation des tests [$] | TESTS SUPPLEMENTAIRES | ACTIONS SUPPLEMENTAIRES |
|---|---|---|---|---|---|
| **2.0** | **Sang total (10 ml) (4ml)** | Hémoculture | Immédiatement 1 | | Incubez et envoyez à l'INRB 1 x/semaine |
| | | | | | |
| **2.01** | **(sérum pour le enfant 5 ml)** | | | | |
| | | | | | |
| **2.1** | **Sang héparine (5 ml) (5 ml)** | Mini-colonne | Immédiatement 2 | | |
| | | (si pas sur reflotron : glycémie) | | | |
| | | CATT sang total | | | |
| | | RDT HAT CORIS | | | |
| | | RDT HAT FIND | | | |
| | | CENTRIFUGATION | | SEPARATION PLASMA HEPARINE ET MISE EN CRYOTUBES (bouchons bleus) | Cryotube en azote liquide, envoyez à l'INRB 1x/3 semaines |
| | | | | | |
| **2.2** | **Sang EDTA (7 ml) (5ml)** | GE (frottis) colorés au Giemsa | | | |
| | | Hémoglobine (hemocue) | | | |
| | | Numération GB + différentiation (hemocue diff) | | | |
| | | RDT HIV DETERMINE | Immédiatement 3 | SI DETERMINE + : UNIGOLD ET DOUBLE CHECK | |
| | | | | SI HIV+ confirmé : CD4 | |
| | | RDT SYPHILIS SD BIOLINE | | | |
| | | RDT MALARIA SD BIOLINE 60 | | | |
| | | RDT MALARIA CARESTART Pldh | | | |
| | | Mise en cryotubes d'une partie du sang complet EDTA (bouchons rouges) puis CENTRIFUGATION | | SEPARATION PLASMA EDTA ET MISE EN CRYOTUBES (bouchons violets) | Cryotube en azote liquide, envoyez à l'INRB 1x/3 semaines |

| ORDRE [£] | PRELEVEMENT * | TESTS A REALISER (ou actions à faire) | PRIORITE pour la réalisation des tests [$] | TESTS SUPPLEMENTAIRES | ACTIONS SUPPLEMENTAIRES |
|---|---|---|---|---|---|
| **3.0** | **Urine (30 ml, min 10 ml)** | Cr Ag LFA (qualitatif) | | MISE EN CRYOTUBES (bouchons jaunes) | |
| | | | | | |
| **4.0** | **Palpation ganglions cervivaux** | Si ganglions typiques, ponction ganglionnaire | immédiatement 1 | | |
| | **Ponction ganglionnaire** | Recherche de tryp à frais | immédiatement 2 | | |

| ORDRE [£] | PRELEVEMENT * | TESTS A REALISER (ou actions à faire) | PRIORITE pour la réalisation des tests [$] | TESTS SUPPLEMENTAIRES | ACTIONS SUPPLEMENTAIRES |
|---|---|---|---|---|---|
| **5.0** | **LCR (12 ml) (6 ml)** | Aspect macroscopique du LCR | immédiatement 1 | | Incubez et envoyez à l'INRB 1 x/semaine |
| | | Culture bactAlert pédiatrique (2 ml) (1 ml) | immédiatement 2 | | Incubez et envoyez à l'INRB 1 x/semaine |
| | | Culture trans-Isolate (0,5 ml) (0,2 ml) | immédiatement 3 | | |
| | | Culture MGIT | immédiatement 4 | | Incubez sur place, suivez la positivité durant 5 semaines. Stérilisez et envoyez toutes les positifs et 10 % des négatifs à l'INRB (tranféré chez MSF pour GenExpert) |
| | | Numération GB (uriglass) | immédiatement 5 | | |
| | | Transvasez 4 ml (2 ml) du LCR dans un tube collecteur. Centrifugez le tube original et le tube collecteur | immédiatement 6 (sur tube le collecteur) | Simple centrigugation modifiée (MSC) sur 4 ml pour les adultes, sur 2 ml pour les enfants. Après lecture, décantez le surnageant dans un cryotube (bouchons verts) | Cryotube en azote liquide, envoyez à l'INRB 1x/3 semaines |
| | | | immédiatement 7 (sur le tube original) | Décantez le surnageant dans 1 tube à hémolyse et un 1 cryotube (bouchons verts) | Cryotube en azote liquide, envoyez à l'INRB 1x/3 semaines |
| | | | immédiatement 8 (sur le tube original) | culot : si HIV+ confirmé ET SI PLUS DE 5 GB, ENCRE DE CHINE | |
| | | | immédiatement 9 (sur le tube original) | culot: SI PLUS DE 5 GB : GIEMSA ET DIFF GB | |

| ORDRE £ | PRELEVEMENT * | TESTS A REALISER (ou actions à faire) | PRIORITE pour la réalisation des tests $ | TESTS SUPPLEMENTAIRES | ACTIONS SUPPLEMENTAIRES |
|---|---|---|---|---|---|
| | | | immédiatement 10 (sur le tube original) | culot: SI PLUS DE 5 GB : GRAM | |
| | | | immédiatement 11 (sur le tube original) | culot: SI PLUS DE 5 GB : ZIEHL | |
| | | | immédiatement 12 sur le surnageant | surnageant en tube à hémolyse : glucorachie ? | |
| | | | immédiatement 13 sur le surnageant | surnageant en tube à hémolyse: RDT Cr Ag LFA (qualitatif) | |
| | | | immédiatement 14 sur le surnageant | SI HIV + surnageant en tube à hémolyse chauffé: Cr Ag LAT (latex) (qualitatif et si + quantitatif) | |

**£ Les prélèvements devront être réalisés dans cet ordre chronologique. 1 prélèvement sanguin sur le bras gauche, 2 prélèvement sanguin sur le bras droit, 3 prélèvement d'urine, 4 ponction ganglionnaire, 5 ponction lombaire. Avant de passer au prélèvement suivant, les analyses prioritaires devront être réalisées (cf. priorité pour la réalisation des tests).**

*** Les prélèvements devront être réalisés dans cet ordre chronologique. EN ROUGE LES VOLUMES A PRELEVER POUR LES ENFANTS DE MOINS DE 14 ANS**

**° Pour les enfants de moins de 14 ans, 1 seule hémoculture est prélevée, sur un seul bras. Tous les prélèvements sanguins, y compris le tube pour sérum sont collectés en même temps (cf point 2.01)**

**$ Les tests prioritaires sont à faire dans cet ordre : soit immédiatement, soit par ordre chronologique (indiqué par un chiffre). Les autres tests peuvent être réalisés plus tard. Pour les patients qui sont inclus en fin d'après-midi ou durant la nuit, les tests non prioritaires peuvent être réalisés le lendemain matin. Dans ce cas, les échantillons de sang doivent être conservés au frigo jusqu'à analyse.**

**Les LCR traumatiques seront analysés tel quels mais référés comme traumatiques.**

## 4. Enregistrements et archives

| **Appendices & formulaires à compléter** | |
|---|---|
| **Numéro** | **Titre** |
| 1 | CRF laboratoire |
| Annexe 1 | Sommaire des test urgents |
| Annexe 2 | Sommaire de l'ordre de tests sur LCR |
| Annexe 3 | Sommaire des valeurs normales |
| Annexe 4 | Sommaire du circuit d'échantillons |
| Annexe 5 | Feuilles à remplir « Assurance de Qualité », |

| **Nom et fonction** | **Date** | **Signature** |
|---|---|---|
| *Auteur* | | |
| Barbara Barbé | 16/09/2012 | |
| *Révisé par* | | |
| Philippe Gillet | 16/09/2012 | |
| *Approuvé par* | | |
| Veerle Lejon | 24/Sep/2012 | |

# Annexe 1 : TESTS URGENTS

## (à faire le même jour que le prélèvement)

- **Sur sang total :**
  - **Hémoculture 1 & 2**
- **Sur sang héparine :**
  - **Mini-colonne**
- **Sang EDTA :**
  - **TDR HIV Determine**
  - **TDR HIV Unigold** } **Si Determine POSITIF**
  - **TDR HIV DoubleCheckGold** } **Si Determine POSITIF**
- **Liquide ganglionnaire :**
  - **Recherche à frais**
- **Sur LCR :**
  - **Aspect macroscopique**
  - **Culture en BactAlert pédiatrique**
  - **Culture en trans-isolate**
  - **Culture en MGIT**
  - **Numération GB**
  - **MSC**
  - **Si GB > 5/µl:**
    - **ET HIV + : Encre de Chine (lecture immédiatement)**
    - **Préparation + fixation lame pour Giemsa**
    - **Préparation + fixation lame pour Gram**
    - **Préparation + fixation lame pour Ziehl**

# Annexe 2 : ORDRE DE TESTS SUR LCR

12 ml de LCR (CS 1) *(6 ml pour enfants)*

1. Aspect macroscopique
2. BactAlert pédiatrique: 2 ml *(1 ml pour enfants)*
3. Trans-isolate: 0,5 ml *(0,2 ml pour enfants)*
4. MGIT: 0,5 ml *(0,5 ml pour enfants)*
   + 0,5 ml OADC
   + 100 µl PANTA
5. Numération des globules blancs en double
6. Tube collecteur/tube de centrifugation de SCM:
   4 ml *(2 ml pour enfants)*
   - Centrifugation 10 min, position 8 (AML centrifuge)
   - Surnageant → Lecture MSC
     → Cryotube CS 2: 1,5 ml
7. Centrifugation:
   - Culot → SI GB > 5/µl:
     - Encre de Chine (SI HIV +)
     - Giemsa
     - Gram
     - Ziehl
   - Surnageant → Cryotube CS 3
     → CrAg LFA (tous les patients)
     → CrAg Latex (SI HIV +)

## Annexe 3 : Valeurs normales

| | Test | Valeurs normales | Plage de mesure |
|---|---|---|---|
| | **SANG TOTAL** | | |
| 1 | Hémoculture | Absence de croissance/virage, absence d'organismes (bactéries) isolées du sang (OB) | |
| | **SERUM** | | |
| 2 | RPR syphilis (qualitatif) | Absence d'agglutination (négatif) | |
| 3 | RPR syphilis (quantitatif) | Absence d'agglutination (négatif) | |
| 4 | RDT CrAg LFA (qualitatif) | Négatif | |
| 5 | CATT dilution (quantitatif) | Absence d'agglutination (négatif) | |
| 6 | Reflotron: glucose | Adulte (à jeun): 60 - 109 mg/dL (augmentation de 2 mg/dL par décennie de vie) | 10.0 - 600 mg/dL |
| 7 | Reflotron: GPT (ALT) | Adulte masculin: ≤ 41 U/L (37°C)<br>Adulte féminin: ≤ 32 U/L (37°C) | 5 - 2000 U/L (37°C) |
| 8 | Reflotron: créatinine | Adulte masculin: < 1.1 mg/dL<br>Adulte féminin: < 0.90 mg/dL | 0.50 - 10.0 mg/dL |
| | **SANG HEPARINE** | | |
| 9 | mAECT (mini-colonne) | Absence de trypanosomes | |
| 10 | CATT sang total (qualitatif) | Absence d'agglutination (négatif) | |
| 11 | Gambiense-sero-K-set (Coris) | Négatif | |
| 12 | RDT HAT FIND | Négatif | |
| 13 | Glucose (appareil) | Adulte (à jeun): 70 - 105 mg/dl | 20 - 600 mg/dl |
| | **SANG EDTA** | | |
| 14 | Goutte épaisse/Giemsa | Absence d'organismes (parasites: Plasmodium, trypanosomes; bactéries: Borrelia) | |
| 15 | Hémoglobine (HemoCue 301) | Adulte masculin: 14 - 18 g/dL<br>Adulte féminin: 12 - 16 g/dL<br>Enfant: 11 - 14 g/dL<br>Nourrisson: 17 - 23 g/dL | 0 - 25.6 g/dL |

<table>
<tr><td>16</td><td>Numération GB + Diff (HemoCue WBC DIFF)</td><td>
Enfants (moyenne ± 2SD)
<table>
<tr><th></th><th>3-6 mois</th><th>1 an</th><th>2-6 ans</th><th>6-12 ans</th></tr>
<tr><td>WBC (x $10^9$/L)</td><td>12 ± 6</td><td>11 ± 5</td><td>10 ± 5</td><td>9 ± 4</td></tr>
<tr><td>Neutrophiles (x $10^9$/L)</td><td>1 - 6</td><td>1 - 7</td><td>1.5 - 8</td><td>2 - 8</td></tr>
<tr><td>Lymphocytes (x $10^9$/L)</td><td>4 - 12</td><td>3.5 - 11</td><td>6 - 9</td><td>1 - 5</td></tr>
<tr><td>Monocytes (x $10^9$/L)</td><td>0.2 - 1.2</td><td>0.2 - 1.0</td><td>0.2 - 1.0</td><td>0.2 - 1.0</td></tr>
<tr><td>Eosinophiles (x $10^9$/L)</td><td>0.1 - 1.0</td><td>0.1 - 1.0</td><td>0.1 - 1.0</td><td>0.1 - 1.0</td></tr>
</table>
Adultes (moyenne ± 2SD)
<table>
<tr><th></th><th>Adulte</th><th>Adulte %</th></tr>
<tr><td>WBC (x $10^9$/L)</td><td>4.0 - 10.0</td><td></td></tr>
<tr><td>Neutrophiles (x $10^9$/L)</td><td>2.0 - 7.0</td><td>40 - 80</td></tr>
<tr><td>Lymphocytes (x $10^9$/L)</td><td>1.0 - 3.0</td><td>20 - 40</td></tr>
<tr><td>Monocytes (x $10^9$/L)</td><td>0.2 - 1.0</td><td>2 - 10</td></tr>
<tr><td>Eosinophiles (x $10^9$/L)</td><td>0.02 - 0.5</td><td>1 - 6</td></tr>
<tr><td>Basophiles (x $10^9$/L)</td><td>0.02 - 0.1</td><td><1 - 2</td></tr>
</table>
</td><td>0.3 - 30 $10^9$ /L</td></tr>
<tr><td>17</td><td>RDT HIV Determine</td><td>Négatif</td><td></td></tr>
<tr><td>18</td><td>RDT HIV Unigold</td><td>Négatif</td><td></td></tr>
<tr><td>19</td><td>RDT HIV DoubleCheckGold</td><td>Négatif</td><td></td></tr>
<tr><td>20</td><td>Lymphocytes T CD4+</td><td>500 - 1000 cellules/µl</td><td>> 6 cellules/µl ± 2 écarts-type. Sous-estimation possible > 800 cellules/µl</td></tr>
<tr><td>21</td><td>RDT Syphilis (SD Bioline)</td><td>Négatif</td><td></td></tr>
<tr><td>22</td><td>RDT malaria SDFK60 (SD Bioline)</td><td>Négatif</td><td></td></tr>
<tr><td>23</td><td>RDT malaria Carestart pLDH</td><td>Négatif</td><td></td></tr>
<tr><td></td><td>URINE</td><td></td><td></td></tr>
<tr><td>24</td><td>RDT CrAg LFA (qualitatif)</td><td>Négatif</td><td></td></tr>
</table>

| | | | |
|---|---|---|---|
| | **LIQUIDE GANGLIONNAIRE** | | |
| 25 | Recherche de tryp dans le liquide ganglionnaire | Absence de trypanosomes | |
| | **LCR** | | |
| 26 | Culture trans-isolate | Absence de croissance, absence d'organismes (bactéries) isolées du LCR (OC) | |
| 27 | Culture BactAlert pédiatrique | Absence de croissance, absence d'organismes (bactéries) isolées du LCR (OC) | |
| 28 | Aspect macroscopique du LCR | Clair et incolore<br>Pas:<br>- trouble ou blanc-grisâtre/purulent (présence de pus)<br>- trouble et rose/rougeâtre (présence de sang: ponction traumatique ou hémorragie subarachnoïdienne)<br>- coloré et jaune (hémorragie ancienne, ictère grave, compression rachidienne)<br>- de formation de caillots après 10 minutes (méningite tuberculeuse, méningite purulente, compression rachidienne) | |
| 29 | MSC (simple centrifugation modifiée) | Absence de trypanosomes | |
| 30 | Culture MGIT | Absence de croissance, absence de détection de *Mycobacterium tuberculosis* | |
| 31 | Numération GB (Uriglass) | 0 - 5/µL | |
| 32 | Encre de Chine | Absence de cryptocoques | |
| 33 | Giemsa/GB diff | 0 - 5/µL | |
| 34 | Ziehl-Neelsen | Absence de BAAR (bacilles alcoolo-acido résistant) | |
| 35 | Glucose | 75% de la glycémie | |
| 36 | RDT CrAg LFA (qualitatif) | Négatif | |
| 37 | CrAg LAT (latex) (qualitatif) | Absence d'agglutination (négatif) | |
| 38 | Crag LAT (latex) (quantitatif) | Absence d'agglutination (négatif) | |

# Annexe 4 : Circuit des échantillons

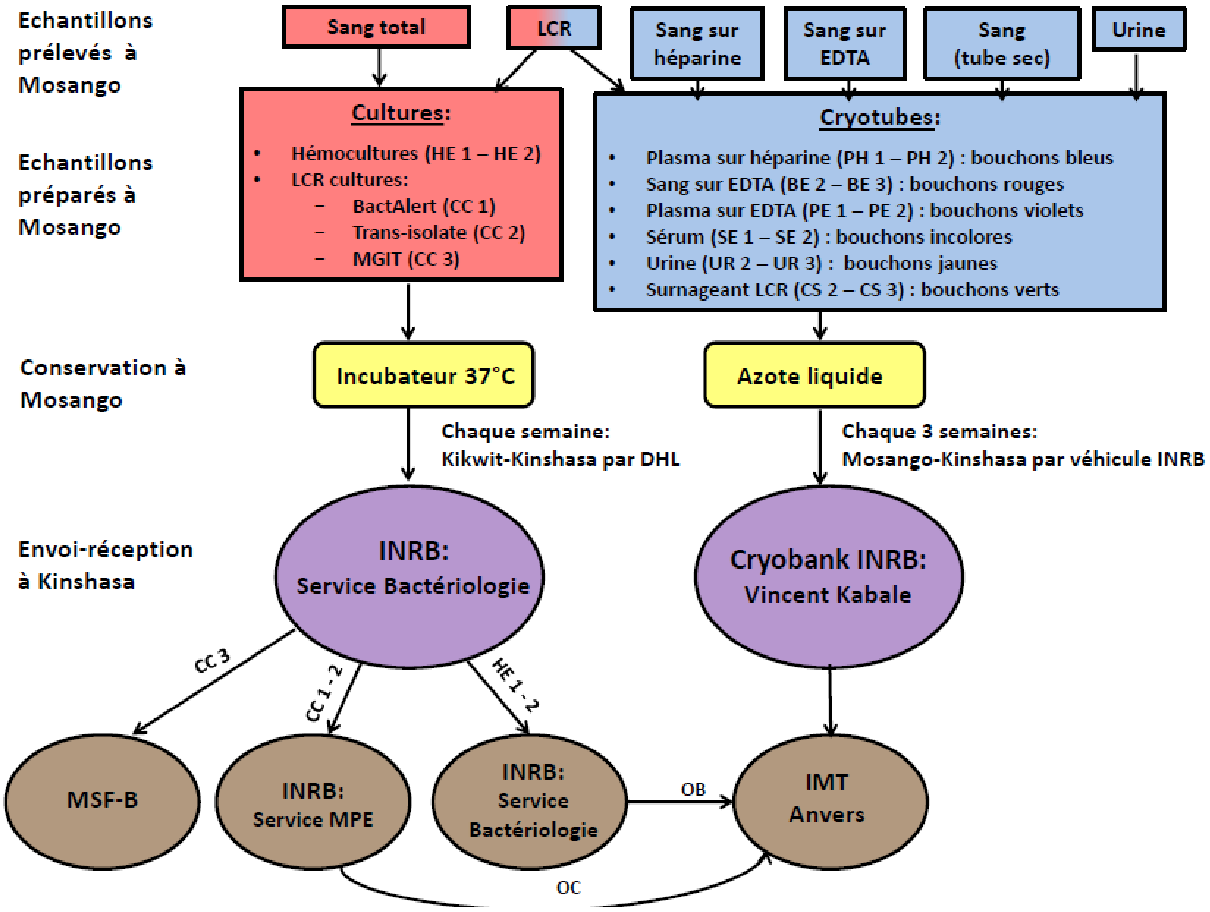

nidiag

# Assurance de qualité
## Lateral Flow Assays

**Mosango**

| Tests de laboratoire | Paramètres de AQ | 09/2012 | 10/2012 | 11/2012 | 12/2012 | 01/2013 | 02/2013 | 03/2013 | 04/2013 | 05/2013 | 06/2013 | 07/2013 | 08/2013 | 09/2013 | 10/2013 | 11/2013 | 12/2013 |
|---|---|---|---|---|---|---|---|---|---|---|---|---|---|---|---|---|---|
| **SD – FIND HAT dipstick**<br>LOT N°:<br>Date de péremption: | % résultats invalides | | | | | | | | | | | | | | | | |
| | % bandes de tests faiblement visibles | | | | | | | | | | | | | | | | |
| | % différence avec mAECT | | | | | | | | | | | | | | | | |
| | % différence avec CATT | | | | | | | | | | | | | | | | |
| **Coris – Gambiense-Sero-K-Set**<br>LOT N°:<br>Date de péremption: | % résultats invalides | | | | | | | | | | | | | | | | |
| | % bandes de tests faiblement visibles | | | | | | | | | | | | | | | | |
| | % différence avec mAECT | | | | | | | | | | | | | | | | |
| | % différence avec CATT | | | | | | | | | | | | | | | | |
| **Cryptococcal Antigen Lateral Flow Assay (CrAg-LFA)**<br>LOT N°:<br>Date de péremption: | % résultats invalides | | | | | | | | | | | | | | | | |
| | % bandes de tests faiblement visibles | | | | | | | | | | | | | | | | |
| | % différence avec test de référence (CrAg Latex) | | | | | | | | | | | | | | | | |
| **SD FK60 malaria (HRP2/pan-pLDH)**<br>LOT N°:<br>Date de péremption: | % résultats invalides | | | | | | | | | | | | | | | | |
| | % bandes de tests faiblement visibles | | | | | | | | | | | | | | | | |
| | % différence avec test de référence (goutte épaisse) | | | | | | | | | | | | | | | | |
| **CARESTART 0121 P.f. pLDH/pan pLDH**<br>LOT N°:<br>Date de péremption: | % résultats invalides | | | | | | | | | | | | | | | | |
| | % bandes de tests faiblement visibles | | | | | | | | | | | | | | | | |
| | % différence avec test de référence (goutte épaisse) | | | | | | | | | | | | | | | | |

Recommendations:
1. Résultats invalides < 2%.
2. Bandes de tests faiblement visibles < 20%.
3. Différence avec le test de référence < 5%.

## Assurance de qualité
### Lateral Flow Assays

**Mosango**

| Tests de laboratoire | Paramètres de AQ | 09/2012 | 10/2012 | 11/2012 | 12/2012 | 01/2013 | 02/2013 | 03/2013 | 04/2013 | 05/2013 | 06/2013 | 07/2013 | 08/2013 | 09/2013 | 10/2013 | 11/2013 | 12/2013 |
|---|---|---|---|---|---|---|---|---|---|---|---|---|---|---|---|---|---|
| **SD Bioline Syphilis 3.0**<br>LOT N°:<br>Date de péremption: | % résultats invalides | | | | | | | | | | | | | | | | |
| | % bandes de tests faiblement visibles | | | | | | | | | | | | | | | | |
| | % différence avec test de référence (Syphilis RPR) | | | | | | | | | | | | | | | | |
| **Determine HIV-1/2**<br>LOT N°:<br>Date de péremption: | % résultats invalides | | | | | | | | | | | | | | | | |
| | % bandes de tests faiblement visibles | | | | | | | | | | | | | | | | |
| **Uni-Gold HIV-1/2**<br>LOT N°:<br>Date de péremption: | % résultats invalides | | | | | | | | | | | | | | | | |
| | % bandes de tests faiblement visibles | | | | | | | | | | | | | | | | |
| **DoubleCheckGold HIV-1/2**<br>LOT N°:<br>Date de péremption: | % résultats invalides | | | | | | | | | | | | | | | | |
| | % bandes de tests faiblement visibles | | | | | | | | | | | | | | | | |

Recommendations:
1. Résultats invalides < 2%.
2. Bandes de tests faiblement visibles < 20%.
3. Différence avec le test de référence < 5%.

Mosango

## Assurance de qualité
### Tests d'agglutination - Lateral flow assays

**MOIS:**

| Tests de laboratoire | Paramètres de AQ | Semaine 1<br>Date: | Semaine 2<br>Date: | Semaine 3<br>Date: | Semaine 4<br>Date: | Semaine 5<br>Date: |
|---|---|---|---|---|---|---|
| | | Résultat | Résultat | Résultat | Résultat | Résultat |
| **Cryptococcal Antigen Lateral Flow Assay (CrAg-LFA)**<br>LOT N°:<br>Date de péremption: | Contrôle positif | | | | | |
| | Contrôle négatif | | | | | |
| **CATT**<br>LOT N°:<br>Date de péremption: | Intensité de réaction du contrôle positif<br>(Cible: ±, +, ++, +++) | | | | | |
| | Intensité de réaction du contrôle négatif<br>(Cible: négatif) | | | | | |
| **Cryptococcal Antigen Latex Test**<br>LOT N°:<br>Date de péremption: | Intensité de réaction du contrôle positif<br>(Cible: 2+, 3+, 4+) | | | | | |
| | Intensité de réaction du contrôle négatif<br>(Cible: 1+, négatif) | | | | | |
| | Sensibilité du réactif latex: Titre du contrôle positif<br>(Cible: 1:4 ± 1 dilution) | | | | | |

## Assurance de qualité
### Tests d'agglutination - Lateral flow assays

**Mosango**

**MOIS:**

| Tests de laboratoire | Paramètres de AQ | Semaine 1<br>Date: | Semaine 2<br>Date: | Semaine 3<br>Date: | Semaine 4<br>Date: | Semaine 5<br>Date: |
|---|---|---|---|---|---|---|
| **Syphilis RPR**<br>LOT N°:<br>Date de péremption: | Intensité de réaction du contrôle positif (++)<br>(Cible: réactif) | | | | | |
| | Intensité de réaction du contrôle positif modéré (+)<br>(Cible: réactif minimale/modéré) | | | | | |
| | Intensité de réaction du contrôle négatif (-)<br>(Cible: non-réactif) | | | | | |
| | Sensibilité du suspension antigène:<br>Titre du contrôle positif (++)<br>(Cible: 1:8) | | | | | |
| | Sensibilité du suspension antigène:<br>Titre du controle positif modéré (+)<br>(Cible: 1:4) | | | | | |

**Assurance de qualité**
**Tests d'agglutination**

**Mosango**

| Tests de laboratoire | Paramètres de AQ | 09/2012 | 10/2012 | 11/2012 | 12/2012 | 01/2013 | 02/2013 | 03/2013 | 04/2013 | 05/2013 | 06/2013 | 07/2013 | 08/2013 | 09/2013 | 10/2013 | 11/2013 | 12/2013 |
|---|---|---|---|---|---|---|---|---|---|---|---|---|---|---|---|---|---|
| **CATT**<br>LOT N°:<br>Date de péremption: | % agglutination faible (±) | | | | | | | | | | | | | | | | |
| | % des contrôles non correctes | | | | | | | | | | | | | | | | |
| **Cryptococcal Antigen Latex Test**<br>LOT N°:<br>Date de péremption: | % agglutination faible (2+) | | | | | | | | | | | | | | | | |
| | % des contrôles non correctes | | | | | | | | | | | | | | | | |
| **Syphilis RPR**<br>LOT N°:<br>Date de péremption: | % agglutination faible (agglutination minimale) | | | | | | | | | | | | | | | | |
| | % des contrôles non correctes | | | | | | | | | | | | | | | | |

Recommendations:
1. Agglutination faible < 20%.
2. Différence avec un test pertinent < 5%.
3. Contrôles non correctes = 0%

**Mosango**

## Assurance de qualité
### Tests microscopiques

| Tests de laboratoire | Paramètres de AQ | 09/2012 | 10/2012 | 11/2012 | 12/2012 | 01/2013 | 02/2013 | 03/2013 | 04/2013 | 05/2013 | 06/2013 | 07/2013 | 08/2013 | 09/2013 | 10/2013 | 11/2013 | 12/2013 |
|---|---|---|---|---|---|---|---|---|---|---|---|---|---|---|---|---|---|
| **Différentiation de globules blancs sur LCR (frottis mince/Giemsa)**<br>LOT N° (Giemsa):<br>Date de péremption: | % différence (> 5%) après relecture par le 2ième lecteur | | | | | | | | | | | | | | | | |
| | pH d'eau tamponnée après préparation | | | | | | | | | | | | | | | | |
| **Goutte épaisse sur sang EDTA/Giemsa**<br>LOT N° (Giemsa):<br>Date de péremption: | % différence (+ vs -) après relecture par le 2ième lecteur | | | | | | | | | | | | | | | | |
| | pH d'eau tamponnée après préparation | | | | | | | | | | | | | | | | |
| **Frottis mince sur LCR/Gram**<br>LOT N° (Gram):<br>Date de péremption: | % différence après relecture par le 2ième lecteur | | | | | | | | | | | | | | | | |
| **Frottis mince sur LCR/Ziehl-Neelsen**<br>LOT N° (Ziehl-Neelsen):<br>Date de péremption: | % différence (+ vs -) après relecture par le 2ième lecteur | | | | | | | | | | | | | | | | |
| **Dynal T4 Quant Kit (sang EDTA)**<br>LOT N°:<br>Date de péremption: | Variation intra-individuel (3 mesures) (CV %) | | | | | | | | | | | | | | | | |

Recommendations:
1. pH d'eau tamponnée 7 - 7.4
2. Différence après relecture par le 2ième lecteur < 5%
3. CV% < 10%

nidiag

**Assurance de qualité**
**Tests complémentaires**

**Mosango**

| | LOT N° | Date de péremption |
|---|---|---|
| Eurotrol Hb 301 Low | | |
| Eurotrol Hb 301 Normal | | |

| | LOT N° | Date de péremption |
|---|---|---|
| Reflotron Precinorm U | | |
| Reflotron Precipath U | | |

**Mois:**

| | Contrôle interne | Semaine 1 Date: | | Semaine 2 Date: | | Semaine 3 Date: | | Semaine 4 Date: | | Semaine 5 Date: | |
|---|---|---|---|---|---|---|---|---|---|---|---|
| | | Résultat | Dans la plage? Oui/Non | Résultat | Dans la plage? Oui/Non | Résultat | Dans la plage? Oui/Non | Résultat | Dans la plage? Oui/Non | Résultat | Dans la plage? Oui/Non |
| **Hémoglobine (HemoCue Hb 301)** LOT N°: Date de péremption: | Eurotrol Hb 301 Low | | | | | | | | | | |
| | Eurotrol Hb 301 Normal | | | | | | | | | | |
| **Glucose (Reflotron)** LOT N°: Date de péremption: | Reflotron Precinorm U | | | | | | | | | | |
| | Reflotron Precipath U | | | | | | | | | | |
| **Créatinine (Reflotron)** LOT N°: Date de péremption: | Reflotron Precinorm U | | | | | | | | | | |
| | Reflotron Precipath U | | | | | | | | | | |
| **GPT/ALT (Reflotron)** LOT N°: Date de péremption: | Reflotron Precinorm U | | | | | | | | | | |
| | Reflotron Precipath U | | | | | | | | | | |
| **Reflotron Clean and Check** LOT N°: Date de péremption: | Valeur 1 | | | | | | | | | | |
| | Valeur 2 | | | | | | | | | | |
| | Valeur 3 | | | | | | | | | | |

**Assurance de qualité**
**Cultures**

**INRB Kinshasa**

| Tests de laboratoire | Paramètres de AQ | 09/2012 | 10/2012 | 11/2012 | 12/2012 | 01/2013 | 02/2013 | 03/2013 | 04/2013 | 05/2013 | 06/2013 | 07/2013 | 08/2013 | 09/2013 | 10/2013 | 11/2013 | 12/2013 |
|---|---|---|---|---|---|---|---|---|---|---|---|---|---|---|---|---|---|
| **Hémoculture 1 (BactAlert), HE 1**<br>LOT N°:<br>Date de péremption: | % contamination | | | | | | | | | | | | | | | | |
| | % positifs après réanalyse des 10% cultures negatives | | | | | | | | | | | | | | | | |
| **Hémoculture 2 (BactAlert), HE 2**<br>LOT N°:<br>Date de péremption: | % contamination | | | | | | | | | | | | | | | | |
| | % positifs après réanalyse des 10% cultures negatives | | | | | | | | | | | | | | | | |
| **Trans-Isolate LCR, CC 1**<br>LOT N°:<br>Date de péremption: | % contamination | | | | | | | | | | | | | | | | |
| **BactAlert LCR, CC 2**<br>LOT N°:<br>Date de péremption: | % contamination | | | | | | | | | | | | | | | | |
| | % positifs après réanalyse des 10% cultures negatives | | | | | | | | | | | | | | | | |
| **MGIT LCR, CC 3**<br>LOT N°:<br>Date de péremption: | % positifs après réanalyse des 10% cultures TB negatives | | | | | | | | | | | | | | | | |

Recommendations:
1. Contamination < 10 %
2. Positifs après réanalyse de 10% cultures négatives < 5%

| | |
|---|---|
| nidiag Better Diagnosis of Neglected Infectious Diseases | **SOP titre :** Utilisation du Reflotron® Plus (Roche) Analyseur Biochimique |
| | **Projet/Étude :** Cette SOP s'applique à toutes les études du WP2 du projet NIDIAG qui sont exécutées en RDC (site du Mosango, province de Bandundu) |

# 1. Domaine et application

Ce document décrit la façon dont le Reflotron Plus (Roche) doit être utilisé. Il donne des instructions pour assurer la sécurité et la précision des mesures des paramètres de chimie clinique : Bilirubine, glucose, GPT (ALT), GOT (AST), urée et créatinine.
Les bandelettes de Reflotron sont utilisées pour déterminer des paramètres de chimie clinique spécifiques. Elles s'utilisent sur des échantillons non-diluées. L'échantillon entre en contact avec les réactifs présents sur la bandelette et la réaction démarre. Une lampe de type LED envoie de la lumière à différentes longueurs d'onde sur la surface de la zone de test. La lumière est reflétée avec une intensité qui dépend de la couleur de la zone de test (= photométrie de réflectance). La réflectance est mesurée et la concentration (des substrats) ou l'activité (des enzymes) est calculée.

# 2. Responsabilités

| Fonction | Activités |
|---|---|
| Technicien de laboratoire | ▪ Respect de cette procédure<br>▪ Prélèvement des échantillons biologiques<br>▪ Stockage du matériel et des réactifs en accord avec les instructions du fabriquant<br>▪ Enregistrement des données (CRF, cahier de laboratoire)<br>▪ Suivi des règles d'assurance qualité et maintien des formulaires des contrôles de qualité effectués<br>▪ Respect des procédures d'entretien de l'appareil |

# 3. Procédures

## 3.1 Précautions

Tous les échantillons de sang et d'urine sont potentiellement infectieux. Respectez les précautions universelles. METTRE DES GANTS À USAGE UNIQUE PENDANT TOUTE LA PROCÉDURE **!**

## 3.2 Matériel et échantillons

### 3.2.1 Matériel fourni et conservation

- Analyseur Reflotron Plus.
  *Utilisation*: entre 15 et 34°C, avec une humidité relative < 95%.
- Bandelettes Reflotron: Bilirubine (totale), glucose, créatinine, GPT (ALT), GOT (AST), urée.
  *Conservation : entre* 2 et 30°C. Après chaque ouverture, refermer le flacon immédiatement.
- Pipette de Reflotron de 32 µl.
- Bandelettes « Clean and Check ».
  *Conservation*: entre 2 et 30°C. Après chaque ouverture refermer le flacon immédiatement.
- Solutions de contrôle: Reflotron Precinorm/Precipath U.
  *Conservation* : Sous forme lyophilisée à 2-8°C jusqu'à la date de péremption.
  Sous forme reconstituée en dessous de 25°C pendant 8 heures, à 4°C pendant 3 jours, à -20°C pendant 8 semaines (si congelée pour la première fois).

### 3.2.2 Matériel supplémentaire requis

- Gants, non stériles, à usage unique.
- Pointes jaunes.
- Clavier compatible IBM.
- Tissus imbibés d'alcool éthanol à 70%.

### 3.2.3 Echantillon à examiner

- Sérum (32 µl par paramètre)
- Durée et température de conservation du sérum*:*
  - Bilirubine : à utiliser immédiatement, maximum 2 heures après la centrifugation à 4-25°C.
  - Glucose : à utiliser immédiatement, au maximum 2 heures après la centrifugation.
  - Créatinine : maximum 24 heures à 4 – 25°C.
  - ALT (GPT) : maximum 3 jours à 20-25°C, ou 7 jours à 4-8°C.
  - AST (GOT) : maximum 4 jours à 20-25°C, 7 jours à 4-8°C.
  - Urée : maximum 7 jours à 4-25°C.

## 3.3 Mode opératoire

### 3.3.1 Contrôle de qualité

- L'analyseur effectue un test de contrôle électronique interne de l'unité optique à chaque démarrage et toutes les 2 heures quand l'appareil reste allumé.
- Effectuez les tests de contrôle interne **une fois par semaine** au début du travail, en utilisant:
    - Reflotron Precinorm U (Roche) : contrôle normal
    - Reflotron Precipath U (Roche) : contrôle anormal (pathologique)
  - Reconstituez les solutions de contrôle lyophilisées avec 2 ml d'eau distillée (eau injectable). Dissolvez le contenu par agitations occasionnelles pendant 30 minutes. Evitez la formation de mousse.
  - Effectuez l'analyse de chaque paramètre (bilirubine, glucose, créatinine, ALT (GPT), AST (GOT) et urée) de la même manière que pour un échantillon de patient (voir « 3.3.3 Procédure »).
  - Contrôlez si les valeurs de chaque paramètre sont comprises dans la plage de valeurs indiquée dans la notice.
    - Si oui : Commencez les tests des patients.
    - Si non : Cherchez la cause du problème (exemples : volume insuffisant de la solution de contrôle). Si possible résolvez le problème et répétez le test de contrôle.
      Contactez le fournisseur si le problème persiste.
- Effectuez le « clean and check » **une fois par semaine** ou **à chaque fois que 100 tests ont été effectués** (l'analyseur le demandera automatiquement après 100 mesures) :
  - Consultez le point « 3.4 Entretien » pour le nettoyage.
  - Utilisez les bandelettes « Clean and Check » (Roche) pour contrôler le système optique. La zone grise sur les bandelettes a une valeur de réflectance bien définie. Le Reflotron Plus détermine la réflectance de la lumière sur la bandelette à 3 longueurs d'onde et affiche les valeurs en pour mil (‰) :
    1ere valeur : réflectance en ‰ à 642 nm
    2ème valeur : réflectance en ‰ à 567 nm
    3ème valeur : réflectance en ‰ à 951 nm

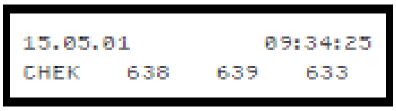


  - Contrôlez si les 3 valeurs sont comprises dans la plage de valeurs affichée sur l'étiquette du flacon.
    - Si oui : Le système optique fonctionne de manière fiable.
    - Si non : Nettoyez l'analyseur et répétez le contrôle. Contactez le fournisseur si le problème persiste.

- Les données de contrôle sont stockées dans la mémoire (jusqu'à 60 résultats et contrôles).

### 3.3.2 Étalonnage

- La bande magnétique logée à l'arrière des bandelettes contient toutes les données spécifiques du test et du lot. Ces données sont utilisées pour le calcul de la concentration ou de l'activité des paramètres de chimie clinique à partir de la réflectance mesurée.
- Une fois par an, un étalonnage par le fournisseur est requis.

### 3.3.3 Procédure

1. Démarrage de l'appareil :
    - Branchez le câble d'alimentation.
    - Si nécessaire, effectuez un « clean and check ». Consultez les points « 3.4 Entretien » et « 3.3.1 Contrôle de qualité ».
    - Allumez l'appareil: appuyez sur la touche on/off à gauche.
        - Tous les segments de l'affichage apparaissent. Contrôlez si tous les segments sont présents.

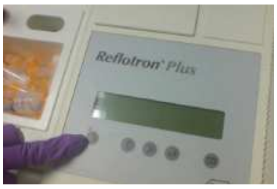


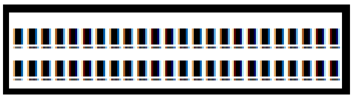

        - La date, l'heure et une identification codée sont affichées.
        - L'analyseur chauffe. Le temps d'attente est affiché.
        - L'analyseur est prêt à l'emploi.
2. Contrôlez la date de péremption des bandelettes de Reflotron. Si elles sont expirées, jetez-les et prenez un nouveau lot de bandelettes.
3. Mettre des gants à usage unique.
4. Si nécessaire, effectuez un test de contrôle interne (voir « 3.3.1 Contrôle de qualité »). Si les résultats des tests de contrôle sont corrects, continuez avec les échantillons de patients.
5. Mélangez bien l'échantillon du patient (inversez manuellement 8-10 fois le tube).
6. Utilisez le clavier pour introduire des données du patient.
7. Enlevez une bandelette du flacon et refermez le flacon immédiatement.
8. Enlevez le film protecteur**. NE pliez PAS la bandelette!** Cela peut endommager la zone de test.
9. Insérez la bandelette dans le support.
10. Prélevez 32 µl d'échantillon avec la pipette de Reflotron.
11. Appliquez l'échantillon sur le centre de la zone d'application (couverte d'une maille). L'échantillon est immédiatement absorbé.
    **NE PAS toucher la zone d'application avec l'embout.**

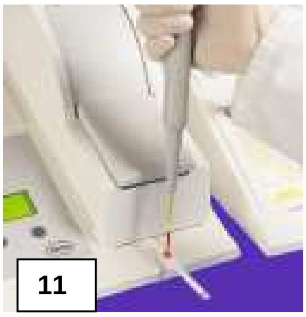


12. Soulevez le rabat. Le message « Insérer la bandelette » est affiché.
13. Insérez la bandelette horizontalement dans la chambre de mesure, au plus tard **15 minutes** après l'application de l'échantillon.

14. Le message « Fermer chambre de mesure est affiché ».
    Note : Si le message « Fermer chambre de mesure » n'est pas affiché, poussez le levier de déblocage vers le haut et réinsérez la bandelette.
15. Fermez le rabat.
16. « Lecture du code » est affiché et le code magnétique est lu.
    Commentaire : Si le message « Code mag. illisible » est affiché, le code magnétique n'est pas lu ou sa lecture est erronée. Enlevez la bandelette et réinsérez-la ou utilisez une nouvelle bandelette.
17. L'abréviation du paramètre est affichée avec le temps d'attente avant l'affichage du résultat.
18. Le résultat, l'abréviation du paramètre, les unités, la date, l'heure et le numéro d'identification du patient sont affichés. Le résultat est imprimé automatiquement. Ouvrez le rabat et enlevez la bandelette. Examinez le développement de la couleur sur la zone de test. Contrôlez si toute la zone de test est colorée.
19. Jetez la bandelette dans un récipient « Bio-Hazard ». NE JAMAIS réutiliser les bandelettes!

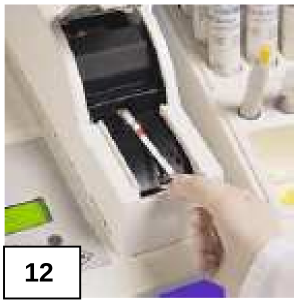


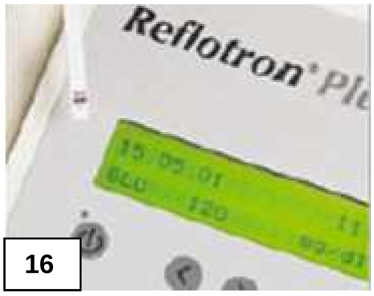


#### 3.3.4 Messages d'erreur :

- Résultat avec un astérisque (*) et le message « Réaction non linéaire »
  → La cinétique de la réaction n'est pas complètement linéaire.
  → Diluez l'échantillon. Consultez la notice du test Reflotron correspondant pour la dilution correcte.
- « Pas de résultat » et « Réaction non linéaire »
  → La cinétique de la réaction n'est pas du tout linéaire.
  → Répétez le test. Si le problème persiste, utilisez une autre technique biochimique pour déterminer la concentration ou l'activité du paramètre. Si aucun résultat ne peut être obtenu, noter « ND » pour « Not Done » dans le CRF.
- Consultez le manuel de l'utilisateur pour tous les autres messages d'erreur possibles.

## 3.4 Entretien

- Effectuez le nettoyage de l'appareil tous les 7 jours, après 100 mesures ou plus souvent si l'appareil est sale.
- Eteignez l'analyseur.
- Nettoyez les parties extérieures de l'analyseur avec un détergent doux ou un désinfectant, comme de l'alcool éthanol à 70%.
- Nettoyez le système de mesure :
  1. Ouvrez le rabat et enlevez toute bandelette restante.
  2. Soulevez le petit rabat à l'intérieur vers l'avant autant que possible.
  3. Utilisez un tissu imbibé d'alcool éthanol à 70%, ou un coton-tige humidifié avec de

l'eau, pour nettoyer le chauffage supérieur, et en particulier l'ouverture.
**NE PAS utiliser un spray désinfectant dans la chambre de mesure !**

4. Essuyez le transporteur, le chauffage inférieur et la tête magnétique avec un tissu imbibé d'alcool éthanol à 70%
5. Laissez le rabat ouvert au moins 10 minutes pour le séchage.

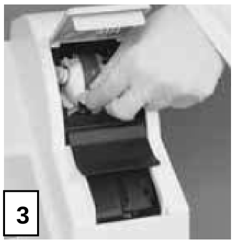


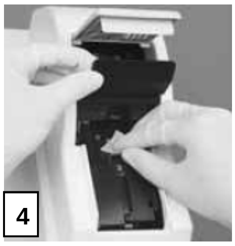


### 3.5 Documentation des résultats

- Enregistrer les résultats des contrôles de qualité (voir 3.3.1) dans les formulaires des contrôles de qualité effectués. Garder ces formulaires dans le Laboratory File.
- Enregistrer les résultats dans le CRF (du syndrome neurologique ou du syndrome fièvre en fonction de l'étude où le patient est inclus).

## 4. Enregistrements et archives

| **Appendices & formulaires à compléter** | |
|---|---|
| **Numéro** | **Titre** |
| 1 | CRF |

## 5. Historique du document

| **Révision** | |
|---|---|
| SOP-WP2-LAB-38-V01-17Sep2012 | Version initiale |
| SOP-WP2-LAB-38-V2.0-12Sep2013 | Ajout des tests pour le syndrome fièvre<br>Correction de la syntaxe |

| **Nom et fonction** | **Date** | **Signature** |
|---|---|---|
| *Auteur* | | |
| Barbara Barbé | 14/04/2013 | [signature] |
| *Révisé par* | | |
| | | |
| *Approuvé par* | | |
| Ninon Horié | 12/09/2013 | [signature] |

| | |
|---|---|
| | **SOP titre :** Mise en culture du LCR en milieu Trans-Isolate (T-I) |
| | **Projet/Étude :** Evaluation de tests rapides en association avec des prédicteurs cliniques et de laboratoire pour le diagnostic de maladies tropicales négligées dans des patients se présentant avec des troubles neurologiques dans les hôpitaux ruraux au Bandundu, République Démocratique du Congo. |

# 1. Domaine et application

Ce document fournit les instructions pour la mise en culture du liquide céphalo rachidien (LCR) en milieu trans-isolate. Le trans-isolate est un milieu diphasique (liquide et solide) qui est utilisé pour l'isolation de N. meningitidis, S. pneumoniae, et H. influenzae dans les liquides céphalorachidiens. Le trans-isolate peut être utilisé comme milieu de croissance, de conservation et de transport. L'identification des bactéries éventuellement présentes sera réalisée à l'INRB (Kinshasa).

# 2. Responsabilités

| Fonction | Activités |
|---|---|
| Technicien de laboratoire | ▪ Met le LCR en culture<br>▪ Incube la culture trans-isolate<br>▪ Expédie les cultures à l'INRB, Kinshasa |

# 3. Procédures

## 3.1 Précautions

- Tous les échantillons de liquide céphalorachidien sont potentiellement infectieux. Respectez les précautions universelles.
  METTEZ DES GANTS À USAGE UNIQUE PENDANT TOUTE LA PROCÉDURE **!**
  METTEZ UN MASQUE RESPIRATOIRE FFP3 PENDANT TOUTE LA PROCÉDURE **!**

## 3.2 Matériel et échantillons

### 3.2.1 Matériel requis

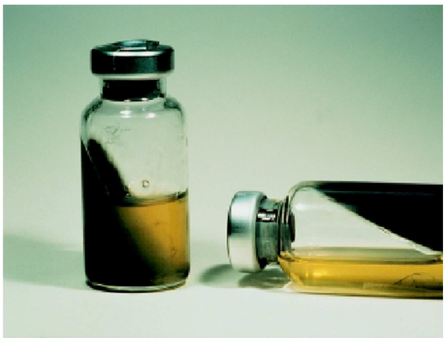

**Figure: Milieux trans-isolate**

- Gants, non stérile, à usage unique
- Masque respiratoire FFP3
- Flacon de trans-isolate (T-I), prêt à l'emploi
  *Conservation* : au réfrigérateur 2 – 8°C.
  *Stabilité* : 6 mois après la date de fabrication
- Alcool éthanol à 70%
- Compresses
- Seringue de 2 ml
- Aiguille 21 G
- Aiguille 18 G
- Camping -gaz
- Coton hydrophile
- Sparadrap
- Système de transport de matériel biologique (triples emballages)

### 3.2.2 Echantillon à examiner

- Tube stérile de liquide céphalorachidien (LCR)
  0.5 ml (pour un adulte) ou 0.2 ml (pour un enfant) (voir SOP-WP2-CLIN-03)
  *Stabilité* : Inoculez le milieu dans l'heure qui suit le prélèvement de LCR.

## 3.3 Mode opératoire

### 3.3.1 Avant l'emploi

1. Vérifiez l'aspect macroscopique du milieu trans-isolate :
   - Le milieu doit contenir un liquide clair
   - Aucune colonie ne doit être visible sur la gélose noire
2. Détruisez le milieu s'il présente des signes de contamination :
   - Le liquide est trouble
   - Présence de colonies sur la gélose

### 3.3.2 Inoculation du milieu trans-isolate (TI)

1. Laissez revenir le milieu T-I à température ambiante avant de l'utiliser.
2. Identifiez le flacon de milieu.
3. Désinfectez le bouchon en caoutchouc du flacon de T-I à l'alcool éthanol à 70%.
4. Déposez le flacon sur le portoir.
5. Laissez sécher le bouchon 1 minute.
6. Attachez une aiguille verte 21 G sur une seringue de 2 ml.
7. Homogénéisez l'échantillon de LCR.
8. Aspirez 0.5 ml stérilement (pour un adulte) ou 0.2 ml (pour un enfant) de LCR du tube stérile ;
9. Injectez stérilement le LCR à travers le bouchon du flacon de T-I.
10. Après injection, introduisez une grosse aiguille 18 G dans le bouchon pour ventiler le milieu.
11. Bouchez l'embout de l'aiguille avec un coton hydrophile pour éviter les contaminations.
12. Fixez le coton avec un sparadrap.
13. Inscrivez les données dans le CRF laboratoire.
14. Incubez le milieu ventilé avec l'aiguille à 37°C jusqu'au jour de l'envoi.
    (<u>min. 2-3 jours, max. 3 semaines</u>).
    *Conservation* : dans l'incubateur (< 40°C), à l'abri de la lumière directe.
    NE JAMAIS RÉFRIGÉRER !

### 3.3.3 Contrôle de croissance

1. Contrôlez avant de préparer les flacons T-I pour l'envoi s'il y a une croissance visible :
   - Le liquide est trouble
   - Présence de colonies sur la gélose
2. S'il n'y a pas de croissance visible : continuez avec « 3.1.4 Préparation pour l'envoi »
3. S'il y a une croissance visible :
   - Etiquetez un deuxième flacon T-I avec le même numéro d'échantillon que le premier flacon T-I.
   - Inoculez stérilement 1 ml du bouillon du premier flacon dans le deuxième flacon. Notez sur le deuxième flacon que c'est un ensemencement du premier flacon (avec un symbole ou un signe).
   - Faites une coloration de Gram sur un étalement du bouillon du premier flacon.
4. Préparez les deux flacons T-I pour l'envoi comme décrit ci-dessous.

### 3.3.4 Préparation pour l'envoi

1. Retirez l'aiguille de ventilation.
2. Emballez les flacons en triple emballage. Envoyez le triple emballage pour cultures et identifications à l'INRB (Kinshasa). Un envoi par semaine.
   Le transport se fait à température ambiante et peut durer <u>1 semaine</u> sans problème.
   NE JAMAIS RÉFRIGÉRER !

## 4. Enregistrements et archives

| **Appendices & formulaires à compléter** | |
|---|---|
| **Numéro** | **Titre** |
| 1 | CRF “laboratoire” |

## 5. Références

Notes pratiques de bactériologie médicale tropicale, septembre 2009. Institut de Médicine Tropicale. Philippe Gillet, Luc Boels, Jan Jacobs.

## 6. Historique du document

| **Révision** | |
|---|---|
| SOP-WP2-LAB-39-V01-17Sep2012 | Version initiale |
| SOP-WP2-LAB-39-V02-27Dec2012 | Addition de la partie “3.1.3 Contrôle de croissance » |

| **Nom et fonction** | **Date** | **Signature** |
|---|---|---|
| *Auteur* | | |
| Barbara Barbé | 27/12/2012 | |
| *Révisé par* | | |
| Philippe Gillet | 28/12/2012 | |
| *Approuvé par* | | |
| Emilie Alirol | 02/01/2013 | |

| | |
|---|---|
| | **SOP titre :** Mise en culture du LCR en flacons Bact/Alert pédiatriques |
| | **Projet/Étude :** Evaluation de tests rapides en association avec des prédicteurs cliniques et de laboratoire pour le diagnostic de maladies tropicales négligées dans des patients se présentant avec des troubles neurologiques dans les hôpitaux ruraux au Bandundu, République Démocratique du Congo. |

# 1. Domaine et application

*Ce document fournit les instructions pour la mise en culture du liquide céphalo rachidien (LCR) en milieu Bact/Alert. Ce milieu permet la culture des bactéries présentes dans le LCR. L'identification des bactéries éventuellement présentes sera réalisée à l'INRB (Kinshasa).*

# 2. Responsabilités

| Fonction | Activités |
|---|---|
| *Technicien de laboratoire* | ▪ *Met le LCR en culture*<br>▪ *Incube la culture en flacons Bact /Alert*<br>▪ *Envoi les cultures à l'INRB, Kinshasa* |

# 3. Procédures

## 3.1 Précautions

- Tous les échantillons de liquide céphalorachidien sont potentiellement infectieux. Respectez les précautions universelles. METTEZ DES GANTS À USAGE UNIQUE PENDANT TOUTE LA PROCÉDURE **!**

## 3.2 Matériel et échantillons

### 3.2.1 Matériel requis

- Gants, non stérile, à usage unique
- Masque respiratoire FFP3
- Flacon à hémoculture Bact/Alert pédiatique (capsule jaune)
- Alcool éthanol à 70%
- Seringue de 2 ml
- Aiguille 21G
- Système de transport de matériel biologique (triples emballages)

### 3.8.3 Echantillon à examiner

- Tube stérile de liquide céphalorachidien (LCR)
  Volume : 2 ml (pour un adulte) ou 1 ml (pour un enfant) (voir SOP-WP2-CLIN-03)
  *Stabilité* : Inoculez le milieu dans l'heure qui suit le prélèvement de LCR.

## 3.9 Mode opératoire

### 3.3.1 Inoculation du milieu Bact/Alert

15. Identifiez le flacon de milieu Bact/Alert pédiatrique.
16. Décollez le code-bare du flacon et collez-le sur la fiche hémoculture CRF laboratoire.
17. Retirez la capsule jaune du flacon Bact/Alert pédiatrique.
18. Désinfectez le bouchon en caoutchouc du flacon à l'alcool éthanol à 70%.
19. Déposez le flacon Bact/Alert sur son portoir.
20. Laissez sécher le bouchon 1 minute.
21. Attachez une aiguille verte 21G sur une seringue de 2 ml.
22. Homogénéisez l'échantillon de LCR.
23. Aspirez 2 ml (pour un adulte) ou 1 ml (pour un enfant) de LCR du tube stérile.

24. Injectez le LCR à travers le bouchon du flacon Bact/Alert pédiatrique.
25. Inscrivez les données dans le CRF laboratoire.
26. Incubez le flacon Bact/Alert pédiatrique à 37 °C jusqu'à l'envoi sur Kinshasa

**NE JAMAIS RÉFRIGÉRER !**

### 3.3.2 Contrôle de croissance

- Contrôlez avant de préparer les flacons Bact/Alert pédiatriques pour l'envoi si la couleur de l'indicateur colorimétrique au fond du flacon a virée.
- S'il n'y a pas de virage : continuez avec « 3.1.3 Préparation pour l'envoi »
- S'il y a un virage:
  - Etiquetez un deuxième flacon Bact/Alert pédiatrique avec le même numéro d'échantillon que le premier flacon.
  - Inoculez stérilement 1 ml du bouillon du premier flacon dans le deuxième flacon. Notez sur la deuxième flacon que c'est un ensemencement du premier flacon (avec un symbole ou une signe).
  - Faites une coloration de Gram sur une étalement du bouillon du premier flacon.
- Préparez les deux flacons Bact/Alert pédiatriques pour l'envoi comme décrit ci-dessous.

### 3.3.3 Préparation pour l'envoi

3. Emballez les flacons en triple emballage. Envoyez le triple emballage pour cultures et identification à l'INRB (Kinshasa). Un envoi par semaine.
   Le transport se fait à température ambiante et peut durer 1 semaine sans problème.
   **NE JAMAIS RÉFRIGÉRER !**

## 4. Enregistrements et archives

| **Appendices & formulaires à compléter** | |
|---|---|
| **Numéro** | **Titre** |
| 1 | CRF "laboratoire" |

## 6. Histoire de document

| **Révision** | |
|---|---|
| SOP-WP2-LAB-40-V01-17Sep2012 | Version initiale |
| SOP-WP2-LAB-40_V02-27Dec2012 | Addition de la partie "3.1.2 Contrôle de croissance » |

| **Nom et fonction** | **Date** | **Signature** |
|---|---|---|
| *Auteur* | | |
| Barbara Barbé | 27/12/2012 | |
| *Révisé par* | | |
| Philippe Gillet | 28/12/2012 | |
| *Approuvé par* | | |
| Emilie Alirol | 02/01/2013 | |

| | |
|---|---|
| | **SOP titre :** *Culture des mycobactéries du LCR en milieu BBL MGIT (BD)* additionné du supplément d'enrichissement OADC, *et d'un complexe d'antibiotiques PANTA* |
| | **Projet/Étude :** Evaluation de tests rapides en association avec des prédicteurs cliniques et de laboratoire pour le diagnostic de maladies tropicales négligées dans des patients se présentant avec des troubles neurologiques dans les hôpitaux ruraux au Bandundu, République Démocratique du Congo. |

# 1. Domaine et application

*Ce document donne les instructions pour la mise en culture du liquide céphalo rachidien (LCR) en milieu BBL MGIT. Ce milieu permet la culture des mycobactéries et contient un indicateur de croissance fluorescent. Après inactivation et décontamination, la croissance des mycobactéries sera confirmée par analyse moléculaire (système GenExpert au laboratoire de MSF à Kinshasa).*

# 2. Responsabilités

| Fonction | Activités |
|---|---|
| *Technicien de laboratoire* | ▪ *Met en culture*<br>▪ *Suit et interprète la croissance bactérienne*<br>▪ *Décontamine les milieux de culture à la fin de l'incubation*<br>▪ *Envoie les milieux de culture sur Kinshasa pour analyse moléculaire*<br>▪ *Enregistre les résultats* |

# 3. Procédures

## 3.1 Précautions

- Tous les échantillons LCR sont potentiellement infectieux. Respectez les précautions universelles. PORTEZ DES GANTS À USAGE UNIQUE PENDANT TOUTE LA PROCÉDURE **!**
  METTEZ UN MASQUE RESPIRATOIRE FFP3 PENDANT TOUTE LA PROCÉDURE **!**
- **N'OUVREZ JAMAIS UN TUBE MGIT APRES AVOIR MIS LE LCR EN CULTURE !**
  Le tube ne pourra être ouvert qu'après double décontamination (2 autoclavage pendant 30 minutes à 121°c, une fois à Mosango avant transport en triple emballage vers Kinshasa et une seconde fois au laboratoire MSF de Kinshasa avant analyse moléculaire).

## 3.2 Matériel et échantillons

### 3.2.1 Matériel fourni et conservation

- <u>Tube avec indicateur de croissance mycobactérienne BBL MGIT</u>™
  Contenant : 110 µl d'un indicateur fluorescent et 4 ml de bouillon. (réf BD 245113).
  Conservez entre 2 et 25°C. **NE PAS CONGELER**
- Brochure explicative

### 3.2.2 Matériel supplémentaire requis

- <u>Supplément nutritif MGIT™ OADC,</u> 15 ml, carton de 6 flacons.
  Chaque flacon est suffisant pour 25 tubes ***MGIT*** (réf BD  245116). Prêt à l'emploi.
  Conservez entre 2 et 8°C.  Minimisez le contact à la lumière. Evitez la congélation.

- Complexe d'antibiotiques MGIT™ PANTA™, lyophilisé, carton of 6 flacons. Chaque flacon est suffisant pour 25 tubes ***MGIT*** (réf BD : 245114). A reconstituer avant emploi.
  Conservez entre 2 et 8 °C. Après reconstitution, conservez les aliquots à -20°C pour une période de 6 mois maximum (ou jusqu'à la date de péremption, si elle est plus courte).
- Seringue de 2ml + aiguille
- Pipete Pasteur stérile
- Eau distillée
- Autoclave
- Papier indicateur de stérilisation (pour stérilisation à la vapeur)
- Lampe UV (longueur d'onde de 365 nm)

#### 3.2.3 Echantillon à examiner

Tube stérile de liquide céphalorachidien (LCR)
Volume : 2 ml (pour un adulte ou pour un enfant) (voir SOP-WP2-CLIN-03)
*Stabilité* : Inoculez le milieu dans l'heure qui suit le prélèvement de LCR.

### 3.3 Mode opératoire

#### 3.3.1 Avant l'emploi

Vérifiez l'aspect macroscopique du milieu MGIT. Détruisez le milieu s'il présente des signes de contamination ou si le tube est endommagé.

#### 3.3.2 Préparation du contrôle positif et négatif

- Contrôle négatif : Tube mégit scellé et non utilisé
- Contrôle positif :
  1. Diluer 0.02 g de sulfite de sodium (tubes pré-préparés) dans 5 ml d'eau injectable.
  2. Videz le bouillon d'un tube MGIT.
  3. Etiquetez le tube comme contrôle positif et notez la date de préparation.
  4. Ajoutez 5 ml de la solution de sulfite de sodium au tube MGIT vide.
  5. Laissez reposer le tube au moins une heure à température ambiante avant de l'utiliser.
  6. **Conserver le tube à température ambiante**.
  7. Le tube ainsi préparé peut être utilisé pendant 4 semaines (si conservé à température ambiante).

#### 3.3.3 Reconstitution de la PANTA

1. Désinfectez le bouchon en caoutchouc du flacon de PANTA à l'alcool éthanol à 70%.
2. Désinfectez le bouchon d'une ampoule d'eau injectable à l'alcool éthanol à 70%.
3. Ajoutez aseptiquement au flacon PANTA (avec une seringue de 4 ml) 3 ml d'eau injectable.
4. Dissolvez le contenu par agitations occasionnelles pendant 10 minutes
5. Etiquetez 20 cryotubes (PANTA reconstitué, date de préparation, date d'expiration)
6. Distribuer (à l'aide d'une seringue de 1 ml) 3 gouttes de PANTA dans les 20 cryotubes.
7. Conservez les aliquots à -20°C pour une période de 6 mois maximum (ou jusqu'à la date de péremption, si elle est plus courte).

### 3.3.4 Préparation du milieu MGIT avant inoculation

1. Laissez revenir le milieu MGIT, le supplément OADC et la Panta reconstituée à température ambiante.
2. Ajoutez aseptiquement au tube MGIT avec une seringue de 2ml, 0.5 ml de la solution OADC.
3. Ajoutez aseptiquement au tube MGIT ave une pipette pasteur stérile 2 gouttes (100µl) de La solution de PANTA reconstituée.
4. Mélangez vigoureusement le tube.

### 3.3.5 Inoculation du milieu MGIT

27. Identifiez le flacon de milieu.
28. Ajoutez du papier collant sur l'étiquette pour évitez qu'elle ne se décolle.
29. Aspirez à l'aide d'une seringue 2 ml (pour un adulte ou un enfant) stérilement de LCR du tube stérile.
30. Distribuez aseptiquement les 2 ml de LCR dans le tube MGIT.
31. Fermez le tube hermétiquement.
32. Mélangez vigoureusement le tube.
33. Inscrivez les données dans le CRF laboratoire.
34. Incubez le milieu à 37°C pour un maximum de 5 semaines.

**NE JAMAIS RÉFRIGÉRER !**

### 3.3.6 Lecture de la croissance en tubes MGIT

1. Lisez une fois par semaine (chaque lundi) les tubes : Retirez les tubes de l'incubateur, placez les tubes sous la lampe UV à côté du contrôle positif et du contrôle négatif.
   - Le tube de contrôle positif doit montrer une fluorescence intense de couleur orange vif intense.
   - Le tube de contrôle négatif ne doit pas montrer qu'une légère fluorescence ou aucune fluorescence.
   - Les tubes mis en culture sont positifs (croissance bactérienne) s'ils montrent une fluorescence orange vive dans le fond du tube se rapprochant de celle observée pour le contrôle positif. Les tubes positifs peuvent aussi présenter une turbidité non homogène ou des petits grains ou des flocons dans le milieu de culture.
2. Reportez les résultats dans le CRF pour les tubes positifs. Placez les tubes positifs à part pour être stérilisé le lundi après-midi (avant envoi en triple emballage le mardi sur Kinshasa).
3. Suivez le virage des tubes durant 5 semaines.
4. Après 5 semaine, tous les tubes qui n'ont pas viré, correspondant aux patients dont le numéro d'inclusion finit par 5 sont aussi envoyé à Kinshasa (après autoclavage, 30 minutes à 121°C) pour contrôle de qualité. Si le tube dont le numéro d'inclusion finissant par 5 est positif, prenez le tube suivant finissant par 6). Placez les tubes négatifs pour contrôle de qualité à part pour être stériliser le lundi après-midi (avant envoi en triple emballage le mardi sur Kinshasa).
5. Après 5 semaines, les tubes qui n'ont pas montré de virage peuvent être éliminés (après autoclavage, 30 minutes à 121°C).

### 3.3.7 Préparation pour l'envoi

4. Collez sur le bouchon de chaque tube, un petit morceau d'indicateur de stérilisation (à la vapeur).
5. Décontaminez les tubes MGIT (autoclavez les tubes 30 minutes à 121°C).
6. Vérifiez si l'indicateur de stérilisation est coloré en brun.
7. Emballez les tubes en triple emballage.

8. Envoyez le triple emballage pour analyse moléculaire à l'INRB (Kinshasa). Un envoi par semaine (le mardi).
Le transport se fait à température ambiante. **NE JAMAIS RÉFRIGÉRER**

### 3.4 Déchets, nettoyage

- Jetez les seringues et les aiguilles dans un conteneur« bio-hazard ».
- Eliminez les milieux MGIT (après autoclavage dans un conteneur « bio-hazard ».

## 4. Définitions & abréviations

- LCR : Liquide céphalorachidien
- MGIT : Mycobacteria Growth Indicator Tube
- OADC : supplément d'enrichissement
- PANTA : complexe d'antibiotiques

## 5. Enregistrements et archives

| **Appendices & formulaires à compléter** | |
|---|---|
| **Numéro** | **Titre** |
| 1 | CRF "laboratoire" |

## 7. Historique du document

| **Révision** | |
|---|---|
| | Version initiale |

| **Nom et fonction** | **Date** | **Signature** |
|---|---|---|
| *Auteur* | | |
| Philippe Gillet | 09/09/2012 | |
| *Révisé par* | | |
| Barbara Barbé | 13/09/2012 | |
| *Approuvé par* | | |
| Veerle Lejon | 17/Sept/2012 | |

| | |
|---|---|
| | **SOP titre** : Différenciation leucocytaire sur frottis de LCR, coloré au Giemsa |
| | **Projet/Étude :** Evaluation de tests rapides en association avec des prédicteurs cliniques et de laboratoire pour le diagnostic de maladies tropicales négligées dans des patients se présentant avec des troubles neurologiques dans les hôpitaux ruraux au Bandundu, République Démocratique du Congo. |

# 1. Domaine et application

- *Ce document donne les instructions pour faire la différentiation leucocytaire sur un frottis de LCR coloré au Giemsa. Les 3 types principaux de leucocytes Neutrophiles, Lymphocytes et Eosinophiles) qu'on peut retrouver dans le LCR se différencient par la taille, la forme du noyau, la couleur des granulations du cytoplasme et d'autres facteurs. La répartition entre les différents types est importante pour le diagnostic du type de méningite. 50 leucocytes sont comptés et le nombre de chaque type est noté. Ensuite la proportion de chaque type est calculée en %.*

# 2. Responsabilités

| Fonction | Activités |
|---|---|
| *Technicien de laboratoire* | ▪ *Prélève le LCR*<br>▪ *Pré-traite l'échantillon*<br>▪ *Exécute le test*<br>▪ *Interprète le test*<br>▪ *Enregistre les résultats* |

# 3. Procédures

## 3.1 Précautions

- Tous les échantillons de LCR sont potentiellement infectieux. Respectez les précautions universelles.
  METTEZ DES GANTS À USAGE UNIQUE PENDANT TOUTE LA PROCÉDURE **!**
  METTEZ UN MASQUE RESPIRATOIRE FFP3 PENDANT TOUTE LA PROCÉDURE **!**
- Tous les alcools sont inflammables, manipulez ces produits loin d'une flamme.

## 3.2 Matériel

### 3.2.1 Matériel requis

- Gants, non stérile, à usage unique
- Lame porte-objet neuve
- Crayon diamant
- Anse de platine
- Flacon contenant un mélange de sable et de désinfectant.
- Lampe à alcool
- Méthanol
- Solution de Giemsa concentrée
- Eau taponnée.
- Pipettes pasteur
- Support de coloration des lames
- Support sèche lame
- Microscope (objectif 100 x)
- Huile à immersion
- Minuterie

- Compteur 5 touches

#### 3.2.2 Echantillon à examiner

LCR prélevé depuis moins d'une heure: une goutte du culot de centrifugation du LCR (10 minutes à 3000 rpm, soit pour la centrifugeuse AML A8 en position 8).

### 3.3 Procédure

96. Notez au crayon diamant le numéro du patient sur une lame neuve.
97. Flambez la lame en la passant 3 x sur la flamme d'une lampe à alcool.
98. Décontaminez l'anse de platine en l'immergeant dans le flacon contenant un mélange de sable et de désinfectant.
99. Flambez l'anse de platine (porter le fil au rouge sur toute sa longueur) puis laissez-la refroidir.
100. Etalez à l'aide de l'anse de platine une goutte du culot de centrifugation du LCR sur la lame flambée et faites un frottis aussi mince que possible.
101. Décontaminez l'anse de platine en l'immergeant dans le flacon contenant un mélange de sable et de désinfectant.
102. Re-flambez l'anse de platine (porter le fil au rouge) pour détruire les bactéries.
103. Laissez sécher à l'air, à l'abri des insectes.
104. Recouvrez la lame de méthanol pur pour fixer le frottis.
105. Eliminez le méthanol et laisser sécher.
106. Déposez la lame fixée sur un support de coloration (des barres en position horizontales). Si plusieurs lames sont colorées en même temps, ne les laissez pas se toucher.
107. Colorez la lame au giemsa dilué comme pour une goutte épaisse, voir sop nr SOP-WP2-LAB-20.
108. Observez la lame au microscope (à l'huile d'immersion, objectif 100 x, oculaire 10 x)
109. Différentiez les 50 premiers globules blancs observés entre Neutrophiles, Lymphocytes et Eosinophiles. Utilisez un compteur à 5 touches pour compter les cellules.

### 3.4 Rendu des résultats

En % : multipliez le nombre de chaque type de globules blancs comptés sur 50 cellules par 2 pour obtenir le résultat en %

### 3.5 L'interprétation des résultats

- On retrouve un nombre accru de leucocytes dans les maladies suivantes :
  - Méningite bactérienne → principalement des neutrophiles
  - Méningite tuberculeuse et méningite virale → principalement des lymphocytes
  - Trypanosomiase africaine → surtout des lymphocytes
  - Cryptococcose → variable
  - Paludisme cérébrale → souvent normal ou légère lymphocytose

## 4. Enregistrements et archives

| Appendices & formulaires à compléter | |
|---|---|
| **Numéro** | **Titre** |
| 1 | CRF laboratoire |

## 5. Références


- Bactériologie médicale tropical. Notes pratiques. Institut de Médicine Tropicale Prince Léopold, Philippe Gillet, Luc Boel, Jan Jacobs, Septembre 2009.
  *http://www.labquality.be/documents/ANALYSIS/BACTERIOLOGY/090803%20Notes%20pratiques%20de%20Bactériologie%20Médicale.pdf*
- Manuel des techniques de base pour le laboratoire médical, OMS, 1982
  *http://www.labquality.be/documents/ANALYSIS/9242541451.pdf*
- Manual of basic techniques for a health laboratory, 2nd edition, WHO, 2003
  *http://www.labquality.be/documents/ANALYSIS/9241545305.pdf*


## 6. Histoire de document

| **Révision** | |
|---|---|
| | Version initiale |

| **Nom et fonction** | **Date** | **Signature** |
|---|---|---|
| *Auteur* | | |
| Barbara Barbé | 17/07/2012 | |
| *Révisé par* | | |
| Philippe Gillet | 10/09/2012 | |
| *Approuvé par* | | |
| Veerle Lejon | 17/Sep/2012 | |

| | |
|---|---|
| nidiag<br>Better Diagnosis of Neglected Infectious Diseases | **SOP titre** : Recherche de trypanosomes à frais dans le liquide ganglionnaire |
| | **Projet/Étude :** Evaluation de tests rapides en association avec des prédicteurs cliniques et de laboratoire pour le diagnostic de maladies tropicales négligées dans des patients se présentant avec des troubles neurologiques dans les hôpitaux ruraux au Bandundu, République Démocratique du Congo. |

# 1. Domaine et application

Dans la THA, on trouve les trypanosomes dans les ganglions lymphatiques au début de la maladie, 2-3 semaines après la transmission du parasite par la mouche tsé-tsé. Ce document donne les instructions pour la recherche d'un ganglion lymphatique, le prélèvement du liquide ganglionnaire, la préparation des lames et l'examen microscopique (selon l'OMS). Une goutte de suc ganglionnaire est prélevée à l'aiguille. Elle est examinée aussitôt à l'état frais entre lame et lamelle. Les trypanosomes (protozoaires flagellés mobiles) y sont facilement retrouvés au microscope sur base de leur mouvement.

# 2. Responsabilités

| Fonction | Activités |
|---|---|
| Technicien de laboratoire | ▪ Prélève le liquide ganglionnaire<br>▪ Prépare les lames<br>▪ Examine la préparation au microscope<br>▪ Enregistre les résultats |

# 3. Procédures

## 3.1 Précautions

- Tous les échantillons de liquide ganglionnaire sont potentiellement infectieux. Respectez les précautions universelles. METTEZ DES GANTS À USAGE UNIQUE PENDANT TOUTE LA PROCÉDURE **!**

## 3.2 Matériel et échantillons

### 3.2.1 Matériel requis

- Gants, non stérile, à usage unique
- Aiguille 21G sèche
- Seringue de 2 ou 5 ml sèche
- Lame de microscope
- Lamelle
- Tampons d'éthanol 70%
- Conteneur à aiguilles
- Microscope, objectif 10x et 40x

### 3.2.2 Echantillon à examiner

- Liquide ganglionnaire : quelques gouttes
- Exécutez le test immédiatement ! (Les trypanosomes perdent leur mobilité après <30 minutes)

## 3.3 Mode opératoire

### 3.3.1 Recherche d'un ganglion lymphatique

Les ganglions lymphatiques se situent dans la chaîne cervicale des ganglions du cou.

1. Palpez les 2 côtés du cou, depuis la base du cou jusqu'au niveau de l'oreille.

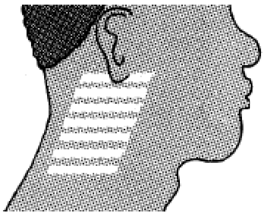

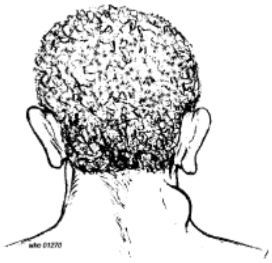

2. Caractéristiques des ganglions atteints :
    - gonflés
    - forment une petite masse ronde de 2-4 cm de large
    - élastiques, glissent sous la peau, résistant peu à la pression
    - non indurés (sauf chez les malades chroniques)

### 3.3.2 Prélèvement du liquide ganglionnaire

1. Mettez des gants à usage unique.
2. Préparez et identifiez une lame.
3. Faire asseoir le malade.
4. Désinfectez l'endroit choisi à l'éthanol 70%.
5. De la main gauche, saisissez le ganglion entre pouce et l'index.
6. Maintenez le ganglion fixe et faites-le ressortir.
7. Entre pouce et le majeur de la main droite, introduisez l'aiguille bouchée par l'index perpendiculairement au centre du ganglion :
    - Pénétrez d'abord sous la peau
    - Puis, pénétrez dans le ganglion

   Attention !!! Ne pas atteindre les jugulaires ou les carotides !
8. De la main gauche, malaxez doucement le ganglion.

   De la main droite, faites pivoter l'aiguille sur elle-même.
9. Le suc ganglionnaire monte dans l'aiguille. L'opération doit durer environ 1 min.
10. Bouchez l'aiguille avec l'index et retirez l'aiguille du ganglion d'un mouvement rapide.
11. Posez un tampon d'éthanol 70% sur le point de ponction.

6

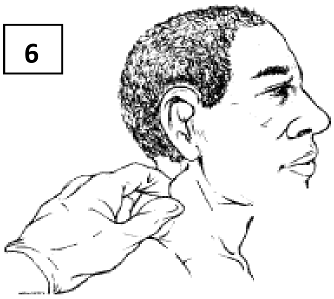

7

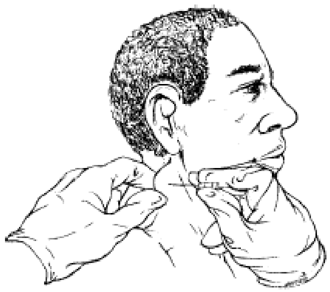

8

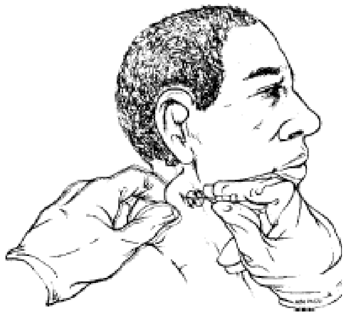

### 3.3.3 Préparation des lames

1. Tirez à fond le piston de la seringue.
2. Adaptez la seringue sur l'aiguille.
3. Poussez doucement le piston à mi-course et déposez une goutte de suc ganglionnaire sur la lame.
4. Jetez l'aiguille dans un conteneur à aiguilles.
5. Couvrez la lame d'une lamelle.
6. Examinez les lames aussitôt au microscope.

### 3.3.4 Examen microscopique

1. Examinez les lames d'abord à l'objectif 10x, puis à l'objectif 40x.
2. Attendez que les courants de liquide cessent dans la préparation.
3. Commencez par examiner les pourtours, près des bords de la lamelle. Les trypanosomes ont tendance à se diriger vers les bords.
4. Examinez le reste de la préparation :
    - La préparation contient des hématies, des leucocytes et des lymphocytes. Essayez de repérer un mouvement entre ces éléments et examinez attentivement pour déterminer s'il s'agit d'un trypanosome.
    - Caractéristiques du trypanosome :
        - Taille : 10 – 21 µm (= 2-3 hématies)
        - Forme : celle d'un poisson allongé et ondulant
        - Aspect (à frais) : clair, très réfringent
    - Les mouvements du flagelle sont surtout distingués à l'état frais. Celui-ci serpente en zigzaguant entre les globules.

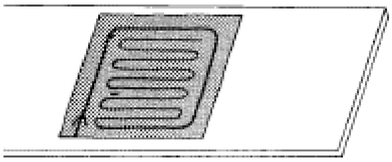

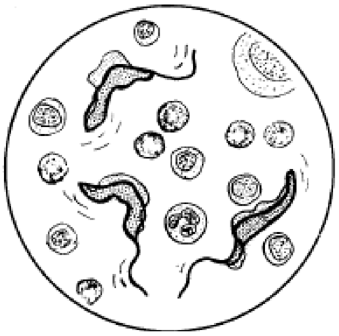

## 4. Enregistrements et archives

| Appendices & formulaires à compléter | |
|---|---|
| **Numéro** | **Titre** |
| 1 | CRF "laboratoire" |

## 5. Références


- OMS, Manuel des techniques de base pour le laboratoire médical, 1982.
- WHO, Manual of basic techniques for a health laboratory, 2nd edition, 2003.


## 6. Histoire du document

| Révision | |
|---|---|
| | Version initiale |

| **Nom et fonction** | **Date** | **Signature** |
|---|---|---|
| *Auteur* | | |
| Barbara Barbé | 17/08/2012 | |
| *Révisé par* | | |
| Philippe Gillet | 10/09/2012 | |
| *Approuvé par* | | |
| Veerle Lejon | 17/Sep/2012 | |

**SOP titre :** Prélèvement sanguin
(hémocultures, sérum, sang héparine et sang EDTA)

**Projet/Étude :** Evaluation de tests rapides en association avec des prédicteurs cliniques et de laboratoire pour le diagnostic de maladies tropicales négligées dans des patients se présentant avec des troubles neurologiques dans les hôpitaux ruraux au Bandundu, République Démocratique du Congo.

# 1. Terrain d'application

Cette procédure décrit les conditions de prélèvement des hémocultures, des tubes de sang et le prétraitement des échantillons sanguins.

# 2. Responsabilités

| Fonction | Activités |
|---|---|
| Technicien de laboratoire<br>Infirmier | ▪ Suit la procédure<br>▪ Réalise les prélèvements<br>▪ Prétraite les échantillons<br>▪ Identifie les échantillons |
| Médecins | ▪ Demande le prélèvement<br>▪ Supervise les prélèvements |

# 3. Procédures

## 3.1 Matériels et consommables

1. Flacon à hémoculture (BactAlert):
    - Adulte (≥14 ans) : 2 flacons à capsule verte Enfant (5ans – 13ans): 1 flacon à capsule jaune

3. Gants d'examen à usage unique (non-stériles)
4. Garrot
5. Compresses (non-stériles)
6. Alcool éthanol à 70%.
7. Povidone iodé à 10%
8. Aguilles épicraniennes (23G pour adult – 21G pour enfant) avec adaptateur vacutainer
9. 2 Adaptateur Vacutainer pour flacon à hémoculture(Nettoyé à l'alcool éthanol à 70%)
10. Réducteur pour tubes Vacutainer (Nettoyé à l'alcool éthanol à 70%)
11. Tubes Vacutainer sérum
12. Tubes Vacutainer héparine
13. Tubes Vacutainer EDTA
14. Pansement sec
15. Conteneur pour objets tranchants

## 3.2 Procédure

### 3.2.1 Remarques générales (voire aussi annexe 1)

- Prélevez les hémocultures <u>avant tout autre prélèvement de sang.</u>
- Prélevez toujours le sang avant l'administration des antibiotiques.

  *-> Pour des patients déjà sous antibiotiques, prélevez juste avant la prochaine administration d'antibiotiques.*

- Prélevez pour les

| <u>Adultes</u> (≥14 ans): | <u>Enfants</u> (5ans – 13ans): |
|---|---|
| - 2 sites de prélèvement différents | - 1 seul site de prélèvement |
| - 2 flacons d'hémoculture à capsule verte | - 1 seul flacon à capsule jaune |
| - 1 tube sérum (9 ml) | - 1 tube sérum (5 ml) |
| - 1 tube héparine (4 ml) | - 1 tube héparine (4 ml) |
| - 1 tube EDTA (6 ml) | - 1 tube EDTA (6 ml) |

- Ne jamais re-capuchonnez l'aiguille.

### 3.2.2 Procédure de prélèvement

1. Préparez tout le matériel et informez le patient.
2. Nettoyez-vous les mains avec un gel alcoolique ou à l'eau savonneuse.
3. Désinfectez la peau au point de prélèvement avec de l'alcool éthanol à 70%. Commencez par désinfecter le site de ponction choisi puis tout autour d'un mouvement circulaire.
4. Laissez sécher pendant 1 minute.
5. Désinfectez la peau au point de prélèvement avec de la povidone iodé à 10%.
6. Laissez sécher pendant 2 minutes. Entretemps continuez avec les points 7-13
   **Ne touchez plus le point de prélèvement !!**
7. Identifiez le flacon à hémoculture et le(s) tube(s)
8. Mettez le flacon à hémoculture dans un portoir et retirez la capsule.
   **Enfant: flacon à capsule jaune (1x)**
   **Adulte: flacon à capsule vert (2x)**
9. Désinfectez le capuchon du flacon avec de la povidone iodé à 10%.
10. Laissez sécher pendant 1 minute.
11. Mettez les gants.
12. Reliez l'épicrânienne à l'adaptateur Vacutainer pour flacons à hémoculture. Visez à fond.
13. Placez le(s) flacon(s) à hémoculture sur un support (table) près du point de prélèvement
14. Placez le garrot.
15. Piquez la veine choisie avec le dispositif de prélèvement
16. Placez l'adaptateur sur le flacon à hémoculture en introduisant l'aiguille dans le capuchon en caoutchouc en enfonçant flacon, retirez le **garrot dès** que le **sang** commence à couler.
17. Laissez entrer le sang jusqu'à 1 trait de 4 ml (Enfant) ou 2 traits de 5 ml (Adulte)
    Enfant (5ans – 13ans) 4 ml (1 trait)
    Adulte (≥ 14ans) 10 ml (2 traits)
    *Si vous ne réussissez pas à prélever le sang, utilisez un nouveau dispositif de prélèvement.*

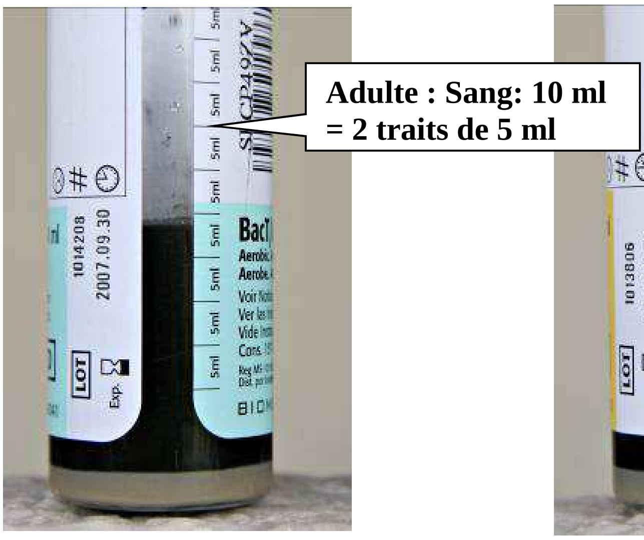


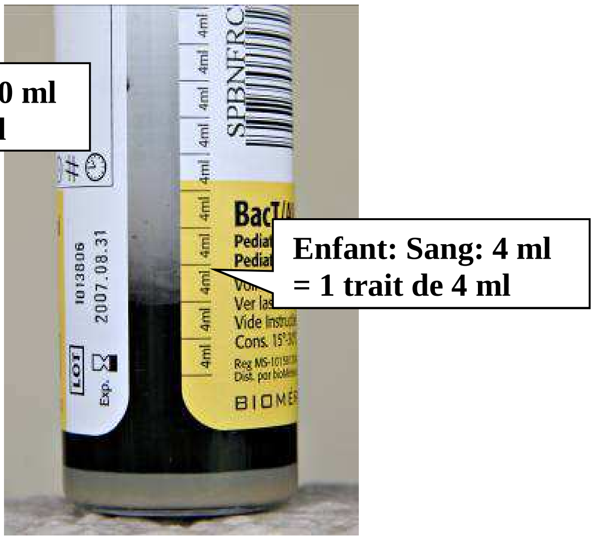

18. Retirez le flacon à hémoculture, ajoutez le réducteur pour tubes Vacutainer.
19. Insérez le tube Vacutainer sérum dans le réducteur et prélevez le tube sérum.

**Uniquement pour les enfants (5 ans – 13 ans):**

20. Enlevez le tube sérum et remplacez- le par un tube héparine (tube de 4 ml).
21. Enlevez le tube héparine et remplacez-le par un tube EDTA (tube de 6 ml).
22. Enlevez le tube EDTA

**Pour tous les patients :**

23. Retirez l'épicrânienne de la veine.
24. Déconnectez l'épicrânienne de l'adaptateur Vacutainer.
25. Jetez le système l'épicrânienne dans le conteneur pour objets tranchants.
26. Faites un pansement sec au point de piqûre.
27. Décollez le code barre du flacon à hémoculture et collez-le sur la fiche hémoculture CRF laboratoire.

**Uniquement pour les adultes :**

Recommencez la même procédure sur l'autre bras (voir points 3-17). *Utilisez un nouveau système de prélèvement (épicrânienne, adaptateur Vacutainer et réducteur), ensuite*

.

1. Insérez le tube Vacutainer héparine dans le réducteur et prélevez le tube héparine.
2. Enlevez le tube héparine et remplacez-le par un tube EDTA.
3. Enlevez le tube EDTA.
4. Retirez l'épicrânienne de la veine.
5. Déconnectez l'épicrânienne de l'adaptateur Vacutainer.
6. Jetez l'épicrânienne dans le conteneur pour objets tranchants.
7. Faites un pansement sec au point de piqûre.
8. Décollez le code barre du flacon à hémoculture et collez-le sur la fiche hémoculture CRF laboratoire.
9. Décontaminez l'adaptateur à l'alcool éthanol à 70%

### 3.2.3 Prétraitement des échantillons

Voir le document SOP-WP2-LAB-37 (liste des tests).

- Conservez les flacons à hémoculture dans l'incubateur à 37°C jusqu'à l'envoi une fois par semaine en triple emballage sur Kinshasa.
- Les tubes héparine et EDTA sont prêt à être analysés.
- Le tube sérum doit être centrifugé avant analyse :
  1. Laisser reposer le tube Vacutainer sérum pendant minimum 10 minutes.
  2. Centrifuger le tube 10 minutes à 3.000 rpm (centrifugeuse AML A8 en position 8).

## 4. Enregistrements et archives

| **Appendices & formulaires à compléter** | |
|---|---|
| **Numéro** | **Titre** |
| 1 | CRF laboratoire |
| Annexe 1 | Bonne pratique de prélèvement du sang |

| **Nom et fonction** | **Date** | **Signature** |
|---|---|---|
| *Auteur* | | |
| Barbara Barbé | 07/09/2012 | |
| *Révisé par* | | |
| Philippe Gillet | 09/09/2012 | |
| *Approuvé par* | | |
| Veerle Lejon | 18/Sep/2012 | |

**SOP-WP2-LAB-44-Annex 1**
**Bonne pratique de prélèvement du sang**

## 1. Ordre de prélèvement (selon CLSI H03-A6)

Pour éviter des erreurs de résultat ou/et le transfert d'un additif indésirable respectez toujours l'ordre de prélèvement suivant :

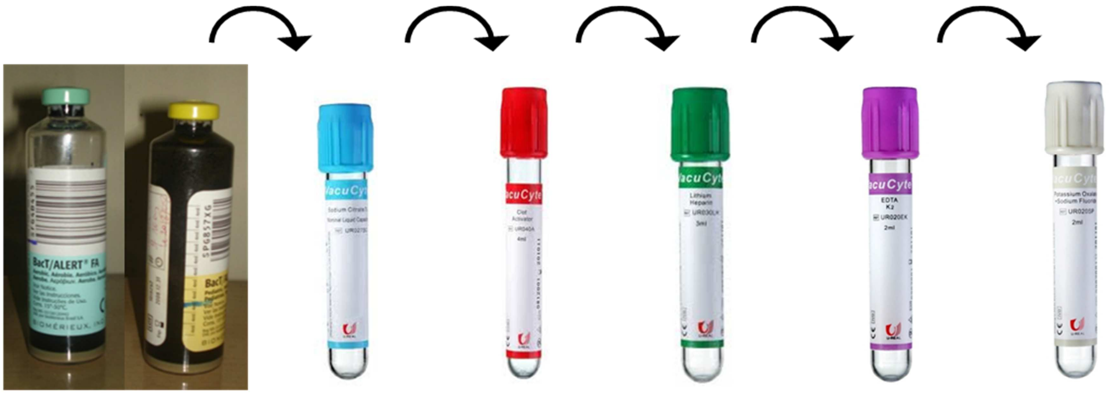

**1. Hémoculture** **2.Citrate (coagulation)** **3. Sérum** **4. Héparin** **5. EDTA** **6. Glycolytic solution (Fluoride)**

**Remarques :**

1. Les couleurs données pour les tubes avec additifs sont à titre d'exemple. Il n'existe pas de code couleur universel pour les différents additifs.

## 2. Sources fréquentes d'erreurs

| | |
|---|---|
| "pompage excessif" | => augmentation d'activité des muscles<br>=> faux résultats (K+, Mg+) |
| Touchez le site de ponction après désinfection | => contamination, risque infection |
| Trop serrez le garrot, laisser le garrot trop longtemps | => faux résultats (K+) |
| Mélangez insuffisamment le sang avec l'additif | => (micro) coagulation |
| Mélangez trop énergiquement le sang | => hémolyse |
| Conditions de prélèvement (à jeune, position patient..) | => faux résultats |
| Re-capuchonnez une aiguille | => risque d'accident et d'infection |

## 3. Sites inappropriés pour un prélèvement de sang

- Un membre paralysé
- Le côté où coule une perfusion
- A proximité d'une brûlure ou d'une plaie

## 4. Pourquoi ne pas prélever dans une veine sous perfusion

1. Pour protéger l'accès intraveineux
2. Pour éviter les interférences de la solution de perfusion.
   a. La solution intraveineuse peut diluer l'échantillon
   b. La concentration en antibiotique (si donner par intraveineuse) peut être élevée et empêcher la croissance des bactéries dans l'hémoculture.

**Comment procéder?**

1. Utilisez une autre veine.

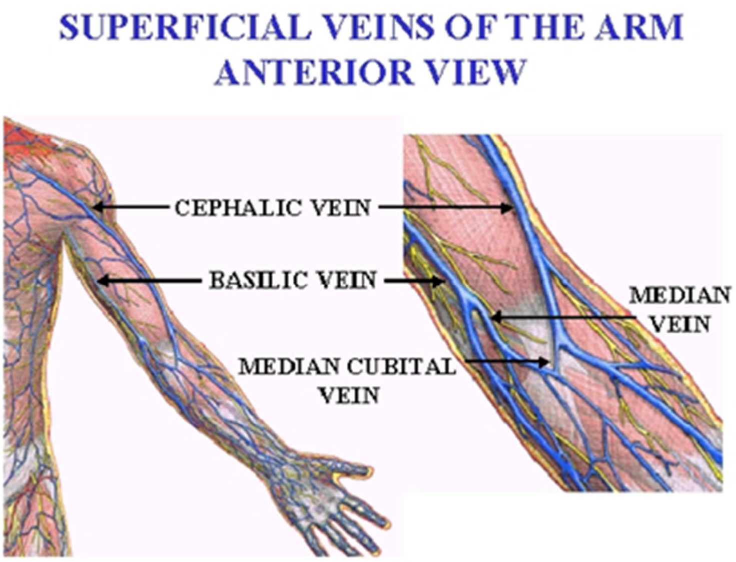


2. Si l'autre bras n'est pas accessible, effectuez les deux ponctions dans le même bras à 2 sites différents. Signalez sur la fiche : même bras, deux sites différents

3. Si aucune autre veine n'est accessible, utilisez un seul site de prélèvement. Signalez sur la fiche : 1 site pour le deux hémocultures. Ceci permettra de différencier des contaminants et des bactéries significatives.

| | |
|---|---|
| | **SOP titre :** Comptage des lymphocytes CD4+ (Dynal T4 Quant Kit/Invitrogen) |
| | **Projet/Étude** : Evaluation de tests rapides en association avec des prédicteurs cliniques et de laboratoire pour le diagnostic de maladies tropicales négligées chez les patients se présentant avec des troubles neurologiques dans les hôpitaux ruraux au Bandundu, République Démocratique du Congo. |

# 1. Domaine et application

*Ce document donne les instructions pour faire un comptage des lymphocytes CD4+ à l'aide du Dynal T4 Quant kit.*
*La séparation cellulaire immuno-magnétique permet un comptage rapide et direct des lymphocytes T CD4+. L'isolement des cellules se fait en 3 étapes et nécessite 30 minutes au total : déplétion des monocytes, séparation des lymphocytes CD4+ et lyse des lymphocytes CD4+ pour libérer les noyaux pour comptage.*
*Les Dynabeads sont des billes de polystyrène uniformes et super-paramagnétiques (4.5 µm de diamètre) couplées avec des anticorps monoclonaux de souris spécifiques dirigés respectivement, contre les antigènes CD14 des monocytes ou les antigènes CD4 des lymphocytes T CD4+. Les Dynabeads se lient à leurs cellules cibles respective ce qui permet de les isolées et de les séparées en utilisant un aimant.*

# 2. Responsabilités

| Fonction | Activités |
|---|---|
| *Technicien de laboratoire* | ▪ *suit la procédure*<br>▪ *prélève l'échantillon*<br>▪ *stocke les consommables correctement*<br>▪ *enregistre les données dans le CRF* |

# 3. Procédures

## 3.1 Précautions

- Tous les échantillons de sang sont potentiellement infectieux. Respectez les précautions universelles. METTEZ DES GANTS À USAGE UNIQUE PENDANT TOUTE LA PROCÉDURE **!**
- La solution de lyse contient du formaldéhyde : substance toxique et carcinogène ; irritante pour les yeux, le nez, la gorge et la peau ; peut entraîner des maux de tête. A UTILISER AVEC PRECAUTION !

## 3.2 Matériel et échantillons

### 3.2.1 Matériel fourni et conservation

Dynal T4 Quant Kit (suffisant pour 80 tests de 125 µl de sang total) :

- o Dynabeads CD14 (étiquette bleue) – 2 ml : couplées avec des anticorps monoclonaux de souris spécifiques des antigènes CD14 de monocytes
- o Dynabeads CD4 (étiquette jaune) – 2 ml : couplées avec des anticorps monoclonaux de souris spécifiques des antigènes CD4 de lymphocytes T CD4+
- o Solution de lyse – 7 ml (A utiliser avec précaution !!)

*Conservation* : à 2-8°C, en évitant toute contamination bactérienne. NE PAS CONGELER ! Gardez les Dynabeads dans leur suspension pendant leur stockage et manipulation.

### 3.2.2 Matériel supplémentaire requis

- Gants, non stérile, à usage unique

- Tampon de lavage/dilution: Tampon phosphate salin isotonique (PBS) additionné de BSA et d'azide de sodium, pH 7.4 :
    - 0.16 g $NaH_2PO_4 . H_2O$ (qualité analytique)
    - 0.98 g $Na_2HPO_4 . 2H_2O$ (qualité analytique)
    - 8.10 g NaCl (qualité analytique)
    - 1 litre d'eau distillée
    - 0.1% (p/v) sérum albumine bovine (BSA)
    - 0.02% azide de sodium
- Une bouteille en verre d'une litre
- Pipette 50 µl, 200 µl, 1000 µl
- Embouts jaunes et bleus
- Micro-tubes coniques
- Portoir de micro-tubes
- Agitateur rotatif permettant l'inclinaison et la rotation des tubes en même temps (Dynal MX1 ou similaire)
- Portoir aimanté (Dynal MPC$^{TM}$-S)
- Solution de coloration de Türk :
    - 100 ml d'eau distillée
    - 3 ml d'acide acétique glacial ($CH_3COOH$)
    - 1 ml de violet de gentiane 1% ou 1 ml du bleu de méthylène aqueux
- Vortex
- Cellule de numération de Neubauer Improved
- Lamelle plane
- Parafilm
- Papier buvard
- Minuterie
- Microscope optique, objectif 40X
- Conteneur « Bio-Hazard »

### 3.2.3 Echantillon à examiner

- Sang total sur EDTA : 150 µl
  *Conservation*: à température ambiante ou à 2-8°C. Exécutez le test au maximum 24 heures après le prélèvement.

## 3.3 Mode opératoire

### 3.3.1 Contrôle de qualité

#### 3.3.1.1 Contrôle interne recommandé

Pour chaque premier patient du mois, réalisez 3 fois le même échantillon. La reproductibilité est fondamentale, puisque le but du comptage est de suivre les variations du nombre de lymphocytes T CD4+. Le coefficient de variation intra-individuel (CV% = écart-type/moyenne) doit être inférieur à 10%.

### 3.3.2 Procédure

#### 3.3.2.1 Préparation des réactifs :

1. Préparation du tampon de lavage/dilution PBS, pH 7.4 :
    - Prenez 0.16 g $NaH_2PO_4$ x $H_2O$ dans la bouteille d'une litre
    - Ajoutez 0.98 g $Na_2HPO_4$ x $2H_2O$ et 8.10 g NaCl
    - Ajoutez 1 litre d'eau distillée
    - Ajoutez 0.1% (p/v) de sérum albumine bovine
    - Pour stockage à long terme : ajoutez 0.02% d'azide de sodium (concentration finale)

2. Préparation du liquide de Türk:
   - Versez 96 ml d'eau distillé dans un flacon
   - Ajoutez 3 ml d'acide acétique glacial
   - Ajoutez 1 ml de violet de gentiane à 1% (ou 1 ml de bleu de méthylène aqueux)

   NE verser JAMAIS d'eau dans l'acide acétique glacial, car cela peut produire suffisamment de chaleur pour faire exploser la bouteille.
   Stockez dans une bouteille ambrée à 2 – 8°C pendant 3 mois.

#### 3.3.2.2 Préparation des échantillons sanguins et déplétion monocytaire

1. Sortiez les flacons de Dynabeads du réfrigérateur. Laissez-les revenir à température ambiante.
2. Mettez les tubes de sang sous agitation pendant 5 minutes sur l'agitateur rotatif Dynal MX1 (vitesse moyenne) à température ambiante.
3. Mettez des gants à usage unique.
4. Identifiez une première série T1 de micro-tubes Dynal avec le numéro de patient.
5. Remettez les Dynabeads CD14 (étiquette bleue) en suspension avant de les utiliser.
6. Ajoutez à l'aide d'une pipette dans chaque micro-tube Dynal T1 identifié :
   - 350 µl de PBS
   - 125 µl de sang total sur EDTA du patient
     Remarques : - Essuyez l'embout de la pipette rempli avec un papier buvard avant de déposer le sang total dans le micro-tube.
     - Rincez bien l'embout dans le tampon PBS (mouvements de « va et vient »)
   - 25 µl de Dynabeads CD14 (étiquette bleue)
     Remarque : Rincez bien l'embout (mouvement de « va et vient »)
7. Pliez des morceaux de Parafilm en 3 et mettez-les entre les micro-tubes T1 et leurs capuchons. Refermez bien les micro-tubes T1.
8. Agitez doucement les micro-tubes T1 à la main pendant 10 secondes (par retournements).
9. Mettez les micro-tubes T1 sur l'agitateur rotatif Dynal MX1 (vitesse moyenne) pendant 10 minutes à température ambiante.
10. Récupérez les micro-tubes T1.
11. Enlevez doucement les bouchons. Faites retomber les gouttelettes de sang restant sur le Parafilm. Jetez ensuite les morceaux de Parafilm dans un conteneur « Bio-Hazard ».
12. Re-suspendez les micro-tubes T1.
13. Placez les micro-tubes T1 sur le portoir aimanté Dynal MPC-S pendant 3 minutes. Laissez les micro-tubes T1 sur le portoir aimanté après l'incubation.
    Remarque : Si l'incubation prend plus de 5 minutes, resuspendez doucement le surnageant avant de le transférer.
14. Le surnageant de micro-tube T1 contient du sang démonocyté.

#### 3.3.2.3 Séparation des lymphocytes CD4+

1. Identifiez une deuxième série T2 de micro-tubes Dynal avec le numéro de patient.
2. Remettez les Dynabeads CD4 (étiquette jaune) en suspension avant de les utiliser.
3. Ajoutez dans chaque micro-tube T2 correspondant :
   - 200 µl de tampon PBS
   - 200 µl de sang démonocyté (= le surnageant du micro-tube T1)
     Remarque : - Gardez les micro-tubes T1 sur le portoir aimanté pendant le prélèvement de sang démonocyté.
     - Pipetez du côté opposé de l'aimant pour éviter d'aspirer les monocytes concentrés par l'aimant sur la paroi du tube.
     - Rincez bien l'embout dans le tampon PBS (mouvements de « va et vient »)

- o 25 µl de Dynabeads CD4 (étiquette jaune)
  Remarque : Rincez bien l'embout (mouvements « va et vient »)

4. Jetez les micro-tubes T1 contenant des rosettes de monocytes (= Dynabeads CD14 qui sont liés à des monocytes).
5. Pliez des morceaux de Parafilm en 3 et mettez-les entre les micro-tubes T2 et leurs capuchons. Refermez bien les micro-tubes T2.
6. Agitez doucement les micro-tubes T2 à la main pendant 10 secondes (par retournements).
7. Mettez les micro-tubes T2 sur l'agitateur rotatif Dynal MX1 (vitesse moyenne) pendant 10 minutes à température ambiante.
8. Récupérez les micro-tubes T2.
9. Enlevez doucement les bouchons. Faites retomber les gouttelettes de sang restant sur le Parafilm. Jetez ensuite les morceaux de Parafilm dans un conteneur « Bio-Hazard ».
10. Resuspendez les micro-tubes T2.
11. Placez les micro-tubes T2 sur le portoir aimanté Dynal MPC-S pendant 3 minutes. Laissez les micro-tubes T2 sur le portoir aimanté après l'incubation.
12. Les micro-tubes T2 contiennent des rosettes de lymphocytes T CD4+ (= Dynabeads CD4 qui sont liés à des lymphocytes T CD4+).
13. Lavage des rosettes:
    - o Eliminez le surnageant par 3 pipetages de 200 µl.
      Remarque : Pipetez sur la paroi opposée pour éviter d'aspirer le complexe Dynabeads-cellules.
    - o Retirez les micro-tubes T2 du portoir aimanté.
    - o Distribuez rapidement 500 µl de tampon PBS dans chaque micro-tube T2 (du côté complexe Dynabeads-cellules)
    - o Placez un morceau de Parafilm triplé sur chaque micro-tube T2. Refermez chaque micro-tube T2.
    - o Agitez doucement les micro-tubes T2 à la main pendant 10 secondes (par retournements).
    - o Retirez les capuchons et les morceaux de Parafilm. Jetez-les dans un conteneur « Bio-Hazard ».
    - o Placez les micro-tubes T2 sur le portoir aimanté Dynal MPC-S pendant 3 minutes.
      Laissez les micro-tubes T2 sur le portoir aimanté après l'incubation.
    - o Eliminez le surnageant par 3 pipetages de 200 µl.
14. Faites une seconde étape de lavage.

### 3.3.2.4 Comptage des lymphocytes CD4+

1. Récupérez les micro-tubes T2.
2. Distribuez rapidement 50 µl de solution de lyse dans chaque micro-tube T2 (du côté cellules).
3. Agitez au vortex pendant 10 secondes.
4. Laissez reposer pendant 5 minutes.
5. Agitez au vortex pendant 10 secondes.
6. Ajoutez 50 µl de liquide de Türk dans chaque micro-tube T2.
7. Agitez au vortex pendant 10 secondes.
8. Laisser reposer pendant 2 minutes.
9. Resuspendez bien les micro-tubes T2.
10. Déposez 15 µl de chaque micro-tube T2 dans une cellule de Neubauer improved montée (lamelle plane).
11. Laissez reposer pendant 1 minute.
    Remarques : Si la lecture des noyaux sous microscope ne peut être faite immédiatement, conservez les micro-tubes T2 au maximum 1 heure à 2-8°C avant la lecture.

12. Placez sous un microscope optique (objectif 40 x) :
    Les noyaux de lymphocytes T CD4+ apparaissent sous forme de points jaune-vert sur un fond violet.
13. Comptez l'ensemble des noyaux dans les 4 grands carrés aux extrémités de la cellule Neubauer improved.
14. Lymphocytes T CD4+/ µl de sang total = le nombre total de noyaux comptés multiplié par 5.

### 3.4 Interprétation des résultats

Valeurs de référence : > 200 lymphocytes T CD4+/µl de sang

## 4. Enregistrements et archives

| **Appendices & formulaires à compléter** | |
|---|---|
| **Numéro** | **Titre** |
| 1 | Cahier de laboratoire |

## 5. Références

- Brochure d'utilisation du fabricant (08-11-10, version 15.41.12)
- Protocole Dynal T4 Quant Kit, Dynal Biotech, préparé par Nicolas Speranza.

## 6. Histoire de document

| **Révision** | |
|---|---|
| | Version initiale |

| **Nom et fonction** | **Date** | **Signature** |
|---|---|---|
| *Auteur* | | |
| Barbara Barbé | 29/08/2012 | |
| *Révisé par* | | |
| Philippe Gillet | 05/09/2012 | |
| *Approuvé par* | | |
| Veerle Lejon | 17/Sep/2012 | |

| | |
|---|---|
| nidiag — Better Diagnosis of Neglected Infectious Diseases | **SOP titre :** Test de glucose (glycémie) HumanSens glucomètre |
| | **Projet/Étude :** Evaluation de tests rapides en association avec des prédicteurs cliniques et de laboratoire pour le diagnostic de maladies tropicales négligées chez les patients se présentant avec des troubles neurologiques dans les hôpitaux ruraux au Bandundu, République Démocratique du Congo. |

# 1. Domaine et application

*Le glucomètre HumaSens est utilisé pour la mesure de la glycémie sur sang total à l'aide des bandelettes de test – HumaSens Glucose. Le glucomètre est étalonné sur plasma pour faciliter la comparaison des résultats avec ceux obtenus par des méthodes biochimiques.*

# 2. Responsabilités

| Fonction | Activités |
|---|---|
| *Technicien de laboratoire* | ▪ *Prélève le sang capillaire*<br>▪ *Exécute le test de glucose*<br>▪ *Enregistre les résultats* |

# 3. Procédures

## 3.1 Précautions

- Tous les échantillons de sang sont potentiellement infectieux. Respectez les précautions universelles.
  METTEZ DES GANTS À USAGE UNIQUE PENDANT TOUTE LA PROCÉDURE **!**

## 3.2 Matériel et échantillons

### 3.2.1 Matériel fourni et conservation

- Glucomètre « HumaSens »
- Bandelettes « HumaSens Glucose »
  *Conservation* : à température ambiante (10 – 30°C). Utilisez les bandelettes de tests dans les 3 mois suivant l'ouverture du flacon.
  Remarques :
    - Evitez toute exposition au soleil ou à une chaleur directe.
    - Bien refermez le flacon après avoir enlevé une bandelette.
- Bandelette de code (glucose)
- Solution de contrôle normal « HumaSens Glucose » (4 ml)
  *Conservation* : à température ambiante (10 - 30°C).
  Utilisez la solution de contrôle dans les 3 moins suivant l'ouverture du flacon.
  Remarque : Evitez l'exposition au soleil direct, à la chaleur, à la réfrigération et à la congélation.

### 3.2.2 Matériel supplémentaire requis

- Gants, non stérile, à usage unique
- Lancettes stériles
- Coton hydrophile
- Tampons de coton noyé en éthanol 70%
- Conteneur pour aiguilles
- Conteneur « Bio-hazard »

### 3.2.3 Echantillon à examiner

- Sang total capillaire frais: 1 µl

## 3.3 Mode opératoire

### 3.3.1 Procédure de codage du glucomètre

- Insérez la bandelette de code dans l'orifice de test du glucomètre HumaSens.
- Le numéro de code de la bandelette est affiché sur l'écran du glucomètre.
- Contrôlez si le numéro de code affiché est le même que celui de la bandelette de code et du flacon des bandelettes de tests.
  Remarque : Consultez le mode d'emploi si les numéros ne correspondent pas.
- « ☺ » s'affiche quand le codage de glucomètre pour le lot respectif de bandelettes a réussi.

### 3.3.2 Test de contrôle

- Effectuez le test de contrôle « HumaSens Glucose » avec chaque nouveau lot de bandelettes et chaque semaine.
- Vérifiez la date de péremption de la solution de contrôle.
- Mélangez bien le flacon avant l'emploi.
- Eliminez les premières 3 gouttes et essuyez le compte-goutte.
- Mettez une goutte de solution de contrôle sur un morceau de Parafilm.
- Suivez la procédure de test, décrit sous 3.3.4.
- Vérifiez si le résultat est compris dans la plage de contrôle indiquée sur le flacon des bandelettes de test.

### 3.3.3 Prélèvement capillaire

- Mettez des gants à usage unique.
- Nettoyez le bout du doigt avec un tampon de coton, imbibé de l'éthanol.
- Laissez le sécher complètement à l'air.
- Piquez le bout du doigt avec une lancette stérile.
- Jetez lancette dans un conteneur pour aiguilles.
- Essuyez la première goutte de sang avec un tampon de coton sec et propre.
- Utilisez la deuxième goutte de sang pour faire le test de glucose.
- Mettez un tampon de coton sur la place de la piqûre.

### 3.3.4 Procédure de test

- Vérifiez la date de péremption des bandelettes de tests.
- Mettez des gants à usage unique.
- Sortez une bandelette de test du flacon.
- Rebouchez immédiatement le flacon.
- Insérez complètement l'extrémité de l'électrode de la bandelette de test (étiquette vers le haut) dans l'orifice pour bandelette du glucomètre.
  Remarque : NE réutilisez PAS les bandelettes de test.
- Le glucomètre s'allume automatiquement.
- Le numéro de code s'affiche.
- Vérifiez que le numéro de code et le symbole du mode de dosage sont le même que ceux indiqués sur le flacon de bandelettes de test.
  Remarque : Si les numéros ne correspondent pas, retirez la bandelette et reprogrammez le glucomètre de nouveau.
- Le glucomètre affiche un symbole en forme de goutte de sang.
- Mettez la goutte de sang au bout de doigt du patient en contact avec la zone d'introduction de la bandelette.
- Le glucomètre bipe pour indiquer que l'échantillon a réussi.
- Un compte à rebours de 10 secondes s'affiche sur l'écran.
- La glycémie est affichée sur l'écran.

Remarque : Les messages « Lo » et « Hi » sont affichés quand le résultat du glucose est < 20 mg/dl et > 600 mg/dl respectivement.

- Retirez la bandelette de test du glucomètre.
- Le glucomètre s'arrête automatiquement.
- Jetez la bandelette dans un conteneur « Bio-hazard ».

## 4. Enregistrements et archives

| **Appendices & formulaires à compléter** | |
|---|---|
| **Numéro** | **Titre** |
| 1 | CRF "laboratoire" |

## 5. Références

- Mode d'emploi de HumaSens glucomètre (17540001F, révision 02, 2009-10)
- Mode d'emploi de HumaSens Glucose bandelettes de test (INF 1754201F, 09-2009-01)
- Mode d'emploi de HumaSens Glucose Control Solution (Normal) (INF 1754502GB, 09-2009-01)

## 6. Histoire de document

| **Révision** | |
|---|---|
| | Version initiale |

| **Nom et fonction** | **Date** | **Signature** |
|---|---|---|
| *Auteur* | | |
| Barbara Barbé | 13/09/2012 | |
| *Révisé par* | | |
| Philippe Gillet | 13/09/2012 | |
| *Approuvé par* | | |
| Veerle Lejon | 17/Sep/2012 | |

| | |
|---|---|
| | **SOP titre** : Procédure pour le remplissage du Case Report Form |
| | **Projet/Étude :** Evaluation de tests rapides en association avec des prédicteurs cliniques et de laboratoire pour le diagnostic de maladies tropicales négligées chez les patients se présentant avec des troubles neurologiques dans les hôpitaux ruraux au Bandundu, République Démocratique du Congo. |

# 1. Champ d' application

Le Case Report Form (CRF) contient toutes les données collectées pendant l'étude. Il y a un CRF par malade. Le but de ce POS est de décrire comment les CRFs doivent être complétés et corrigés. Il s'applique à toutes les études NIDIAG et à tout le personnel impliqué dans le remplissage et la vérification des CRFs.

# 2. Responsabilités

| Fonction | Activités |
|---|---|
| Investigateur du site (ou personnel délégué, par ex infirmiers) | - Remplir le CRF selon le POS<br>- Assurer la lisibilité, la complétude et l'exactitude des données et leur consistance avec les documents source (dossier médical du patient, résultats de laboratoire ou de test clinique)<br>- Conserver le CRF dans un endroit approprié<br>- Corriger les erreurs signalées par le Moniteur de l'étude ou par le responsable Qualité du site |
| Responsable Qualité du site et Moniteur externe | - Vérifier que les données sont exactes, complètes et à-jour.<br>- Signaler des erreurs et des inconsistances à l'investigateur<br>- Assurer que des erreurs détectées pendant le monitoring sont corrigées par l'investigateur |

# 3. Procédures

## 3.1 Confidentialité

- Le CRF est un document anonyme. Le nom du malade, son numéro de téléphone et son adresse ne peuvent pas apparaitre sur le CRF. Les seuls identifiants présents sur le CRF sont le numéro du patient, ses initiales, date de naissance et son âge ;
- Il est possible de conserver des copies de documents source (par exemple dossier médical ou résultats de laboratoire) seulement si ces copies sont anonymes
- Ne conservez pas le formulaire de consentement éclairé dans le CRF

## 3.2 Compléter le CRF

- Utilisez un bic pour remplir le CRF.
- Toutes les entrées doivent être faites en MAJUSCULES.
- Ecrivez seulement dans les champs pré-imprimés.
- Vérifiez que les données repris dans le CRF correspondent à celles dans le document source.
- Ne laissez aucune question sans réponse. Si la réponse n'est pas connue, alors marquez NK pour Not Known (Non Connu). Si une procédure n'a pas été exécutée, marquez ND (Not Done). Si une question n'est pas applicable, marquez "NA" (Not Applicable).
- Le CRF doit être tenu à jour pendant que le patient est dans l'étude
- Signez et datez le CRF chaque fois qu'une visite est terminée. Ainsi vous marquez votre responsabilité par rapport à l'exactitude des données.

### 3.3 Correction de CRF

- Chaque correction dans le CRF doit être date et paraphé (ou signé) .
- L'entrée originale doit rester lisible. N'utilisez pas de marqueur correcteur ou de liquide correcteur pour effacer l'entrée originale que vous voulez modifier. Tracez une seule ligne à travers l'erreur et écrivez la réponse correcte à côté de l'entrée originale.
- Les instructions pour le remplissage des CRF seront ajoutées aux premières pages du CRF. Selon les évolutions dans l'étude, des amendements à ces instructions pourront être rajoutés.

### 3.4 Conservation et accès

- Les CRFs doivent être gardés dans un endroit sûr et sous clef. L'accès est strictement réservé au personnel de l'étude
- Les CRF doivent être gardés en respect de la législation locale et pour au moins deux ans après le début de l'étude

## 4. Définitions et abréviations

- CRF= Case Report Form: un document pré-imprimé qui est destiné à collecter toutes les données requises par le protocole.
- Investigateur (ou Investigateur de site): Une personne en charge de la mise en œuvre d'une étude clinique dans un site d'étude
- Responsable qualité d'un site : Une personne qui veille à ce que les systèmes qualité soient respectés à chaque étape de NIDIAG sur une base journalière. Il/elle supervise toute activité de recherche et assure que les études sont menées en accord avec le protocole, les SOPs, GCP/GCLP et les regulations nationales.
- Moniteur: Un moniteur est une personne qui vérifie que l'étude est menée, documenté et rapportée en accord avec le protocole d'étude, Good Clinical Practice (GCP), et Standard Operating Procedures (SOPs).
- Documents source: ceci sont les documents originaux. Ils consistent de données en lien avec l'étude comme le dossier médical,, des fichiers administratifs, des résultats de laboratoire, des rapports de consultation, des registres de pharmacie etc ..
- POS (SOP) : Procédure Opérationnelle Standardisé

## 5. Archives

| Annexes & Formulaires à remplir | |
|---|---|
| **Nombre** | **Titre** |
| 1 | Case Report Form |

## 6. Document History

*Indicate previous versions of the SOP and the changes made*

| Révision | |
|---|---|
| SOP-WP6-DATA-01-V1-01Feb2012 | Vérsion initiale |
| SOP-WP6-DATA-01-V1.1-18Jun2012 | Traduction de V01 de l'Anglais en Français |

| Name and function | Date | Signature |
|---|---|---|
| *Auteur (traduction SOP-WP6-DATA-01-V1-01Feb2012)* | | |
| *Pascal Lutumba* | 18 June 2012 | [signature] |
| *Reviewed by* | | |
| | | |
| *Approved by* | | |
| *Veerle Lejon* | 18 June 2012 | [signature] |

**SOP titre :** Procédure pour la gestion des données en RDC

- Circulation des CRFs
- Saisie des données (Data entry)
- Nettoyage des données (Data cleaning)
- Faire des requêtes (Querying & DCF management)
- Fermer la base des données (Database Lock)
- Backup des données

**Projet/Étude :** Evaluation de tests rapides en association avec des prédicteurs cliniques et de laboratoire pour le diagnostic de maladies tropicales négligées chez les patients se présentant avec des troubles neurologiques dans les hôpitaux ruraux au Bandundu, République Démocratique du Congo.

## 1. Champ d' application

Le but de cette SOP est de décrire comment les données doivent être rentrées dans la base de données. En plus les méthodes sont décrits qui doivent assurer que la saisie c'est fait avec des CRFs complètes et que les données seront corrigées systématiques, jusqu'à elles sont exactes et à-jour.
Le POS s'applique à l'étude NIDIAG NEURO et au personnel impliqué dans :

- la circulation et la vérification des CRFs ;
- la saisie des données dans la base des données de l'étude :

## 2. Responsabilités

| Fonction | Activités |
|---|---|
| Investigateur du site (ou personnel délégué, par ex infirmiers) | - Remplir le CRF selon le POS WP6 DATA 01<br>- Assurer la lisibilité, la complétude et l'exactitude des données et leur consistance avec les documents source (dossier médical du patient, résultats de laboratoire ou de test clinique)<br>- Conserver le CRF dans un endroit approprié<br>- Corriger les erreurs signalées par le Moniteur ou le personnel de la gestion des données de l'étude<br>- Encore contrôler la complétude et l'exactitude des données avant d'envoyer le CRF pour la saisie des données. |
| Personnel du Laboratoire INRB et MSF | - Vérifier que les données sont exactes, complètes et à-jour.<br>- Assurer que des erreurs détectées sont corrigées. |
| Assistante administrative de l'INRB | - Point focale pour la distribution du CRF entre le site et l'INRB. |
| Personnel de gestion des données de l'INRB | - Tracer les CRF pages pour obtenir un CRF complète<br>- Responsable pour la saisie des données<br>- Gérer la circulation des requêtes au but de compléter et corriger les données.<br>- Assurer le backup des données<br>- Exécuter ces activités en fonction du gestionnaire des données central (située à l'ITM). |
| Gestionnaire des données central (situé à ITM) | - Coordination et point focale de la gestion des données pour les études NEURO, FIEVRE et DIGESTIVE. |

# 3. Procédures

## 3.1 Circulation des CRFs

- **Le site (Mosango)**
  - Envoie les CRF pages originales concernant les tests laboratoires au INRB (Kinshasa), en particulière au Administration. Pour le CRF v 1.1 ce sont les pages 31-40 ; pour le CRF v 1.2 ce sont les pages 33-42.
  - Dès que les CRF pages copies avec les résultats laboratoire (INRB ; MSF) seront retournés, arrangez-vous pour l'envoie des CRF complètes vers le Administration de l'INRB.
  - Vérifiez bien que avant l'envoie tous les CRFs sont complétés, exactes et signés.
  - Gardez des copies des pages envoyer. Arrangez-les par patient.
- **INRB (Kinshasa) :**

  **Administration**
  - Supervisez que les pages 31-38 (CRF v 1.1) ou 33-40 (CRF v 1.2) seront distribués vers le Laboratoire de l'INRB.
  - Supervisez que les pages 39-40 (CRF v 1.1) ou 41-42 (CRF v 1.2) seront distribués vers le Laboratoire de MSF.
  - Assurez que les pages retournant des LAB INRB et MSF sont complétés avec des résultats et que ces pages originales sont classées dans un endroit sûr à l'Administration. .Assurer aussi que des copies des pages des LAB INRB et MSF sont redistribués vers le site (Mosango).
  - Assurez que les CRF complètes venant du site seront distribué au personnel de gestion des données de l'INRB.

  **Laboratoire** :
  - Complétez bien les pages 31-38 (CRF v 1.1) ou 33-40 (CRF v 1.2). Suivez bien les instructions générales et retournez les pages complètes et exactes à l'Administration de l'INRB.
- **MSF (Kinshasa) :**

  **Laboratoire** :
  - Complétez bien les pages 39-40 (CRF v 1.1) ou 41-42 (CRF v 1.2). Suivez bien les instructions générales et retournez les pages complètes et exactes à l'Administration de l'INRB.

## 3.2 Saisie dans la base de données (Data Entry)

- Vérifiez que chaque CRF est complété, exacte et signé, avant de commencer la première saisie.
- Suivez les instructions pour l'entrée des données (voie l'annexe 1).
- Assurez-vous de compléter toujours la première saisie avant de commencer avec la deuxième saisie.
- Après la première saisie, inscrivez vos initiales et date au première page du CRF. Après la deuxième saisie, inscrivez les initiales de l'autre encodeur et date aussi au première page du CRF.
- Complétez le fichier du 'CRF Tracking'.Ce fichier récolte l'info sur la tracé, la complétude et l'état du gestion des données.de chaque CRF (voie l'annexe 2).
- Assurez-vous pour chaque CRF entrée dans la base des données, que toutes les données sont complétées et que les requêtes sont résolues.
- Attendez pour appuyer sur 'finalize' du chaque deuxième saisie, jusqu'au moment de la fermeture de la base des données (voie aussi 3.5)

## 3.3 Nettoyer la base des données (Data Cleaning)

- Suivez les instructions pour l'entrée des données aussi pour nettoyer la base des données (voie l'annexe 1).
- Cliquez 'Check and save' dans la base des données pour voir des erreurs possibles.
- Utilisez 'Show Compare' dans la base des données pour résoudre les différences entre la première et deuxième saisie.
- Assurez-vous que pour chaque CRF les corrections seront faites avant d'appuyer sur 'finalize'. Après de 'finaliser' les données en deuxième saisie, le CRF ne peut plus être corrigé.

## 3.4 Circulation des requêtes (Querying and DCF management)

- En cas que des requêtes doivent être envoyé vers le site ou les laboratoires, utilisez les formulaires pour laisser clarifier les données (→ DCF= Data Clarification Forms; voie l'annexe 3 et 4).
- Assignez pour chaque formulaire envoyé un nouveau numéro.
- Complétez le fichier du 'DCF Tracking'. Ce fichier récolte l'info sur la tracé et sur l'état du requête(s) de chaque DCF (voie l'annexe 5). Si nécessaire, correcte la base des données sur base de la réponse reçu du site ou laboratoires.
- Gardez les formulaires, si éléctronic dans un fichier 'DCF', si papier dans un classeur 'DCF'. Organisez-vous pour faires 3 sub fichiers ou sections : 1. 'DCFs posés'. ; 2. DCFs 'répondus ; 3 DCFs résolus.

## 3.5 Fermeture de la base des données (Database Lock)

- Quand la base des données est complète et tous les requêtes sont résolus il faut encore appuyez sur 'finalize' du chaque deuxième saisie. Après de 'finaliser' la deuxième saisie, la base des données ne peut plus être changée et les données sont prêtes pour être transférer au l'Investigateur ou statisticien.
- Le moment de la fermeture de la base des données est décidé avec, au minimum, le Gestionnaire des données central et l'Investigateur.

## 3.6 Backup des données et mesures de sécurités

- Faites régulièrement un backup des données, par préférence chaque jour.
- Faîtes et gardez le backup au l'ordinateur (dans fichier 'Backup) et aux 2 flash discs, chaque fois.
- Complétez chaque fois le formulaire du backup (voie l'annexe 6).
- Gardez l'ordinateur et les flash discs dans un endroit sûr. Seulement les personnel autorisé peuvent avoir accès à l''ordinateur et aux flash discs.
- L'ordinateur et les flash discs ne peuvent que être utilisés pour des raisons professionnelles et pour le projet NIDIAG.
- Le responsable pour le backup doit envoyer le backup chaque vendredi au responsable gestionnaire des données central de l'ITM.

## 4. Définitions et abréviations

- CRF= Case Report Form: un document pré-imprimé qui est destiné à collecter toutes les données requises par le protocole.
- Investigateur (ou Investigateur de site): Une personne en charge de la mise en œuvre d'une étude clinique dans un site d'étude

- Moniteur: Un moniteur est une personne qui vérifie que l'étude est menée, documenté et rapportée en accord avec le protocole d'étude, Good Clinical Practice (GCP), et Standard Operating Procedures (SOPs).
- Documents source: ceci sont les documents originaux. Ils consistent de données en lien avec l'étude comme le dossier médical, des fichiers administratifs, des résultats de laboratoire, des rapports de consultation, des registres de pharmacie etc ...
- POS (SOP) : Procédure Opérationnelle Standardisé

## 5. Archives

| **Annexes & Formulaires à remplir** | |
|---|---|
| **Nombre** | **Titre** |
| 1 | Instructions pour l'entrée des données |
| 2 | Fichier pour tracer les CRFs (CRF Tracking File) |
| 3 | 'Formulaire pour faire des requêtes (Data Clarification Form - multiple requêtes par formulaire) |
| 4 | 'Formulaire pour faire des requêtes (Data Clarification Form - une requête par formulaire) |
| 5 | Fichier pour tracer les requêtes envoyées (DCF Tracking File) |
| 6 | Formulaire pour enregistrer les backups des données |

## 6. Document History

*Indicate previous versions of the SOP and the changes made*

| **Révision** | |
|---|---|
| *SOP-WP6-DATA-02-V1-04DEC2013* | *Version Initiale* |

| **Name and function** | **Date** | **Signature** |
|---|---|---|
| *Auteur* | | |
| *Harry van Loen* | 04 Dec 3013 | |
| *Reviewed by* | | |
| *Emmanuel Bottieau*<br><br>*Barbara Barbé* | 12 Dec 2013<br><br>13 Dec 2013 | |
| *Approved by* | | |
| Raffaella Ravinetto | 16 Dec 2013 | |

## Annexe 1 : Instructions entrée des données

**Lisez** ces Instructions avant chaque jour de travail pendant les premières semaines!
**Gardez** une copie de ces instructions près de vous pendant que vous entrez les données!

# INSTRUCTIONS POUR L'ENTREE DES DONNEES NIDIAG NEURO

Ecrites par H. van Loen

## Table des Matières


Table de Matière ……………………………………………………………………….....1

1. Remplir le LogIn – Ouvrir la base des données…..……………………………………2
2. Chercher un 'fichier de participant' dans la Data Entry module………………………3
3. Créer un nouveau 'fichier de participant'……………………………………………...3
4. Instructions Générales…………………………………………………………………6
5. Instructions Spécifiques ………………………...…………………………………..7
   - 5.1 Tous les formulaires…………………………………………………………….8
     - 5.1.1 Check and Save…………………………………………………………….8
     - 5.1.2 Dates………………………………………………………………………….8
   - 5.2 Page 1-3……………………………………………………………………………8
   - 5.3 Page 4 Inclusion/Exclusion..……………………………………………………8
   - 5.4 Page 12-13…………………………………………………………………………8
   - 5.5 Page 44-56…………………………………………………………………………9
   - 5.6 Page 57-58…………………………………………………………………………9
   - 5.7 Page 59-61…………………………………………………………………………9
6. Première saisie/Deuxième saisie des données…………………………………………9
7. Utilisez le TAB 'Finalize'……………………………………………………………10
8. Exit du Base des données…………………………………………………….......11
9. Backup du Base des données……………………………………………….........12

# 1. REMPLIR LE LOGIN – OUVRIR LA BASE DES DONNÉES

Entrez en naviguant dans: C:/NIDIAG/ETUDE NEURO/Database comme affiché ci-dessous:

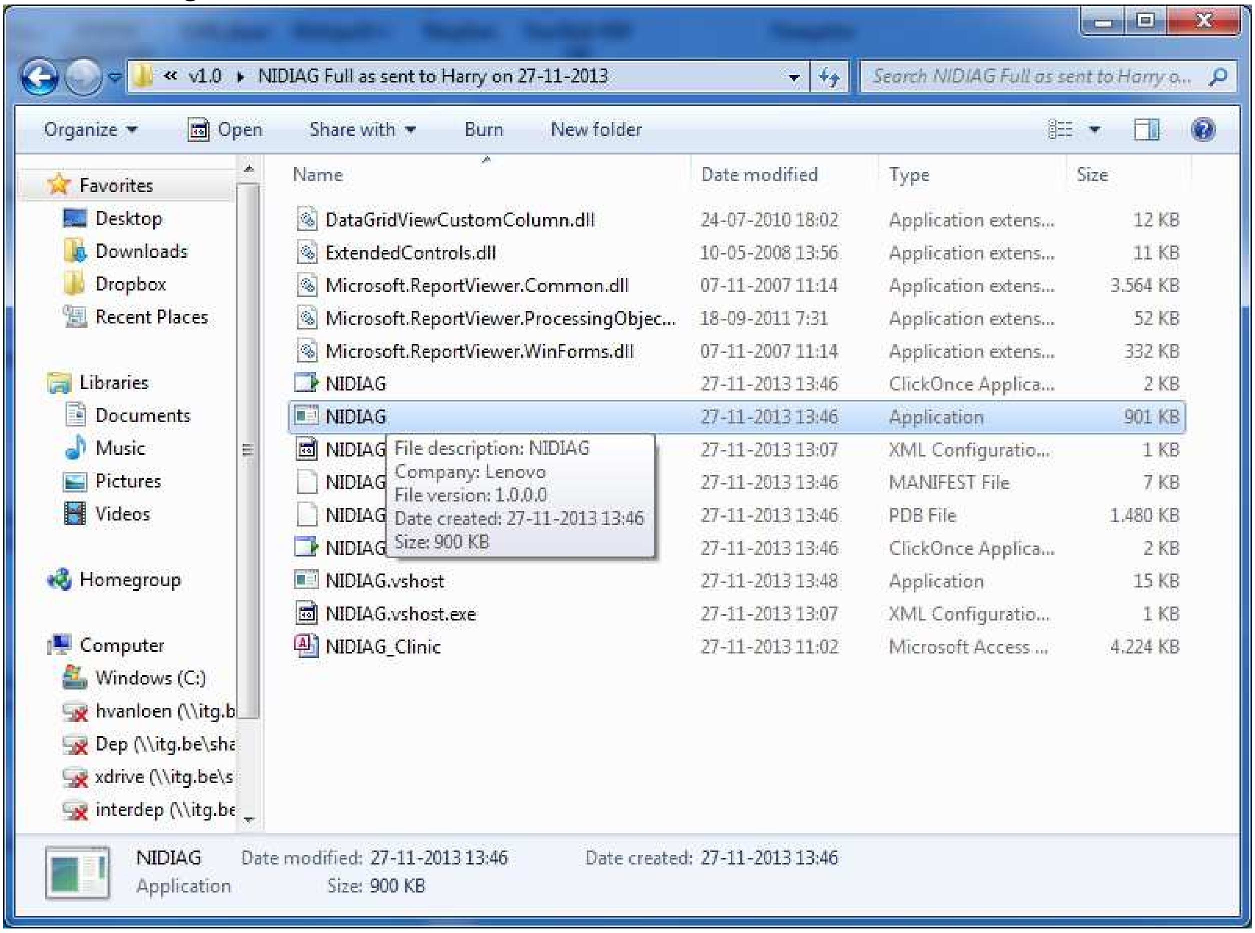


Vous verrez l'écran suivant où vous pouvez entrer votre nom d'utilisateur et votre mot de passe qui vous ont été procurés

**Si vous avez oublié votre mot de passe, veuillez contacter ITM, Belgique par courrier électronique vers hvanloen@itg.be**

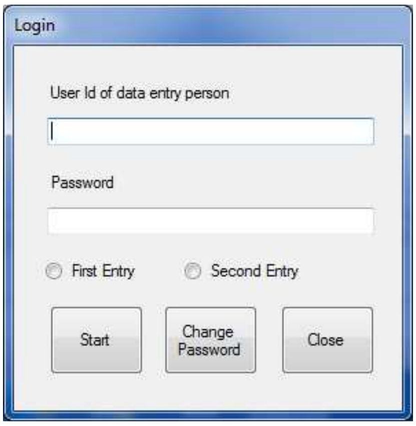


Note : Pour la première fois : Cliquez sur « Change Password » pour changer le mot de passe qui vous été procuré dans un mot de passe personnel. Gardez le mot de passe bien !

Cliquez 'First Entry' (ou Second Entry si nécessaire)

Cliquez 'Start'

Maintenant vous rentrez dans la BASE DE DONNÉES:

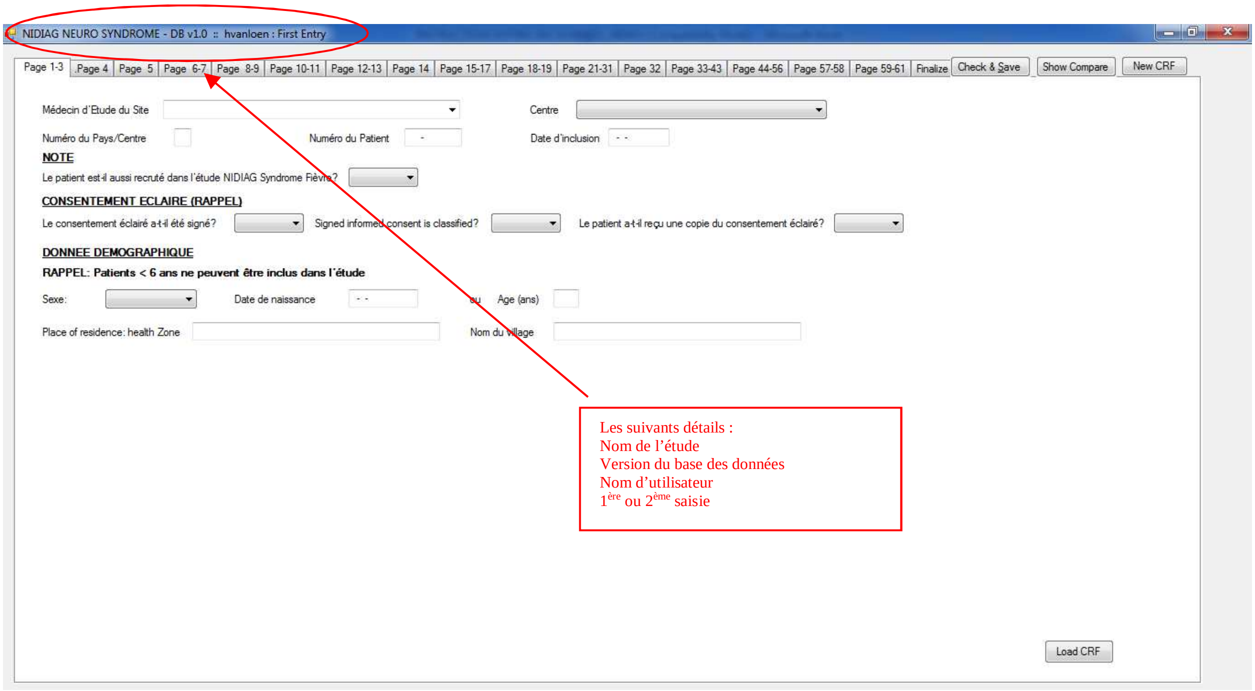


## 2. Chercher un 'fichier de participant' dans la BASE DE DONNÉES

Après être entré dans la **Base de données**, naviguez vers la première TAB (page 1-3), en particulier la question 'Numéro du patient'.

Ecrivez le numéro que vous cherchez et puis cliquez 'Load CRF'→ Le message **'Loading successful for the patient ID ….'**. et toutes les données du patient vont être présentées.

## 3. Créer un nouveau 'fichier de participant'

Si aucun participant n'a été trouvé en utilisant l'option de recherche (voir ci-dessus Chapitre 2), ceci veut dire que ce participant n'a pas été introduit dans la Base de données auparavant.
Vous recevrez le message: **'No such record found'.**

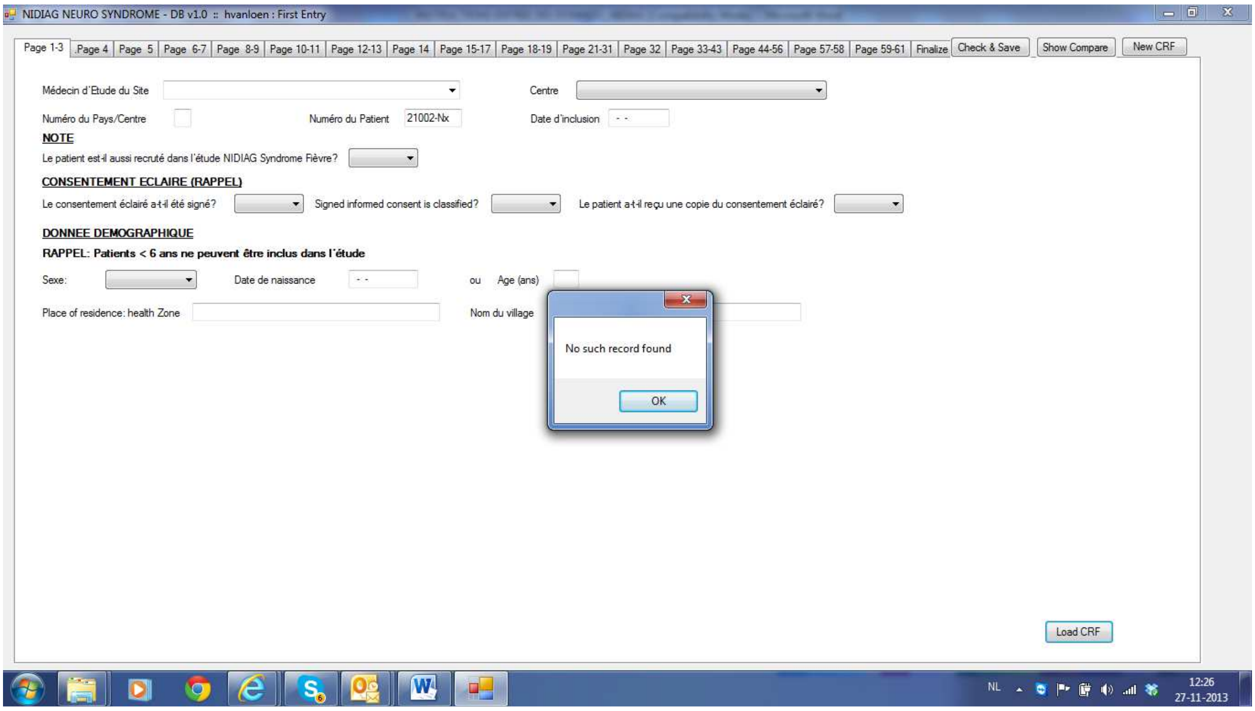


Veuillez contrôler doublement le ‘Participant number’ (numéro du participant) dans le CRF et le numéro du participant que vous avez complété dans l’option de recherche. Corrigé si nécessaire.

Si aucun participant n’a été trouvé, un nouveau participant doit être créé.
Naviguez vers la dernière TAB (New CRF), et cliquez ‘New CRF’
Vous recevez le message: **‘The current CRF is not FINALIZED yet. Are you sure to start NEW CRF’**

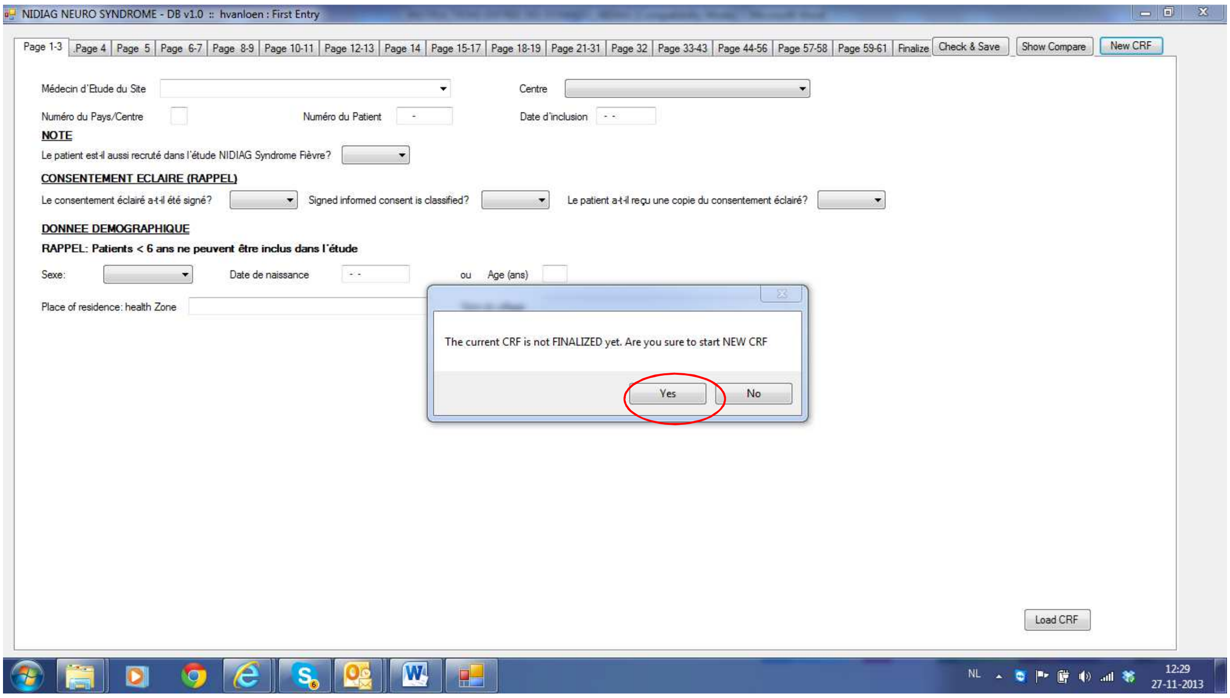


Cliquez ‘Yes’
Maintenant vous êtes entré dans un nouveau fichier du participant.

Notez que tous les formulaires sont encore 'blancs' = pas encore complétés.

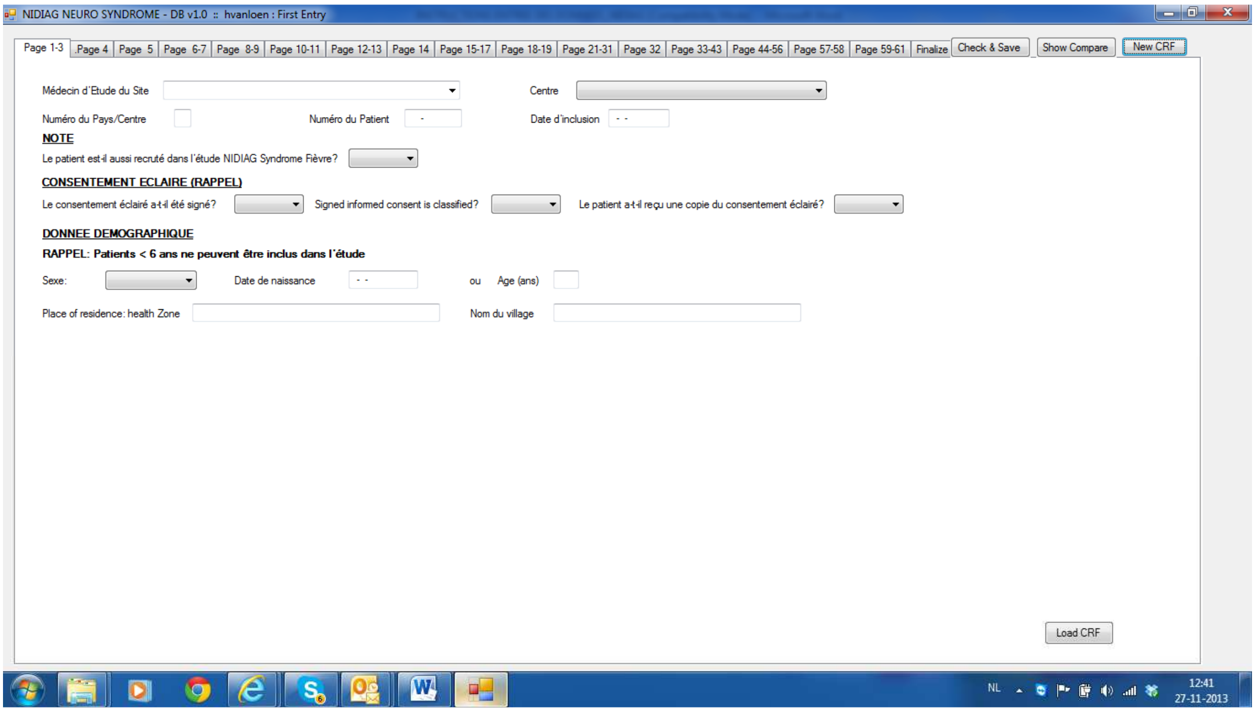


Premièrement vous devriez enregistrer le participant sur la première page (Page 1-3) avant de compléter les autres données du CRF!!

Maintenant ce nouveau participant a été créé et vous pouvez commencer à rentrer les données.

## 4. Instructions Générales

- Les documents en papier que vous utilisez sur lesquelles ces données sont écrites (par exemple par le docteur ou le laborant de l'étude) sont appelés le **'Case Report Form' (CRF)**.
- Les 'TABs' avec les pages, rangées de 'gauche à droite', reflètent le CRF (le base des données c'est en fait un miroir du CRF).
- Cliquez de l'un à l'autre TAB pour ouvrir les différents pages.
- Introduisez les données une par une du haut en bas si elles sont déjà complétées dans le CRF.
- Vérifiez ce qui est nécessaire dans le base des données et cherchez cette information dans le CRF, pas dans le sens inverse.
- A la fin de l'entrée d'une TAB dans la base de données veuillez signer (prénom ou initiales) et afficher la date au CRF document.
- Complétez tous les formulaires du même TAB (ou visite) avant de procéder avec le TAB (ou visite) suivante.
- Si une donnée est inconnue introduisez :
  - NC (format textes ; Non Connu)
  - 99-99-9999 (format dates)

- **!Veuillez introduire exactement ce qui est écrit dans le CRF dans la base des données, avec les suivants exceptions:**
  - **Utilisez le format Date : dd-MM-yyyyy** (et donc pas dd/mm/yyyy ; si nécessaire vous deviez changer le format 'short date', see also 'control panel' >> 'region and language settings' >> additional settings)
  - **Dates Inconnu dans le CRF :**
    - 99/mm/yyyy → tapez 01-MM-YYYY
    - 99/99/yyyy →tapez 01-07-YYYY
    - 99/99/9999 →tapez 99/99/9999
  - **Complétez toutes les données en LETTRES CAPITALES**
  - **Utilisez le format de nombre avec mettre de zéro en avant si necessaire :** e.g. 01 en place de 1 quand il y a deux digits ; 065 en place de 65 quand il y a 3 digits…

# 5. Instructions spécifiques

## 5.1 Tous les formulaires

### 5.1.1. Check and save

- Après de compléter les pages de chaque TAB, cliquez sur ‘Check and save’.
- Vous recevez le message: **‘Do you want to save this page?’**
- Continuez avec ‘Oui’ (ou cliquez ‘Non’ si vous voulez encore corriger)

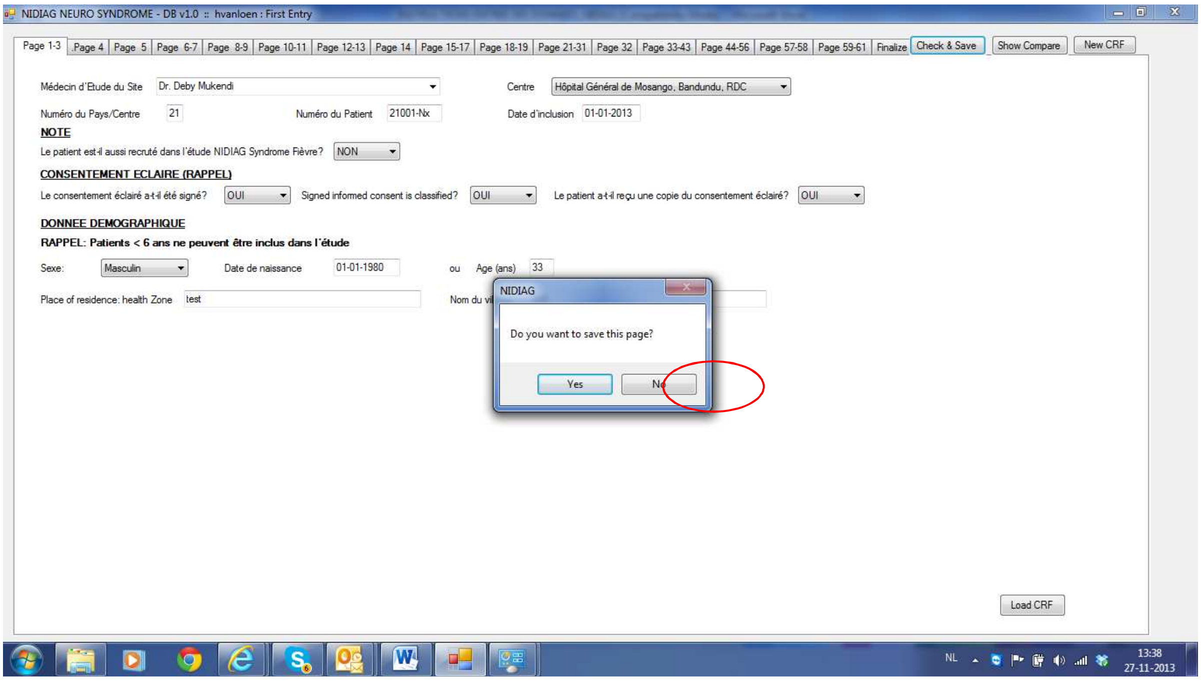


- Attention : des avertissements ou messages erreurs rouges indiquent des données manquantes ou incohérentes !

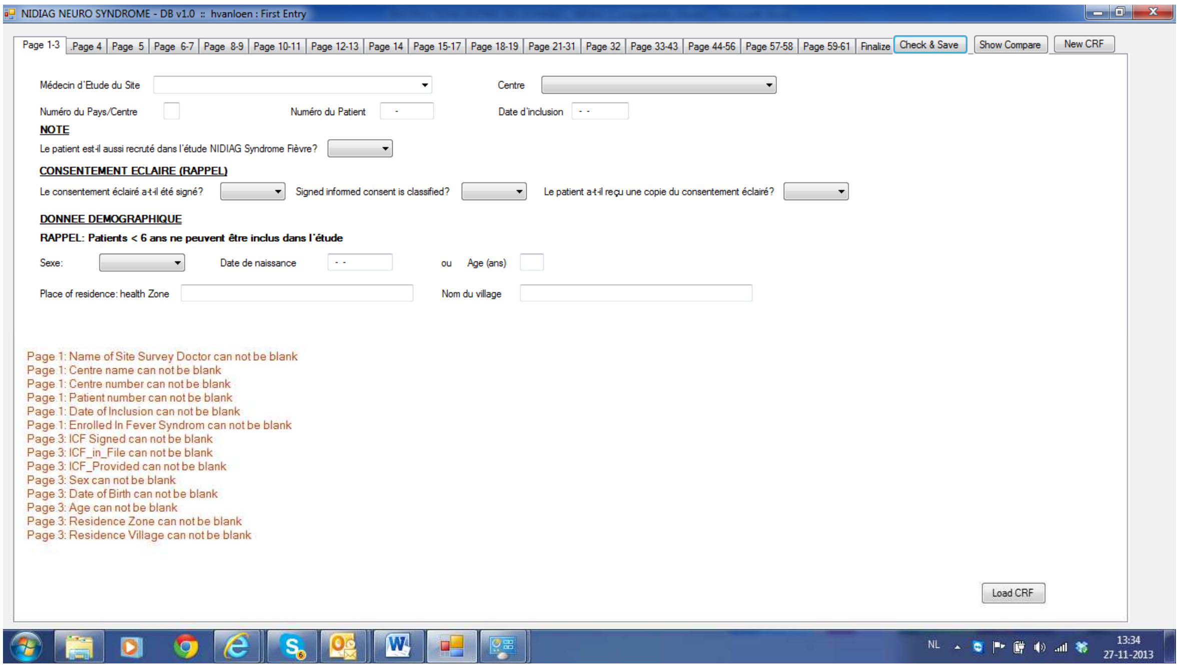

### 5.1.2. Dates (en general)

- Si un avertissement apparait du suivant type :
  …Date must be before today…
  …Future dates are not allowed…
  …Date must be between 01-SEP-2011 and 01-JUL-2015
  …Date must be between 01-SEP-2012 and 01-JUL-2015
  Contrôlez bien la date pour la question en particulier

## 5.2 Page 1-3

- Complétez ce formulaire en premier lieu quand vous créez un nouveau fichier de participant!!!
- Assurez-vous que ce participant n'est pas encore dans le CRF (évitez des duplicates de participants!)
- Vérifiez le Numéro du patient entre la base de données et le CRF.
- Si un avertissement apparait du suivant type :
  Page 3: Date of inclusion must be between 01-Sep-2012 and 01-Jul-2015
  Contrôlez bien le date pour la question 'Date de inclusion '

## 5.3 Page 4 : Inclusion/Exclusion

- Si un avertissement apparait du suivant type :
  Page 4: Al least one inclusion criteria must be met
  Page 4: At least one exclusion criteria met. Patient cannot be enrolled in the study.
  Contrôlez bien les questions Oui/Non des critères d'inclusions/exclusions

- Si un avertissement apparait du suivant type :
  Page 4: Inclusion criteria not met. Patient cannot be enrolled in the study. Please check!.
  Contrôlez bien la question 'SELECTION POUR L'ETUDE.

- Si un avertissement apparait pour le numéro de participant:
  Mismatch in Patient_Number from Page-1.
  Contrôlez bien avec le numéro de participant de la première TAB (page 1-3)

## 5.4 Page 12-13

Questions Poids, Taille, Fréquence respiratoire, Pouls, Tension artérielle, Glasgow coma scale : il faut **mettre un zéro au début du chiffre (quand nécessaire) : e.g.**
Poids : 65 → 065
Taille : 99 → 099
Fréquence respiratoire : 60 → 060
Pouls : 75 → 075
Tension artérielle : 90/50 → 090/050
Glasgow coma scale : 8/15 → 08/15

Question Température : il faut **mettre un zéro après le point décimal** ex. 38. → 38.0

## 5.5 Page 33-43

- Assuréz bien que les pages 33-40 (qui viennent du laboratoire du INRB) et page 41-42 (qui viennent du MSF) sont présentes et complétes.

Le tableau en haute compiles pour chaque culture une nouvelle ligne.
Les résultats et diamétre 'Antibiotiques ROSCO' , pour chaque culure, seront entrer dans le tableau en bas à gauche.
Les résultats GeneXpert (CRF page 42) seront entrer dans le tableau en bas à droite.

## 5.6 Page 44-56

Le tableau en haute compiles pour chaque visite une nouvelle ligne.
Les résultats du tableaux 'Diagnostic' du CRF, pour chaque visite, seront entrer dans le tableau en milieu.
Les résultats Fiche De Conclusion Fin D'etude (CRF page 54-56) seront entrer dans le tableau en bas.

## 5.7 Page 57-58

Le tableau en haute compiles pour chaque visite non-planifiée une nouvelle ligne.
Les résultats Nouvelles investigations principlaes (CRF page 58) seront entrer dans le tableau en bas.

## 5.8 Page 59-61

- Si un avertissement apparait du suivant type :
  DATE de FIN cannot be before DATE de DEBUT
  Contrôlez bien les dates de FIN et DÉBUT
- L'indication du fiche du médication doit être la même (ou cohérente) avec la description du symptôme du fiche du suivi de clinique.

## 6. Première saisie/ Deuxième saisie des données

Pour première saisie → sélectionnez 'First Entry' et 'puis 'Start'

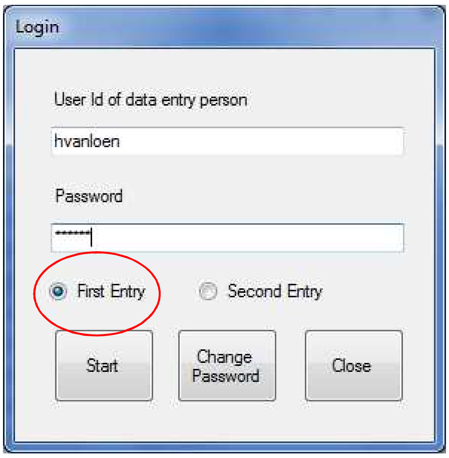

**Note : avant de commençer avec la deuxième saisie, vous devez terminer et completez** la première saisie

Pour deuxième saisie → sélectionnez 'Second Entry' et 'puis 'Start'

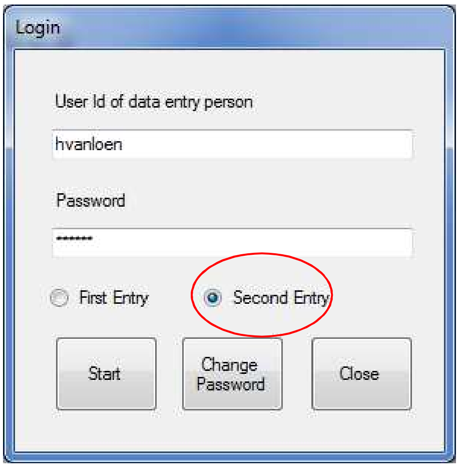


**La personne qui fait la deuxième saisie a la possibilité et la responsabilité pour faire les corrections des données. Il/elle doit toujours vérifié bien les données du base des données avec le CRF !**

Les différences entre première et deuxième saisie seront indiqué à chaque TAB du deuxième saisie de moment qu'on clique **'Check and save'**. Autre moyen pour voir différences entre première et deuxième saisie c'est de cliquer dans deuxième saisie sur **'Show compare'.** Ce bouton montre pour chaque TAB les résultats de la première saisie qui sont différents. C'est à la personne qui fait la deuxième saisie de choisir que le résultat de la première saisie ou deuxième saisie ou autre résultat doit être entré.

## *7. Utilisez le TAB 'Finalize'*

Après compléter toutes les données pour la première saisie il faut cliquez 'Finalize'. Après ça ce n'est plus possible de modifier les données pour la première saisie.
On ne peut pas commencer la deuxième saisie sans 'finaliser ' la première saisie.

La deuxième saisie donne toujours l'opportunité pour corriger ou pour entrer des données manquantes.
Après compléter toutes les données pour la deuxième saisie il faut aussi cliquez 'Finalize'. Mais après ça ce n'est plus possible de modifier les données pour ni première saisie, ni deuxième saisie. **C'est pour cette raison que le moment de cliquer 'Finalize' est prévu comme ultime étape, juste avant la fermeture de la base des données.**

## *8. Exit de la Base de données*

Pour fermer la base de données cliquez sur la croix rouge en haut à droite.

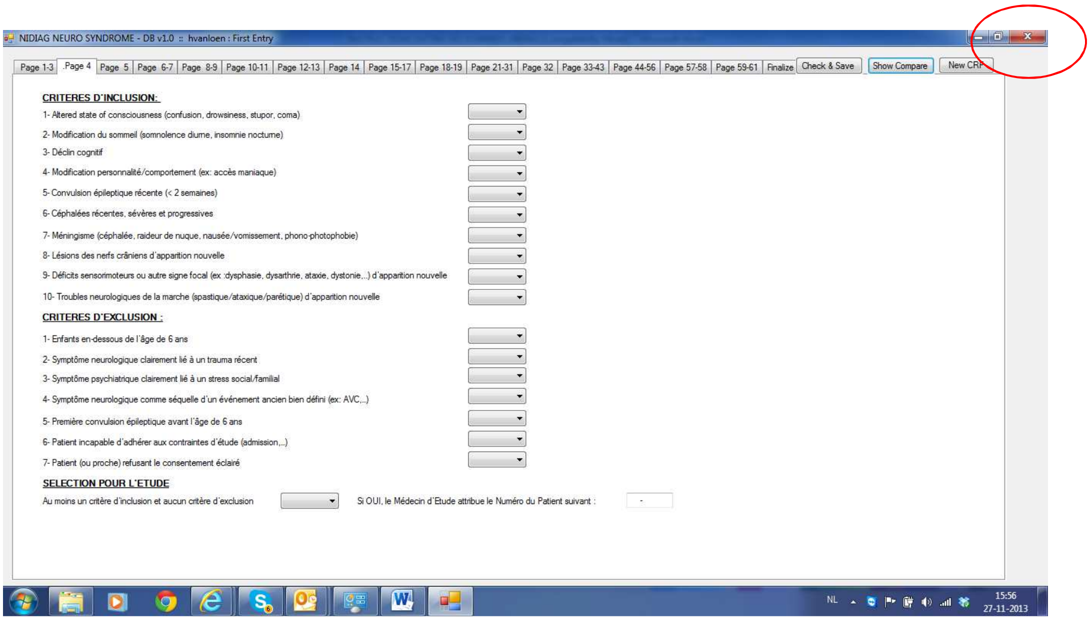


## *9. Backup du Base des données*

**Il faut un backup des données chaque fois qu'on termine ou à la fin du chaque jour.**
**Ce backup doit être sauvegardé dans un dossier particulier pour les backups de la base de données. Il faut le sauver sur l'ordinateur de l'étude et aussi sur un lecteur flash USB.**

**C'est important de sauver le backup comme suivant :**
**NIDIAG_NEURO_DB_v1.0_date (avec la date).**
**Gardez les backups avec les différentes dates.**

**Gardez l'ordinateur de l'étude et aussi le lecteur flash USB dans un endroit sûr.**

## Annexe 2 : CRF tracking

| CRF version:<br><br>1.1 ou 1.2 | CRF Complète: Oui/ Non | Date Reçu CRF:<br><br>_ _/ _ _/ _ _ _ _ | CRF page/data manquants: | Initiales | Date 1st Saisie fait:<br>_ _/ _ _/ _ _ _ _ | Initiales | Date 2nd Saisie fait :<br>_ _/ _ _/ _ _ _ _ | Initiales | Requêtes non résolues: Oui/ Non | Remarques |
|---|---|---|---|---|---|---|---|---|---|---|
| | | | | | | | | | | |
| | | | | | | | | | | |
| | | | | | | | | | | |
| | | | | | | | | | | |
| | | | | | | | | | | |
| | | | | | | | | | | |
| | | | | | | | | | | |
| | | | | | | | | | | |
| | | | | | | | | | | |
| | | | | | | | | | | |
| | | | | | | | | | | |
| | | | | | | | | | | |
| | | | | | | | | | | |
| | | | | | | | | | | |
| | | | | | | | | | | |
| | | | | | | | | | | |
| | | | | | | | | | | |
| | | | | | | | | | | |

\- NEURO STUDY-

# DATA CLARIFICATION FORM

DCF N° __ __ __ __

From DMC (Kinshasa ) ou __ __ __ __ __ __ __ __ __ __ __

To Site Mosango ou __ __ __ __ __ __ __ __ __ __ __ __ __

No du Patient __ __ __ __ __ __ - __ __ CRF version : _._ (1.1 ou 1.2)

Nom CRF section __ __ __ __ __ __ __ __ __ __ __ __ __ __ __ __ __ __ __ __ __ __ __ __ __ __ __ __ __ __ __ __ _

Visite __ __

Données à clarifier Date de la visite __ __ /__ __ __ __/__ __

| **Page numéro** | **Données à clarifier** | **Réponse** | **Résolu (Oui ou Non)** | **Ré-posé (Oui ou Non)** | **DCF N°** |
|---|---|---|---|---|---|
| | | | | | |
| | | | | | |
| | | | | | |
| | | | | | |

________________________________ ________________________________

Requête posé par (initiales et date) Répondu par (initiales et date)

____________________________________

Base des données mis à jour (initiales and date)

# NEURO STUDY- DATA CLARIFICATION FORM

**DCF N°** __ __ __ __

| No du Patient °: | Visite+Date Visite : | CRF section: | Données à clarifier: | CRF page: | CRF version: |
|---|---|---|---|---|---|
| | | | | | |

| **Requête (Query) :** |
|---|
| |

Posé par: __ __ __ __ __ __ __ __ __ __ __ __ __ __ (Prénom /Nom) Date __ __/__ __ __/__ __ __ __ (DD/MMM/YYYY)

| **Réponse:** |
|---|
| |

Réponse par: __ __ __ __ __ __ __ __ __ __ __ __ __ __ (Prénom/Name) Date __ __/__ __ __/__ __ __ __ (DD/MMM/YYYY)

**Note: État ne peut être rentré par les Moniteurs y Gestion des Données**

| **État Requête:** | Base des Données mis à jour : |
|---|---|
| O Résolu à Date __ __/__ __ __/__ __ __ __ (DD/MMM/YYYY)<br>O Ré-posé comme DCF N° __ __ __ __ | O Oui à Date __ __/__ __ __/__ __ __ __ (DD/MMM/YYYY) |

## Annexe 5 : DCF tracking

| DCF N°:<br><br>_ _ _ _ | Participant N°:<br><br>21_ _ _-_ _ | Date DCF<br>Posé par DMC:<br>_ _/ _ _/ _ _ _ _ | Date DCF<br>Répondu par site<br>_ _/ _ _/ _ _ _ _ | Date DCF<br>Résolu par DMC:<br>_ _/ _ _/ _ _ _ _ | DCF re-posé:<br><br>Oui / Non | Re-posé with DCF N°:<br><br>_ _ _ _ |
|---|---|---|---|---|---|---|
| | | | | | | |
| | | | | | | |
| | | | | | | |
| | | | | | | |
| | | | | | | |
| | | | | | | |
| | | | | | | |
| | | | | | | |
| | | | | | | |
| | | | | | | |
| | | | | | | |
| | | | | | | |
| | | | | | | |
| | | | | | | |
| | | | | | | |
| | | | | | | |
| | | | | | | |
| | | | | | | |

**Annexe 6 : Data back-up log**

# Evaluation of Rapid Diagnostic Tests in association with clinical and laboratory predictors for the diagnosis of Neglected Tropical Diseases in patients presenting with neurological disorders in rural hospitals of Bandundu, Democratic Republic of Congo

## *"NIDIAG neuro syndrome"*
## DATA BACKUP

**Procédure:**

**Pour des raisons de sécurité il faut faire régulièrement un backup des données , par préférence même chaque jour.**

Faîtes et gardez le backup au l'ordinateur et aux 2 flash discs chaque fois.

Gardez l'ordinateur et les flash discs dans un endroit sûr. Seulement les personnel autorisé peuvent avoir accès à l''ordinateur et aux flash discs.

L'ordinateur et les flash discs ne peuvent que être utilisés pour des raisons professionnelles et pour le projet NIDIAG.
Le responsable pour le backup doit envoyer le backup chaque vendredi au responsable manager de gestion des données à l'ITM.

Complétez le Log pour chaque backup qui est fait :
***p.e.: Le Samedi, Dimanche ou jours congés, marquez NA (Non applicable).***

**Mois………………………**

| **Date** | **Backup Faites?** | | **Si Non, pourquoi pas?** | **Staff Initiales** |
|---|---|---|---|---|
| | **Oui** | **Non** | | |
| **1** | | | | |
| **2** | | | | |
| **3** | | | | |
| **4** | | | | |
| **5** | | | | |
| **6** | | | | |
| **7** | | | | |
| **8** | | | | |
| **9** | | | | |
| **10** | | | | |
| **11** | | | | |
| **12** | | | | |
| **13** | | | | |
| **14** | | | | |
| **15** | | | | |
| **16** | | | | |

| | | | | |
|---|---|---|---|---|
| **13** | | | | |
| **14** | | | | |
| **15** | | | | |
| **16** | | | | |
| **17** | | | | |
| **18** | | | | |
| **19** | | | | |
| **20** | | | | |
| **21** | | | | |
| **22** | | | | |
| **23** | | | | |
| **24** | | | | |
| **25** | | | | |
| **26** | | | | |
| **27** | | | | |
| **28** | | | | |
| **29** | | | | |
| **30** | | | | |
| **31** | | | | |

| | |
|---|---|
| nidiag Better Diagnosis of Neglected Infectious Diseases | **SOP titre:** Procédure d'obtention du consentement éclairé |
| | **Projet/étude:** Cette SOP s'applique à toutes les études NIDIAG |

# 1. Champ d'application

Avant qu'un patient ne décide à participer dans une étude NIDIAG, il/elle devra être informé de l'objectif de l'étude, des examens cliniques, et des bénéfices et risqué lies à sa participation. Si le patient confirme sa volonté de participer à l'étude, alors il/elle signera le Formulaire de Consentement Eclairé (FCE) et il met la date. .
L'objectif de cette procédure opérationnelle standardisé (POS) est de décrire les procédures et les responsabilités afin d'obtenir le consentement éclairé des participants dans les études NIDIAG.

# 2. Responsabilités

| Fonction | Activités |
|---|---|
| Investigateur sur le site | - Donner au malade toute l'information nécessaire afin qu'il/elle puisse prendre une décision informée, et vérifier qu'il/elle a tout compris.<br>- Obtenir et documenter ce consentement libre donné par le patient (ou son représentant légal/parent) avant qu'il/elle soit soumis à quelconque procédure liée à l'étude, y inclus les procédures de dépistage qui ne font pas partie des soins cliniques de routine<br>- Conserver les FCE dans un endroit approprié<br>- Documenter les retraits de consentement pendant l'étude |
| Responsable Qualité sur le site et Moniteur Externe | - Vérifier que le FCE soit complet, daté et signé par l'investigateur et le patient (ou son représentant légal/parent)<br>- Vérifier que le retrait de consentement soit bien documenté |

# 3. Procédures

## 3.1 Chronologie

- Il faut obtenir le consentement éclairé de tous les patients (ou son représentant légal/parent) avant que tout test ou évaluation lié à l'étude ne soit faite. Ceci inclut les procédures de dépistage qui ne font pas partie des soins cliniques de routine, et inclut toute procédure qui ne fait pas partie des soins cliniques de routine.

## 3.2 Information et discussion

### 3.2.1 Information

- Donnez au malade (ou son représentant légal/parent) une information appropriée et complète sur l'étude, selon l'information contenue dans le Formulaire de Consentement Eclairé (FCE) et en accord avec les Principes de la déclaration de Helsinki et des Guidelines de l'IOMS et ICH et toute régulation nationale d'application.

- Plus spécifiquement, expliquez les points suivants :
  - Les objectifs de l'étude;
  - Les procédures de l'étude, et plus spécifiquement toutes les procédures diagnostiques additionnelles qui seront faites selon le protocole de l'étude;
  - La durée de participation, le nombre de visites et la durée du suivi selon le protocole de l'étude;
  - Les risques et inconvénients, y compris les risques liés aux procédures diagnostiques qui ne font pas partie des procédures de routine (ex. prise de sang additionnelles, etc...);
  - Les bénéfices attendus pour le patient, s'il y en a, et les bénéfices attendus pour la communauté ;
  - Les procédures alternatives qui sont disponibles et leur bénéfices et risques;
  - Le remboursement des frais de transport comme décrit dans le protocole;

- Que le participant peut à tout moment refuser de participer ou se retirer de l'étude sans sanction ou perte de bénéfices;
- Mécanismes d'indemnisation en cas de tort subi par la participation dans l'étude;
- Le fait que toutes les données de l'étude seront gardées confidentielles et que l'identité du sujet sera confidentiel aussi quand le résultat de l'étude sera publié ;
- Le fait que le sujet (ou son représentant légal/parent) sera informé si une nouvelle donnée apparaitrait qui pourrait influencer leur participation dans l'étude;
- La personne à contacter pour obtenir plus d'information sur l'étude;

## 3.3 Langage

- Donner l'information dans un langage simple, qui sera compris par le malade (ou son représentant légal/parent). Eviter le jargon médical.
- Utiliser la langue maternelle du patient tant que possible.
- Donnez suffisamment de temps au patient (ou son représentant légal/parent) pour lire le FCE et poser des questions sur l'étude. Répondez à toutes ses questions
- Assurez-vous que le patient ne se sent pas obligé de participer dans l'étude.

### 3.3.1 Analphabétisme

- Si le patient (ou son représentant légal/parent) ne sait ni lire ni écrire, un témoin impartial doit être présent pendant toute la discussion sur le consentement éclairé.
- Décidez au cas par cas sur le besoin d'un témoin impartial, même pour des patients qui ont reçu une certaine éducation formelle.

## 3.4 Patients non compétents sur le plan légal

### 3.4.1 Situations chroniques

- Pour des patients qui sont légalement non compétents (handicaps physiques ou mentaux, mineurs) on obtiendra le consentement de leur représentant légal. L'investigateur devra s'assurer que cette personne est légalement autorisée à le faire.
- Dès que possible, par exemple chez les adolescents, on demandera l'assentiment du patient, pour s'assurer que celui–ci n'est pas forcé à participer.

### 3.4.2 Situations d'urgence

- Au cas où le patient est admis dans un état critique qui l'empêche de décider, la procédure de consentement éclairé ne doit pas retarder les soins.
- Dans ces cas, le consentement d'un représentant légal sera obtenu initialement. L'investigateur devra s'assurer que cette personne est légalement autorisée à le faire.
- Une fois l'urgence passée, et le patient rétabli, le consentement du patient devra être obtenu. Si le patient refuse à ce moment de participer, toute information déjà collectée devra être écartée de l'étude.

## 3.5 Signature et documentation

- Après explication et discussion, le patient (ou son représentant légal/parent) confirmera son consentement par écrit, en apposant la date et signant le FCE.
- L'investigateur qui mène l'interview, ou la personne qualifiée à laquelle l'investigateur aurait délégué la procédure de consentement éclairé doit également signer et dater le FCE.
- Si le patient est illettré, une empreinte digitale peut remplacer la signature, pourvu qu'un témoin impartial soit présent pendant l'entièreté de la discussion de consentement éclairé. Ce témoin impartial doit aussi signer et dater le FCE. En signant le FCE, le témoin atteste que l'information contenue dans le FCE et toute autre information écrite a été correctement expliqué et apparemment comprise par le malade et que le consentement éclairé a été donné de son plein gré.
- Le FCE doit être signé en deux copies originales. Une copie sera donnée au patient (ou son représentant légal/parent) et l'autre sera gardé dans le Investigator Master File ou dans un autre endroit sûr, accessible uniquement à l'équipe de l'étude.

### 3.6 Après la signature

- Il relève de la responsabilité de l'investigateur de s'assurer que toute nouvelle information qui pourrait influencer la volonté du patient à participer dans l'étude est donnée au patient en temps utile.
- Si pendant l'étude le patient (et/ou parent/gardien/ représentant légal) décide de retirer son consentement, il/ elle est libre à le faire. Indiquez la date/temps et la raison de ce retrait dans le CRF et autre document d'étude comme le recruitment log.

## 4. Archivage et accès au FCE

- Les FCE signés doivent être gardés dans le Investigator Master File (IMF) et gardés sous clef dans un endroit sûr. L'accès doit être strictement restreint au personnel médical de l'étude.
- Ne gardez pas le FCE dans le Case Report Form (CRF).
- Les personnes suivantes sont autorisées à consulter le FCE:
  - L'investigateur principal du site
  - Les investigateurs du site, les cliniciens et les infirmiers
  - Le responsable Qualité du site
  - Les moniteurs externes
  - Les représentants des Comités Ethiques, si ceci est requis par la règlementation du pays
  - Les représentants des autorités régulatrices
- Le FCE doit être conservé avec les autres documents d'études pendant au moins 10 ans après la fin de l'étude

## 5. Définitions et abbréviations

### 5.1 Définitions

- Consentement Eclairé: Le consentement éclairé est le processus par lequel le participant à une étude confirme de son plein gré qu'il ou elle est d'accord de participer dans une étude. Seulement des participants qui ont complètement compris tous les aspects relatifs à leur participation à un étude peuvent faire un jugement valable en la matière et donner leur consentement pour participer dans une étude
- Confidentialité: Le respect de la confidentialité des sujets de l'étude y compris leur identité et toute information médicale personnelle.
- Investigateur: Toute personne médicale impliqué dans le déroulement de l''étude, et responsable pour l'étude et pour les droits, santé et bien-être des sujets dans l'étude
- Témoin impartial: une personne indépendante de l'étude qui ne peut pas être influence par des personnes impliquées dans l'étude et qui observe le processus de consentement éclairé lorsque le patient ne sait pas lire. Ce témoin lira le FCE et toute autre information écrite donnée au patient.

### 5.2 Abbréviations

- CRF: Case Report Form
- FCE: Formulaire de Consentement Eclairé
- IMF: Investigators Master File
- POS: Procédure opératoire standardisée

## 6. Données et archives

| Annexes & Formulaires | |
|---|---|
| **Nombre** | **Titre** |
| 1 | Formulaire de Consentement Eclairé |
| | |

## 7. Historique du Document

*Indiquer les versions préalables du POS et révisions*

| Révision | |
|---|---|
| SOP-WP6-DOC-01-V01-01Feb2012 | Vérsion initiale |
| SOP-WP6-DOC-01-V01.1-18Jun2012 | Traduction de V01 de l'Anglais en Français |

| Nom et function | Date | Signature |
|---|---|---|
| *Auteur (traduction SOP-WP6-DOC-01-V01-01Feb2012)* | | |
| *Pascal Lutumba* | 18 June 2012 | |
| *Revisé par* | | |
| | | |
| *Approuvé par* | | |
| *Veerle Lejon* | 18 June 2012 | |

| | |
|---|---|
| | **SOP titre:** Système de numérotation à utiliser dans les études NIDIAG WP2 |
| | **Projet/étude:** Cette SOP s'applique à toutes les etudes NIDIAG |

## 1. But et application

Dans le but de protéger la confidentialité du patient, toutes les données de la recherche doivent demeurer anonymes, donc toutes les informations des personnes (c.à.d. noms, téléphone, numéro, adresse, etc.) doivent être ôtées du CRF, des étiquettes et des autres documents de l'étude et doivent être remplacées par un numéro de l'étude unique pour le patient. Ce numéro unique est attribué à partir de l'inclusion du participant dans l'étude et permet de retracer ses informations médicales et les spécimens biologiques. Ce SOP décrit comment un numéro d'étude du patient unique est crée et comment les échantillons des patients sont numérotés et étiquetés.

## 2. Responsabilités

| Fonction | Activités |
|---|---|
| Investigateur du site | - Attribuer un numéro d'étude unique à chaque participant inclut en respectant ce SOP<br>- Établir une liste d'identification détaillant les correspondances entre le numéro de l'étude et le nom du patient<br>- Se rassurer que la liste d'identification est gardée dans un lieu sûre et que son accès est limité à l'équipe des investigateurs sur le site<br>- Se rassurer que le numéro du patient est le même sur touts les documents et les échantillons de l'étude |
| Investigateur du site<br>Technicien de laboratoire | - Attribuer un numéro de spécimen de l'étude unique à chaque spécimen récolté durant l'étude en respectant ce SOP<br>- Établir un journal détaillant les numéros des spécimens de l'étude, des numéros de l'étude du patient, le type d'échantillon biologique et la date et le moment de la collecte |
| Manager (gestionnaire) de qualité | - Vérifier que ce SOP est utilisé<br>- Vérifier que la liste d'identification des patients et que le journal des spécimens de l'étude sont correctes, à jour, et gardés d'une manière sûre<br>- Vérifier que le numéro d'étude du patient est le même pour touts les documents et touts les échantillons de l'étude<br>- Vérifier que les échantillons de l'étude sont identifiés et étiquetés en respectant le protocole de l'étude, la liste d'identification du patient et ce SOP |

## 3. Liste d'identification des patients

- Reprendre touts les patients potentiellement éligibles à inclure dans "la Liste d'Identification des patients" (cfr. Figure 1, annex 1). Il y a une "Liste d'Identification des patients" par syndrome et par centre.
- Indiquez les informations suivantes :
    1) Nom, âge, sexe du patient
    2) Si le patient a été inclus dans l'étude ou pas
    3) La raison de non-inclusion si cela est applicable (ex: refus de participer, moins de 5 ans… etc.)
    4) Le numéro d'étude du patient (ce ne sont que les patients inclus dans l'étude qui auront un numéro, voir ici-bas)
    5) La date de l'inclusion dans l'étude

| Patient identification list | | | | | | | |
|---|---|---|---|---|---|---|---|
| Country & study centre (name, number): Nepal, Dankuta Hospital (61) | | | | | | | |
| Row nr | Patient's name (Last, First) | Age *(years)* | Sex (M/F) | Included (yes /no) | Reason for non inclusion | Patient Study n° | Inclusion date *(dd/mmm/yyyy)* |
| 1 | Dupont Francis | 41 | M | yes | na | 61001-Fx | 14/07/2012 |
| 2 | Piccard Marie | 4 | F | no | The patient is below 5 | na | na |
| 3 | | | | | | | |
| 4 | | | | | | | |
| 5 | | | | | | | |

**Figure 1**: Exemple d'une liste d'identification des patients pour le syndrome de la fièvre

# 4. Numéro d'étude du patient

- Donner un numéro d'étude du patient unique à chaque patient inclut dans l'une des études NIDIAG.
- Le numéro d'étude du patient consiste en 4 champs :
  1) Le numéro du pays (1 digit, voir le tableau 1)
  2) Le numéro du centre (1 digit, voir le tableau 1)
  3) Le numéro d'ordre du patient (3 digits)
  4) Le code NIDIAG du syndrome (2 lettres voir le tableau 2)

Ex: pour le premier patient inclus dans l'étude Clinique du Syndrome neurologique à Mosango, le numéro du patient est 21001-Nx.

Le numéro d'étude du patient est attribué après inclusion dans l'étude, c.à.d. après control des critères d'inclusion et d'exclusion et après que le patient ait signé le consentement éclairé.

## 4.1 Champ 1 et 2: numéro du pays et numéro du centre

Le premier digit du numéro d'étude du patient indique le pays ou le patient a été inclus. Le second digit du numéro d'étude du patient indique le site de l'étude ou le patient a été inclus.

**Tableau 1:** Liste de numérotation des pays et des centres (2 premiers digit du numéro d'étude du patient)

| Numéro du pays | Numéro du centre | pays | Site de l'étude |
|---|---|---|---|
| 1 | 1 | Cambodge | Sihanouk Hospital Center of HOPE |
| 2 | 1 | RD Congo | Hôpital rural de Mosango |
| 2 | 2 | RD Congo | Hôpital rural de Yasa – Bonga |
| 3 | 1 | Indonésie | Tulehu Hospital |
| 3 | 2 | Indonésie | Waai Health Center |
| 4 | 8 | Côte d'Ivoire | Hôpital Méthodiste de Dabou |
| 5 | 1 | Mali | INRSP Laboratoire de Réference de Parasitologie, Bacteriologie et Virologie |
| 5 | 2 | Mali | Niono Health Center |
| 6 | 1 | Népal | Dhankuta Hospital |
| 6 | 2 | Népal | Koshi Zonal Hospital |
| 6 | 3 | Népal | BPKIHS |
| 7 | 1 | Soudan | Tabarak Allah Hospital |

## 4.2 Champs 3: numéro d'ordre du patient

Dans chaque centre, touts les patients inclus dans l'étude (ne tenant pas compte du syndrome étudié) reçoivent un numéro d'ordre constitué de 3 digits.

Le premier patient inclus reçoit le numéro 001, le second 002 etc.

## 4.3 Champ 4: Syndrome

Le dernier champ du numéro du patient consiste en des lettres qui indiquent le syndrome pour lequel le patient a été inclus.

**Tableau 2:** Liste des syndromes et leurs lettres

| Lettre | Syndrome |
|---|---|
| Nx | Neurologique |
| Fx | Fébrile |
| Dx | Digestif |
| FN or NF | Fébrile et neurologique |
| FD or DF | Fébrile et digestif |

## 4.4 Exemples

1) Le premier patient inclus à l'Hôpital rural de Mosango au Congo reçoit le numéro 21001. Il est inclus dans l'étude clinique des syndromes neurologiques, son numéro d'étude du patient complet devient : 21001-Nx.
2) Le second patient inclut a l'Hôpital rural de Mosango au Congo reçoit le numéro 21002. Il est inclus dans l'étude Clinique des syndromes fébriles, son numéro d'étude du patient complet est : 21002-Fx.
3) Le troisième patient inclus à l'Hôpital rural de Mosango au Congo reçoit le numéro 21003. Il est inclus dans l'étude Clinique des syndromes neurologiques puis fébrile, il reçoit alors le numéro d'étude du patient : 21003-FN. Si le patient avait été inclus premièrement pour le syndrome fébrile puis pour le syndrome neurologique, il aurait reçu le numéro: 21003-NF.

# 5. Numéro de spécimen de l'étude

Touts les échantillons récoltés d'un patient durant la période de l'étude devraient être identifiés, en tout temps, par un numéro unique de spécimen de l'étude. Ceci s'applique aux échantillons utilisés pour les tests index, pour les tests de références et pour le stockage a long terme.

Le numéro de spécimen de l'étude consiste en:

1) Le numéro d'étude du patient
2) L'abréviation du type de spécimen (2 lettres, voir tableau 3 ici-bas, et annex 5)
3) Le numéro du spécimen sur le numéro total des spécimens collectés (important lorsqu'il y a plus d'un tube collecté).

**Tableau 3:** Liste des types d'échantillons et leurs abréviations

| type de Spécimen | Abréviation du type de spécimen |
|---|---|
| Sang pour hemoculture/ Blood for **HE**moculture | HE |
| Sang (tube sec) /**B**lood (**D**ry tube) | BD |
| Sang sur EDTA /**B**lood on **E**DTA | BE |
| Sang sur héparine /**B**lood on **H**eparin | BH |
| Plasma sur EDTA /**P**lasma on **E**DTA | PE |
| Plasma sur héparine /**P**lasma on **H**eparin | PH |
| Sérum /**SE**rum | SE |
| Urine/**UR**ine | UR |
| Liquide céphalo rachidien /**C**erebo**S**pinal fluid | CS |
| Culture de liquide céphalo rachidien /**C**erebrospinal fluid **C**ulture | CC |
| Selles /**ST**ool | ST |
| Aspiration du suc ganglionnaire /**L**ymph **N**ode aspirate | LN |
| Aspiration de moelle osseuse /**B**one **M**arrow aspirate | BM |
| Crachat /**S**putu**M** | SM |
| Organismes (bactéries) isolées du sang /**O**rganisms (bacteria) isolated from **B**lood | OB |
| Organismes (bactéries) isolées du liquide céphalo rachidien /**O**rganisms (bacteria) isolated from **C**erebrospinal fluid | OC |

## 5.1 Exemples

1) le premier patient inclus à l'Hôpital rural de Mosango au Congo reçoit le numéro 21001. Il est inclus dans le syndrome neurologique, son numéro d'étude du patient complet est : 21001-Nx.

- Du sang est collecté, en respect du protocole d'étude :
    - Pour l'hémoculture, et est numérotée 21001-Nx-HE1
    - Dans un tube sec, et est numérotée 21001-Nx-BD1. Si les 2 tubes de sérum sont préparés à partir du sang du tube, alors ils seront numérotés 21001-Nx-SE1 et 21001-Nx-SE2.
    - Sur héparine et est numéroté 21001-Nx-BH1. Si les 2 tubes de plasma sont préparés a partir du sang du tube, alors ils seront numérotés 21001-Nx-PH1 et 21001-Nx-PH2.
- L'urine est également collectée et le numéro du spécimen sera 21001-Nx-UR1
- Finalement, 4 échantillons de LCR seront collectés. Les 4 tubes seront numérotés comme suit : 21001-Nx-CS1, 21001-Nx-CS2, 21001-Nx-CS3, et 21001-Nx-CS4.

2) le numéro de spécimen 61014-Fx-PH1 indique que ce plasma sur héparine (premier tube collecté) vient du 14ieme patient inclus au Népal au Dhankuta Hospital, qui a participé pour le syndrome fébrile.

## 5.2 Journal des spécimens de l'étude

Touts les spécimens récoltés d'un patient de l'étude durant la période de l'étude devront être enregistrés dans le "journal des spécimens de l'étude" (voir figure 2, annex2). Il y a un Journal des Spécimen de l'étude par centre.

Les informations suivantes devront être indiquées :

1) Numéro d'étude du patient
2) Type de l'échantillon
3) Date de collection
4) Temps de collection
5) Étiquette du spécimen de l'étude (applicable seulement en utilisant des feuilles Excel)
6) La personne qui a récolté l'échantillon
7) Date de l'envoi
8) Organisation à laquelle l'échantillon a été envoyé
9) Personne à laquelle l'échantillon est envoyé
10) Date de réception de l'échantillon
11) Commentaires (si applicable)

**Study specimen log**

Country & study centre (name, number): RD Congo, Mosango (21)

| Row nr | Patient study n° | Sample type (__ __) | Date collection (dd/mmm/yyyy) | Time collection (hh:mm) | Study specimen label | Collected by | Date of shipment (dd/mm/yyyy) | Sent to Organization | Sent to person | Date of receipt | Comments |
|---|---|---|---|---|---|---|---|---|---|---|---|
| 1 | 21001-Nx | HE1 | 12/Mar/2012 | 10:24 | 21001-Nx-HE1-12/Mar/2012 | nurse x | 16/03/2012 | INRB | Lunguya | 17/03/2012 | |
| 2 | 21001-Nx | BD1 | 12/Mar/2012 | 10:24 | 21001-Nx-BD1-12/Mar/2012 | nurse x | na | na | na | na | |
| 3 | 21001-Nx | BH1 | 12/Mar/2012 | 10:25 | 21001-Nx-BH1-12/Mar/2012 | nurse x | na | na | na | na | |
| 4 | 21001-Nx | UR1 | 12/Mar/2012 | 10:45 | 21001-Nx-UR1-12/Mar/2012 | nurse x | na | na | na | na | |
| 5 | 21001-Nx | PH1 | 12/Mar/2012 | 11:00 | 21001-Nx-PH1-12/Mar/2012 | tech y | na | na | na | na | |
| 6 | 21001-Nx | PH2 | 12/Mar/2012 | 11:00 | 21001-Nx-PH2-12/Mar/2012 | tech y | 16/03/2012 | ITM | Jan Jacobs | 17/03/2012 | |
| 7 | 21001-Nx | CS1 | 12/Mar/2012 | 12:05 | 21001-Nx-CS1-12/Mar/2012 | Dr X | na | na | na | na | |
| 8 | 21001-Nx | CS2 | 12/Mar/2012 | 12:05 | 21001-Nx-CS2-12/Mar/2012 | Dr X | na | na | na | na | |
| 9 | 21001-Nx | CS3 | 12/Mar/2012 | 12:05 | 21001-Nx-CS3-12/Mar/2012 | Dr X | 16/03/2012 | ITM | Jan Jacobs | | lost |
| 10 | 21001-Nx | CS4 | 12/Mar/2012 | 12:05 | 21001-Nx-CS4-12/Mar/2012 | Dr X | 16/03/2012 | ITM | Jan Jacobs | 17/03/2012 | |
| 11 | 21001-Nx | SE1 | 12/Mar/2012 | 15:56 | 21001-Nx-SE1-12/Mar/2012 | tech y | na | na | na | na | |
| 12 | 21001-Nx | SE2 | 12/Mar/2012 | 15:56 | 21001-Nx-SE2-12/Mar/2012 | tech y | 16/03/2012 | ITM | Jan Jacobs | 17/03/2012 | tube damaged |
| 13 | | | | | | | | | | | |
| 14 | | | | | | | | | | | |
| [illegible] | | | | | | | | | | | |

**Figure 2:** un exemple du journal des spécimens de l'étude. Cet exemple correspond à celui donné au dessus sous le point 5.1 le sérum et le plasma ont été réalisés à partir du sang collecté sur un tube sec ou hépariné comme indiqué dans les commentaires. Pour l'envoi, la date de l'envoi, l'organisation et la personne qui reçoivent et le reçu de confirmation sont indiqués. Les autres échantillons ont été analysés sur le site, sur quoi, "non applicable (na) est rempli. Des listes de colisages de cryotubes et de cultures sont en annexes 3 et 4.

# 6. Étiquetage des spécimens de l'étude

L'étiquetage des spécimens de l'étude consiste en 2 champs :

1) Numéro d'identification des spécimens (tel que : 21001-Nx-CS1 et 61014-Fx-PH1)
2) Format de la date de collection : JJ-MM-AAAA par exemple : 13-FEB-2012

MM est l'abréviation en anglais des mois, listés dans le tableau 4.

**Tableau 4:** Liste des abréviations à utiliser pour le mois

| Mois | Abréviation |
|---|---|
| **Jan**vier/**Jan**uary | JAN |
| Février/**Feb**ruary | FEB |
| **Mar**s/**Mar**ch | MAR |
| Avril/**Apr**il | APR |
| Mai/**May** | MAY |
| Juin/**Jun**e | JUN |
| Juillet/**Jul**y | JUL |
| Aout/**Aug**ust | AUG |
| **Sep**tembre/**Sep**tember | SEP |
| **Oct**obre/**Oct**ober | OCT |
| **Nov**embre/**Nov**ember | NOV |
| **Déc**embre/**Dec**ember | DEC |

Les sites de l'étude sont responsables d'imprimer les étiquettes des spécimens. L'écriture doit être claire, en encre noir permanent en lettres majuscules (n'utilisez pas les imprimantes à jet d'encre parce que l'encre se dissoudra au contact de l'eau).

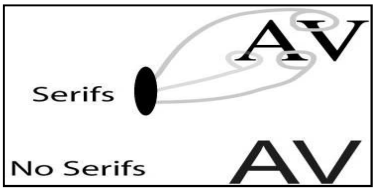


La taille du type de lettres sur l'étiquette doit être au moins de 9-points.

Le type de lettre sur l'étiquette est de préférence un type "sans serif" (voir la figure no serifs) tel qu'Arial

Alignez le texte à gauche

## 6.1 Exemples d'étiquettes

1) Une étiquette pour un échantillon de sang sur EDTA n°1 collecté du patient 115 au Tabarak Allah (Soudan) pendant l'étude sur le syndrome fébrile. Date de collection 23 Aout 2012:

| |
|---|
| 71115-Fx-BE1<br>23-AUG-2012 |

2) Une étiquette pour l'échantillon d'urine n°1 collecté du patient 89 à Mosango (RD Congo) pendant l'étude sur le syndrome neurologique. Date de collection 2 Juin 2013:

| |
|---|
| 21089-Nx-UR1<br>02-JUN-2013 |

## 6.2 Position correcte de l'étiquette

- <u>Tubes large (plus long que l'étiquette):</u>

1) Tenez le tube de l'échantillon horizontalement avec le bouchon dans la main gauche.
2) Attachez l'étiquette du patient de manière à être lue de la gauche à droite en commençant par le bas du bouchon (directement sur l'étiquette du fabricant).
3) Étiqueter aussi haut que possible sur le tube (pour permettre une plus grande largeur non couverte, facilitant le placement des tubes dans les portoirs etc.)

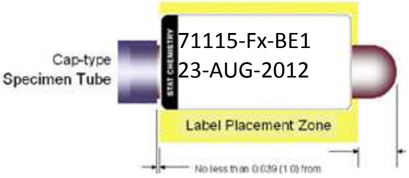


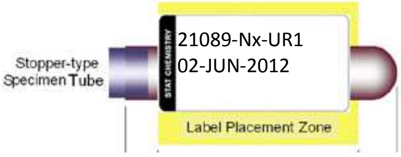


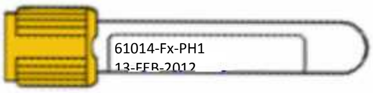

- <u>Petites tubes (plus petits que les étiquettes, par exemple, les cryotubes):</u>
  1) tenez le tube verticalement.
  2) Attachez l'étiquette en étant sure que le texte ne chevauche pas.
  3) Si vous utilisez des étiquettes en papier sur des tubes qui seront mis au frais ou congelés: couvrez complètement l'étiquette avec ruban adhésif transparent (le ruban adhésif devra totalement dépasser l'étiquette) pour éviter que l'étiquette ne tombe du tube.

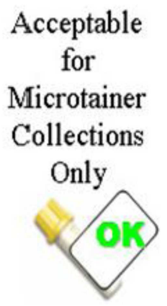


## 7. Rapports and Archives

| **Appendices & Formulaires à remplir** | |
|---|---|
| **Numéro** | **Titre** |
| Annex 1 | Liste d'identification des patients |
| Annex 2 | Journal des spécimens de l'étude |
| Annex 3 | Liste de colisage cryotubes |
| Annex 4 | Liste de colisage cultures |
| Annex 5 | Abréviations des spécimens |

## 8. Histoire du Document

| **Révisions** | |
|---|---|
| SOP-WP6-DOC-02-V01-25Jun2012 | *Version Initiale* |
| SOP-WP6-DOC-02-V01.1-09Jul2012 | *Traduction de l'anglais en français* |
| SOP-WP6-DOC-02-V02.1-18Sep2012 | *Annexes 3-5 ajoutés* |

| **Nom et fonction** | **Date** | **Signature** |
|---|---|---|
| *Auteur* | | |
| *Philippe Gillet* | 16/09/2012 | |
| *Approuvé par* | | |
| Veerle Lejon | 18/Sep/2012 | |

**Annexe 1: Liste d'identification des patients**

| Liste d'identification des patients |
|---|
| Pays et centre d'étude (nom, numéro): |

| N° de colonne | Nom du patient (nom, prénom) | Age *(années)* | Sexe (M/F) | Inclus (yes /no) | Raison de non inclusion | n° d'étude du patient | date d'inclusion *(jj/mmm/AAAA)* |
|---|---|---|---|---|---|---|---|
| 1 | | | | | | | |
| 2 | | | | | | | |
| 3 | | | | | | | |
| 4 | | | | | | | |
| 5 | | | | | | | |
| 6 | | | | | | | |
| 7 | | | | | | | |
| 8 | | | | | | | |
| 9 | | | | | | | |
| 10 | | | | | | | |
| 11 | | | | | | | |
| 12 | | | | | | | |
| 13 | | | | | | | |
| 14 | | | | | | | |
| 15 | | | | | | | |
| 16 | | | | | | | |

**Annexe 2 : Journal des spécimens de l'étude**

## Journal des spécimens de l'étude

Pays et centre d'étude (nom, numéro):

| Num. Col | N° d'etude du patient | d'échantillon (_ _ _) | Date de collection (jj/mmm/AAAA) | [illegible] collection (hh:mm) | étiquette du spécimen d'étude | Collecté par | Date de l'envoi (jj/mm/AAAA) | Envoyé à l'Organisation | Envoyé à la personne | Date de réception | Commentaires |
|---|---|---|---|---|---|---|---|---|---|---|---|
| 1 | | | | | | | | | | | |
| 2 | | | | | | | | | | | |
| 3 | | | | | | | | | | | |
| 4 | | | | | | | | | | | |
| 5 | | | | | | | | | | | |
| 6 | | | | | | | | | | | |
| 7 | | | | | | | | | | | |
| 8 | | | | | | | | | | | |
| 9 | | | | | | | | | | | |
| 10 | | | | | | | | | | | |
| 11 | | | | | | | | | | | |
| 12 | | | | | | | | | | | |
| 13 | | | | | | | | | | | |
| 14 | | | | | | | | | | | |
| 15 | | | | | | | | | | | |
| 16 | | | | | | | | | | | |

**SOP-WP6-DOC-02-V02.2-18Sep2012-annex3**

# LISTE DE COLISAGE - CRYOTUBES

**________, _____ /_____ /20_____**

Place, date de l'envoi

**Envoi à ________________________________, des :**

**pour :**

☒ **Cryotubes avec sang total, plasma EDTA, plasma héparine, sérum, urine, LCR**

☒ **Stockage à – 70°C**

| N° | N° de patient (ex. 21001-Nx) | PH 1 | PH 2 | BE 2 | BE 3 | PE 1 | PE 2 | SE 1 | SE 2 | UR 2 | UR 3 | CS 2 | CS 3 | Date de prélèvement | Heure de prélèvement | Commentaires |
|---|---|---|---|---|---|---|---|---|---|---|---|---|---|---|---|---|
| 1 | | | | | | | | | | | | | | / /20 | : | |
| 2 | | | | | | | | | | | | | | / /20 | : | |
| 3 | | | | | | | | | | | | | | / /20 | : | |
| 4 | | | | | | | | | | | | | | / /20 | : | |
| 5 | | | | | | | | | | | | | | / /20 | : | |
| 6 | | | | | | | | | | | | | | / /20 | : | |
| 7 | | | | | | | | | | | | | | / /20 | : | |
| 8 | | | | | | | | | | | | | | / /20 | : | |
| 9 | | | | | | | | | | | | | | / /20 | : | |
| 10 | | | | | | | | | | | | | | / /20 | : | |
| 11 | | | | | | | | | | | | | | / /20 | : | |
| 12 | | | | | | | | | | | | | | / /20 | : | |
| 13 | | | | | | | | | | | | | | / /20 | : | |
| 14 | | | | | | | | | | | | | | / /20 | : | |
| 15 | | | | | | | | | | | | | | / /20 | : | |
| 16 | | | | | | | | | | | | | | / /20 | : | |
| 17 | | | | | | | | | | | | | | / /20 | : | |
| 18 | | | | | | | | | | | | | | / /20 | : | |
| 19 | | | | | | | | | | | | | | / /20 | : | |
| 20 | | | | | | | | | | | | | | / /20 | : | |

Type d'échantillon : PH = plasma sur héparine, BE = sang sur EDTA, PE = plasma sur EDTA, , SE = sérum, UR = urine, CS = liquide céphalorachidien.

**Envoyé par**
**Nom :**

**Date :**
**Signature :**

**Reçu par**
**Nom :**

**Date :**
**Signature :**

## SOP-WP6-DOC-02-V02.1-18Sep2012-Annex4
# LISTE DE COLISAGE - CULTURES

**____________, _____ /_____ /20_____**

Place, date de l'envoi

**Envoi à ______________________________________________, des :**

- ☐ **Hémocultures (HE 1 & 2)**
- ☐ **LCR : Cultures BactAlert pédiatriques (CC 1)**
- ☐ **LCR : Cultures trans-isolates (CC 2)**
- ☐ **LCR : Cultures MGIT (CC 3)**

**pour :**

- ☐ **Examen bactériologique à ___________________________** (Endroit).
  **Date distribution à service bactériologie: _____ /_____ /20_____**
- ☐ **Envoi vers _______________________________________** (Endroit).
  **Date remisé: _____ /_____ /20_____**

| N° | N° de patient (ex. 21001-Nx) | HE 1 | HE 2 | CC 1 | CC 2 | CC 3 | Date de prélèvement | Heure de prélèvement | Commentaires |
|---|---|---|---|---|---|---|---|---|---|
| 1 | | | | | | | / /20 | : | |
| 2 | | | | | | | / /20 | : | |
| 3 | | | | | | | / /20 | : | |
| 4 | | | | | | | / /20 | : | |
| 5 | | | | | | | / /20 | : | |
| 6 | | | | | | | / /20 | : | |
| 7 | | | | | | | / /20 | : | |
| 8 | | | | | | | / /20 | : | |
| 9 | | | | | | | / /20 | : | |
| 10 | | | | | | | / /20 | : | |
| 11 | | | | | | | / /20 | : | |
| 12 | | | | | | | / /20 | : | |
| 13 | | | | | | | / /20 | : | |
| 14 | | | | | | | / /20 | : | |
| 15 | | | | | | | / /20 | : | |

Type d'échantillon : HE = sang pour hémoculture, CC = culture de liquide céphalorachidien.

| Envoyé par | Reçu par |
|---|---|
| **Nom :** | **Nom :** |
| **Date :** | **Date :** |
| **Signature :** | **Signature :** |

**Annexe 5 : Abréviations des spécimens**

SOP-WP6-DOC-02-V02.1-18 Sep2012-annex5

| Abréviation | Type de spécimen |
|---|---|
| **HE** | Sang pour hémoculture: **HE 1 – HE 2** |
| **BH** | Sang sur héparine: **BH 1** |
| **PH** | Plasma sur héparine: **PH 1 – PH 2** |
| **BE** | Sang sur EDTA: **BE 1 – BE 2 – BE 3** |
| **PE** | Plasma sur EDTA: **PE 1 – PE 2** |
| **BD** | Sang (tube sec): **BD 1** |
| **SE** | Sérum: **SE 1 – SE 2** |
| **UR** | Urine: **UR 1 – UR 2 – UR 3** |
| **CS** | Liquide céphalorachidien (LCR): **CS 1 – CS 2 – CS 3** |
| **CC** | Culture de liquide céphalorachidien (LCR)<br>**CC 1**: BactAlert<br>**CC 2**: Trans-isolate<br>**CC 3**: MGIT |
| **OB** | Organismes (bactéries) isolées du sang |
| **OC** | Organismes (bactéries) isolées du liquide céphalorachidien (LCR) |

| | |
|---|---|
| nidiag — Better Diagnosis of Neglected Infectious Diseases | **SOP titre:** Gestion des documents de l'étude |
| | **Projet/étude:** Cette SOP s'applique à toutes les etudes NIDIAG |

# 1. Champ d'application

Cette procédure donne une liste de tous les documents essentiels qui doivent être collectés par les équipes de recherche pour les études cliniques NIDIAG. Elle décrit aussi comment ces documents doivent être gérés, conservés et archivés.

# 2. Responsabilités

| Fonction | Activités |
|---|---|
| Investigateur Principal dans chaque site | - Compiler tous les documents essentiels relatifs aux travaux exécutés sur le site d'étude et les conserver dans le Site Investigator File (SIF)<br>- S'assurer que le SIF est maintenu à jour<br>- S'assurer que tous les documents essentiels sont conservés dans les conditions adéquates et pour la période requise<br>- S'assurer que l'accès aux documents essentiels est strictement réservé au personnel autorisé<br>- S'assurer que des copies de tous les documents essentiels produits sur le site soient envoyé au pays coordinateur (excepté les documents qui portent une identification de patient) |
| Chef de laboratoire dans chaque site | - Compiler tous les documents essentiels relatifs aux travaux de laboratoire exécutés sur le site et les conserver dans le Laboratory File (LF)<br>- S'assurer que le LF est maintenu à jour<br>- S'assurer que tous les documents essentiels sont conservés dans les conditions adéquates et pour la période requise<br>- S'assurer que l'accès aux documents essentiels est strictement réservé au personnel autorisé<br>- S'assurer que des copies de tous les documents essentiels produits au laboratoire du site sont envoyées au coordinateur du pays (excepté les documents qui portent une identification de patient) |
| Investigateur coordinateur du pays (Country Coordinating Investigator) | - Compiler tous les documents essentiels relatifs aux travaux exécutés dans le pays et les conserver dans le Investigator Master File (IMF)<br>- S'assurer que le IMF est maintenu à jour<br>- S'assurer que tous les documents essentiels sont conservés dans les conditions adéquates et pour la période requise<br>- S'assurer que l'accès aux documents essentiels est strictement réservé au personnel autorisé<br>- S'assurer que des copies de tous les documents essentiels produits sur les différents sites du pays et ceux produits par la coordination soient partagés avec le WP2 Task Leader et que les sites reçoivent toute l'information essentielle nécessaire pour leur travail |
| WP2 Task Leader | - Compiler tous les documents essentiels relatifs au travail exécuté dans chaque « task » (Syndrome Digestif, Syndrome Fébrile et Syndrome Neurologique) et les conserver dans le Sponsor Master File (SMF). Cette responsabilité peut être déléguée, et la délégation doit être documentée. |

| | |
|---|---|
| | - S'assurer que la SMF est tenue à jour<br>- S'assurer que tous les documents essentiels soient conserves sous des conditions appropriés et pour la période de temps requise<br>- S'assurer que l'accès aux documents essentiels est strictement réservé au personnel autorisé<br>- Assurer que l'information nécessaire est partagée avec l'investigateur coordinateur du pays |
| Responsable Qualité (Site Quality manager(QM) ou équivalent), et Moniteur Externe | - Vérifier que ce SOP soit respecté par tous les destinataires, et plus spécifiquement<br>a. vérifier que la IMF, SIF et LF soient complets et à jour<br>b. vérifier que l'accès est strictement réservé aux individus autorisés<br>- Signaler toutes les déviations majeures ou systématiques de cette SOP aux chefs des WP2 et WP6 |

# 3. Procédures

## 3.1 Compilation et gestion des documents essentiels de l'étude:

Une liste de tous les documents à collecter et archiver se trouve en fin de ce POS. Les documents d'étude doivent être gardés dans 4 classeurs différents :

- Laboratory File (LF): Le « Laboratory File » comprend tous les documents essentiels relatifs au travail de laboratoire sur le site. Il n'y a qu'une seule Laboratory File par site d'étude. Le personnel de laboratoire est responsable pour garder ce dossier à jour et doit le conserver de façon correcte.
- Site Investigator File (SIF): Le « Site Investigator File » comprend tous les documents essentiels relatifs à l'exécution de l'étude sur le site. Il n'y a qu'une seule SIF par site d'étude. Les Investigateurs Principaux dans chaque site sont responsables pour garder ce dossier à jour et doivent le conserver de façon correcte.
- Country Investigator's Master File (IMF): Le « Country Investigator's Master File » comprend tous les documents essentiels relatifs à la mise en œuvre de l'étude – au niveau pays. Il n'y a qu'une seule IMF par pays. L'investigateur coordinateur du pays est responsable pour garder ce dossier à jour et doit le conserver de façon correcte.
- Sponsor Master File (SMF): Le « Sponsor's Master File » comprend tous les documents essentiels relatif à l'étude selon les guidelines ICH GCP. Il n'y a qu'une seule Sponsor Master File par syndrome clinique. Le Task Leader, ou la personne à laquelle il a délégué la fonction, est responsable pour garder ce dossier à jour et doit le conserver de façon correcte.

Voir le Tableau 1 pour une information plus détaillée sur quel document doit être archivé dans quel classeur. Archivez les documents dans l'ordre spécifié au Tableau 1.

## 3.2 Conservation et accès aux documents de l'étude

Tous les documents doivent être conservés dans un endroit sûr.

Le « Laboratory File » doit être gardé au laboratoire. Le « Site Investigator File » doit être conservé sous la responsabilité de l'investigateur principal dans chaque site. Le « Investigator's Master File » doit être conservé sous la responsabilité de l'investigateur coordinateur du pays. Le « Sponsor Master File » doit être conservé sous la responsabilité du Task Leader du Syndrome respectif.

L'accès aux documents de l'étude doit être strictement reserve au personnel impliqué dans NIDIAG.

- L'accès au « Site Investigator File » est réservé au personnel impliqué directement dans les soins au malade (infirmiers de l'étude, investigateurs du site, etc…)

- L'accès au « Laboratory File » est réservé au personnel de laboratoire (techniciens de laboratoire, chef de laboratoire, etc…) et superviseurs, y compris le l'investigateur coordinateur du pays et le responsable qualité (ou fonction équivalente)
- L'accès au « Investigator's Master File » doit être reservé a l'investigateur coordinateur du pays et son équipe
- L'accès au « Sponsor Master File » doit être reservé au Task Leader et son équipe.

Le personnel de l'étude doit prévenir la destruction accidentelle des documents de l'étude. Ceci inclut la protection contre les incendies et inondations.

A leur demande et sous un accord de confidentialité, les site Managers de Qualité du site, moniteurs de l'études, auditeurs externes, inspecteurs (autorités nationales regulatoires) et Comités Ethiques peuvent être donnés un accès direct à tous les documents de l'étude.

## 3.3 Entretien

- Tous les documents d'étude doivent être tenua à jour. L'investigateur Principal dans chaque site et l'investigateur coordinateur du pays doivent, entre autres, s'assurer que la version la plus récente du protocol, Formulaire de Consentement Eclairé (FCE), Case Report Form (CRF) et Standard Operating Procedures (SOP/POS) soient utilisés. Les Investigateurs dans chaque site sont responsables de conserver tous les documents mentionnés dans l'Annex 1 à jour et complets.
- Le chef de laboratoire doit vérifier régulièrement que, entre autres, les valeurs normales incluses dans le « Laboratory File » correspondent à celles utilisées pour les analyses, que le registre des échantillons de l'étude (the study specimen log),et l'enregistrement des échantillons conservés soient mis à jour, càd en accord avec le recrutement de l'étude, et que l'information concernant l'expédition des échantillons vers les laboratoires de référence soient correctement enregistré. Le chef de laboratoire est aussi responsable pour la maintenance de tous les documents de laboratoire listés en Annex 1.
- L'investigateur coordinateur du pays ou son délégué est responsable du maintien de tous les documents du « Investigators Master File » listés sous Annex 1. L'investigateur coordinateur du pays doit s'assurer que les changements dans les documents essentiels soient communiqués à temps au Investigateur Principal dans chaque site et au WP2 Task Leader si relevant.
- Le Task Leader et ses délégués doivent assurer que le contenu du Sponsor Master File soit régulièrement mis à jour et correspond à l'état d'avancement de l'étude dans chaque pays partenaire.

Tous les changements qui surviennent pendant l'étude doivent être documentés dans le SIF, LF (par ex: si on recrute une nouvelle personne pour l'étude, alors il faudra ajouter le CV de cette personne, établir un nouveau contrat, et actualiser l'organigramme de l'étude et la feuille de signatures).

## 3.4 Archivage

L'investigateur Principal dans chaque site doit conserver le « Site Investigator File » jusqu'à ce que le Country Coordinating Investigator le reprenne à la fin de l'étude.

Le chef de laboratoire doit garder le Laboratory File jusqu'à ce que l'investigateur coordinateur du pays le reprenne à la fin de l'étude.

L'investigateur coordinateur du pays doit garder le « Country Investigator's Master File » et tout autre document relatif à l'étude pendant la période prescrite par les autorités régulatrices du pays et au moins 5 ans après la terminaison de l'étude.

Le Task Leader doit conserver le Sponsor's Master File et tout autre document de l'étude pendant une période de temps qui correspond aux normes des autorités régulatrices du pays et jusqu'à au moins 5 ans après la fin de l'étude.

## 4. Définitions and abbréviations

Documents Essentiels: Des documents qui de façon individuelle et collective permettent d'évaluer la conduite d'une étude et la qualité des données produites. Ces documents sont importants pour la réussite de l'étude. Ils sont aussi essentiels pour démontrer la compliance avec les normes GCP/GCLP, le protocole, les POSs et pour permettre le contrôle de qualité. Une liste de documents essentiels est donnée dans l'Annex 1.

Documents Source: Ce sont des documents originaux. Ils consistent de documents originaux, comme des dossiers médicaux, des dossiers administratifs, des rapports de laboratoire, notes de consultation, registre de pharmacie etc ...

CRF: Case Report Form

ICF: Informed Consent Form

IMF: Investigator Master File

LF: Laboratory File

SIF: Site Investigator File

SMF: Sponsor Master File

SOP/POS: Standard Operating Procedure / Procédure Opératoire Standardisée

## 5. Archivage

| **Appendices & Forms for completion** | |
|---|---|
| **Number** | **Title** |
| NA | NA |

## 6. Historique du document

| **Révision** | |
|---|---|
| SOP-WP6-DOC-03-V01-24May2012 | Version initiale |
| SOP-WP6-DOC-03-V01.1-18June2012 | Traduction de V01 de l'Anglais en Français |

| **Nom et fonction** | **Date** | **Signature** |
|---|---|---|
| *Auteur (traduction SOP-WP6-DOC-03-V01-24May2012)* | | |
| Pascal Lutumba | 18 Juin 2012 | |
| *Revisé par* | | |
| | | |
| *Approuvé par* | | |
| Veerle Lejon | 18 Juin 2012 | |

**Tableau 1:** Liste des documents essentiels à conserver dans le SIF, LF

| Titre du document | SIF | LF | IMF | SMF |
|---|---|---|---|---|
| **1. Documents de l'étude** | | | | |
| Protocole de l'étude signé et amendements | x | x | x | x |
| Une copie vierge du Case Report Form (CRF) | | | x | x |
| Une copie vierge du Informed Consent Form (ICF) ainsi que toute information donnée aux participants de l'étude | X | | x | x |
| Study SOPs | x | x | x | x |
| Nidiag ethical charter | X | | X | X |
| **2. Documents régulatoires** | | | | |
| Toute la correspondance avec le IRB de ITM et EC de l'Université d' Anvers (UZA) y compris l'approbation finale du protocole d'étude et de ses amendements | x | | | x |
| Toute correspondence avec les comités éthiques nationaux IRB/EC y compris l'approbation finale du protocole d'étude et de ses amendements | x | | x | x |
| Autorisation des autorités régulatrices nationales (si nécessaire) | x | | x | x |
| Rapports intermédiaires aux ECs/IRBs | | | x | x |
| Autorisations d'importations pour les tests rapides (RDTs) (si nécessaire) | | | x | x |
| Document de l'assurance | | | x | x |
| **3. Personnel de l'étude et contrats** | | | | |
| Document concernant la délégation des taches (mis à jour avec dates et signatures) de tout le personnel sur le site de l'étude, y compris le laboratoire | x | | x | |
| Document concernant la délégation des taches (mis à jour avec dates et signatures) de tout le personnel au niveau du Sponsor | | | | x |
| Liste des contacts sur le site | X | | x | x |
| Liste des contacts de sponsor (par protocole) | X | | X | X |
| CVs de tout le personnel impliqués dans l'étude (investigateurs, sous-investigateurs, techniciens de labo, etc…) | x | | x | x |
| Feuille de signature de tout le personnel autorisé sur le site, y compris le laboratoire | x | | x | x |
| Certificat de formation GCP/GCLP pour tout le personnel impliqué dans l'étude y compris le laboratoire | x | | x | x |
| Copie du NIDIAG grant agreement | | | x | x |
| Accord signé entre le personnel de l'étude et institution partenaire | | | x | x |
| 4. **Gestion des RDTs** | | | | |
| Documentation de l'expédition des RDTs (y compris les documents de dédouanement) | x | | x | x |
| Gestion de stock des RDT sur le site | | x | x | |
| 5. **Echantillons Biologiques** | | | | |
| Valeurs normales / range spécifiques pour le pays pour procédures de laboratoire | | x | x | x |
| Documents relatifs au Contrôle de Qualité des Procédures de Laboratoire | | x | x | |

| | | | | |
|---|---|---|---|---|
| Documents d'expédition des échantillons biologiques (aux laboratoires nationaux et internationaux de référence) | | x | x | |
| Registre de specimens d'étude, y compris les documents sur les échantillons biologiques retenus | | x | x | |
| Diagramme du circuit des échantillons spécifique par pays | | x | x | x |
| Formulaire de résultats des RDTs | | x(2) | x(1) | |
| Registre des temperatures mis à jour | | x | | |
| **6. Monitoring** | | | | |
| Monitoring Plan | | | x | x |
| Visite d'initiation de l'étude ou lettre de suivi | x | | x | x |
| Rapports de visites de monitorage ou lettre de suivi | | | | x |
| Close-out Monitoring Report ou lettre de suivi | | | | x |
| **7. Participants à l'étude** | | | | |
| Liste d'identification des patients | x | | x | |
| Tous les documents de consentement éclairé et d'assentiment | x | | | |
| Documents Source (y compris les dossiers médicaux et les résultats d'investigations de laboratoire) | x(2) | x(2) | x(1) | |
| CRFs mise à jour, signés et dates | x(1) | | x(2) | |
| **8. Correspondence** | | | | |
| Toute la correspondence essentielle entre le Trial Management Group et/ou le country coordinating investigator and/or the site(s) | x | X | x | x |

(1) Copies (2) Originaux

SIF: Site Investigator File; LF: Lab File; IMF: Investigator Master File; SMF: Sponsor Master File

| | |
|---|---|
| nidiag Better Diagnosis of Neglected Infectious Diseases | **SOP titre:** Comment écrire des Procédures Opératoires Standardisées (SOP) |
| | **Projet/étude**: Cette SOP s'applique à toutes les etudes NIDIAG |

# 1. But et application

Cette procédure fournit une ligne directrice sur la façon d'écrire une procédure opératoire standard (SOP), y compris la façon de formater le document. Le but d'une SOP est de fournir des instructions détaillées sur la façon d'effectuer une tâche pour que n'importe quel membre de l'équipe puisse effectuer la tâche correctement à chaque fois. Le but ou l'objectif d'une SOP devrait reformuler et élargir un titre bien écrit. Une SOP bien écrite facilitera la formation. La meilleure SOP est celle qui transfert précisément les informations appropriées et facilite la conformité avec la lecture et l'utilisation de la SOP. Cette SOP des SOPs est destinée aux chefs des WP, aux chefs des tâches et ceux qui seront impliqués dans l'écriture des SOPs. Elle s'applique à toutes les SOP développées par le consortium NIDIAG.

# 2. Responsabilités

| Fonction | Activités |
|---|---|
| Auteur de la SOP | ▪ Rédaction de la SOP en consultation avec les utilisateurs auxquels elle est destinée.<br>▪ Correction de la SOP en fonction de la réaction du WP6.<br>▪ Rendre la SOP disponible à tous les utilisateurs auxquels elle est destinée<br>▪ Modifiez (Amendez) la SOP si nécessaire. |
| Représentant du WP6 | ▪ Faire une révision du draft initial de la SOP et des amendements antérieurs.<br>▪ Divulguer et approuver formellement les versions de la SOP.<br>▪ Rendre la SOP disponible au consortium via le site Web NIDIAG. |
| Directeur (Manager) de Qualité de Site et<br>Moniteur Externe | ▪ S'assurer de la conformité de la SOP pour tous les utilisateurs auxquels elle est destinée.<br>▪ S'assurer que la version de la SOP utilisée est la plus récente et qu'elle est approuvée par le WP6<br>▪ Faire rapport au PI et au WP6 en cas de non adhérence aux SOPs. |

# 3. Procédures

## 3.1 Écriture d'une SOP

- Écrire une SOP par activité de l'étude. Exemple : Réalisation de la ponction lombaire, le traitement, le transport et le stockage des échantillons de liquide céphalorachidien, la détection des trypanosomes par microscope etc. Ne pas mélanger trop d'activités dans une SOP.
- S'assurer que vous êtes familiers avec la procédure à décrire dans la SOP. Si ce n'est pas le cas, demandez à quelqu'un qui exécute la procédure régulièrement de vous la montrer. Faites lire le brouillon à cette personne avant de le soumettre au WP6 pour révision.
- Décrivez en détail comment la procédure sera exécutée.
- Inscrivez les étapes dans un ordre chronologique comme dans l'exemple ci-dessous.

<u>Préparation d'une tasse de thé</u> :

1. Rassembler une tasse et une soucoupe.

2. Placer le sachet de thé dans la tasse.
3. Faire bouillir de l'eau dans la bouilloire.
4. Ajouter de l'eau dans la tasse et le sachet de thé.
5. Laisser le thé infuser.
6. Retirer le sachet de thé.
7. Ajouter du lait et sucre (si désiré).

- Utilisez un langage simple, en langage d'action par exemple ' pesez 10 mg ' plutôt que ' 10 mg devraient être pesés '.
- Indiquez dans la section "Responsabilités" les responsabilités de chacun (qui fait quoi). N'utilisez pas le nom des gens, utilisez plutôt les fonctions des utilisateurs / l'intitulé du poste par exemple : le technicien de laboratoire, le médecin …
- Incluez toutes les informations nécessaires pour exécuter la procédure, pas plus.
- Utilisez le moins de mots possibles, si des étapes différents sont impliquées dans une même activité, utilisez des tirets ·
- Si possible, ajoutez des informations visuelles comme des diagrammes, des organigrammes, des images ou des tableaux.
- Avoir un lecteur spécifique à l'esprit. Connaissez le type de personne qui lira la SOP et en écrivant, adaptez-la à la personne à laquelle elle est destinée.
- Évitez "faîtes ceci ou faîtes cela en alternative".
- Évitez "quand c'est nécessaire".
- S'assurer que tous les termes techniques et les acronymes sont définis dans la section "définition".

## 3.2 Format de la SOP

- Utiliser le modèle de SOP fourni par le WP6. Le modèle est disponible sur le site de NIDIAG
(www.nidiag.org ) ·
- Chaque SOP devrait avoir un identifiant unique qui comprend :
  - Le numéro du groupe des activités sous lequel la SOP est entrain d'être développé,
  - Un acronyme se référant au type de procédure (LAB : pour la SOP de laboratoire, DOC : pour la SOP destinée à gestion de la documentation, CLIN : pour la SOP clinique, DATA : pour la SOP liée à la gestion des données, QUAL : pour la SOP en rapport avec l'assurance qualité et le control de qualité,
  - le numéro de la SOP.

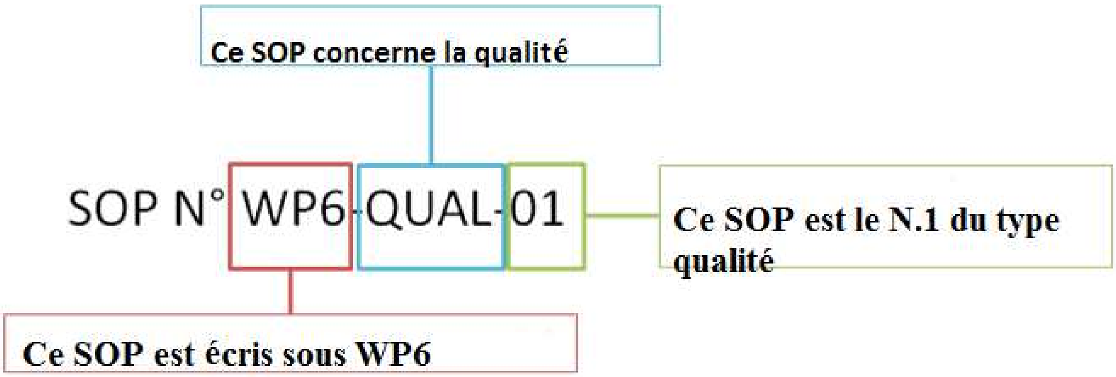

- Si la procédure est longue, la description des procédures peut être divisée et écrite avec des titres plus petits. Par exemple ' 3.1 Matériels, 3.2 Préparation des réactifs, 3.3. Opération et maintenance,….
- A chaque page de la SOP indiquer :
  - Le numéro de la SOP, le numéro de la version et la date de la version.
  - Le numéro de page et le nombre total de pages.

### 3.3 Révision des SOP et contrôle des versions

- Chaque SOP devrait être revue par un représentant autorisé du WP6. Le premier brouillon doit circuler comme la version 0. Les commentaires et les corrections du WP6 doivent être incorporés dans ce brouillon pour créer la version 1. Le WP6 est responsable de l'approbation finale (le OK final) du document.
- La SOP doit être signée par l'auteur de la SOP et par la personne qui la revoit et l'approuve.
- Les corrections et les modifications de la version initiale doivent aussi être soumises pour examen au WP6. La version 2 devrait être créée seulement après obtention des remarques et de l'approbation du WP6.
- Chaque version ultérieure consécutive et les raisons de chaque modification faite à la SOP doivent apparaître dans la section "Histoire du Document" à la fin de la SOP.

### 3.4 Langue des SOP

Les SOPs seront écrites en anglais. Cependant, les SOP destinées aux francophones seront traduites en français.

## 4. Définitions

**Investigateur Principal (PI)**: Personne responsable de la conduite globale de l'étude sur un site donné. L'investigateur principal est le leader responsable de l'équipe impliquée dans le projet NIDIAG.

**Procédures Opératoires standardisées (SOP/POS):** les SOP sont publiées pour instruire spécifiquement des employés/ des membres de l'équipe dans des domaines de responsabilité, les instructions de travail, le cahier des charges (les spécifications) appropriés et les rapports exigés.
Les SOPs décrivent des procédures, qui doivent être suivies pour pouvoir s'accorder aux principes de Bonnes Pratiques Cliniques et de Bonnes Pratiques de Laboratoire ou à d'autres règles statutaires et règlements. Les procédures peuvent prendre la forme d'un récit, d'un organigramme, d'une carte du processus, de l'impression d'une partie d'écran d'ordinateur ou la combinaison de toute autre forme appropriée, cependant, les SOPs doivent être écrites dans un style grammatical approprié et effectif. (Par exemple anglais/français simple).

**Formulaire**: un formulaire est un document qui doit être imprimé au moment de l'utilisation et rempli dans le but de devenir un rapport (par exemple : formulaire du journal de la bibliothèque, ou pour le but de devenir un outil de présentation Visuel.

**Modèle**: un modèle est un formulaire devant être utilisé comme un modèle pour créer d'autres documents.

**Présentation Visuelle**: une présentation visuelle est une forme n'exigeant l'ajout d'aucune donnée supplémentaire, (c'est-à-dire aucun rapport écrit), qui fournit des informations visuelles pour décrire le processus, par exemple "En panne" l'étiquette collée sur une machine. Les informations peuvent être en forme d'images ou des photographies; organigramme; mode d'emploi; ou avis.

La forme de présentation visuelle est d'habitude localisée dans une position permanente, ou peut être en utilisation pendant une période spécifique, par exemple pour un seul lot. Les pages provenant d'une forme de présentation visuelle unique doivent être placées ensemble dans un emplacement indiqué.

## 5. Rapports et archives

| **Appendices & Formulaires à remplir** | |
|---|---|
| **Numéro** | **Titre** |
| NA | NA |

## 6. Histoire du document

| **Révision** | |
|---|---|
| SOP-WP6-QUAL-01-V1-02Nov2011 | Version initiale |
| SOP-WP6-QUAL-01-V01.1-09Jul2012 | Traduction de V1 de l'Anglais en Français |

| **Nom et fonction** | **Date** | **Signature** |
|---|---|---|
| *Auteur (traduction SOP-WP6-DOC-01-V01-01Feb2012)* | | |
| Pascal Lutumba | 06 Juillet 2012 | |
| *Approuvé par* | | |
| Veerle Lejon | 09 Juillet 2012 | |

| | |
|---|---|
| nidiag — Better Diagnosis of Neglected Infectious Diseases | **SOP titre:** Monitorage externe |
| | **Projet/étude:** Cette SOP s'applique à toutes les études cliniques du projet NIDIAG (WP2). |

# 1. Domaine d'application

Les visites de monitorage sont effectuées tout au long des études cliniques NIDIAG pour s'assurer que:

*1)* les droits et le bien-être des sujets de recherche sont protégés
*2)* l'étude est réalisée conformément aux BPC, aux exigences réglementaires et au protocole de l'étude
*3)* les données de l'étude sont exactes, complètes et vérifiables à partir des documents sources.

Cette procédure décrit comment des visites de monitorage externes sont effectuées, documentées et suivies, conformément aux principes des Bonnes Pratiques Cliniques de l'OMS et de l'ICH.

# 2. Responsabilités

| Fonction | Activités |
|---|---|
| Moniteur Externe | ▪ Le plan de visites de monitorage se fait conformément au plan de monitorage, comme convenu avec le chef du WP6.<br>▪ Vérifier que l'étude se déroule conformément aux exigences réglementaires/éthique nationales et internationales, avec le protocole de l'étude et ses amendements, et avec les SOP NIDIAG.<br>▪ Rapporter toute déviation dans le rapport de visite de monitorage.<br>▪ Envoyer le rapport de visite de monitorage au responsable du WP6 concerné. |
| Gestionnaire de la Qualité (QM) ou fonction équivalente | ▪ Soutenir le moniteur externe dans l'accomplissement de ses tâches.<br>▪ Assister aux visites de monitorage. |
| Investigateur principal du Site | ▪ Soutenir le moniteur externe dans l'accomplissement de ses tâches.<br>▪ Assister aux visites de monitorage et s'assurer que tout le personnel clé de l'étude est présent.<br>▪ S'assurer que le moniteur externe a accès aux documents et aux installations de l'étude.<br>▪ Veiller à ce que les mesures correctives énumérées dans le rapport de visite de monitorage et/ou la lettre de suivi soient mises en œuvre. |
| Investigateur coordinateur du pays ou son/sa délégué(e)<br><br>Chef du WP2 ou son/sa délégué(e) | ▪ Vérifier que les actions correctives énumérées dans le rapport de visite de monitorage et/ou la lettre de suivi sont mises en œuvre (un suivi conjoint aura lieu au sein du groupe de gestion des essais de l'étude clinique spécifique). |
| Chef du WP6 ou son/sa délégué(e) | ▪ Effectuer le lien entre le Groupe de la gestion des essais et le moniteur.<br>▪ Mettre en place un plan de monitorage pour chaque site d'étude.<br>▪ S'assurer que le moniteur reçoit toute nouvelle information |

| | |
|---|---|
| | clé pertinente relative au statut et à la conduite de l'étude.<br>▪ Examiner les rapports des visites de monitorage et/ou les lettres de suivi.<br>▪ Envoyer en temps opportun une copie du rapport de visite de monitorage et/ou la lettre de suivi au chef du WP2 ou à son/sa délégué(e), à l'investigateur coordinateur du pays, et aux membres concernés du groupe de gestion de l'essai.<br>▪ Envoyer une copie du rapport de visite de monitorage et/ou de la lettre de suivi à l'investigateur principal du site. |

# 3. Procédures

## 3.1 Calendrier de monitorage et planification de la visite

Le chef du WP6, en consultation avec l'investigateur coordinateur du pays, le chef du WP2 et les membres concernés du Groupe de gestion de l'essai, déterminera le calendrier approprié des visites de monitorage pour chaque étude clinique (ex : plan de monitorage du pays).

Le moniteur est responsable de la planification des visites de monitorage conformément au plan de monitorage établi.
Avant la visite, le moniteur doit:

- Revoir le protocole de l'étude, le CRF, les outils de monitorage (le rapport de visite de monitorage et/ou les lettres de suivi, les SOPs NIDIAG,….) les rapports précédents, et tout autre document clé remis par le chef du WP6.
- Informer le chef du WP6 et le PI du site au moins 2 semaines avant la visite de monitorage, et leur demander de veiller à ce que tout le personnel clé soit présent pendant la visite.
- Envoyer un programme de visite provisoire, en accord avec le chef du WP6 et le PI du site.
- Discuter avec le chef du WP6 des derniers développements de l'étude et s'accorder sur les lignes de communication rationnelle, de manière à ce que le chef du WP6 puisse communiquer avec le moniteur si nécessaire pour le suivi des observations issues de la visite.
- Se renseigner sur le nombre de patients inclus dans le site d'étude depuis la dernière visite.
- S'assurer que le temps disponible pour la visite de monitorage est suffisant pour compléter toutes les activités prévues.

## 3.2 Visite d'initiation de l'étude

Elle est réalisée après réception de l'ensemble du matériel de l'étude, après l'approbation du CE et des AC, et avant le début du recrutement. Au minimum, les activités suivantes doivent être réalisées:

*Formation à l'éthique et aux BPC:* le moniteur doit rencontrer le PI du site ainsi que le personnel de l'étude, afin de vérifier qu'ils ont une bonne compréhension des exigences éthiques et des BPC. Si nécessaire, il/elle effectuera une formation sur les BPC ou une séance de remise à niveau.

*Revue du Protocole et du CRF :* le moniteur passera en revue le protocole et le CRF avec tous les investigateurs et l'équipe de l'étude.

*Site Investigator File (SIF) et Dossier du Laboratoire*: le moniteur vérifiera l'exhaustivité de ces deux dossiers (réf. SOP No WP6-DOC-03 sur la gestion des documents de l'étude). Si logistiquement réalisable et si le temps le permet, le moniteur pourra également vérifier la conformité de l'Investigator Master File (IMF) ou Dossier principal de l'étude.

*Visite des installations du site* : le moniteur visitera les installations du site, y compris le laboratoire, pour confirmer qu'ils sont adéquats pour la conduite des activités de l'étude. Il/elle doit également vérifier qu'un espace suffisant et un équipement adéquat sont disponibles pour le stockage du matériel de l'étude, pour l'archivage du SIF et des documents sources.

*Rapport:* le moniteur documentera les points mentionnés précédemment dans un rapport de visite de monitorage, qui sera ensuite envoyé au chef/délégué du WP6. Il est de la responsabilité du chef du WP6 de le distribuer en temps opportun à l'investigateur coordinateur du pays, au chef/délégué du WP2, et aux membres concernés du Groupe de gestion de l'essai et à l'investigateur principal du site pour le suivi des résultats et les mesures correctives.

*Exceptions:* le chef du WP6 peut décider de sauter la visite d'initiation de l'étude si tous les points ont été pris en compte et documentés lors des visites précédentes et que des mesures correctives ont été implémentées.

### 3.3. Visites de monitorage de routine

La première visite de suivi a lieu le plus tôt possible, et au plus tard un mois après le début du recrutement, de sorte que les erreurs importantes ou les problèmes systématiques soient identifiées et corrigées dans les premières phases de l'étude. Au minimum, les activités suivantes devront être réalisées:

*Suivi de la visite précédente* : le moniteur s'assurera du suivi des observations relevées lors des visites précédentes.

*Nouveau personnel :* si de nouveaux employés ont été nommés, le moniteur vérifiera le CV, le journal de délégation des tâches et les certificats de formation. Si nécessaire, il/elle effectuera une séance de formation/remise à niveau aux BPC.

*Progression de l'étude* : le moniteur discutera du statut de recrutement (prévus vs atteint) avec l'investigateur principal du site. Il/elle vérifiera également si la procédure d'orientation des patients fonctionne de façon satisfaisante.

*Consentement éclairé* : le moniteur vérifiera que le consentement éclairé a été obtenu et qu'il est bien documenté pour chaque patient avant /elle subisse quelconque procédure spécifique à l'étude (réf : SOP WP6-DOC-01 sur le consentement éclairé).

*Conformité avec le protocole* : le moniteur vérifiera la conformité globale de l'étude avec le protocole et ses amendements. Tout écart(s) grave(s) et/ou systématique(s) sera(seront) porté(s) à l'attention de l'investigateur coordinateur du pays et du chef du WP2. Une action corrective devra être planifiée.

*CRF* : le moniteur fournira des conseils et un soutien sur les procédures à suivre pour le remplissage du CRF, et (quand cela est approprié) pour effectuer la saisie des données.

*Vérification des données sources (SDV) :* Le Moniteur examinera les dossiers des patients, afin de s'assurer que les informations entrées dans le CRF correspondent aux observations originales que l'on retrouve dans les registres de l'hôpital, les résultats de laboratoires. Le CRF doit être cohérent et précis. La SDV est réalisée sur la base d'un échantillon de CRFs (par exemple, sur 15 % de tous les CRFs) et le pourcentage doit être augmenté si les résultats ne sont pas jugés satisfaisants.

*SIF et Dossier du Laboratoire*: le moniteur vérifiera la complétion du SIF et du Dossier du Laboratoire (réf. SOP No WP6-DOC-03 sur la gestion des documents d'étude).

*Tests de diagnostic rapide* : le moniteur vérifiera que leur stockage, leur transport et leurs conditions de distribution sont appropriés, et vérifiera que les dates de péremption n'ont pas été dépassées. Il/elle examinera les fiches de stock.

*Rapport:* le moniteur documentera les points mentionnés précédemment dans un rapport de visite de monitorage, qui sera ensuite envoyé au chef/délégué du WP6. Il est de la responsabilité du chef du WP6 de le distribuer en temps opportun à l'investigateur coordinateur du pays, au

chef/délégué du WP2 et aux membres concernés du Groupe de gestion de l'essai et à l'investigateur principal du site pour le suivi des résultats et les mesures correctives (le chef du WP6 peut choisir d'envoyer au PI soit une lettre de suivi résumant les principales conclusions et les mesures correctives nécessaires ou une copie du rapport de monitorage lui-même).

### 3.4. Visite de clôture

Elle est réalisée après que la dernière visite de routine ait été faîte; toutes les données ont été saisies dans la base de données et toutes les questions ont été résolues. Au minimum, les activités suivantes devront être réalisées:

*Suivi de la visite précédente* : le moniteur s'assurera du suivi de toutes les actions en attente des précédentes visites. Un plan d'action sera adopté pour les mesures correctives ne pouvant pas être mises en œuvre pendant la visite.

*Evaluation finale de l'étude* : le nombre final de patients sélectionnés, recrutés, perdus de vue et ayant complétés l'étude, doit être vérifié et joint au rapport de la visite de clôture.

*Tests de diagnostiques rapides non utilisés* : le moniteur vérifiera les fiches de stock et effectuera un inventaire final pour s'assurer qu'il n'y a pas de divergences. Les tests qui sont enregistrés dans le pays peuvent être donnés à la pharmacie de l'hôpital. Pour les produits non enregistrés, des dispositions seront définies par le chef du WP6.

*SIF*: le moniteur vérifiera la complétion du SIF, et vérifiera qu'il est bien conservé chez l'investigateur coordinateur du pays. Le dossier sera alors disponible en cas audits ou d'inspections (réf. SOP-WP6-DOC-03 sur la gestion des documents de l'étude).

*Rapport*: le moniteur documentera les points mentionnés précédemment dans un rapport de clôture de visite, qui sera envoyé au chef /délégué du WP6. Il est de la responsabilité du chef du WP6 de le distribuer en temps opportun à l'investigateur coordinateur/délégué du pays, au chef/délégué du WP2, aux membres concernés du Groupe de gestion de l'essai et à l'investigateur principal du site pour le suivi des résultats et des mesures correctives (le chef du WP6 peut choisir d'envoyer au PI soit une lettre de suivi résumant les principales conclusions et les mesures correctives nécessaires ou une copie du rapport de visite lui-même).

*Exceptions*: le chef du WP6 peut décider de sauter la visite de clôture de site à condition que l'ensemble des points mentionnés ci-dessus aient été pris en compte et documentés lors des visites précédentes (par exemple, par le responsable du laboratoire) et que des mesures correctives aient été implémentées.

### 3.5. Remarque concernant la supervision du laboratoire

La plupart des moniteurs cliniques n'ont pas d'expertise spécifique sur les procédures liées au laboratoire. En raison des caractéristiques du projet NIDIAG, le monitorage clinique externe doit être associé à des visites de supervision régulières effectuées par des experts de laboratoire.

## 4. Définitions et abréviations

<u>Audit :</u> Vérification systématique, menée indépendamment de ceux qui sont directement impliqués dans l'étude clinique, pour déterminer si la conduite de l'étude est conforme au protocole et si les données reportées sont cohérentes avec la documentation sur place.

<u>Formulaire d'enregistrement des Cas (CRF)</u> : Document utilisé pour enregistrer les données de chaque sujet d'étude durant toute la durée de l'étude, tel que défini dans le protocole. Les données doivent être collectées selon des procédures qui garantissent la préservation, la conservation et la récupération d'informations et qui permettent un accès aisé pour la vérification, l'audit et l'inspection.

Conformité : Respect des exigences liées à la recherche, aux Bonnes Pratiques Cliniques et à toutes autres exigences réglementaires applicables.

Bonnes Pratiques Cliniques (BPC) : Normes pour les études cliniques qui englobe la conception, la conduite, le monitorage, la terminaison, l'audit, les analyses, les rapports et la documentation des études. Les BPC veillent à ce que les études soient scientifiquement et éthiquement conformes et que les propriétés cliniques du produit pharmaceutique (diagnostique, thérapeutique ou prophylactique) investigué soient bien documentés.

Consentement éclairé : Confirmation volontaire d'un sujet pour participer à une étude clinique donnée, et de sa documentation. Ce consentement doit être recherché une fois que le sujet a reçu toutes les informations importantes relatives à l'étude, y compris une explication de son statut de recherche, ses objectifs, ses avantages potentiels, ses risques et ses inconvénients, les traitements alternatifs disponibles, les droits et les responsabilités du sujet conformément à la révision actuelle de la Déclaration d'Helsinki.

Inspection : Vérification officielle menée par les autorités compétentes sur le site de l'étude et/ou sur le site du promoteur afin de s'assurer du respect des Bonnes Pratiques Cliniques.

Investigateur : Tout personnel médical qui est à un moment donné impliqué dans la conduite de l'étude et qui est responsable de l'étude, des droits, de la santé et du bien-être des sujets de l'étude. L'investigateur possède les qualifications et compétences requises par les lois et règlements locaux, et sont référencées dans un curriculum vitae à jour.

Moniteur : Personne désignée par le promoteur pour le monitorage, le compte-rendu de la progression de l'étude ainsi que la vérification des données.

Dossier Patient/sujet : Ensemble des données comprenant toutes les informations pertinentes sur le patient ou le sujet (comme le dossier médical, les rapports de consultation) permettant d'authentifier l'information reportée dans les formulaires de rapport de cas, devant être vérifiés et, si nécessaire, complétés ou corrigés.

Investigateur principal : Investigateur servant de coordinateur au sein de chaque site d'étude.

Protocole : Document précisant le contexte, le rationnel et les objectifs de l'étude : il décrit sa conception, sa méthodologie et son organisation, y compris ses considérations statistiques, et les conditions dans lesquelles l'étude doit être exécutée et gérée. Le protocole doit être daté et signé par l'investigateur, l'institution concernée et le promoteur.

Amendement au protocole : Description écrite d'une modification ou une clarification formelle du protocole.

Données sources : Tous les dossiers ou les copies certifiées des observations originales, les résultats cliniques ou d'autres activités qui sont nécessaires à la reconstruction et à l'évaluation de l'étude clinique. Par exemple les notes de laboratoire, les notes de service, les calculs, les documents ainsi que les enregistrements de données provenant d'instruments automatisés ou exactes, des copies conformes de photocopies, des microfiches, etc. Les données brutes peuvent aussi inclure des négatifs photographiques, des microfilms ou des supports magnétiques (par exemple : disquettes).

Vérification des données sources : Procédures effectuées pour s'assurer que les données contenues dans le rapport final de l'étude sont conforment avec les observations originales. Ces procédures peuvent s'appliquer aux données brutes, aux données des formulaires de rapport de cas (en copie papier ou sous forme électronique), aux impressions d'ordinateur, aux analyses statistiques et aux tableaux.

Groupe de gestion de l'essai : Groupe de personnes et de fonctions du WP2 qui sont conjointement responsables de la mise en place appropriée, de la conduite et de la gestion quotidienne de l'essai clinique.

| | |
|---|---|
| AC | Autorités Compétentes |
| BPC | Bonnes Pratiques Cliniques |
| CE | Comité d'Ethique |
| CRF | Formulaire d'enregistrement des Cas (Case Report Form) |
| CV | Curriculum Vitae |
| EI | Evénement Indésirable |
| ICH | Conférence Internationale pour l'Harmonisation |
| IMF | Dossier principal de l'étude (Investigator Master File) |
| OMS | Organisation Mondiale de la Santé |
| PI | Investigateur Principal |
| QM | Gestionnaire de la Qualité (Quality Manager) |
| SDV | Vérification des Données Source |
| SIF | Dossier de l'étude du site (Site Investigator File) |
| SOP | Procédure Opératoire Standardisée |
| WP | Groupe de travail (Work Package) |

## 5. Enregistrement et archives

| **Appendices & Formulaire à compléter** | |
|---|---|
| **Numéro** | **Titre** |
| Annexe 1 | Rapport de visite de Monitorage |

## 6. Historique du Document

| **Révision** | |
|---|---|
| SOP-WP6-QUAL-02-V2-05Dec2012 | Dernière vérsion du SOP en Anglais |
| SOP-WP6-QUAL-02-V1.1-20Jan2015 | Traduction de la SOP en français |

| **Nom et fonction** | **Date** | **Signature** |
|---|---|---|
| *Auteur* | | |
| Raffaella Ravinetto | 5 Décembre 2012 | |
| *Révisé par* | | |
| Emilie Alirol | 5 Décembre 2012 | |
| *Traduit par* | | |
| *Equipe du Mali* : Moussa Sacko, Renion Saye | 29 Mai 2014 | |
| *Approuvé par* | | |
| Ninon Horié | 20 Janvier 2015 | |

**Annexe 1 : Rapport de visite de Monitorage**

| REPORT OF MONITORING VISIT N° | | | |
|---|---|---|---|
| Syndrome: | | | |
| Study protocol n°: | | | |
| Country Coordinating Investigator: | | | |
| Study Site : | | | |
| Country : | | | |
| Monitor : | | Visit date : | Previous Visit: |
| Visit type | ☐ Initiation | ☐ Routine | ☐ Close-out |

| STAFF | | | | | |
|---|---|---|---|---|---|
| | Name | Available at this visit | CV in the SIF? | GCP training? | Signed delegation log? |
| Principal Investigator | | ☐yes ☐no | ☐yes ☐no | ☐yes ☐no | ☐yes ☐no |
| Sub-Investigator | | ☐yes ☐no | ☐yes ☐no | ☐yes ☐no | ☐yes ☐no |
| Sub-Investigator | | ☐yes ☐no | ☐yes ☐no | ☐yes ☐no | ☐yes ☐no |
| Sub-Investigator | | ☐yes ☐no | ☐yes ☐no | ☐yes ☐no | ☐yes ☐no |
| Study nurses | | ☐yes ☐no | ☐yes ☐no | ☐yes ☐no | ☐yes ☐no |
| Study nurses | | ☐yes ☐no | ☐yes ☐no | ☐yes ☐no | ☐yes ☐no |
| Study nurses | | ☐yes ☐no | ☐yes ☐no | ☐yes ☐no | ☐yes ☐no |
| Head of Lab | | ☐yes ☐no | ☐yes ☐no | ☐yes ☐no | ☐yes ☐no |
| Lab technician | | ☐yes ☐no | ☐yes ☐no | ☐yes ☐no | ☐yes ☐no |
| Lab technician | | ☐yes ☐no | ☐yes ☐no | ☐yes ☐no | ☐yes ☐no |
| Lab technician | | ☐yes ☐no | ☐yes ☐no | ☐yes ☐no | ☐yes ☐no |
| Quality Manager | | ☐yes ☐no | ☐yes ☐no | ☐yes ☐no | ☐yes ☐no |
| Data management staff | | ☐yes ☐no | ☐yes ☐no | ☐yes ☐no | ☐yes ☐no |
| Other staff | | | | | |
| | | ☐yes ☐no | ☐yes ☐no | ☐yes ☐no | ☐yes ☐no |
| | | ☐yes ☐no | ☐yes ☐no | ☐yes ☐no | ☐yes ☐no |
| | | ☐yes ☐no | ☐yes ☐no | ☐yes ☐no | ☐yes ☐no |

| STAFF INVOLVED IN THE TRIAL | |
|---|---|
| Is the study team adequately trained and motivated to conduct the study? | ☐yes ☐no ☐NA |
| Is there sufficient time allocated to the study team to conduct the study efficiently? | ☐yes ☐no ☐NA |

| | |
|---|---|
| Was there any change in staff since the previous visit? | ☐yes ☐no ☐NA |
| Please document change; | |
| Has a signed and dated CV been filed in the SIF and collected for the IMF? | ☐yes ☐no ☐NA |
| Has the site delegation log been updated? | ☐yes ☐no ☐NA |
| Did the new staff member follow a documented GCP training? | ☐yes ☐no ☐NA |
| Has this person been sufficiently trained in all study documents? | ☐yes ☐no ☐NA |
| Additional Comments | |

| **GENERAL DISCUSSION** | |
|---|---|
| HAS THE FOLLOWING BEEN DISCUSSED WITH THE PI AND RELEVANT STUDY STAFF? | |
| Protocol/amendment? | ☐yes ☐no ☐NA |
| ICF and informed consent process? | ☐yes ☐no ☐NA |
| Investigator and study staff responsibilities? | ☐yes ☐no ☐NA |
| Handling and storage of CRF and Source Documents? | ☐yes ☐no ☐NA |
| Data collection? | ☐yes ☐no ☐NA |
| Participant's follow-up? | ☐yes ☐no ☐NA |
| Biological samples handling, transport and storage? | ☐yes ☐no ☐NA |
| Accountability of RDTs and other study materials? | ☐yes ☐no ☐NA |
| Additional Comments | |

| **MASTER FILES** | |
|---|---|
| SITE INVESTIGATOR FILE (SIF) | |
| Are all essential documents filed in the SIF? | ☐yes ☐no ☐NA |
| Was the SIF reviewed with the PI and relevant study staff? | ☐yes ☐no ☐NA |
| Were copies of all site specific documents collected for the IMF? | ☐yes ☐no ☐NA |
| Is the SIF kept in an appropriate place? | ☐yes ☐no ☐NA |
| Is it possible to lock the place? | ☐yes ☐no ☐NA |
| Is the access restricted to authorized staff? | ☐yes ☐no ☐NA |
| LABORATORY FILE (LF) | |
| Are all essential documents filed in the SIF? | ☐yes ☐no ☐NA |
| Was the LF reviewed with the lab head and relevant study staff? | ☐yes ☐no ☐NA |
| Were copies of all site specific documents collected for the IMF? | ☐yes ☐no ☐NA |
| Is the LF kept in an appropriate place? | ☐yes ☐no ☐NA |
| Is it possible to lock the place? | ☐yes ☐no ☐NA |
| Is the access restricted to authorized staff? | ☐yes ☐no ☐NA |
| COUNTRY INVESTIGATOR MASTER FILE (IMF) | |
| Are all essential documents filed in the SIF? | ☐yes ☐no ☐NA |

Was the LF reviewed with the lab head and relevant study staff? ☐yes ☐no ☐NA

Is the IMF kept in an appropriate place? ☐yes ☐no ☐NA

Is it possible to lock the place? ☐yes ☐no ☐NA

Is the access restricted to authorized staff? ☐yes ☐no ☐NA

Additional Comments

## RECRUITMENT (FOR ROUTINE AND CLOSE-OUT MONITORING VISITS ONLY)

### RECRUITMENT NUMBERS

Is the Patient Identification List sent to the country coordinating investigator on a regular basis? ☐yes ☐no ☐NA

Are the Screening and recruitment log sent to the country coordinating investigator and to the sponsor (WP2 leader) on a regular basis? ☐yes ☐no ☐NA

Number of patients included*

Number of patients withdrawn/dropped out*

Is reaching the recruitment target by the end of the recruitment period attainable? ☐yes ☐no ☐NA

If no; what actions will be taken to increase recruitment?

Additional Comments

### OVERALL CIRCUIT OF PATIENTS

How is screening organized?

How is recruitment organized?

Is patient's referral working satisfactorily? ☐yes ☐no ☐NA

If not, explain:

Is patient's follow-up working satisfactorily? ☐yes ☐no ☐NA

If not, explain:

Do study participants have access to proper care? ☐yes ☐no ☐NA

If not, explain:

Do screened but non-recruited patients have free access to all diagnostic procedures? ☐yes ☐no ☐NA

If not, explain:

Additional Comments

☐

## PROTOCOL, ICF & CRF

### INFORMED CONSENT FORMS (ICF)

Does the study team use the latest version of the ICF? ☐yes ☐no ☐NA

Is the informed consent procedure conducted properly? ☐yes ☐no ☐NA

If no, was the site staff retrained in the ICF procedure? ☐yes ☐no ☐NA

If yes, who attended this training?

Were ICF checked? ☐yes ☐no ☐NA

If yes, from patient n° until patient n°

Where all ICF correctly completed? ☐yes ☐no ☐NA

If no, please list deviations:

patient n° Deviation:

patient n° Deviation:

Patient(s) n° Deviation:

patient(s) n° Deviation:

patient(s) n° Deviation:

patient(s) n° Deviation:

patient(s) n° Deviation:

patient(s) n° Deviation:

### PROTOCOL

Was the overall compliance of the research conduct with the protocol and amendments verified? ☐yes ☐no ☐NA

Have protocol deviations been detected? ☐yes ☐no ☐NA

If yes, please list deviations:

Patient(s) n° Deviation:

patient(s) n° Deviation:

patient(s) n° Deviation:

patient(s) n° Deviation:

patient(s) n° Deviation:

patient(s) n° Deviation:

patient(s) n° Deviation:

patient(s) n° Deviation:

Are all protocol deviations documented? ☐yes ☐no ☐NA

Are there systematic errors to be reported? ☐yes ☐no ☐NA

If yes, please add which errors and what action was taken

Additional Comments

### CRF (FOR ROUTINE AND CLOSE-OUT MONITORING VISITS ONLY)

Was SDV performed during this visit? ☐yes ☐no ☐NA

If not, specify:
………………………………………………………………………

Had the Quality Manager performed 100% SDV prior to this visit? ☐yes ☐no ☐NA

If not, specify: ………………………………………………………………

If yes, CRF fully checked at this visit:

From patient n° until patient n° ; from patient n° until patient n° ; from patient n° until patient n°

Were CRF legible, accurate and complete? ☐yes ☐no ☐NA

Indicate estimated error rate: %

Are there any corrections pending? ☐yes ☐no ☐NA

If yes, specify:

For Close-Out Monitoring Visit Only:

Have all outstanding queries and issues been resolved? ☐yes ☐no ☐NA

Have copies of the final CRFs and data queries been filed? ☐yes ☐no ☐NA

Were other forms legible, accurate and complete?

Patient Identification List ☐yes ☐no ☐NA

Study Specimen Log ☐yes ☐no ☐NA

Additional Comments

**STUDY TESTS AND MATERIALS**

Where are the study diagnostic tests kept?

Who is responsible for the storage and distribution of the tests?

Are transportation, storage and dispensing conditions of tests appropriate? ☐yes ☐no ☐NA

Is the amount of tests left still sufficient? ☐yes ☐no ☐NA

Was any tests expired? ☐yes ☐no ☐NA

If yes, please indicate which test and action taken:

Are the accountability logs consistent with the number of recruited patients and the remaining stock? ☐yes ☐no ☐NA

Is the history of shipment reception of tests accurately documented? ☐yes ☐no ☐NA

Is there temperature control log in the facility? ☐yes ☐no ☐NA

Did the temperature exceed the allowed temperatures? ☐yes ☐no ☐NA

if yes, what action was taken?

Is there any concern about the quality of the study diagnostic tests? ☐yes ☐ no ☐ NA

if yes, please indicate which test and action taken:

Additional Comments

| LABORATORY | |
|---|---|
| EXPERT SUPERVISION | |
| WHEN WAS THE LAB LAST VISITED BY A NIDIAG LAB EXPERT? ……………..<br>WAS THE VISIT REPORT AVAILABLE? ……………………………………..<br>WERE THE EXPERT RECOMMENDATIONS IMPLEMENTED?<br>IF NOT, EXPLAIN WHY AND DESCRIBE FURTHER PLANS :……………………………… | |
| FACILITY | |
| Is the design and location of the laboratory facility appropriate to the study needs? | ☐yes ☐no ☐NA |
| Does the laboratory allow for separation of areas meant for study specimens reception, analysis and storage? | ☐yes ☐no ☐NA |
| Does the laboratory have back up facility in the event of power failure? | ☐yes ☐no ☐NA |
| Does the laboratory ensure safety to the work and the worker? | ☐yes ☐no ☐NA |
| Is access to data on paper, magnetic and other storage device restricted only to authorized personnel? | ☐yes ☐no ☐NA |
| Additional Comments | |
| EQUIPMENT AND MATERIAL | |
| Is the laboratory furnished with all necessary and appropriate items of equipment? | ☐yes ☐no ☐NA |
| Are the laboratory personnel trained in the operation of the equipment? | ☐yes ☐no ☐NA |
| Are the equipments cleaned and maintained well by the personnel? | ☐yes ☐no ☐NA |
| Are the equipments calibrated periodically and records of calibration documented? | ☐yes ☐no ☐NA |
| Are systems in place to detect faults in the equipment? e.g. temperature log for freezers, refrigerators and incubators, biological indicators for autoclave | ☐yes ☐no ☐NA |
| Are the materials / reagents used fit for purpose? | ☐yes ☐no ☐NA |
| Are the reagents labelled appropriately with the identity, concentration, date of preparation, date of opening and expiry, storage conditions? | ☐yes ☐no ☐NA |
| Are the reagents and materials stored under appropriate conditions? | ☐yes ☐no ☐NA |
| Additional Comments | |
| STUDY SPECIMENS | |
| Are study specimens stored in a safe manner (under lock and key custody)? | ☐yes ☐no ☐NA |
| Are the conditions for storage of samples sufficient? | ☐yes ☐no ☐NA |
| Did the storage temperature exceed the allowed temperatures? | ☐yes ☐no ☐NA |
| if yes, which samples were affected, and what action was taken? | |
| Are specimens received in the laboratory coded with a unique number to allow tracking of the sample from receipt to reporting? | ☐yes ☐no ☐NA |
| Are the SOPs followed in the performance of the sample analysis? | ☐yes ☐no ☐NA |

Are analytical procedures validated and performance checked periodically with suitable controls? ☐yes ☐no ☐NA

Are deviations in the performance of analytical procedures documented and reported to the lab director? ☐yes ☐no ☐NA

Are reference ranges available for each analytical procedure performed? ☐yes ☐no ☐NA

Does specimen transport follow proper organization, packaging, shipping to ensure integrity, timely and safe transfer? ☐yes ☐no ☐NA

Additional Comments

## DATA

Are data generated recorded directly, promptly and accurately? ☐yes ☐no ☐NA

Are data recorded in a lab notebook before being entered in the database? ☐yes ☐no ☐NA

If yes, please specify (a) if this concern a part of data or all data, (b) who enters the data into the study database and (c) who verifies the accuracy of transcription:

Is there a system for back up of electronic data as a routine? ☐yes ☐no ☐NA

Is access to the data restricted only to authorized personnel? ☐yes ☐no ☐NA

Additional Comments

## QUALITY CONTROL

Is the internal quality control (IQC) performed on a daily basis? ☐yes ☐no ☐NA

Has the IQC procedure been reviewed by all lab technicians? ☐yes ☐no ☐NA

Is an external quality control (EQC) performed on a regular basis? ☐yes ☐no ☐NA

If yes, are records of the EQC participation archived? ☐yes ☐no ☐NA

Additional Comments

## COMMENTS

| Monitor : | Reviewer : |
|---|---|
| Print name : | Print name : |
| Signature : | Signature : |
| Date : dd mmm yyyy | Date : dd mmm yyyy |

| PENDINGS – FOLLOW-UP ACTIONS REQUIRED | | | |
|---|---|---|---|
| Pending (Please repeat all lines until date done is completed.) | Responsible | Date Due | Date done |
| ***Staff*** | | | |
| | | | |
| ***Master Files*** | | | |
| | | | |
| ***Recruitment*** | | | |
| | | | |
| ***Protocol, ICF, CRFs*** | | | |
| | | | |
| ***Study Tests and materials*** | | | |
| | | | |
| ***Laboratory*** | | | |
| | | | |
| ***General*** | | | |
| | | | |

| Monitor : | Reviewer : |
|---|---|
| Print name : | Print name : |
| Signature : | Signature : |
| Date : | Date : |
| dd mmm yyyy | dd mmm yyyy |

| | |
|---|---|
| nidiag — Better Diagnosis of Neglected Infectious Diseases | **SOP titre:** Activités de Contrôle de la Qualité Interne |
| | **Projet/étude:** Cette SOP s'applique à tous les sites NIDIAG. |

## 1. Domaine d'application

Toutes les études NIDIAG sont conduites en accord avec la Charte d'éthique NIDIAG, les lois et règlements nationaux et internationaux, les Bonnes Pratiques Cliniques de Laboratoire (GCLP) de l'OMS ainsi que l'ICH et les recommandations des Bonnes Pratiques des Essais Cliniques (GCP). Le système de monitoring (GCP/GCLP) implémenté dans le projet NIDIAG inclus 2 composantes complémentaires : 1) une composante Contrôle de la Qualité Interne (CQI) et 2) une composante Contrôle de la Qualité Externe (CQE) incluant le monitoring du laboratoire. La composante CQI implique des Responsables de la Qualité (ou Quality Managers) qui veilleront à réaliser un contrôle de la qualité à intervalles réguliers aux sites d'études.
Comme la supervision GCP/GCLP requiert différents champs de compétences d'expertise, l'investigateur principal coordinateur du pays peut décider de déléguer les différentes tâches du QM à une ou deux personne(s) de son équipe, selon ses/leurs compétences et expertises propres.
Cette procédure décrit les responsabilités d'un QM au site d'étude et spécifie comment les activités qu'il est amené à faire s'articulent autour du travail de la composante monitoring externe (SOP-WP6-QUAL-02) et du monitoring du laboratoire.
Cette SOP ne n'applique pas à la RDC.

## 2. Responsabilités

| Function | Activities |
|---|---|
| Responsable Qualité (QM) du site | ▪ S'assurer que le personnel impliqué dans l'étude est informé sur tous les aspects GCP/GCLP et les respecte<br>▪ S'assurer que l'équipe est formée au protocole d'étude et aux SOPs NIDIAG pertinentes.<br>▪ Conduire un contrôle de la qualité interne à intervalles réguliers pour toutes les activités liées à l'étude.<br>▪ Documenter le contrôle de la qualité effectué en utilisant le Rapport du QM (Annexe1.1) et discuter les observations GCP/GCLP identifiées avec l'investigateur principal et l'équipe.<br>▪ Rapporter toute observation majeure au responsable du WP6 – et à l'investigateur du site – qui coordonnera la communication avec le Moniteur Externe et/ou le Moniteur du Laboratoire, selon les besoins.<br>▪ Soutenir l'Investigateur Principal (PI), le Moniteur Externe et le Superviseur du Laboratoire dans l'organisation et la conduite des visites de monitoring (SOP-WP6-QUAL-02).<br>▪ Répondre présent pendant les visites de monitoring.<br>▪ Assister le PI du site dans l'implémentation des actions correctives suite aux CQI et CQE. |
| External Monitor<br>Lab Monitor | ▪ Conseiller et soutenir le QM sur la manière d'effectuer les contrôles de la qualité entre les visites de monitoring externes.<br>▪ Revoir toute observation majeure rapportée par le QM et si |

| | |
|---|---|
| | nécessaire, discuter ces observations avec le responsable du WP6. |
| Investigateur Principal (PI) du site | ▪ S'assurer que le QM a accès à tous les documents de l'étude et aux autres installations/équipements.<br>▪ S'assurer que le QM a suffisamment de temps à consacrer aux activités demandées, sans que cela se fasse au détriment de ses autres tâches de routine.<br>▪ S'assurer que le QM reçoit toute information clé pertinente au statut de l'étude. Implémenter les actions correctives pour répondre aux observations relevées par le QM. |
| Investigateur Coordinateur du Pays | ▪ Assigner les tâches du QM à un ou deux membre(s) de son équipe. |
| Responsable du WP6 ou son/sa représentante | ▪ Etre le lien entre le "Trial Management Group » et le QM.<br>▪ Guider le QM dans la planification de ses activités de contrôle de la qualité à chaque site d'étude.<br>▪ Revoir les rapports du QM et envoyer une copie à l'investigateur coordinateur du pays, au représentant du WP4 et aux membres concernés du Trial Management Group.<br>▪ Rester en contact avec le QM et le PI régulièrement, les conseiller s'il y a des problèmes de qualité et/ou de fréquence du contrôle de la qualité. |

# 3. Procédures

## 3.1 Formation du personnel de l'étude

Tout personnel impliqué dans le projet NIDIAG doit recevoir une formation sur le protocole de l'étude, les SOPs qui sont pertinentes pour son travail, les Bonnes Pratiques des essais Cliniques (GCP) et les Bonnes Pratiques Cliniques de Laboratoire (GCLP). Le QM est en charge de former, avec l'aide du PI et des moniteurs externes/de laboratoire, le nouveau personnel qui n'aura pas eu l'occasion de suivre les workshops GCP/GCLP du projet NIDIAG avant que le recrutement de patients ne commence. Les modèles d'outils de formation sont disponibles sur le site NIDIAG (www.nidiag.org) ou peuvent être obtenus via le représentant du WP6.

Le QM et le PI sont chargés de former tout personnel nouvellement impliqué dans le projet NIDIAG suite à un remplacement ou s'ils estiment nécessaire de former à nouveau le personnel de l'étude.

## 3.2 Description des activités de Contrôle de la Qualité Interne

### 3.2.1. Vérifier les Formulaires de Consentement Eclairé (FCE)

Vérifier la compliance avec la SOP-WP6-DOC-01. En particulier, le QM devrait idéalement être présent lors de la discussion autour du consentement éclairé pour les 10 premiers patients inclus dans l'étude. Puis, il peut assister à cette discussion tous les 25 patients à condition que le patient

accepte. Il/Elle doit vérifier que le processus d'obtention du Consentement Eclairé obéit aux Principes de la Déclaration de Helsinki, OMS et aux recommandations Bonnes Pratiques des essais Cliniques (GCP).

Vérifier que **tous** les patients inclus dans l'étude ont donné leur consentement éclairé écrit :

- Vérifier que les noms, la date et l'heure de la signature sont appropriés.
- Vérifier si oui ou non un représentant légal/ un témoin indépendant était requis pour chacun des cas, selon les GCP et la SOP-WP6-DOC-01.
- Pour les sujets légalement incompétents, vérifier si l'assentiment était requis et si oui, s'il a été obtenu.
- Pour les patients inclus en situation d'urgence, vérifier que le consentement a été initialement obtenu d'un parent proche et plus tard réitéré par le (la) patient(e) lui (elle)-même.
- Vérifier que le consentement a été obtenu avant toute procédure liée à l'étude.
- Vérifier avec l'investigateur que chaque participant a bien reçu une copie signée de son FCE.
- Vérifier que tous les formulaires de consentement éclairés sont stockés de manière sécurisée dans le Dossier de l'Investigateur du Site (SIF).

Documenter la revue/vérification des FCE dans le rapport du QM.

### 3.2.2. Revue des Cahiers d'Observation (CRF) et des formulaires de collecte des données de laboratoire

Vérifier que la "Liste d'identification des patients" est complète, juste et cohérente avec l'enregistrement du centre (vérifier le nom du patient, ses initiales, sa date de naissance/son âge, le numéro d'enregistrement, la date d'admission).

Vérifier que les numéros des patients ont bien été attribués selon la SOP-WP6-DOC-02.

Ecrire le nombre de patients présélectionnés (ou « screenés ») et le nombre de patients inclus dans le rapport du QM.

Pour tous les patients, vérifier que le CRF est bien rempli selon la SOP-WP6-DATA-01 (SOP-WP6-DATA-03 pour le « Sydrome digestif »). S'assurer que les données saisies sont lisibles et qu'elles ont été entrées uniquement par le personnel autorisé à remplir les CRFs et les formulaires de collecte des données.

Confronter les données entrées dans le CRF du patient avec les documents sources, ex : le dossier médical du patient, les notes de laboratoire, les notes du médecin, le registre du centre d'étude et toute autre documentation source disponible sur chaque site.

En particulier, veiller à vérifier :

- Les résultats des tests de référence effectués sur le site doivent être consistants dans tout le CRF, les notes de laboratoire et tous autres formulaires de laboratoire.
- Les résultats des tests de référence effectués dans les laboratoires de référence doivent être consistants dans tout le CRF et dans les formulaires des laboratoires de référence.
- Les résultats des tests index doivent être consistants dans tout le CRF, les notes de laboratoire et toute autre documentation source disponible à chaque site (si applicable).
- Les médicaments prescrits sont consistants dans tout le CRF, le dossier médical du patient et les notes du médecin de l'étude.
- Le délai des examens cliniques, de la collecte des échantillons et des investigations du laboratoire sont consistantes dans tous les documents.

Concernant les résultats des tests index, la documentation source est le registre de laboratoire ou le carnet de laboratoire au Cambodge, au Népal et au Soudan.

En cas de divergence entre le CRF et les documents sources, les rapporter dans le rapport du QM.

Rappeler au PI que toute donnée manquante doit être complétée et que les entrées incorrectes lors de la saisie doivent être corrigées par l'investigateur responsable du site.

S'assurer que le format des corrections effectuées dans le CRF ou dans tout autre formulaire de collecte de données soit fait selon la SOP-WP6-DATA-01.

#### 3.2.3. Revue du Dossier de l'Investigateur du Site (SIF) / Dossier du Laboratoire (LF)

Le Dossier de l'Investigateur du Site (ou SIF) contient tous les documents essentiels de l'étude, ceux devant être collectés et classés par l'équipe pendant toute la durée du projet NIDIAG.

Le Dossier du Laboratoire (ou LF) contient tous les documents liés au laboratoire essentiels devant être collectés et classés par l'équipe pendant toute la durée du projet NIDIAG.

Aider l'investigateur principal et le responsable du laboratoire à vérifier que le SIF et le LF sont complétés et à jour selon la SOP-WP6-DOC-03.

Vérifier que l'investigateur du site envoie les copies des documents pertinents à l'investigateur coordinateur du pays.

#### 3.2.4. Echantillons Biologiques

Toutes les activités du laboratoire doivent être contrôlées par un QM avec des connaissances appropriées.

Certains échantillons collectés pendant l'étude sont traités et analysés directement sur le site. D'autres sont envoyés dans des laboratoires de référence (à l'intérieur du pays ou à l'étranger), alors que d'autres sont stockés en vue de futures recherches.

Vérifier que l'acheminement des échantillons marche correctement et selon la SOP traitant le sujet.

Vérifier que l'acheminement, le traitement, la gestion et le traitement des déchets contaminés suivent bien les principes de précaution.

Vérifier que le la « liste d'identification des échantillons de l'étude » (ou un document équivalent) est complet, correct et cohérent avec le registre du centre et le CRF (vérifier le numéro du patient, la date de collecte de l'échantillon, le type d'échantillon collecté, et si l'échantillon a été envoyé ou pas, etc…).

Vérifier que les échantillons sont correctement numérotés et étiquetés selon la SOP-WP6-DOC-02. Contrôler que les échantillons ne sont pas étiquetés avec les informations personnelles du patient (nom, adresse, numéro de téléphone).

S'assurer que les échantillons sont correctement stockés et en particulier que :

- L'accès est réservé au personnel autorisé.
- Les échantillons sont stockés séparément des échantillons collectés dans le cadre d'activités de routine.
- La température de stockage est adéquate et en accord avec les SOPs et est contrôlée régulièrement par le personnel responsable.

Vérifier que les analyses effectuées sur le site sont en accord avec le protocole et les SOPs NIDIAG applicables. Contrôler que :

- Les analyses sont effectuées par un personnel formé de manière adéquate.

- Les échantillons de moins bonne qualité (ex : volume collecté insuffisant, échantillon contaminé, etc…) sont rejetés à la réception.
- Les déviations par rapport aux SOPs sont documentées dans le(s) carnet(s) de laboratoire.
- Les analyses rapportant des résultats de test anormaux ou hors fourchette/ contrôles invalides sont répétés.
- Les résultats sont validés par le directeur du laboratoire avant d'être transmis au PI du site.
- L'aveugle est respecté pour les tests index.
- Les résultats sont transmis dans des délais raisonnables au PI du site.

Vérifier que l'envoi des échantillons aux laboratoires de référence (dans ou en dehors du pays) est adéquat: s'assurer que le délai d'envoi est en accord avec les SOPs NIDIAG applicables, que la température pendant le transport est adéquat et que toutes les mesures de précautions sont prises pour les échantillons dangereux. Egalement vérifier que la date et l'heure de l'envoi des échantillons, la durée de transport, la date et l'heure de réception des échantillons au laboratoire de référence est documenté dans la « Liste d'identification des échantillons ».

Les activités liées au laboratoire pour l'étude « Syndrome digestif » doivent être activement monitorées par le Responsable de la Qualité. Ce dernier devrait régulièrement relire 5-10% des échantillons traités. 10% de tous les échantillons de selles devraient être préservés dans une solution SAF pour permettre un contrôle de la qualité plus tard. Le QM pour le « Syndrome digestif » peut procéder à la vérification des éléments suivants :

- Pour les échantillons conservés dans le milieu SAF : Mini-FLOTAC, technique de concentration Formol-Ether (les échantillons conservés dans le milieu SAF peuvent être analysés plusieurs semaines après l'échantillonnage).
- Sur les lames colorées à l'acide : contrôle de la qualité en examinant 10% des échantillons (les lames colorées à l'acide peuvent être analysées plusieurs mois après l'échantillonnage).
- Si cela est faisable, prendre une photo de chaque résultat obtenu avec un TDR au moment où le résultat est lu et documenté (ce temps est TDR-spécifique, ex : après 15 min de réaction). Les photos seront à comparer par le QM avec les résultats enregistrés.
- Pour la plupart des autres tests de laboratoire (examen direct des selles, Kato-Katz, technique Baermann, cultures sur plaques de gélose Koga agar), aucune conservation n'est prévue mais les échantillons « frais » doivent être examinés par le Responsable de la Qualité le jour même.
- Les cultures bactériologiques issues des selles ne sont pas effectuées dans le laboratoire du site mais dans des institutions spécialisées (ex : Institut Pasteur en Côte d'Ivoire, l'Institut National de Recherche en Santé Publique au Mali). Le QM doit contrôler que le « process » des échantillons NIDIAG du « Syndrome digestif » suit les recommandations standards d'assurance qualité de l'institut.

### 3.2.5. Stockage et comptabilité des TDRs index

Vérifier que les tests index qui sont évalués dans les études NIDIAG sont stockés de manière appropriée. Veuillez en particulier vérifier que :

- L'accès aux TDRs est strictement réservé au personnel autorisé.
- La température de stockage est régulièrement contrôlée par le personnel responsable. Les kits TDRs ne doivent pas être utilisés s'ils sont endommagés.

Egalement contrôler que :

- Les TDRs sont utilisés avant leur date de péremption.

- Les TDRs sont utilisés uniquement pour les patients inclus dans l'étude
- Un système adéquat de comptabilité des TDRs est en place.

## 3.3 Fréquence des activités de contrôle qualité

Les activités de contrôle qualité doivent être effectuées régulièrement pendant toute la durée de l'étude, au moins tous les 25 patients inclus dans l'étude (voir Table 1).

Le contrôle de la qualité doit être effectué avant toute visite de monitoring de routine. L'investigateur principal doit s'assurer que le personnel de l'étude implémente toutes les actions correctives suggérées par le QM suite aux déviations/observations reportées, cela AVANT la prochaine visite de monitoring externe.

Les activités liées au contrôle de la qualité, lorsqu'elles sont faîtes régulièrement comme indiqué ci-dessous, représentent environ 3 jours de travail.

Table 1: Fréquences suggérées pour les différentes activités de Contrôle de la Qualité

| **Activités du Contrôle de la Qualité interne** | **Fréquence des activités du Contrôle de la Qualité** |
|---|---|
| Vérification des formulaires de consentements éclairés | Pour TOUS les patients, tous les 10 patients inclus |
| Vérification de la « Liste d'Identification des Patients » | Pour TOUS les patients, au moins tous les 25 patients inclus |
| Revue du CRF | Pour TOUS les patients, au moins tous les 25 patients inclus |
| Revue des dossiers de l'étude : SIF, IMF et LF | Au moins une fois par mois et à chaque fois qu'il y a une modification majeure (ex: changement de personnel, amendement du protocole d'étude, réception de nouveaux lots de TDRs) |
| Vérification des échantillons biologiques | Pour TOUS les patients, au moins tous les 25 patients |
| Vérification du stockage et de la comptabilité des tests index | Au moins une fois par mois et à chaque réception de nouveaux TDRs |

## 3.4 Rapports

A chaque fois que des activités de contrôle de la qualité sont effectuées, un rapport du QM doit être complété, partagé et discuté avec l'investigateur principal du site ainsi que le responsable du WP6 et/ou ses représentants.

Toute déviation critique ou majeure doit être rapportée sans délai (en plus de l'investigateur principal du site) au responsable du WP6 ou à ses représentants, qui le fera circuler aux personnes concernées du TMG, cela en vue d'aider à formuler les actions correctives.

Les QMs sont encouragés à regrouper leurs activités de contrôle de la qualité sur une période de 2-3 jours et à adapter la fréquence de ces contrôles en fonction du recrutement et des problèmes rencontrés. Dans la mesure du possible, le QM doit soumettre ses rapports complets au représentant du WP6, même si certaines activités ne se font pas à la même fréquence.

# 4. Définitions et Abréviations

## 4.1 Définitions

Laboratory File (LF): Le Dossier du Laboratoire comprend tous les documents essentiels liés au travail de laboratoire au niveau du site d'étude. Il y a un Dossier de Laboratoire par site d'étude. Le personnel du laboratoire est responsable de le maintenir à jour et de s'assurer qu'il est entreposé dans un endroit approprié.

Site Investigator File (SIF): Le Dossier de l'Investigateur du Site comprend tous les documents essentiels et les formulaires en relation avec la conduite de l'étude au niveau du site d'étude. Il y a un dossier de ce type par centre d'étude. Les investigateurs du site sont responsables de le maintenir à jour et de s'assurer qu'il est entreposé à un endroit approprié.

Country Investigator's Master File (IMF): Le Dossier de l'Investigateur du Pays comprend tous les documents essentiels liés à la conduite de l'étude au niveau du pays. Il y a un dossier de ce type par pays. L'investigateur coordinateur du pays est responsable de le maintenir à jour et de l'entreposer dans un endroit approprié.

Déviation critique: Conditions, pratiques ou processus qui porte atteinte aux droits, à la sécurité ou au bien-être des sujets ou à la qualité et à l'intégrité des données. Elles doivent être documentées et reflétées dans les rapports annuels ainsi dans que le rapport final de l'étude. Des actions correctives doivent immédiatement être implémentées.

Déviation majeure: Conditions, pratiques ou processus qui peuvent porter atteinte aux droits, à la sécurité ou au bien-être des sujets ou à la qualité et à l'intégrité des données. Elles doivent être documentées et, si nécessaire, reflétées dans les rapports annuels ainsi que dans le rapport de fin d'étude. Des actions correctives doivent être implémentées.

Assurance Qualité (AQ): Ensemble des activités préétablies et systématiques mises en œuvre pour s'assurer que la recherche est réalisée et que les données sont générées, recueillies par écrit, documentées, enregistrées et rapportées conformément aux bonnes pratiques cliniques et aux dispositions législatives et réglementaires en vigueur.

Contrôle Qualité (CQ): Techniques et activités à caractère opérationnels mises en œuvre dans le cadre du système d'assurance de la qualité, pour vérifier que les exigences de qualité applicables aux activités liées à la recherche sont satisfaites.

Documents sources: Documents originaux, données et enregistrements présentant un intérêt pour la recherche (dossiers médicaux, fichiers administratifs, comptes rendus de laboratoire, notes de service, registres de dispensation de la pharmacie, etc…).

## 4.2 Abréviations

CQ : Contrôle Qualité

CQE: Contrôle de Qualité Externe

CQI: Contrôle de Qualité Interne

CRF: Case Report Form (ou Cahier d'Observation)

FCE: Formulaire de Consentement Eclairé

GCLP: Good Clinical Laboratory Practice (ou Bonnes Pratiques Cliniques de Laboratoire)

GCP: Good Clinical Practice (ou Bonnes Pratiques Cliniques)

ICH : Conférences Internationales sur l'Harmonisation

IMF: Country Investigator Master File (ou Dossier de l'Investigateur Principal du Pays)

OMS : Organisation Mondiale de la Santé

PI: Investigateur Principal

LF: Laboratory File (ou Dossier du Laboratoire)

QM: Quality Manager (ou Responsable de la Qualité)

SIF: Dossier de l'Investigateur du Site

SOP: Procédures Opératoires Standardisées

TDR: Test de Diagnostic Rapide

TMG : Trial Management Group

WP6 : Work Package 6

## 5. Registres et Archives

| **Annexes & Formulaires à compléter** | |
|---|---|
| **Numéro** | **Titre** |
| SOP-WP6-QUAL03-Annexe1 | Modèle de Rapport du Responsable de la Qualité |

| **Révision** | |
|---|---|
| Version 1.1 (initiale) | Traduction en français par Ninon Horié (SOP de référence: SOP-WP6-QUAL-03-V3-17Mar2014). |

## 6. Historique du Document

| **Nom et Fonction** | **Date** | **Signature** |
|---|---|---|
| *Auteur* | | |
| Emilie Alirol | 01 February 2013 | 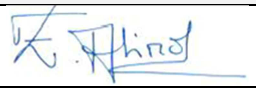 |
| *Revu par* | | |
| Ninon Horié | 14th July 2014 | 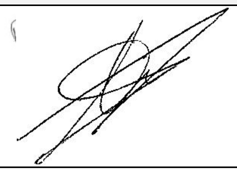 |
| *Approuvé par* | | |
| Raffaella Ravinetto | 30th July 2014 | 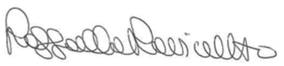 |

**Annexe 1 : Modèle de Rapport du Responsable de la Qualité**

| RAPPORT DU RESPONSABLE DE LA QUALITE N° | | |
|---|---|---|
| Syndrome: | | |
| Protocole d'étude n°: | | |
| Site d'étude : | | |
| Pays : | | |
| PI du Site: | | |
| Investigateur Coordinateur du Pays: | | |
| Responsable de la Qualité : | | |
| Moniteur Externe : | | |
| Date du Rapport : | (jj/mm/aaaa) | |
| Nombre de patients (au moment du rapport) : | Présélectionnés: | Inclus: |
| Patients contrôlés cette fois : | Du patient n° | Au patient n° |
| Dans le cas où il y a 2 Responsables de la qualité à ce site, merci de mentionner comment les tâches sont réparties: | | |
| | | |

| **CONSENTEMENT ECLAIRE** | |
|---|---|
| Le processus de consentement éclairé est en accord avec la SOP-WP6-DOC-01 | ☐oui ☐non ☐NE |
| Tous les patients inclus dans l'étude ont donné leur consentement écrit | ☐oui ☐non ☐NE |
| Le consentement a été obtenu avant toute procédure liée à l'étude | ☐oui ☐non ☐NE |
| Le PI du site (ou son délégué) a signé et daté le formulaire de consentement | ☐oui ☐non ☐NE |
| Lorsque cela est approprié, le consentement a été obtenu du représentant légal et/ou d'un témoin indépendant | ☐oui ☐non ☐NE |
| Lorsque cela est approprié, l'assentiment de l'enfant a été obtenu | ☐oui ☐non ☐NE |
| Pour les patients admis en urgence, le consentement initial du proche parent a été obtenu puis dans un second temps celui du patient lui-même | ☐oui ☐non ☐NE |
| Tous les patients inclus dans l'étude ont reçu une copie de leur FCE | ☐oui ☐non ☐NE |

Les FCE sont conservés de manière sécurisée dans le Dossier de l'Investigateur du Site ☐oui ☐non ☐NE

Si des déviations ont été observées dans le processus d'obtention du consentement éclairé, veuillez les lister ci-dessous:

Patient(s) n° Déviation:

Patient(s) n° Déviation:

Patient(s) n° Déviation:

Patient(s) n° Déviation:

Patient(s) n° Déviation:

Commentaires supplémentaires et actions correctives proposées (incluant la formation du personnel d'étude si approprié):

**CAHIERS D'OBSERVATION (CRFS) ET OUTILS DE COLLECTE DES DONNEES DE LABORATOIRE**

Les CRFs sont completés selon la SOP-WP6-DATA-01 ☐oui ☐non ☐NE

Les CRFs et les outils de collecte des données de laboratoire sont lisibles, corrects, lisibles et complets ☐oui ☐non ☐NE

Les CRFs et les outils de collecte des données de laboratoire sont complétés uniquement par le personnel autorisé ☐oui ☐non ☐NE

Les CRFs et les outils de collecte des données de laboratoire sont consistants avec les documents sources ☐oui ☐non ☐NE

Les corrections du CRF et des outils de collecte des données de laboratoire sont faits selon la SOP-WP6-DATA-01 ☐oui ☐non ☐NE

Les CRFs et les outils de collecte de données de laboratoire sont stockés dans un endroit approprié ☐oui ☐non ☐NE

Si vous notez des déviations dans le processus de collecte des données, veuillez les lister ci-dessous :

Patient(s) n° Déviation:

Patient(s) n° Déviation:

Patient(s) n° Déviation:

Patient(s) n° Déviation:

Patient(s) n° Déviation:

Commentaires supplémentaires et actions correctives proposées (incluant la formation du personnel d'étude si approprié):

| ECHANTILLONS BIOLOGIQUES | |
|---|---|
| La collecte, l’acheminement , la gestion et l’envoi des échantillons biologiques se fait en accord avec les SOPs NIDIAG applicables | ☐oui ☐non ☐NE |
| La liste des échantillons collectés est complète et à jour | ☐oui ☐non ☐NE |
| Les échantillons sont étiquetés avec un numéro unique déterminé selon la SOP-WP6-DOC-02 | ☐oui ☐non ☐NE |
| Le processus respecte la confidentialité du patient | ☐oui ☐non ☐NE |
| Les échantillons de l’étude sont stockés de manière sécurisée (endroit fermé à clé et accès réduit au personnel) | ☐oui ☐non ☐NE |
| La température de stockage est adéquate et contrôlée régulièrement | ☐oui ☐non ☐NE |
| Toutes les analyses sont effectuées en accord avec les SOPs NIDIAG applicables | ☐oui ☐non ☐NE |
| Les resultats des analyses sont enregistrés et transmis au PI du site dans des délais raisonnables | ☐oui ☐non ☐NE |
| Les analyses aboutissant à des resultats de tests anormaux ou des contrôles hors fourchette sont documentées et répétées | ☐oui ☐non ☐NE |
| Les échantillons dangereux sont manipulés avec précaution et jetés de manière appropriée | ☐oui ☐non ☐NE |
| Les échantillons de l’étude sont envoyés selon les SOPs NIDIAG applicables et selon les réglementations internes et nationales | ☐oui ☐non ☐NE |

Si des déviations dans les processus de gestion des échantillon sont détectées (tube manquant, hémolyse, volume d’échantillon inapproprié, décongélation), merci de les lister ci-dessous:

| | |
|---|---|
| Patient(s) n° | Déviation: |
| Patient(s) n° | Déviation: |
| Patient(s) n° | Déviation: |
| Patient(s) n° | Déviation: |
| Patient(s) n° | Déviation: |

Commentaires supplémentaires et actions correctives proposées (incluant la formation du personnel de l’étude si approprié):

| TESTS DIAGNOSTIC RAPIDES | |
|---|---|
| Les TDRs sont stockés de manière sécurisée (accès restreint + fermable à clé) | ☐oui ☐non ☐NE |
| La temperature de stockage suit les recommandations du fabricant | ☐oui ☐non ☐NE |
| Les TDRs sont utilisés uniquement pour les patients inclus dans | ☐oui ☐non ☐NE |

| | |
|---|---|
| l'étude NIDIAG | |
| Les TDRs sont utilisés (et seront utilisés) avant la date d'expiration | ☐oui ☐non ☐NE |
| Le système de comptabilité des TDRs est correct et à jour | ☐oui ☐non ☐NE |
| Commentaires supplémentaires et actions correctives proposées (incluant la formation du personnel de l'étude si approprié): | |
| **Autre information pertinente:** | |
| | |

Rapport écrit par le(s) Responsable(s) de la Qualité:

Nom(s):

Signature et date:

Rapport approuvé par le PI du site:

Nom:

Signature(s) et date:

Rapport envoyé au représentant du WP6 (………….. …………………….): le ……/……/..…….. [DATE]

| | |
|---|---|
| nidiag<br>Better Diagnosis of Neglected Infectious Diseases | **SOP titre:** Visites de Supervision du Laboratoire (GCLP) |
| | **Projet/étude:** NIDIAG: Cette SOP s'applique à toutes les études cliniques NIDIAG (WP2). |

## 1. Domaine d'application

Toutes les études NIDIAG doivent être menées dans le respect de la charte éthique NIDIAG, les réglementations nationales et internationales applicables, les Bonnes Pratiques Cliniques de Laboratoire (GCLP) de l'OMS, l'ICH et les Bonnes Pratiques Cliniques (GCP) ainsi que les directives de l'OMS sur les GCP. Le système de surveillance des GCP/GCLP à mettre en œuvre tout au long de l'étude NIDIAG comporte 2 composantes complémentaires: 1) Une composante de Contrôle de la Qualité Interne (CQI) et 2) une composante de Contrôle de la Qualité Externe (CQE).
Cette procédure décrit comment les visites de supervision de laboratoire doivent être effectuées, documentées et suivies dans le respect des principes des GCLP.

## 2. Responsabilités

| Fonction | Activités |
|---|---|
| **Superviseur du laboratoire** | ▪ Vérifie que l'essai clinique se déroule en conformité avec les exigences des GCLP, le protocole de l'étude et les amendements, et les SOPs du projet NIDIAG.<br>▪ Rapporte toute déviation dans le Rapport de Visite de Laboratoire.<br>▪ Envoie le rapport de visite de laboratoire au moins au Responsable du WP2, l'investigateur coordinateur du pays, l'investigateur principal du site et le coordinateur du WP6*.<br>▪ Aborde les questions spécifiques entre deux visites. |
| **Moniteur Externe** | ▪ Rapporte toute déviation écart concernant le laboratoire dans le rapport de monitoring. |
| **Responsable de la Qualité (QM) ou fonction équivalente** | ▪ Soutient le moniteur de laboratoire dans l'accomplissement de ses tâches.<br>▪ Assiste aux visites de suivi de laboratoire. |
| **Investigateur principal (PI) du site** | ▪ Soutien le moniteur de laboratoire dans l'accomplissement de ses tâches.<br>▪ Assiste aux visites de suivi de laboratoire et s'assure que tout le personnel-clé de l'étude est présent.<br>▪ S'assure que le moniteur de laboratoire a un accès total aux documents et aux installations de l'étude.<br>▪ Veille à ce que les mesures correctives énumérées dans le rapport de visite de laboratoire et/ou dans la lettre de suivi sont implémentées. |
| **Investigateur coordonateur du pays** | ▪ S'assure que le moniteur de laboratoire reçoit toute nouvelle information clé correspondant au statut et à la conduite de l'étude.<br>▪ Vérifie que les actions correctives figurant dans le rapport de visite de laboratoire et/ou dans la lettre de suivi sont implémentées. |
| **Coordinateur de suivi du WP6*** | ▪ S'assure que le moniteur de laboratoire a reçu les rapports du moniteur externe. |

* La coordination du monitoring WP6 est assurée par **Raffaella Ravinetto** pour le syndrome neurologique, **Ninon Horié** pour le syndrome de fièvre et **Peiling Yap** pour syndrome digestif.

## 3. Visites de supervision de laboratoire à effectuer

### a. Planification de la visite de supervision de laboratoire

Le coordonnateur du WP6, en consultation avec l'investigateur coordonnateur pays et le responsable du WP2, détermineront le calendrier approprié pour les visites de suivi de laboratoire pour chaque étude clinique. Les visites de supervision de laboratoire ont lieu sur le site même et dans les laboratoires de référence et l'administration centrale, s'il y a lieu.

Avant la visite, le responsable du laboratoire doit:

- Revoir le protocole de l'étude, les CRFs, les SOPs de l'étude et les rapports du superviseur moniteur externe/laboratoire précédents, ainsi que d'autres documents clés fournis par l'investigateur coordonnateur du pays ou par le responsable du WP2.
- Informer le l'investigateur coordonnateur du pays et le PI du site au moins 1 mois avant la visite de supervision de laboratoire, et leur demander de veiller à ce que toutes les personnes clés de l'étude soient présentes lors de la visite.
- Envoyer un programme provisoire de visite à l'investigateur coordonnateur pays.

### b. Visites de supervision de laboratoire à effectuer

#### i. Visite d'évaluation du laboratoire

L'évaluation du laboratoire est effectuée avant le début du recrutement. Elle est effectuée par le responsable du WP2 ou une personne déléguée qualifiée, conjointement avec l'investigateur coordonnateur pays et, idéalement avec le superviseur de laboratoire (si déjà identifié) ou un délégué avec des connaissances de routine sur les procédures de laboratoire et les aspects GCLP. Un rapport avec les résultats et les conclusions des évaluations est préparé et distribué parmi l'équipe de coordination de l'étude.

Lors de la visite d'évaluation du laboratoire, les aspects suivants sont vérifiés (les listes de contrôle peuvent être utilisées comme appui):

- Les conditions générales de laboratoire (les installations, la source d'électricité, quel type de tests de laboratoire est effectué, le type et la qualité des réactifs et du matériel, le système de stockage, l'enregistrement de laboratoire, etc.)
- La disponibilité et la connaissance du personnel de laboratoire (nombre suffisant/insuffisant de personnes impliquées au laboratoire, personnel de laboratoire compétent ou pas, personne(s) remplaçante(s) disponible(s) ou pas)
- Le système de gestion de qualité du laboratoire (contrôles de qualité disponibles, participation au contrôle qualité externe, documentation, etc.,)

Les décisions et mesures suivantes doivent être prises avant la visite d'initiation de l'étude :

- Décider quels tests (index) doivent être effectués sur le site et lesquels doivent être effectués dans les laboratoires de référence. S'assurer que tous les tests (index) sont validés avant leur utilisation.
- Elaborer un plan analytique ou alternativement, s'assurer que ce type d'information est disponible par d'autres moyens (SOPs, dossier de laboratoire), ex : le diagramme d'échantillons et des résultats ; la SOP sur la manipulation, le stockage et la gestion des

échantillons de l'étude; la documentation sur les valeurs normales, la SOP sur la gestion des données ...

- S'assurer qu'un CRF de bonne qualité est développé en collaboration avec le « Data Manager » et les statisticiens du WP6.
- S'assurer que tous les équipements nécessaires, les tests et réactifs pour les tests de laboratoire à effectuer sont disponibles.
- S'assurer que les SOPs sont disponibles pour toutes les procédures de laboratoire et des activités associées (par exemple, l'assurance de la qualité, la gestion des données). Si nécessaire, préparer des SOPs spécifiques au site.
- S'assurer qu'un système de commande efficace est mis en place, ceci permettant une livraison de routine régulière et à temps des kits et réactifs d'essai (soit localement ou de manière centralisée au niveau du site coordonnateur de l'étude).

→ Si l'une des conditions ci-dessus n'est pas remplie, l'étude NE DOIT PAS être initiée.

### ii. Visite d'initiation du site d'étude

La visite d'initiation de l'étude est effectuée après réception de tout le matériel de l'étude, après l'approbation du Comité d'Ethique, et avant le début du recrutement. Idéalement la visite est effectuée en collaboration avec le moniteur externe et/ou le responsable du WP2. Au minimum, les activités suivantes doivent être réalisées

- *Formation au Bonnes Pratiques Cliniques de Laboratoire (GCLP):*

  En discussion avec le coordinateur du WP6 responsable du monitoring et le moniteur du site, une formation formelle aux GCLP est donnée à l'ensemble du personnel n'ayant pas participé à l'atelier NIDIAG sur les GCP/GCLP, soit par le moniteur externe et/ou le superviseur du laboratoire en collaboration avec le QM, avant le début du recrutement.

- *Revue du Protocole* : les aspects de laboratoire du protocole sont examinées avec tout le personnel de l'étude, le plan analytique et les documents associés sont discutés (par exemple le diagramme d'échantillon et résultats)

- *Formation au Laboratoire* : Si nécessaire, une formation est donnée sur toutes les SOPs se rapportant aux activités de laboratoire (procédures de laboratoire, prise en charge/stockage/gestion des échantillons d'étude, données sources et gestion des données, système de stockage, assurance qualité, etc.)

- *Formation au Remplissage du CRF* : une formation sur le remplissage de la partie laboratoire du formulaire de report de cas (CRF) est donnée.

- *Dossier de Laboratoire* : le dossier de laboratoire est vérifié pour exhaustivité (réf. SOP WP6-DOC-03 sur la Gestion des documents de l'étude).

- *Installation du Laboratoire* : le superviseur du laboratoire doit confirmer que le laboratoire est adéquat pour la conduite des activités de l'étude (espace de stockage pour le matériel de laboratoire et les documents sources, relevé des températures).

- *Pilotage* : si tous les résultats sont satisfaisants, une étude de pilotage (avec 10-20 patients) peut être effectuée à la fin de la visite d'initiation, sous la responsabilité du superviseur de laboratoire et/ou du moniteur externe/ du responsable du WP2. Au cours de cette étude de pilotage, toutes les activités sont effectuées comme si de vrais patients étaient recrutés. Les actions correctives sont alors appliquées en cas de besoin.

- *Début du recrutement* : si la phase pilote est satisfaisante, le recrutement peut commencer.

Des rapports de supervision de laboratoire de la visite d'initiation et de la phase de pilotage sont préparés, documentés et envoyés au responsable du WP2/délégué, à l'investigateur coordonnateur du pays/délégué, et à l'investigateur principal du site pour le suivi des déviations et les actions correctives.

*Exceptions*: le responsable du WP2 peut décider de sauter la visite d'initiation de l'étude, si tous les points ont été abordés et documentés sur les visites précédentes, et que des mesures correctives ont été implémentées.

### iii. Visites de supervision de routine du laboratoire

Les visites de supervision de routine du laboratoire ont lieu comme prévu avec le coordinateur du WP6 responsable du monitoring et l'investigateur coordonnateur du pays (idéalement tous les 3 mois au début de l'étude, tous les 6-8 mois à un stade plus avancé de l'étude). Ces visites peuvent coïncider avec les visites du moniteur GCP et/ou du responsable du WP2.

Au minimum, les aspects suivants doivent être vérifiés:

- *Suivi de la visite précédente* : suivi de toutes les activités en attentes énumérées lors des visites précédentes.
- *Installations du laboratoire* : vérifier s'il y a eu des problèmes concernant les installations du laboratoire au cours de l'étude. Ceux-ci doivent être reportés dans un registre (par exemple : problèmes avec l'électricité).
- *Gestion des stocks et commande des produits de laboratoire :*
    - o Vérifier que les produits de laboratoire sont stockés dans un environnement approprié (au sec, au frais, à l'abri des inondations/rongeurs, à la bonne température de stockage – air conditionné, réfrigérateur, etc.)
    - o Vérifier qu'un relevé de température adéquat est en place dans la pièce de stockage, les réfrigérateurs, les congélateurs, les incubateurs et le laboratoire. (voir SOP-WP6-QUAL-06 « Comment installer et utiliser un thermomètre "min/max" »). Les formulaires de contrôle de la température doivent être classés dans le Dossier de Laboratoire (LF).
    - o Vérifier qu'un bon système de gestion des stocks est en place pour les produits de laboratoire utilisés pour l'étude NIDIAG: fiches de stocks et l'inventaire mensuel de stocks (voir SOP-WP6-QUAL-07-V01-24Sep2012 « Gestion des stocks »). Les inventaires mensuels de stocks doivent être classés dans le Dossier de Laboratoire (LF).
    - o Vérifier que le principe « premier entré, premier sorti » est respecté: utiliser en priorité les produits qui expireront en premier.
    - o Vérifier qu'il existe un récapitulatif de ce qui a été commandé (l'information est disponible selon l'endroit où la commande a eu lieu: soit au niveau de la coordination du site d'étude ou au niveau du pays/site d'étude).
        - ▪ Quels éléments ont été commandés, leur quantité, à quel moment la commande a-t-elle été effectuée ?
        - ▪ Numéro de lot et date de péremption des articles commandés.
        - ▪ Si livraison d'articles de laboratoire : date de l'envoi, accusé de réception, relevé de température (contrôle de l'humidité si nécessaire) durant l'envoi d'articles via une sonde de température.

- si les tests index sont commandés au niveau central (au niveau de la coordination de l'étude):
  - Vérifier la disponibilité des enregistrements de suivi (voir point précédent).
  - Vérifier qu'un kit par lot de tests index de la liste est conservé au niveau de la coordination de l'étude.
  - Vérifiez que les instructions d'utilisation (IFU) sont appliquées :
    - Numéro de version de l'IFU et la date d'émission,
    - Pour chaque nouvelle version de l'IFU, adapter la SOP si nécessaire.

- *Echantillons biologiques:*
  - Vérifier que tous les spécimens biologiques sont traités selon les SOPs applicables et en vigueur (par exemple : La biosécurité). vérifier si l'échantillonnage se déroule convenablement.
  - Vérifier que tous les spécimens biologiques sont correctement étiquetés (voir SOP-WP6-DOC-02-V3.1-18Jul2014_DIG « Système de numérotation » pour syndrome digestif).
  - Vérifiez que le volume d'échantillons requis est recueilli comme décrit dans les SOPs applicables (par exemple : SOP-WP2-LAB-44 « Prélèvement de sang »), et que cette information est correctement reportée (dans les CRF / formulaires spécifiques d'échantillonnage).
  - Vérifiez que le volume d'échantillons restant est collecté dans des récipients appropriés (par exemple : cryo-flacons de 2 ml) selon les SOPs applicables et que les échantillons stockés sont correctement enregistrés dans le « Log des spécimens de l'étude » se trouvant dans le Dossier de Laboratoire.
  - Vérifier que le « Log des spécimens de l'étude » ou document(s) équivalent(s) est(sont) complet(s), juste(s) et cohérent(s) avec le registre du centre et le CRF (vérifier le numéro du patient, la date de prélèvement de l'échantillon, le type d'échantillon prélevé, si l'échantillon a été expédié ou pas, etc.).

  - *Conservation des échantillons biologiques :*
    - Vérifier que tous les échantillons biologiques sont correctement conservés jusqu'à ce qu'ils soient acheminés ou jusqu'à la fin de l'étude (par exemple -70°C pour les restes d'échantillons de patients, incubateur pour les cultures de selles/sang).
    - Vérifier qu'un inventaire du contenu du congélateur est disponible : pour le congélateur à -70°C, avec des relevés quotidiens de température (Dossier de Laboratoire).
    - Vérifier que les isolats bactériens sont correctement conservés (voir SOP-WP6-LAB-01) jusqu'à leur acheminement ou jusqu'à la fin de l'étude.

  - *Transport des échantillons biologiques:*
    - Vérifier que la liste de colisage (« Packing List ») est remplie pour chaque transport et une copie de la liste de colisage est conservée dans le Dossier de Laboratoire.

- Vérifier que le transport des échantillons biologiques dans le pays (par exemple, transport à partir du site d'étude vers un labo de référence) ou entre pays (exemple : du site d'étude à ITM Anvers) est fait selon les SOP applicables: triple emballage (biosécurité), température correcte (par exemple, les packs de glace, sonde de température).
- Vérifier que la date et l'heure d'envoi des échantillons, la durée du transport, la date et l'heure de réception des échantillons au laboratoire de référence sont documentés dans le « Log des spécimens de l'étude ».

- *Tests de laboratoire :*
  - Vérifier que les formulaires de demande de laboratoire sont en place (quels échantillons doivent être prélevés, quels tests à faire, etc.) et sont conformes au protocole d'étude et aux SOPs en vigueur.
  - Vérifier que le matériel et réactifs sont correctement utilisés pour les tests de laboratoire. Aucun des produits utilisés ne doit être expiré.
  - Vérifier que les valeurs normales spécifiques à tous les tests pertinents de laboratoire du pays sont disponibles dans le Dossier de Laboratoire et le Dossier de l'investigateur du site (SIF).
  - Vérifier que les tests sont interprétés selon les SOPs applicables.
  - Vérifier que les résultats des tests sont correctement enregistrés dans des registres de laboratoire et/ou le CRF.
  - Vérifier que toutes les données sources (résultats des tests) sont correctement stockés dans une armoire fermée à clé et peuvent être sorties pour la vérification des données sources par le moniteur externe.
    Vérifier que les résultats sont rapportés au PI de manière adéquate et à temps.

- *Tests Index :*
  - Vérifier que les tests index sont correctement faits et conservés (voir SOP-WP6-QUAL-05 « Manipulation et stockage des TDR »)
  - Vérifier qu'aucun des tests d'index utilisés n'est expiré.
  - Vérifier que les tests index ne sont utilisés que pour les patients qui sont recrutés dans l'étude.
  - Vérifier que la liste des tests index est correctement remplie.
  - Vérifier que les tests index sont effectués conformément aux SOPs applicables (volume correct d'échantillon, tampon correct utilisé, nombre de gouttes de tampon utilisé correct, lecture du résultat au bon temps).
  - Vérifier que les micropipettes sont utilisées à la place des dispositifs de transfert de sang inclus dans les kits de TDR.
  - Vérifier que les procédures de répétition de test et la documentation des résultats (changement de couleur du dessicant, présence de la ligne de contrôle, présence de bruit de fond, intensité des lignes test) sont correctement effectuées.
  - Vérifier que les résultats sont correctement interprétés selon les SOPs en vigueur.
  - Vérifier que les photos des tests index sont prises, et que ces données sont soigneusement enregistrées sur un ordinateur (avec des sauvegardes régulières).

- Vérifier que toutes les sources de données (résultats des tests index) et les CRFs sont correctement conservés (par exemple séparément du reste du CRF) et qu'elles peuvent être sorties pour la vérification des données par le moniteur externe.

- *Contrôle de la qualité* :
  - Vérifier que les contrôles de la qualité internes sont réalisés conformément aux SOPs applicables :
    - Contrôles internes quotidiens/hebdomadaires pour la biochimie, l'hématologie, la sérologie, etc.
    - Souches ATCC et paramètres de qualité pour la bactériologie (% contamination, % organismes cliniquement significatifs, volume de sang prélevé pour la culture en bouteille).
    - Deuxième lecture des tests spécifiques (voir SOPs spécifiques)
  - Effectuer une relecture des lames positives au microscope (tels que les lames de paludisme, la coloration de Gram) et un pourcentage de lames négatives au microscope (maintenir l'aveugle des résultats originaux).
  - Consulter le rapport de l'évaluation externe de la qualité si le laboratoire participe à un programme d'EQA (par exemple, EQA pour le diagnostic des bactéries pour les sites NIDIAG dans le premier trimestre de 2014).
  - Vérifier le rapport d'audit de la qualité si cela est effectué (en accord avec TMG).
  - Vérifier que tous les dossiers de contrôles de qualité sont classés dans le Dossier de Laboratoire

- *SOPs :*
  - Vérifier que tous les tests et tous les équipements possèdent une SOP à jour.
  - Vérifier que toutes les versions des SOPs sont classées dans le Dossier de Laboratoire.
  - Vérifier que seules les dernières versions des SOPs sont utilisées au laboratoire.
  - Vérifier que les SOPs sont examinées et révisées régulièrement.

- *CRF :*
  - S'il y a lieu, vérifier si le technicien de laboratoire qui remplit le CRF est formé aux bonnes pratiques de documentation (GDP).
  - Vérifier que la partie laboratoire des CRFs est dûment remplie selon les GDP.
  - Vérifier que les CRFs de laboratoire sont conservés en toute sécurité jusqu'à ce qu'ils soient renvoyés au PI.
  - Pour l'étude fièvre: vérifier que la partie des tests index du CRF est conservée séparément du reste du CRF et qu'elle a été envoyée directement à la personne saisissant les données, sans passer par le PI (maintenu en aveugle par rapport aux résultats des tests index).
  - La vérification des données sources des CRFs est effectuée par le moniteur externe.

- *Equipement :*
  - Inventaire de l'équipement
    - Vérifier que l'inventaire de l'équipement est en place dans le laboratoire
  - Plan de maintenance

    - Vérifier que le plan de maintenance est en place pour l'équipement ou si dans les SOP de l'équipement, il est mentionné comment le matériel doit être maintenu et à quelle moment cette maintenance devrait se faire.
  - Entretien et réparation
    - Vérifier qu'une liste de bord est en place pour indiquer si un équipement est hors service, ainsi que les mesures prises et les résultats.
    - Vérifier que si un équipement est en panne, cela est clairement indiqué sur la machine
  - Etalonnage
    - S'il y a lieu, vérifier si l'étalonnage est effectué en cas de besoin, et si un enregistrement de ces étalonnages est noté dans le Dossier de Laboratoire.

- *Contrôle de l'infection et Gestion des déchets :*
  - Vérifier que le laboratoire est propre, si les bancs sont désinfectés tous les jours, si les protections personnelles requises utilisés (blouses de laboratoire, gants, masques, etc.)
  - Vérifier qu'une procédure spécifique à la gestion des déchets du site est en place et est correctement appliquée.
  - Vérifier si la procédure pour la gestion des produits périmés (SOP en préparation pour NIDIAG) est correctement appliquée.

- *Dossier de Laboratoire (LF):*
  - Examiner le Dossier de Laboratoire et vérifier s'il est complet (voir SOP-WP6-DOC-03 « Gestion des documents de l'étude »): toutes les SOPs, tous les formulaires de contrôle de la qualité, toutes les listes de colisage remplies, les « Logs des spécimens de l'étude », les informations d'envoi des listes de tests index, les valeurs normales, etc.

Un rapport de supervision de la visite du laboratoire est préparé, documenté et envoyé au coordinateur du WP6, au responsable du WP2/délégué, à l'investigateur coordonnateur du pays/délégué, et au PI du site pour le suivi des dévations et actions correctives.

# 4. Définitions et Abréviations

## 4.1 Définitions

Dossier de Laboratoire (LF): le dossier de laboratoire comprend tous les documents essentiels relatifs aux travaux de laboratoire au niveau du site. Il y a un dossier de laboratoire par site d'étude. Le personnel du laboratoire est responsable de sa mise à jour et doit s'assurer qu'il est conservé de manière adéquate.

Dossier de l'Investigateur Principal du site (SIF): le dossier de l'investigateur principal du site comprend tous les documents essentiels et les formulaires relatifs à la conduite de l'étude au niveau du site. Il y a un dossier de ce type par site d'étude. Les investigateurs du site sont responsables de sa mise à jour et doivent s'assurer qu'il est conservé de manière adéquate.

Assurance Qualité (AQ): Ensemble des activités préétablies et systématiques mises en oeuvre pour s'assurer que la recherche est réalisée et que les données sont générées, recueillies par écrit,

documentées, enregistrées et rapportées conformément aux bonnes pratiques cliniques et aux dispositions législatives et réglementaires en vigueur.

Contrôle Qualité (CQ): Techniques et activités à caractère opérationnels mises en oeuvre dans le cadre du système d'assurance de la qualité, pour vérifier que les exigences de qualité applicables aux activités liées à la recherche sont satisfaites.

Documents Sources: Documents originaux, données et enregistrements présentant un intérêt pour la recherche (dossiers médicaux, fichiers administratifs, comptes rendus de laboratoire, notes de service, registres de dispensation de la pharmacie, etc…).

### 4.2 Abréviations

AQ : Assurance Qualité

CQE : Contrôle de la Qualité Externe

CQI : Contrôle de la Qualité Interne

CRF : Case Report Form (Formulaire de Rapport de Cas)

EQA : External Quality Assessment (Evaluation Externe de la Qualité)

GCP : Good Clinical Practices (Bonnes Pratiques Cliniques)

GCLP : Good Clinical Laboratory Practices (Bonnes Pratiques Clinique de Laboratoire)

GDP : Good Documenting Practices (Bonnes Pratiques de Documentation)

IFU : Instruction For Use (Instructions d'Usage)

LF : Laboratory File (Dossier de Laboratoire)

PI : Principal Investigator (Investigateur Principal)

QM : Quality Manager (Responsable de la Qualité)

SIF : Site Investigator File (Dossier de l'Investigateur principal du Site)

SOP : Standard Operating Procedure (Procédure Opératoire Standard)

TDR : Test de Diagnostic Rapide

TMG : Trial Management Group

WP : Work Package

## 5. Registres et archives

| **Annexes et formulaires à compléter** | |
|---|---|
| **Numéro** | **Titre** |
| NA | NA |

## 6. Historique du document

| **Révision** | |
|---|---|
| *SOP-WP6-QUAL-04-v1-23Feb2014* | *Version initiale en anglais* |
| *SOP-WP6-QUAL-04-v1.1-23Feb2014* | *Traduction de la version initiale en français* |

| **Nom et fonctions** | **Date** | **Signature** |
|---|---|---|
| *Auteur* | | |
| Barbara Barbé | 23/02/2014 | |
| *Revisé par* | | |
| Jean-T. Coulibaly | 30/07/2014 | |
| *Approuvé par* | | |
| Ninon Horié | 04/08/2014 | |

| | |
|---|---|
| nidiag Better Diagnosis of Neglected Infectious Diseases | **SOP titre :** Manipulation et conservation des tests rapides |
| | **Projet/étude:** NIDIAG: Cette SOP s'applique à toutes les études cliniques NIDIAG (WP2). |

# 1. Champ d'application

Cette SOP décrit la façon dont les tests rapides doivent être manipulés, conservés et inventoriés. Elle se concentre sur les tests index évalués dans les études neurologique et fièvre du projet NIDIAG mais s'applique également aux autres tests rapides.

# 2. Responsabilités

| Fonction | Responsabilités |
|---|---|
| Technicien de laboratoire | ▪ Respect de cette SOP<br>▪ Conservation des tests rapides aux conditions adéquates<br>▪ Gestion des stocks de tests rapides<br>▪ Maintien à jour de l'inventaire des stocks<br>▪ Vérification et documentation de la température de stockage |
| Responsable du laboratoire | ▪ Supervision des activités mentionnées ci-dessus |

# 3. Procédures

## 3.1 Matériel

- Formulaires mentionnés sous "4. Documents et archives"
- Thermomètre Min-Max
- Armoire fermant à clé
- Récipient « bio-hazard »

## 3.2 Procédure

### 3.2.1 Manipulation des tests rapides

#### 3.2.1.1 Précautions générales

1. Porter des gants.
2. Manipuler chaque échantillon biologique comme s'il était potentiellement infectieux.
3. Laisser les réactifs atteindre la température ambiante avant l'emploi.
4. Ne jamais utiliser un test après sa date d'expiration.
5. Ne jamais utiliser un test si le test est endommagé ou si l'emballage est ouvert.
6. Garder l'emballage fermé jusqu'à l'utilisation.
7. Utiliser le test immédiatement après ouverture de l'emballage.
8. Vérifier la couleur du dessicateur présent dans l'emballage. Si la couleur a changé (le dessiccateur est saturé) jeter le test et en utiliser un nouveau.
9. Inscrire le numéro de patient et la date sur le test (ou sur le tube si c'est une bandelette).
10. Ne pas mélanger les réactifs/ le matériel de différents tests rapides.

11. Certains tampons (diluants du test) contiennent de l'azide comme conservateur. Eviter tout contact avec la peau.
12. Ne jamais remplacer le tampon par un autre liquide.
13. Ne pas réutiliser un test déjà utilisé.
14. Eliminer les tests utilisés, les échantillons et tout autre matériel potentiellement contaminé dans les récipients à déchets « bio-hazard »

#### 3.2.1.2 Procédures spécifiques pour la manipulation et la conservation des tests rapides de NIDIAG

1. Pour chaque test index, se référer à la SOP NIDIAG correspondante.

### 3.2.2 Conservation des tests rapides

#### 3.2.2.1 Conditions générales de conservation

1. Conserver les tests dans un endroit sec et frais, à l'abri de la lumière directe du soleil.
2. Conserver les tests à la température indiquée par le fabriquant.
3. Ne pas congeler les tests ou les réactifs fournis avec les tests.
4. Certains tests peuvent être conservés au frigo. Laisser le test atteindre la température ambiante avant l'emploi.

#### 3.2.2.2 Conditions spécifiques de conservation des tests index

1. Conserver les tests index dans une armoire fermée à clé.
2. Restreindre l'accès au personnel de l'étude.
   Note: Les tests index doivent être utilisés uniquement pour les patients inclus dans les études NIDIAG.
3. Conserver les tests index à une température comprise dans la fourchette indiquée dans le tableau 1. Eviter le plus possible de dépasser les valeurs supérieures.
4. Vérifier la température de conservation 2 fois par jour. Relever les valeurs de températures à l'aide du formulaire « Relevé de température » en **annexe 1**. Si les tests index sont conservés au réfrigérateur, utiliser la SOP-WP6-QUAL-06-annexe 1 pour enregistrer les températures.
   Note: Se référer à la **SOP-WP6-QUAL-06** pour l'installation et l'utilisation du thermomètre Min-Max. La sonde du thermomètre peut, mais ne doit pas nécessairement être immergée dans un tube d'eau.
5. Inscrire la température de la pièce dans le CRF (formulaire tests index) au moment où le test est réalisé.

**Tableau 1. Fourchette des températures de conservation des tests index**

| Test index | Fourchette de température |
|---|---|
| Gambiense-Sero-K-Set (Coris Bioconcept) | 4°C - 30°C |
| HAT FIND dipstick (Standard Diagnostics) | 1 - 40°C |
| CATT R250 | Antigène CATT:<br>- lyophilisé: 2 - 8°C ou -20°C<br>- reconstitué: 2 - 8°C (1 semaine) ou 37°C (8 heures)<br>Tampon CATT: 2 - 8°C.<br>Contrôle positif/négatif lyophilisé: 2 - 8°C ou -20°C |
| Malaria Antigen P.f/Pan SDFK60 (Standard Diagnostics) | 1 - 40°C |
| Carestart malaria pLDH (Access Bio) | 4 - 30°C |
| SD Syphilis 3.0 (Standard Diagnostics) | Température ambiante |
| CrAg Lateral Flow Assay (IMMY) | Température ambiante (22 - 25°C) |
| Typhidot Rapid IgM (Reszon Diagnostics) | 2 - 28°C |
| Test-it Typhoid IgM (Life Assay) | 4 - 28°C |
| Test-it Leptospira IgM (Life Assay) | 4 - 28°C |
| Salmonella typhi IgG/IgM (Standard Diagnostics) | 2 - 30°C |
| Leptospira IgG/IgM (Standard Diagnostics) | 1 - 30°C |

### 3.2.3 Gestion des stocks de tests rapides et de l’inventaire

#### 3.2.3.1 Formulaire d’utilisation des tests index

1. Pour chaque test index, indiquer quel numéro de lot a été utilisé pour quel patient dans le formulaire d’utilisation des tests index (**Annexe 2**).
2. Maintenir le formulaire à jour à chaque changement de lot.
3. Conserver le formulaire dans le « Laboratory File ».

#### 3.2.3.2 Carte de stock et inventaire mensuel

1. Utiliser une carte de stock par lot en respectant la **SOP-WP6-QUAL-07**.
2. Compter le nombre de tests restant à la fin de chaque mois et remplir l’inventaire mensuel en fonction. Se référer à la **SOP-WP6-QUAL-07** et à son **Annexe 2.**

## 4. Documents et archives

| **Annexes et formulaires** | |
|---|---|
| **Nom / référence** | **Titre** |
| CRF Neuro et/ou Fièvre | |

| | |
|---|---|
| SOP-WP5-QUAL-05-V1.1-20Aug2013-annex1 | Feuille d'enregistrement quotidien - Température ambiante |
| SOP-WP5-QUAL-05-V1.1-20Aug2013-annex2 | Formulaire d'utilisation des tests index |
| SOP-WP6-QUAL-06-V01-24Sep2012-annex1 | Feuille d'enregistrement quotidien - Réfrigérateur |
| SOP-WP6-QUAL-07-V1.1-04Feb2013-annex1 | Carte de stock |
| SOP-WP6-QUAL-07-V1.1-04Feb2013-annex2 | Inventaire mensuel |

## 5. Historique des modifications

| **Révision** | |
|---|---|
| SOP-WP6-QUAL-05-V01-08Feb2013 | Version initiale |
| SOP-WP6-QUAL-05- V1.1-20Aug2013 | - Traduction en Français<br>- Adaptation du tableau 1:<br>- Ajout du CATT R250<br>- Adaptation de la température de stockage du test index rk28<br>- Addition des tests index qui sont utilisés pour l'étude neurologique en RDC |

| **Nom et fonction** | **Date** | **Signature** |
|---|---|---|
| Auteur | | |
| Barbara Barbé | 20/08/2013 | |
| Revue et approuvé par | | |
| Ninon Horié | 28/08/2013 | |

# HOPITAL GENERALE DE REFERENCE, MOSANGO, LABORATOIRE

**Feuille d'enregistrement quotidien de la température (Température ambiante)**

**Mois/Année:______________________ Localisation:_____________________**

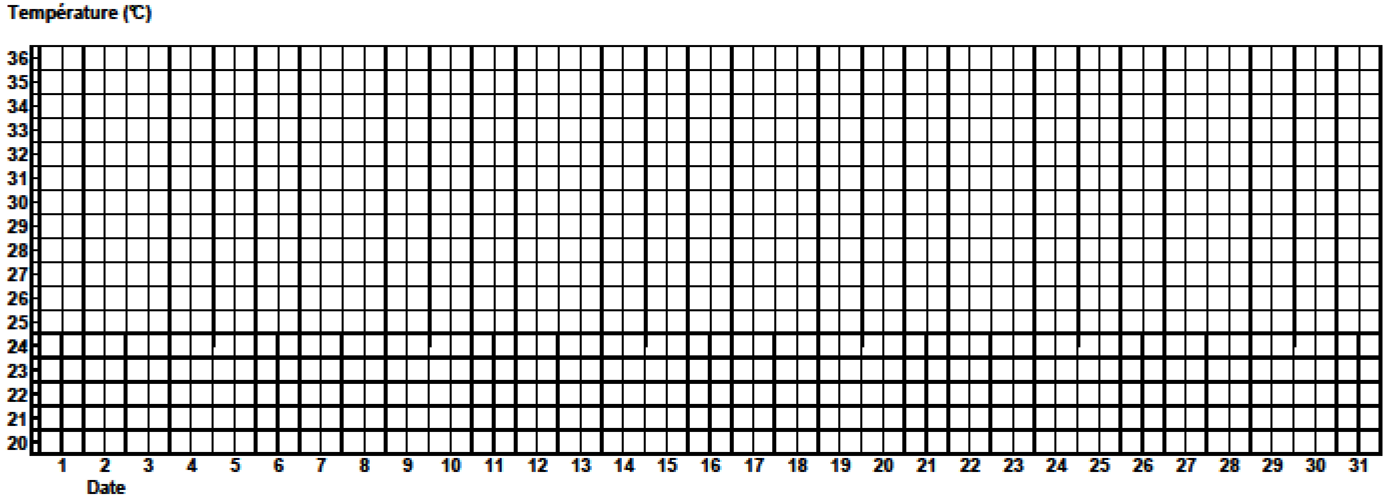


| Date | 1 | 2 | 3 | 4 | 5 | 6 | 7 | 8 | 9 | 10 | 11 | 12 | 13 | 14 | 15 | 16 | 17 | 18 | 19 | 20 | 21 | 22 | 23 | 24 | 25 | 26 | 27 | 28 | 29 | 30 | 31 |
|---|---|---|---|---|---|---|---|---|---|---|---|---|---|---|---|---|---|---|---|---|---|---|---|---|---|---|---|---|---|---|---|
| MIN T (℃) | | | | | | | | | | | | | | | | | | | | | | | | | | | | | | | |
| MAX T (℃) | | | | | | | | | | | | | | | | | | | | | | | | | | | | | | | |
| INITIALES LAB TECH | | | | | | | | | | | | | | | | | | | | | | | | | | | | | | | |

**Rapport d'action:**

| Date | Mesure prise | Résultat | Technicien de laboratoire | Remarque(s) |
|---|---|---|---|---|
| | | | | |
| | | | | |
| | | | | |

**Revisé par:________________**

SOP-WP6-QUAL05-Annex1(French)

# Formulaire d'Utilisation des Tests Index

**Site d'Etude :**

**Nom de l'Investigateur Principal :** **Nom du responsable du laboratoire :**

- **Gambiense-Sero-K-Set (Coris Bioconcept)**

Le lot n° ________________ a été utilisé du patient n° ☐☐☐☐☐-☐☐ au patient n° ☐☐☐☐☐-☐☐

Le lot n° ________________ a été utilisé du patient n° ☐☐☐☐☐-☐☐ au patient n° ☐☐☐☐☐-☐☐

Le lot n° ________________ a été utilisé du patient n° ☐☐☐☐☐-☐☐ au patient n° ☐☐☐☐☐-☐☐

- **HAT FIND dipstick (Standard Diagnostics)**

Le lot n° ________________ a été utilisé du patient n° ☐☐☐☐☐-☐☐ au patient n° ☐☐☐☐☐-☐☐

Le lot n° ________________ a été utilisé du patient n° ☐☐☐☐☐-☐☐ au patient n° ☐☐☐☐☐-☐☐

Le lot n° ________________ a été utilisé du patient n° ☐☐☐☐☐-☐☐ au patient n° ☐☐☐☐☐-☐☐

☐ NA (test non disponible)

- **CATT R250**

Le lot n° ________________ a été utilisé du patient n° ☐☐☐☐☐-☐☐ au patient n° ☐☐☐☐☐-☐☐

Le lot n° ________________ a été utilisé du patient n° ☐☐☐☐☐-☐☐ au patient n° ☐☐☐☐☐-☐☐

Le lot n° ________________ a été utilisé du patient n° ☐☐☐☐☐-☐☐ au patient n° ☐☐☐☐☐-☐☐

| | |
|---|---|
| nidiag Better Diagnosis of Neglected Infectious Diseases | **SOP titre :** Comment installer et utiliser le thermomètre « Min-Max » ? |
| | **Project/study:** Evaluation of Rapid Diagnostic Tests in association with clinical and laboratory predictors for the diagnosis of Neglected Tropical Diseases in patients presenting with neurological disorders in rural hospitals of Bandundu, Democratic Republic of Congo. |

# 1. Domaine et application

Ce SOP d'écrit l'installation et l'utilisation d'un thermomètre afin d'assurer le monitoring des températures et de permettre le relèvement quotidien des températures réelles, maximales et minimales d'un réfrigérateur, congélateur, incubateur ou bain marie afin de s'assurer de sa performance. Une sonde est immergée dans un liquide (eau pour les frigos, incubateurs ou bain marie ; glycérine pour les congélateurs) pour temporiser l'enregistrement des fluctuations de température à court terme.

# 2. Responsabilités

| Fonction | Activités |
|---|---|
| Technicien de labo / responsable de qualité | Installe le thermomètre<br>Relève les températures<br>Enregistre les températures |

# 3. Procédures

## 3.1 Matériel

- Thermomètre min/max -50/+70°C
- Eau / Glycérine
- Tube à essai
- Colle ou scotch

## 3.2 Procédure

### 3.2.1. Préparation du thermomètre

1. Installez la batterie du thermomètre en respectant la polarité.
   **(Si rien ne s'affiche à l'écran, remplacez la batterie).**
2. Remplissez un tube avec l'eau (frigo/incubateur) ou glycérine (congélateur)

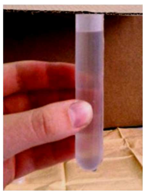

*Figure 1: Remplissez le tube en laissant un peu de place pour la sonde et le bouchon.*

3. Faites un trou dans un bouchon pour pouvoir y faire passer la sonde. Plongez la sonde du thermomètre dans le tube à essai et fermez le bouchon. Collez le tout en évitant des bulles d'air.

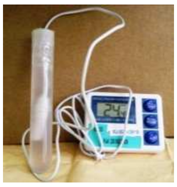

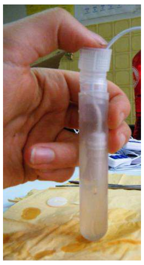

### 3.2.2 Installation du thermomètre

1. Fixez le tube contenant la sonde à l'intérieur, au milieu de l'appareil.
2. Attachez le thermomètre à l'extérieur d'appareil en utilisant les aimants.

### 3.2.3 Prélèvement des températures

1. Vérifiez sur l'écran que la température est affichée en °C ; sinon, sélectionnez "°C" avec le bouton rouge "°C/ °F" à l'arrière du thermomètre.
2. Vérifiez sur l'écran que la température du réfrigérateur est affichée; sinon, sélectionnez "FRIDGE" avec le bouton "ROOM/ FRIDGE". ("ROOM" affiche la température ambiante)

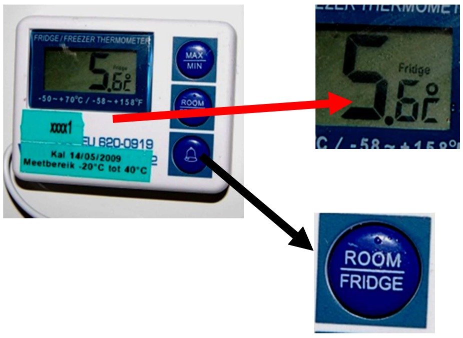


*Figure 2: sélectionnez "FRIDGE" avec le bouton "ROOM/ FRIDGE" pour registrer la température dans le réfrigérateur/congélateur/incubateur.*

3. Selon la procédure notez la température du réfrigérateur deux fois par jour le matin et le soir (annexe 1).
4. Notez également chaque soir les températures maximales et minimales respectivement en les sélectionnant à l'aide du bouton "Min/Max".

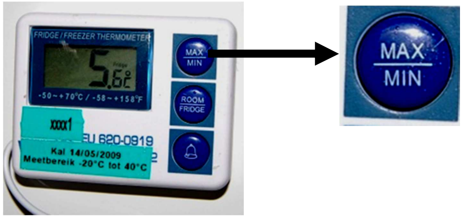


*Figure 3: sélectionnez la température maximale et minimale avec le bouton "Min/Max".*

5. Après avoir noté les températures maximales et minimales, réinitialisez les valeurs min/max à la température actuelle. Enfoncez le bouton "Min/Max" pendant 2 secondes (jusqu'au 2$^{ème}$ bip sonore).

## 5. Enregistrements et archives

| **Appendices & formulaires à compléter** | |
|---|---|
| **Numéro** | **Titre** |
| SOP-WP6-QUAL-06-V01-24Sep2012-annex1 | Feuille d'enregistrement quotidien de la température (Réfrigérateur)<br>Feuille d'enregistrement quotidien de la température (Incubateur) |

## 6. Histoire de document

| **Révision** | |
|---|---|
| SOP-WP6-QUAL-06-V01-19Sep2012 | Version initiale |

| **Nom et fonction** | **Date** | **Signature** |
|---|---|---|
| Auteur | | |
| Philippe Gillet | 18/Sep/2012 | |
| Révision par | | |
| Barbara Barbe | 18/Sep/2012 | |
| Approuvé by | | |
| Veerle Lejon | 24/Sep/2012 | |

## ENDROIT:

**Feuille d'enregistrement quotidien de la température (Réfrigérateur)**

**Mois/Année:_______________________ Réfrigérateur ID:_____________________ (Plage attendue: 2 - 8°C)**

**Localisation:___________________________**

**Température (℃)**

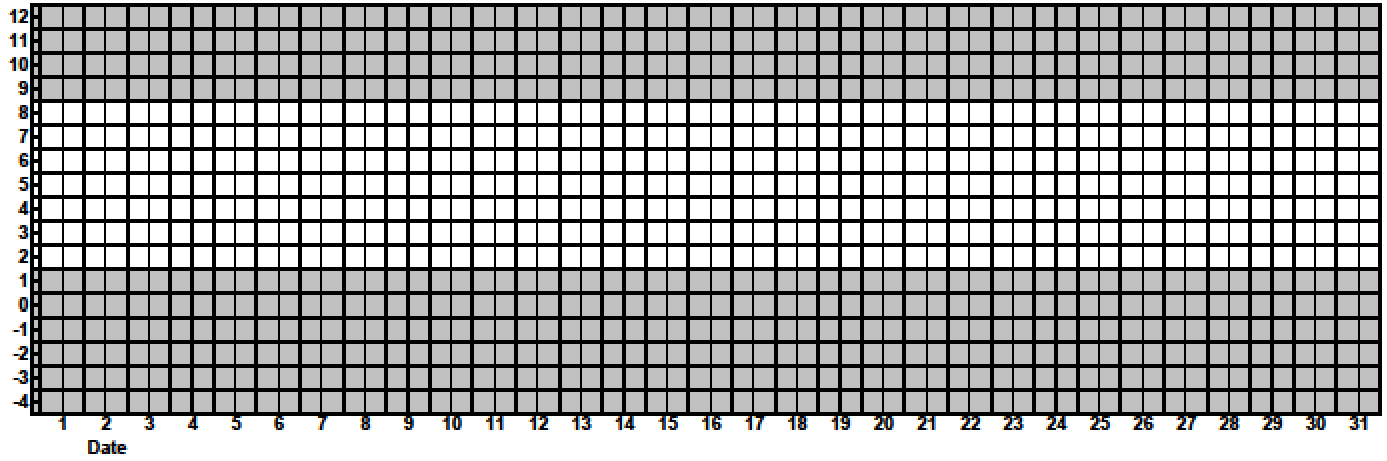


| Date | 1 | 2 | 3 | 4 | 5 | 6 | 7 | 8 | 9 | 10 | 11 | 12 | 13 | 14 | 15 | 16 | 17 | 18 | 19 | 20 | 21 | 22 | 23 | 24 | 25 | 26 | 27 | 28 | 29 | 30 | 31 |
|---|---|---|---|---|---|---|---|---|---|---|---|---|---|---|---|---|---|---|---|---|---|---|---|---|---|---|---|---|---|---|---|
| MIN T (℃) | | | | | | | | | | | | | | | | | | | | | | | | | | | | | | | |
| MAX T (℃) | | | | | | | | | | | | | | | | | | | | | | | | | | | | | | | |
| INITIALES LAB TECH | | | | | | | | | | | | | | | | | | | | | | | | | | | | | | | |

**Rapport d'action:**

| Date | Mesure prise | Résultat | Technicien de laboratoire | Remarque(s) |
|---|---|---|---|---|
| | | | | |
| | | | | |
| | | | | |

**Revisé par:________________**

| | |
|---|---|
| | **SOP titre :** Gestion du stock |
| | **Project/study:** Evaluation of Rapid Diagnostic Tests in association with clinical and laboratory predictors for the diagnosis of Neglected Tropical Diseases in patients presenting with neurological disorders in rural hospitals of Bandundu, Democratic Republic of Congo. |

# 1. Domaine et application

Ce SOP décrit le suivi des réactifs, des consommables et des équipements à réaliser pour NIDIAG .

# 2. Responsabilités

| Fonction | Activités |
|---|---|
| Technicien de labo / responsable de qualité | Gère le stock.<br>Enregistre les entrées et les sorties du stock.<br>Réalise mensuellement un inventaire physique. |

# 3. Procédures

## 3.1 Matériel

- Fiche de stock (cf. SOP-WP6-QUAL-07-V01-24sep2012-annex1)
- Inventaire (document manuscrit ou informatique (cf. SOP-WP6-QUAL-07-V01-24sep2012-annex2.xlsx)

## 3.2 Procédure

1. Notez toute entrée de réactifs, consommables ou équipement sur les fiches de stock. **Une fiche de stock par produits et par date de péremption.**
2. Notez toute sortie de réactifs ou consommables. Les sorties du stock vers le laboratoire ne se font que par conditionnement complet (exemple pour les tests malaria : le conditionnement est de 25 tests par boite, les sorties ne se font que par boites)
4. Réalisez à la fin de chaque mois un inventaire des réactifs et des consommables. Cet inventaire peut être soit manuscrit sur base d'un impression du document SOP-WP6-QUAL-07-V01-annex2.xlsx, soit informatique sur base du même document.
5. Envoyez chaque mois l'état du stock (soit sous format manuscrit soit sous format informatique).

# 5. Enregistrements et archives

| Appendices & formulaires à compléter | |
|---|---|
| **Numéro** | **Titre** |
| SOP-WP6-QUAL-07-V01-24Sep2012-annex2.xlsx | Document excel pour le suivi du stock |
| SOP-WP6-QUAL-07- V01-24Sep2012-annex1.docx | Fiche de stock |

## 6. Histoire de document

| **Révision** | |
|---|---|
| SOP-WP6-QUAL-07-V01-24Sep2012 | Version initiale |

| **Nom et fonction** | **Date** | **Signature** |
|---|---|---|
| Auteur | | |
| Philippe Gillet | 18/Sep/2012 | |
| Révision par | | |
| Barbara Barbe | 18/Sep/2012 | |
| Approuvé by | | |
| Veerle Lejon | 19/Sep/2012 | |

nidiag

## FICHE DE STOCK (SOP-WP6-QUAL-V01-24sep2012-annex1)

| | | | |
|---|---|---|---|
| **Description de l'article** | ............................................................................................................ | | |
| **N° de LOT** | .................................... | **Date de péremption** | .................................... |
| **Conditionnement (unités/emballage)** | .................................... | **Commentaires** | .................................... |

| DATE | IN (unités) | OUT (unités) | STOCK (unités) | Commentaires | Initiales Signature |
|---|---|---|---|---|---|
| STOCK TRANSFÉRÉ DE LA FICHE PRÉCÉDENTE | | | | | |
| | | | | | |
| | | | | | |
| | | | | | |
| | | | | | |
| | | | | | |
| | | | | | |
| | | | | | |
| | | | | | |
| | | | | | |
| | | | | | |
| | | | | | |
| | | | | | |
| | | | | | |
| | | | | | |
| | | | | | |
| | | | | | |
| | | | | | |
| | | | | | |
| | | | | | |
| | | | | | |
| | | | | | |
| | | | | | |
| | | | | | |
| | | | | | |

nidiag

**Gestion de stock**
**Consommation mensuelle**

**MOIS:**

| | Déscription de l'article | Numéro de lot | Date de péremption JJ/MM/AAAA | Conditionnement | Stock initial du mois Date: / / | Stock reçu pendant le mois Date: / / | Stock final du mois Date: / / | Consommation mensuelle | Commentaires |
|---|---|---|---|---|---|---|---|---|---|
| 1 | Aiguille BD Vacutainer Precision glide 21G | | / / | | | | | | |
| 2 | Aiguille ponction lombaire adulte, 22G | | / / | | | | | | |
| 3 | Aiguille ponction lombaire pédiatrique, Fino-Ject, 25G | | / / | | | | | | |
| 4 | BactAlert blood collection adapter insert (pour tubes) | | / / | | | | | | |
| 5 | BactAlert blood collection adaptor cap (pour hémocultures) | | / / | | | | | | |
| 6 | BactAlert FA (adultes) | | / / | | | | | | |
| 7 | BactAlert PF (pédiatrique) | | / / | | | | | | |
| 8 | Batteries, type AA, 1.5 V | | / / | | | | | | |
| 9 | Batteries, type C (LR14,HR14), 1.5 V | | / / | | | | | | |
| 10 | BD vacutainer K2EDTA, 6 ml (bouchon pourpre) | | / / | | | | | | |
| 11 | BD vacutainer LH (héparine),4 ml (bouchon vert) | | / / | | | | | | |
| 12 | Blood collection adaptor cap (pour hémocultures): Terumo Venoject Quick Fit Holder | | / / | | | | | | |
| 13 | Bouchon BLEU | | / / | | | | | | |
| 14 | Bouchon INCOLORE | | / / | | | | | | |
| 15 | Bouchon JAUNE | | / / | | | | | | |
| 16 | Bouchon ROUGE | | / / | | | | | | |
| 17 | Bouchon VERT | | / / | | | | | | |
| 18 | Bouchon VIOLET | | / / | | | | | | |
| 19 | CARESTART malaria pLDH 3 lines (Pan/P.f) | | / / | | | | | | |
| 20 | Cartouche à gaz C206 pour camping gaz | | / / | | | | | | |
| 21 | CATT accessoire kit (250 tests) T. gambiense | | / / | | | | | | |
| 22 | CATT D10 accessoire kit, T. gambiense | | / / | | | | | | |
| 23 | CATT D10 kit réactifs, T. gambiense | | / / | | | | | | |
| 24 | CATT kit réactifs (250 tests) T. gambiense | | / / | | | | | | |
| 25 | Chase buffer, Alere (pour test VIH Determine) | | / / | | | | | | |
| 26 | Compresses stériles | | / / | | | | | | |
| 27 | Comprimés de tampon, pH 7.2 | | / / | | | | | | |
| 28 | Conteneur à aiguille | | / / | | | | | | |
| 29 | Conteneur pour l'urine, 60 ml (stérile) | | / / | | | | | | |
| 30 | CrAg lateral flow assay | | / / | | | | | | |

| | |
|---|---|
| nidiag Better Diagnosis of Neglected Infectious Diseases | **SOP titre :** Gestion des produits périmés et déclassés |
| | **Project/study:** Cette POS est applicable pour toutes les etudes du projet NIDIAG |

# 1. Domaine et application

Ce document fournit les instructions pour la gestion des produits périmés et déclassés (médicaments, réactifs de laboratoire, kits TDR, etc.) dans les études du projet NIDIAG.

# 2. Responsabilités

| Fonction | Activités |
|---|---|
| Technicien de laboratoire | ▪ Collecter tous les produits périmés et déclassés du laboratoire.<br>▪ Centraliser tous les produits périmés et déclassés du laboratoire dans le stock central du site (bureau PI du site). |
| Investigateur principale du site | ▪ Collecter tous les médicaments périmés.<br>▪ Centraliser tous les médicaments périmés dans le stock central du site (bureau PI du site).<br>▪ Assurer que tous les produits périmés et déclassés sont dans une boîte marquée « hors usage ».<br>▪ Envoyer les produits périmés et déclassés du laboratoire et les médicaments périmés à l'INRB Kinshasa avec le véhicule. |
| Investigateur principale du pays | ▪ Réceptionner et classer les produits périmés et déclassés au stock central à l'INRB (bureau PI du pays) comme « HORS USAGE » jusqu'à la fin de l'étude NIDIAG.<br>▪ Compléter le registre des produits périmés et déclassés.<br>▪ Maintenir à jour le registre des produits périmés et déclassés. |

# 3. Procédures

## 3.1 Procédure au site (Mosango)

- Vérifiez toujours la date d'expiration du produit avant de l''utiliser (réactif du laboratoire, kit TDR, médicament, etc.).
- S'il est périmé ou déclassé, n'utilisez pas ce produit ! Stockez le produit dans une boîte où il est inscrit « HORS USAGE ! » dans le stock central du site (bureau PI du site).
- A la fin du mois, envoyez le(s) boîte(s) avec les produits périmés et déclassés à l'INRB Kinshasa par véhicule.

## 3.2 Procédure à l'INRB

- Compléter un registre pour des produits périmés et déclassés (Voir exemple en **Annexe 1**).
- Réceptionnez les produits périmés et déclassés. Mettez le registre à jour après chaque réception.
- Stockez les produits au stock central (bureau du PI) dans des boîtes marquées «HORS USAGE ! »
- Notez la raison pour déclasser les produits comme "Hors usage" dans le registre sous "Raison" (ex. produit périmé, produit avec qualité inférieure (se référer à un rapport)).

- Si des produits périmés sont utilisés pour l'enseignement ou pour une formation, notez-le dans le registre sous « Commentaires ».
- Détruisez les produits périmés et déclassés conformément aux procédures de gestion des déchets locaux à la fin de l'étude NIDIAG.

## 4. Enregistrements et archives

| **Appendices & formulaires à compléter** | |
|---|---|
| **Numéro** | **Titre** |
| SOP-WP6-QUAL-08-V1.1-Annexe 1 | Registre des produits périmés et déclassés |

## 5. Histoire du document

| **Révision** | |
|---|---|
| **SOP-WP6-QUAL-08-V1.1-05Mar2014** | Version initiale |

| **Nom et fonction** | **Date** | **Signature** |
|---|---|---|
| *Auteur* | | |
| Barbara Barbé | 05/03/2014 | |
| *Révisé par* | | |
| Jan Jacobs | 05/03/2014 | |
| *Approuvé par* | | |
| Ninon Horié | 07/03/2014 | |

# Registre des produits périmés et déclassés

| Nom du produit | Numéro de lot | Date d'expiration | Condition-nement | Quantité | Date de réception | Raison* | Commentaire |
|---|---|---|---|---|---|---|---|
| | | | | | | | |
| | | | | | | | |
| | | | | | | | |
| | | | | | | | |
| | | | | | | | |
| | | | | | | | |
| | | | | | | | |
| | | | | | | | |
| | | | | | | | |
| | | | | | | | |
| | | | | | | | |
| | | | | | | | |
| | | | | | | | |
| | | | | | | | |
| | | | | | | | |
| | | | | | | | |
| | | | | | | | |
| | | | | | | | |
| | | | | | | | |
| | | | | | | | |

**ex.* Produit périmé, produit avec qualité inférieure (se référer à un rapport)
