# Supplementary material for: The Art of Writing and Implementing Standard Operating Procedures (SOPs) for Laboratories in Low-Resource Settings: Review of Guidelines and Best Practices
Source: PLoS Negl Trop Dis. 2016 Nov 3;10(11):e0005053. doi: 10.1371/journal.pntd.0005053 (PMC5094690; doi:10.1371/journal.pntd.0005053)
Supplement: S2 Appendix — (PDF) [file pntd.0005053.s002.pdf]

**Overview of the laboratory Quality Management System (QMS) and other documents assessed for the writing and implementation of SOPs**

Quality Management System (QMS) documents are categorized as Standards, Guidelines or Regulatory documents (see Table 1 for the list of assessed QMS documents) [9]. Standards are consensus documents that simply list up all requirements without further explanation or guidance to implementation – a well-known example in clinical laboratories is ISO 15189. In addition to providing the QMS requirements, guidelines also explain and describe how to integrate them into the laboratory, an example is CLSI QMS01-A4. Regulations are standards imposed by regulatory authorities (*e.g.* the Ministry of Health or authorized body) at the national level. Most regulations are based on international standards but may differ in stringency and focus: an example is the CLIA in the United States [31].

The **Clinical and Laboratory Standards Institute (CLSI)** (http://clsi.org/) is a non-profit membership organization that facilitates the development of clinical laboratory testing standards based on input from and consensus among industry, government, and health care professionals. **CLSI QMS01-A4** from 2011 (formerly GP26-A4) entitled “Quality Management System: A model for laboratory services; Approved guideline” combines requirements of several QMS documents including ISO 15819 and in turn has been adopted by other QMS documents (*e.g.* NIH DAIDS guidelines for GCLP standards, version 3.0, 2013). **CLSI QMS02-A6** from 2013 entitled “Quality Management System: Development and management of laboratory documents; Approved guideline” presents evidence based suggestions for the preparation of different types of laboratory documents, their management and control.

The **International Organization for Standardization (ISO)** (http://www.iso.org/iso/home.html) is an independent, non-governmental international organization, existing of 162 national standards bodies, that develops consensus-based International Standards. To date, they have published over 21.000 International Standards. **ISO 17025** “General requirements for the competence of testing and calibration laboratories” was the first internationally published standard on laboratory quality management in 1978. The version used here is from 2005. **ISO 15189** “Medical laboratories — Requirements for quality and competence” is one of the most widely used quality standards worldwide, on which regional and international accreditation organizations base their accreditation requirements [9].

The **Joint Commission International (JCI)** (http://www.jointcommissioninternational.org/) is the oldest and largest standards-setting and accrediting body in health care in the United States. It identifies, measures, and shares best practices in quality and patient safety worldwide. The JCI published its second edition of “**Accreditation standards for clinical laboratories**” in 2010. It is a highly elaborate document that provides quality assurance and quality control standards for laboratory practice.

The **Strengthening Laboratory Management Toward Accreditation (SLMTA)** (https://slmta.org/) is a structured quality improvement program for resource-limited settings. It was launched in 2009 and consists of a series of short courses and work-based improvement projects, supported by site visits and mentoring. It provides a toolkit (https://slmta.org/tool-kit/english) consisting of different modules aiming to strengthen laboratory management, achieve immediate laboratory improvement and accelerate the preparedness towards accreditation in accordance with the Stepwise Laboratory Quality Improvement Process Towards Accreditation (SLIPTA) program from the Regional Office for Africa of the World Health Organization (WHO-AFRO).

The **World Health Organization (WHO)** (http://www.who.int/en/) directs and coordinates international health within the United Nations’ system. WHO’s “Special Programme for Research and

Training in Tropical Diseases" (TDR) adopted the Principles on Good Laboratory Practice (GLP) developed by the Organization for Economic Co-operation and Development (OECD) from 1981 (and revised in 1998) and included them into a reference and support document in 2001 (and revised in 2009) called "**Good Laboratory Practice (GLP). Quality practices for regulated non-clinical research and development**". This document offers a GLP training, a guide to the stepwise implementation of GLP and, as annex, the OEDC principles of GLP and related guidance documents.
WHO also translated the "**Good Clinical Laboratory Practice (GCLP)**" document developed by the Clinical Committee of the British Association of Research Quality Assurance (BARQA) from 2002 (revised in 2012) into a WHO document in 2009 to make it widely available. In 2011, WHO published a handbook on "**Laboratory Quality Management System**"  based on training sessions and modules provided by U.S. Centers for Disease Control and Prevention (CDC) and WHO, and on CLSI guidelines for implementation of ISO 15189 (CLSI GP26-A3).

The Enterprise and Industry Directorate-General from the **European Commission (EC)** (http://ec.europa.eu/index_en.htm) published guideline **ENTR/F/2/SF/jr (2009)D/869** on the readability of the labelling and package leaflet of medicinal products for human use in 1988, with a revision made in 2009. The recommendations about readability and legibility of the package leaflet can be extrapolated to other documents such as SOPs. The guideline also provides specific recommendations for the blind and partially-sighted and describes how to consult patient groups for the evaluation of comprehensibility of the package leaflet.

The **U.S. Food and Drug Administration (FDA)** (http://www.fda.gov/) provided a guideline in 1993 "Write it right - Recommendations for developing user instruction manuals for medical devices used in home health care", and a second one in 2001 "Guidance on medical device patient labeling; Final guidance for industry and FDA reviewers". Both guidelines include detailed information on readability, design, lay-out and pretesting of the developed documents or labelling. The second document provides this information in its annexes, while the main document gives recommendations on the content of medical device patient labeling.
