# Supplementary material for: The Art of Writing and Implementing Standard Operating Procedures (SOPs) for Laboratories in Low-Resource Settings: Review of Guidelines and Best Practices
Source: PLoS Negl Trop Dis. 2016 Nov 3;10(11):e0005053. doi: 10.1371/journal.pntd.0005053 (PMC5094690; doi:10.1371/journal.pntd.0005053)
Supplement: S1 Fig — (PDF) [file pntd.0005053.s003.pdf]

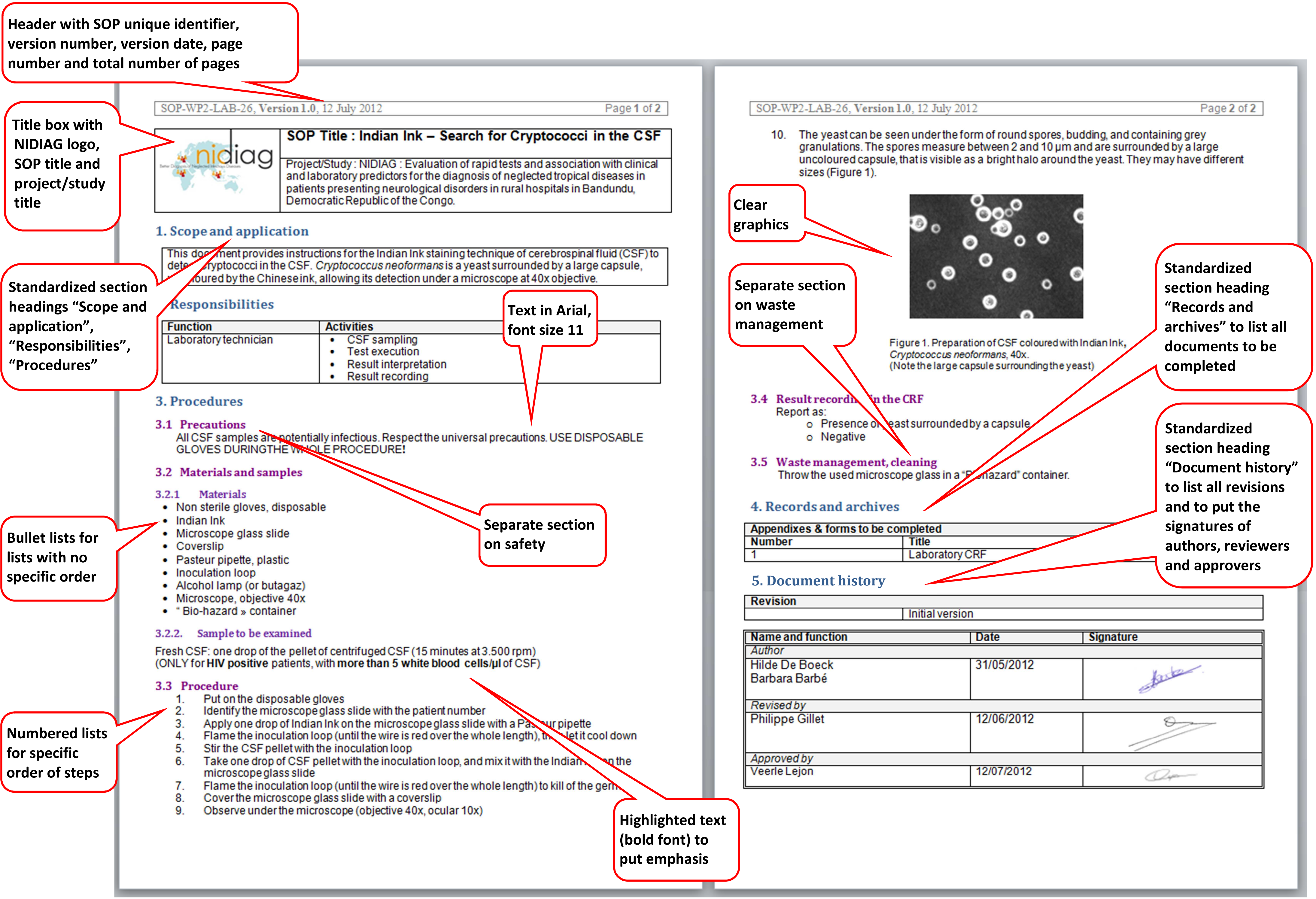

nidiag

SOP Title : Indian Ink – Search for Cryptococci in the CSF

Project/Study : NIDIAG : Evaluation of rapid tests and association with clinical and laboratory predictors for the diagnosis of neglected tropical diseases in patients presenting neurological disorders in rural hospitals in Bandundu, Democratic Republic of the Congo.

1. Scope and application

This document provides instructions for the Indian Ink staining technique of cerebrospinal fluid (CSF) to detect cryptococci in the CSF. *Cryptococcus neoformans* is a yeast surrounded by a large capsule, coloured by the Chinese ink, allowing its detection under a microscope at 40x objective.

Responsibilities

Function	Activities
Laboratory technician	• CSF sampling<br>• Test execution<br>• Result interpretation<br>• Result recording

3. Procedures

3.1 Precautions

All CSF samples are potentially infectious. Respect the universal precautions. USE DISPOSABLE GLOVES DURING THE WHOLE PROCEDURE!

3.2 Materials and samples

3.2.1 Materials

- Non sterile gloves, disposable
- Indian Ink
- Microscope glass slide
- Coverslip
- Pasteur pipette, plastic
- Inoculation loop
- Alcohol lamp (or butagaz)
- Microscope, objective 40x
- « Bio-hazard » container

3.2.2. Sample to be examined

Fresh CSF: one drop of the pellet of centrifuged CSF (15 minutes at 3.500 rpm)
(ONLY for **HIV positive** patients, with **more than 5 white blood cells/µl** of CSF)

3.3 Procedure

1. Put on the disposable gloves
2. Identify the microscope glass slide with the patient number
3. Apply one drop of Indian Ink on the microscope glass slide with a Pasteur pipette
4. Flame the inoculation loop (until the wire is red over the whole length), then let it cool down
5. Stir the CSF pellet with the inoculation loop
6. Take one drop of CSF pellet with the inoculation loop, and mix it with the Indian [illegible] on the microscope glass slide
7. Flame the inoculation loop (until the wire is red over the whole length) to kill of the germ[illegible]
8. Cover the microscope glass slide with a coverslip
9. Observe under the microscope (objective 40x, ocular 10x)

10. The yeast can be seen under the form of round spores, budding, and containing grey granulations. The spores measure between 2 and 10 µm and are surrounded by a large uncoloured capsule, that is visible as a bright halo around the yeast. They may have different sizes (Figure 1).

Figure 1. Preparation of CSF coloured with Indian Ink, *Cryptococcus neoformans*, 40x.
(Note the large capsule surrounding the yeast)

3.4 Result recording in the CRF

Report as:

- Presence of yeast surrounded by a capsule
- Negative

3.5 Waste management, cleaning

Throw the used microscope glass in a "Biohazard" container.

4. Records and archives

Appendixes & forms to be completed	
**Number**	**Title**
1	Laboratory CRF

5. Document history

Revision	
	Initial version

Name and function	Date	Signature
Author		
Hilde De Boeck<br>Barbara Barbé	31/05/2012	[signature]
Revised by		
Philippe Gillet	12/06/2012	[signature]
Approved by		
Veerle Lejon	12/07/2012	[signature]
