# Supplementary figures and images for: The Art of Writing and Implementing Standard Operating Procedures (SOPs) for Laboratories in Low-Resource Settings: Review of Guidelines and Best Practices
Source: PLoS Negl Trop Dis. 2016 Nov 3;10(11):e0005053. doi: 10.1371/journal.pntd.0005053 (PMC5094690; doi:10.1371/journal.pntd.0005053)

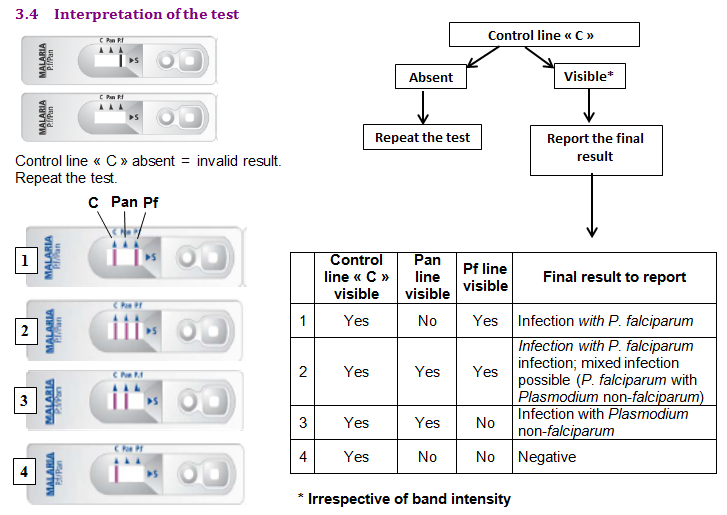

Supplement: S2 Fig — Clear drawings, a flowchart, and a decision table are used. The SOP is based on the generic WHO job aid of a malaria RDT (http://www.who.int/malaria/areas/diagnosis/rapid-diagnostic-tests/generic_PfPan_training_manual_web.pdf). (TIF) [file pntd.0005053.s004.tif]

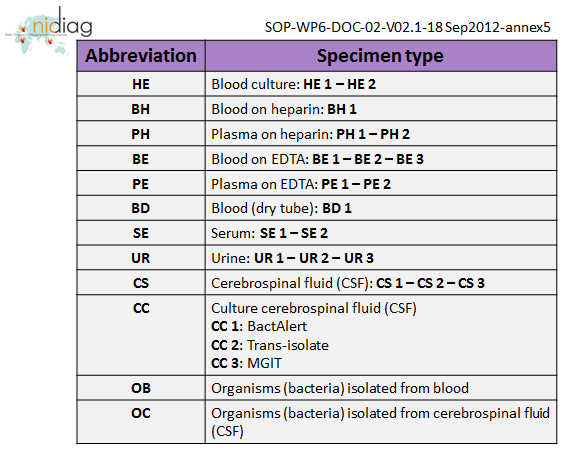

Supplement: S3 Fig — This job aid represents a table summarizing the NIDIAG study specimen numbering information extracted from SOP-WP6-DOC-02-V02.1-18Sep2012, and was put as its annex five. The job aid has a clear title linking it to an approved SOP, thus making it subject to document control. Font type Calibri, and type sizes of 18 and 24 points were used for table text and headers, respectively, allowing the text to be easily read when printed out and posted on a wall. The “bold” font style was appropriately used to highlight the column titles and abbreviations. (TIF) [file pntd.0005053.s005.tif]
